# Mitochondrial related genome-wide Mendelian randomization identifies putatively causal genes for multiple cancer types

**DOI:** 10.1016/j.ebiom.2022.104432

**Published:** 2023-01-10

**Authors:** Yanni Li, Kristina Sundquist, Naiqi Zhang, Xiao Wang, Jan Sundquist, Ashfaque A. Memon

**Affiliations:** a Center for Primary Health Care Research, Lund University/Region Skåne, Malmö, Sweden; b Department of Family Medicine and Community Health, Icahn School of Medicine at Mount Sinai, New York, United States; c Department of Population Health Science and Policy, Icahn School of Medicine at Mount Sinai, New York, United States; d Center for Community-Based Healthcare Research and Education (CoHRE), Department of Functional Pathology, School of Medicine, Shimane University, Matsue, Japan

**Keywords:** Mendelian randomization, Mitochondrial dysfunction, Cancers, Colocalization, Pharmaceutical targets, MR, Mendelian randomization, SMR, Summary-data-based MR, MtDNA, Mitochondrial DNA, IVs, Instrumental variables, GWAS, Genome-wide association studies, SNPs, Single nucleotide polymorphisms, eQTL, Expression quantitative trait loci, mQTL, Methylation quantitative trait loci, pQTL, Protein quantitative trait loci, HEIDI, Heterogeneity independent instruments, LD, Linkage disequilibrium, CpG, Cytosine-guanine dinucleotides, IVW, Inverse variance weighting, MR-PRESSO, MR Pleiotropy Residual Sum and Outlier

## Abstract

**Background:**

Mitochondrial dysfunction is a hallmark of cancer. However, it is unclear whether it is a cause of cancer. This two-sample Mendelian randomization (MR) analyses, uses genetic instruments to proxy the exposure of mitochondrial dysfunction and cancer summary statistics as outcomes, allowing for causal inferences.

**Methods:**

Summary statistics from 18 common cancers (2107–491,974 participants), gene expression, DNA methylation and protein expression quantitative trait loci (eQTL, mQTL and pQTL, respectively, 1000–31,684 participants) on individuals of European ancestry, were included. Genetic variants located within or close to the 1136 mitochondrial-related genes (in *cis*) and robustly associated with the mitochondrial molecular alterations were used as instrumental variables, and their causal associations with cancers were examined using summary-data-based MR (SMR) analyses. An additional five MR methods were used as sensitivity analyses to confirm the casual associations. A Bayesian test for colocalization between mitochondrial molecular QTLs and cancer risk loci was performed to provide insights into the potential regulatory mechanisms of risk variants on cancers.

**Findings:**

We identified potential causal relationships between mitochondrial-related genes and breast, prostate, gastric, lung cancer and melanoma by primary SMR analyses. The sensitivity and the colocalization analyses further refined four genes that have causal effects on three types of cancer. We found strong evidence of positive association of *FDPS* expression level with breast cancer risk (OR per SD, 0.66; 95% CI, 0.49–0.83; *P* = 9.77 × 10^−7^), *NSUN4* expression level with both breast cancer risk (OR per SD, 1.05; 95% CI, 1.03–1.07; *P* = 5.24 × 10^−6^) and prostate cancer risk (OR per SD, 1.06; 95% CI, 1.03–1.09; *P* = 1.01 × 10^−5^), *NSUN4* methylation level with both breast and prostate cancer risk, and *VARS2* methylation level with lung cancer risk.

**Interpretations:**

This data-driven MR study demonstrated the causal role of mitochondrial dysfunction in multiple cancers. Furthermore, this study identified candidate genes that can be the targets of potential pharmacological agents for cancer prevention.

**Funding:**

This work was supported by Styrelsen för 10.13039/501100009736Allmänna Sjukhusets i Malmö Stiftelse för bekämpande av cancer (20211025).

**Research in context**

**Evidence before this study**

Previous studies have shown associations between dysfunctions in mitochondrial DNA (mtDNA), mtDNA copy number or mitochondrial-related nuclear genes and different cancer risks. However, these studies did not investigate causal inferences between mitochondrial dysfunction and cancers. We searched PubMed for studies in any language using the search terms “mitochondrion OR mitochondria OR mitochondrial dysfunction” AND “Mendelian randomization OR Mendelian randomisation” AND “cancer OR cancers”. Of the yielded 4 studies, three studies’ outcomes were COVID-19, dementia and type 2 diabetes, respectively. The other study was a meta-analysis study that presented heterogeneous estimates for the effect of mtDNA copy numbers on different cancer risks and suggested applying Mendelian randomization for unraveling the casual correlation of mtDNA copy number with cancer risk.

**Added value of this study**

This data-driven study fills the gap by using Mendelian randomization to examine the potential causal relationship between mitochondrial dysfunction characterized by genetic predisposition in all mitochondrial-related genes and common cancer risks. Our findings provide evidence for the potential causal effect of mitochondrial dysfunction on breast, prostate and lung cancer, after sensitivity and colocalization analyses. In addition, we identified a shared putative causal gene, *NSUN4*, for both breast and prostate cancer. All associations underscore the importance of mitochondrial dysfunction in the pathogenesis of multiple cancer types.

**Implications of all the available evidence**

Our data-driven analyses support the increasing values in the application of publicly accessible datasets to inform public health. To date, our European population-based large-scale study and the available evidence, indicate that individuals with mitochondrial dysfunction have a higher risk of a certain type of cancer, and point to the necessity of objective measurement of mitochondrial function in epidemiologic studies. For the identified putative causal genes, it is feasible to be added to the genetic screening project for better cancer prevention. True causal effects of mitochondrial dysfunction on cancers might be more complex and need larger genetic datasets and sophisticated experimental studies to further confirm.

## Introduction

Mitochondria are the essential organelles that regulate cellular energy production, metabolism, proliferation and apoptosis. An altered mitochondrial function is a well-known hallmark of cancer, which is commonly characterized by abnormal mitochondrial morphology, deficient mitochondrial copy numbers, aberrant energetic metabolism, accumulation of reactive oxygen species (ROS), imbalanced biogenesis and mitophagy.^1^ A mild mitochondrial dysfunction may enhance the amplification and invasion of cancer cells while a severe level of dysfunction may cause cell death to inhibit tumorigenesis. Thus, understanding the roles of mitochondrial dysfunction is essential for cancer research. Mitochondrial dysfunction is a complex cellular process that exhibits a spectrum of pathological conditions although there is no specific biomarker/s to define mitochondrial dysfunction.^2^^,^^3^ With the exception of the 37 critical bioenergetic genes encoded by the mitochondrion itself, the mitochondrial-related genome encompasses more than 1000 additional nuclear genes, and the genetic predisposition in those genes will potentially cause mitochondrial dysfunction.^4^ Many experimental and epidemiological studies have attempted to infer the causal relationship between mitochondrial dysfunction and cancer by exploring the selective mitochondrial DNA (mtDNA) and mitochondrial-related nuclear DNA mutations that affect mitochondrial function and are associated with the risk of specific cancer types.^5^^,^^6^ However, the results generated from those studies are inconsistent and one of the reasons is the methodologies used in these studies, which do not consider the effect of confounders to differentiate between cause and consequence. Therefore, a comprehensive analysis of all genes related to mitochondrial dysfunction in multiple cancer types by a robust method is required to determine whether mitochondrial dysfunction per se is a cause or consequence of cancer.

Mendelian randomization (MR) is a method that uses genetic variants as instrumental variables (IVs) to explore the potential causal association between lifetime exposure and outcome. In MR, the use of the conceptional random allocation of alleles avoids bias from unobserved confounders such as lifestyle and environmental factors and the problem with reverse causality.^7^ The two-sample MR allows for the assessment of the IVs-exposure association and IVs-outcome association generated from different populations.^8^ Genome-wide association studies (GWAS) exploit the genetic associations with traits based on single nucleotide polymorphisms (SNPs) and integration of the GWAS data with gene expression and methylation GWAS have allowed for the identification of expression or methylation quantitative trait loci (eQTL or mQTL).^9^^,^^10^ A summary-data-based MR (SMR) has extended and developed the conception of MR that can utilize the independent GWAS summary statistics data and QTL data to prioritize potential causal genes from hits identified in GWAS.^9^ By applying this method followed by a heterogeneity independent instruments (HEIDI) test, the potential causal associations were distinguished from the widespread linkage disequilibrium (LD) in the genome.

To our knowledge, there has been no MR study investigating the potential causal relationship between mitochondrial dysfunction and the risk of common types of cancer. Therefore, in this study, we aimed to investigate the causal relationship between mitochondrial dysfunction characterized by genetic predisposition in mitochondrial-related genes and multiple cancer types by the comprehensive two-sample MR analysis.

## Methods

This study was conducted following the reporting guideline of the Strengthening the Reporting of Observational Studies in Epidemiology (STROBE, Supplementary STROBE-MR checklist table).^11^

### Study design

**Fig. 1:**
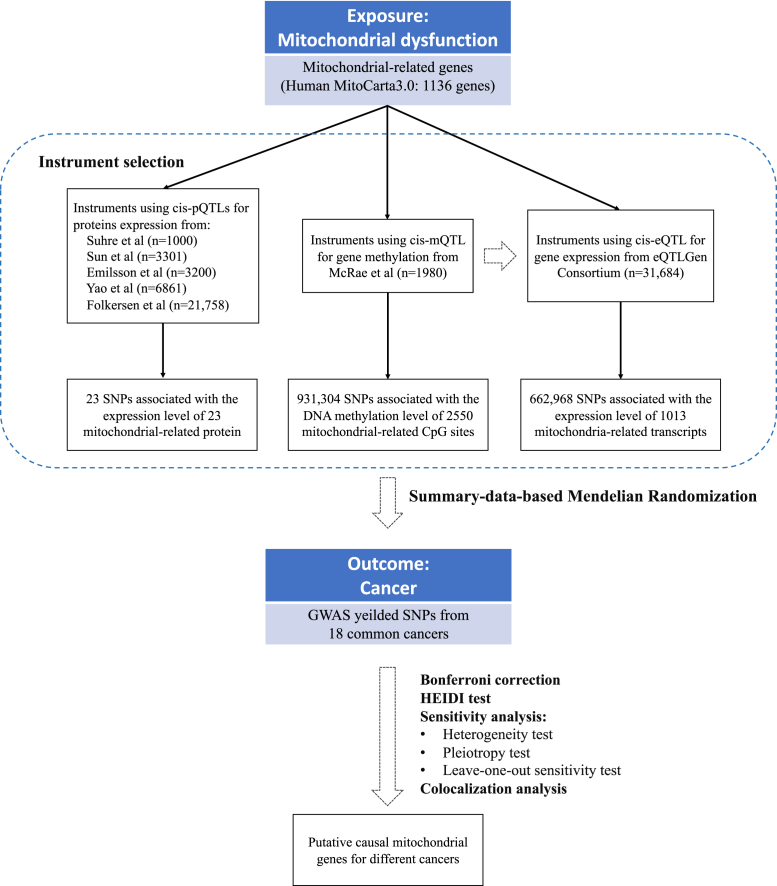
**Flowchart of the analyses performed**.

Fig. 1 summarizes the design of the present study and the workflow of the selection of genetic variants and analytical methods. To determine the mitochondrial dysfunction characterized by the genetic predisposition in the mitochondrial-related genome constituting from both mitochondrion and nuclear, we extracted the inventory of 1136 known mitochondrial-related genes from the human MitoCarta3.0 database.^4^

To generate eQTL instruments for mitochondrial genes, genetic variants located within 1000 kb on either side of the coding sequence (in *cis*) that are robustly associated with gene expression were extracted using eQTLs summary statistics obtained from the eQTLGen Consortium (https://www.eqtlgen.org/cis-eqtls.html). The eQTLGen Consortium contains information on 10,317 trait-associated single nucleotide polymorphisms (SNPs) from 31,684 individuals.^12^ However, the eQTLGen did not include variants associated with the expression level of genes located on the X and Y chromosomes and mtDNA. From cis-eQTL, 662,968 SNPs associated with the expression of 1013 mitochondrial-related transcripts were selected. MR *cis*-mQTL instruments for genetic variants robustly associated with mitochondrial gene methylation were extracted using summary data from a meta-analysis of two cohorts (n = 1980).^10^ In total, 931,304 SNPs were selected corresponding to 2550 mitochondrial-related DNA methylation CpG sites. MR *cis*-pQTL instruments for genetic variants associated with the expression of mitochondrial-related proteins were selected from five proteome datasets,^13^, ^14^, ^15^, ^16^, ^17^ and 23 SNPs that were robustly associated with 23 mitochondrial-related protein expressions were selected. All SNPs included in the initial analysis had at least a suggestive *P*_snp-mitodys_ <5 × 10^−8^.

GWAS summary statistics for cancer outcomes were obtained from publicly available databases. A total of 18 types of cancers were included. The details of all QTL and GWAS datasets for this study are presented in Supplementary Table S1 and Supplementary methods.

### Statistical analysis

The main analyses involved three stages: primary SMR analyses, sensitivity analyses and colocalization analyses.

Mendelian randomization requires meeting three core assumptions (Supplementary methods). As an extension of the MR concept, SMR was developed to estimate the pleiotropic association between genetically determined traits (e.g., gene expression, DNA methylation, or protein abundance as exposure) and complex traits of interest (e.g., disease phenotype as outcome).^9^ To meet MR assumptions in our study, the causal association was calculated as:

$$\beta_{mitodys-cancer}=\beta_{SNP-cancer}/\beta_{SNP-mitodys}.$$

β_mitodys-cancer_ is calculated as the estimated effect size of mitochondrial dysfunction on cancer, where β_SNP-mitodys_ is the estimated effect size of SNP on mitochondrial dysfunction (a genetic variant—exposure trait association) and β_SNP-cancer_ is the estimated effect size of SNP on cancer (the same genetic variant—outcome trait association).

Here, we performed SMR using the Linux version 1.0.3 of SMR software in the command line using default options (https://yanglab.westlake.edu.cn/software/smr/#Overview). Odds ratio (OR) estimates of mitochondrial dysfunction on the risk of cancer were obtained as follows: OR_mitodys-cancer_ = exp (β_mitodys-cancer_), where OR is the odds ratio estimate per 1-ln increment in mitochondrial genome levels and exp is the base of the natural logarithm.

Sensitivity analyses were conducted after completing the primary SMR analyses with 5 additional MR methods, including MR Egger, weighted median, inverse variance weighting (IVW), simple mode and weighted mode by using the TwoSampleMR R package. Each of these methods calculates the estimates of the causal effect based on slightly different assumptions about the instrument validity and therefore provide robust evidence of our findings (Supplementary methods). All analyses in this part were performed using R software (version 4.1.2, www.r-project.org).

HEIDI test is one of the colocalization methods that use external reference to estimate the LD. To refine the results, we performed another Bayesian test for the colocalization of two traits using the coloc R package (https://chr1swallace.github.io/coloc/, version 5.1.0) to estimate the posterior probability of shared variants.^18^ For each leading SNP in the investigated cancer GWAS database, all SNPs within 100 kb up and downstream of the leading SNPs were retrieved for colocalization analysis to analyze the posterior probability of H4 (PP.H4), and PP.H4 > 0.8 is the well-applied cut-off for the evidence of colocalization of the GWAS and QTL association (Supplementary methods).

### Role of the funding source

The funder of the study had no role in study design, data collection, data analysis, data interpretation, or writing of the report.

### Ethics

All summarized statistics utilized in the MR analyses were generated by previous studies, for which ethical approval and individual consent were obtained for all original studies.

## Results

### MR analysis of mitochondrial genome-wide *cis*-eQTLs and cancer outcomes

**Fig. 2:**
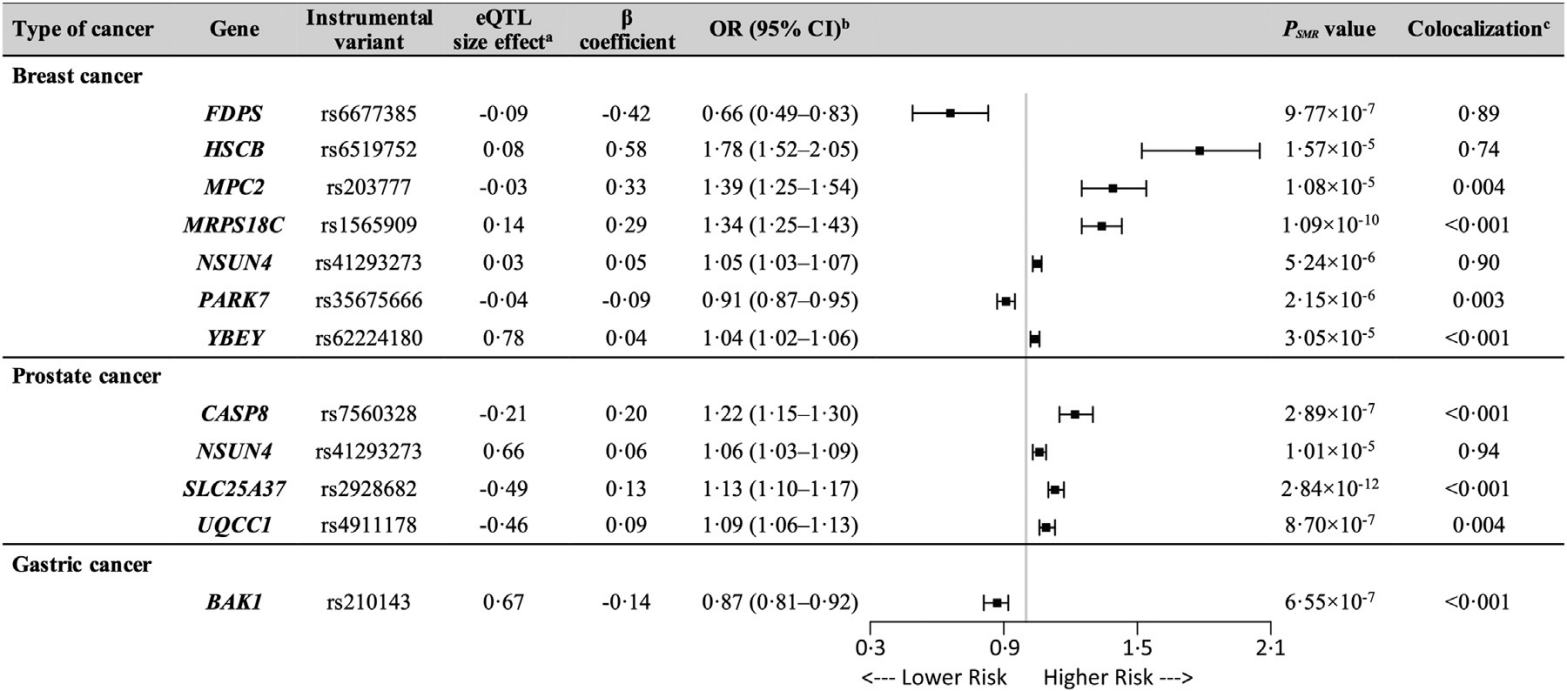
**Mendelian randomization results for the association between the expression of mitochondrial genes and cancer risk.**^a^Represents the effect size (β) of a variant on mRNA expressions. β > 0 means positive association, and β < 0 means negative association. ^b^Odd ratios were calculated by the expectation of causal estimate (β coefficient). ^c^‘Colocalization’ indicates PP.H4 between eQTLs and cancer outcomes. PP.H4 > 0.8 is the well-applied cut-off for the evidence of colocalization.

After SMR testing, the associations of 662,968 SNPs from blood representing mitochondrial-related gene expression and cancer outcomes were obtained (Fig. 1). To control the genome-wide type I error, we performed multiple testing corrections, with the results showing strong evidence of an association (*P*_SMR_<4.936 × 10^−5^ [Bonferroni correction, *P* < 0.05/1013]) followed by the HEIDI test (*P*_HEIDI_>0.01) implemented in SMR software to investigate if the association was due to a shared causal variant and not pleiotropy. We thus identified 7 association signals across 7 unique genetic loci for breast cancer, 4 association signals across 4 unique genetic loci for prostate cancer and one association signal for gastric cancer. We found no significant genetic correlation for the other cancer types. Sensitivity analysis using additional MR methods relying on similar assumptions was conducted and shown to support our findings (Supplementary Table S2, Supplementary Figure S1 and S2). We further performed colocalization analysis to rule out confounding by LD; strong evidence of colocalization between cancer GWAS and eQTL exists if the posterior probability of shared causal variant across gene expression and cancer (PP.H4) is >0.80. The causal estimates are expressed as β coefficients, and the odds ratios (OR) for 1 standard deviation (SD) change in mitochondrial gene expression level was calculated by the expectation of the β coefficient, as presented in Fig. 2 and Supplementary Table S3.

For breast cancer, one SD decrease of *FDPS* expression was associated with 34% lower risk (OR: 0.66, 95% CI: 0.49–0.83, *P*_SMR_ = 9.77 × 10^−7^) while 1 SD increase of *NSUN4* expression was associated with 5% higher risk (OR: 1.05, 95% CI: 1.03–1.07, *P*_SMR_ = 5.24 × 10^−6^). When sub-grouping breast cancer according to the intrinsic molecular subtypes, the causal associations showed a similar trend only with luminal A-like breast cancer. Interestingly, we found a robust causal association (OR per SD, 1.17; 95% CI: 1.12–1.23, *P*_SMR_ = 1.85 × 10^−8^) between *MTX1* expression and luminal A-like breast cancer, specifically. We also found a strong causal association (OR per SD, 1.26; 95% CI: 1.15–1.37, *P*_SMR_ = 2.94 × 10^−5^) between *COX11* expression and luminal B-like/HER2-negative cancer (Supplementary Table S4, Supplementary Fig. S3). For prostate cancer, one SD increase of *NSUN4* expression was associated with 6% higher risk of cancer (OR: 1.06, 95% CI: 1.03–1.09, *P*_SMR_ = 1.01 × 10^−5^).

Most importantly, our results show that the expression level of *NSUN4* increased by rs41293273 is associated with higher risk of both breast cancer and prostate cancer.

### MR analysis of mitochondrial genome-wide *cis*-mQTLs and cancer outcomes

**Fig. 3:**
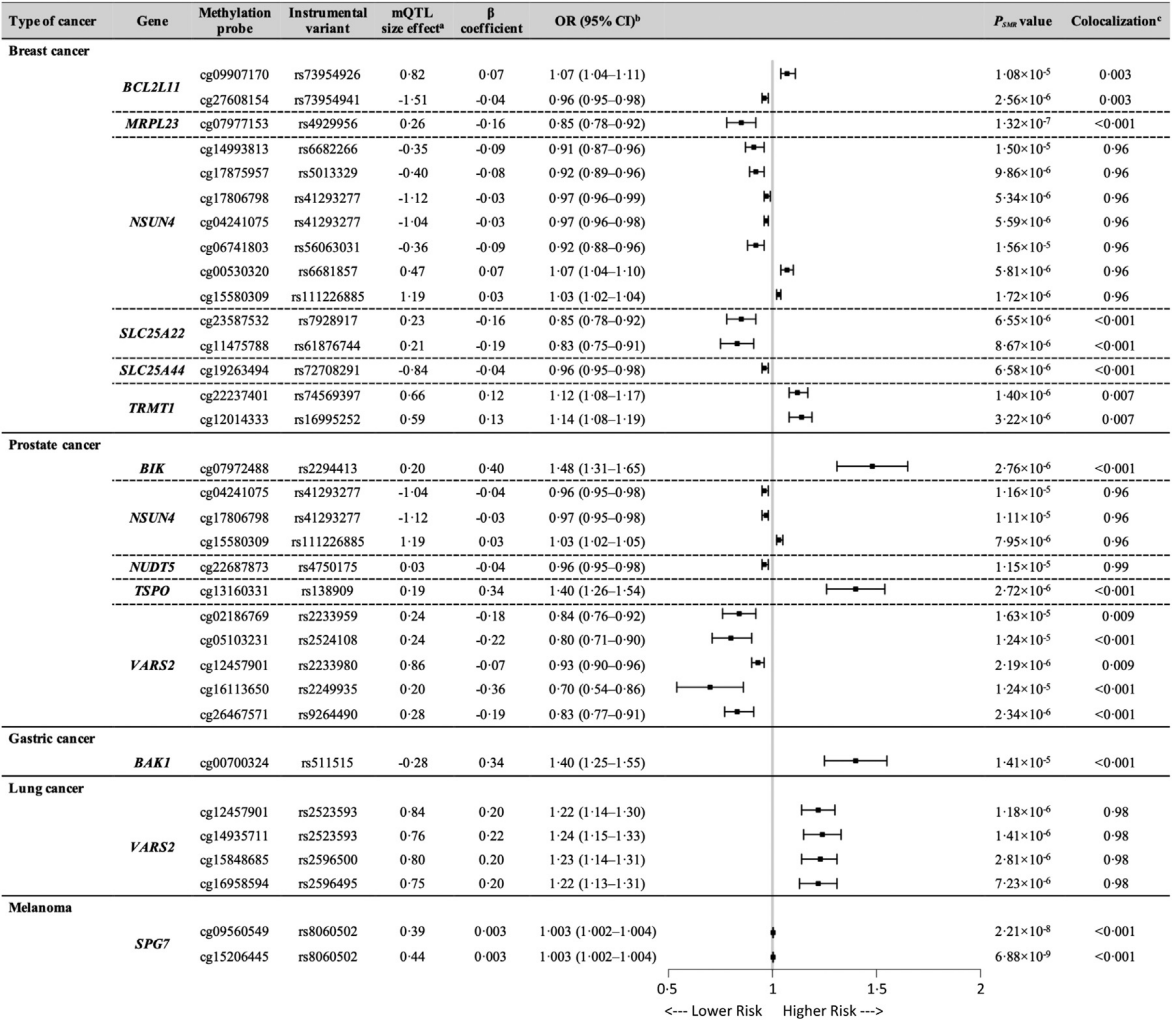
**Mendelian randomization results for the association between mitochondrial gene methylations and cancer risk.**^a^Represents the effect size (β) of a variant on DNA methylation. β > 0 means positive association, and β < 0 means negative association. ^b^Odd ratios were calculated by the expectation of causal estimate (β coefficient). ^c^‘Colocalization’ indicates PP.H4 between mQTLs and cancer outcomes. PP.H4 > 0.8 is the well-applied cut-off for the evidence of colocalization.

For the causal association between the DNA methylation of the mitochondrial-related genome and cancer outcomes, Bonferroni correction (*P*_SMR_ < 1.961 × 10^−5^) and HEIDI test were performed. We identified a total of 15 association signals across 14 unique genetic loci for breast cancer, 11 association signals across 10 unique genetic loci for prostate cancer, one association signal for gastric cancer, 4 association signals across 3 unique genetic loci for lung cancer and 2 association signals across one unique genetic locus for melanoma (Fig. 3 and Supplementary Table S5). The sensitivity analysis supported the same associations (Supplementary Table S6, Supplementary Fig. S4 and S5).

The colocalization analysis showed that different genetic variants regulating *NSUN4* had different effects on methylation levels, hence the outcome. For example, one SD decrease of *NSUN4* methylation by rs6682266 was associated with 9% lower risk of breast cancer (OR: 0.91, 95% CI: 0.87–0.96, *P*_SMR_ = 1.50 × 10^−5^), and conversely, one SD increase of *NSUN4* methylation by rs6681857 was associated with 7% higher risk of breast cancer (OR: 1.07, 95% CI: 1.04–1.10, *P*_SMR_ = 5.81 × 10^−6^). Here, in total, we found 6 unique loci that regulated the methylation level of 7 different CpG sites in *NUSU4*, and were positively associated with the risk of breast cancer (Fig. 3). Analysis on breast cancer molecular subtypes with *NSUN4* methylation showed a similar causal association but only for luminal A-like breast cancer (Supplementary Table S7 and Supplementary Fig. S6). Out of these 6 loci, two were also positively associated with risk of prostate cancer. Furthermore, an increase of 1 SD of *NUDT5* methylation was associated with 4% lower risk of prostate cancer (OR: 0.96, 95% CI: 0.95–0.98, *P*_SMR_ = 1.15 × 10^−5^) (Fig. 3). For lung cancer, one SD increase of *VARS2* methylation was associated with more than 20% higher risk of cancer depending on different methylation CpG sites (Fig. 3). We found no significant causal associations between mitochondrial genome methylation and other cancer risks.

Furthermore, gene methylation is known to influence gene expression. Here, we also performed SMR analysis on the causal association between mitochondrial-related gene methylation and expression by mapping the gene methylation to expression through shared genetic variants. After multiple testing corrections and the HEIDI test, we obtained the gene list for the mitochondrial gene expression regulated by DNA methylation CpG sites (Supplementary Table S8). For the putative causal genes that we identified above, SMR results showed that the methylation of *NSUN4*, which was regulated by rs6682266, rs5013329, rs56063031 and rs6681857, was associated with *NSUN4* expression, and *VARS2* methylation by rs2596495 was also associated with *VARS2* expression (Supplementary Table S8).

### MR analysis of mitochondrial genome-wide *cis*-pQTLs and cancer outcomes

Only 23 proposed mitochondrial-related SNPs were extracted from *cis*-pQTLs and no causal association was found based on our suggested threshold after the SMR analyses. One possible explanation could be that the pQTL datasets are not comprehensively developed, and very few genetic variants that are robustly associated with protein levels were identified.

### Phenome-wide scan of identified genetic variants

To exclude possible pleiotropy of investigated cancers, we performed phenome-wide scan analysis on the identified variants, using both GWASATLAS^19^ and PhenoScanner^20^ databases. The databases enable the investigation of genetic variants across multiple disease traits. The Manhattan plots were used to show phenome-wide scan results of identified genetic variants on different disease traits with any possible effect allele obtained from GWASATLAS (Supplementary Fig. S8–S10). The phenome-wide scan of all identified genetic variants with disease traits from PhenoScanner was also listed according to the following selection criteria: 1) the SNPs shared the same effect allele with our results, 2) the association reached GWAS significance (*P* < 5 × 10^−8^) and 3) the absolute value of size effect (β) >0.01 Supplementary Table S9). Interestingly, variants related to the gene expression and DNA methylation of *NSUN4* that were causally associated with both breast and prostate cancer were not found to be associated with all available secondary traits (Supplementary Fig. S7 and S8). This further suggests that the causal relationship between *NSUN4* and breast and prostate cancer identified in this study is robust. However, rs6677385 (*FDPS* expression associated) was associated with secondary traits such as metabolic-related blood urea nitrogen and Crohn's disease (Supplementary Fig. S9a). The rs4750175 (*NUDT5* methylation associated) was also associated with endocrine-related traits (risk of type 2 diabetes, Supplementary Fig. S9b). Three SNPs associated with *VARS2* methylation were further associated with multiple traits such as metabolic, skeletal, respiratory, psychiatric, immunological and endocrine-related traits (Supplementary Fig. S10). The genetic variants that are associated with the secondary traits may potentially introduce horizontal pleiotropy, further investigations to rule out pleiotropy are needed.

### Bi-directional MR analysis of mitochondrial dysfunction and cancers

GWAS summary statistics were available only for mtDNA copy number variation, which has been suggested as a surrogate biomarker for mitochondrial dysfunction. Currently, a GWAS dataset specifically containing genetic variant association with mtDNA copy number has been published.^21^ We used this dataset to explore whether mitochondrial dysfunction is a consequence of cancer and conducted bidirectional MR analyses on mtDNA copy number and cancers. Results showed that the directions of causal association were cancer type-specific; here mtDNA copy number variation has causal effects on cervical cancer, specific subtype of ovarian cancer (Supplementary Table S10), while triple-negative breast cancer, head and neck cancer were causally associated with mtDNA copy number variation (Supplementary Table S11).

## Discussion

In this study, we demonstrate that mitochondrial dysfunction characterized by genetic predisposition has causal effect on cancers, and identified important putative causal mitochondrial-related genes as follows: 1) *FDPS* for breast cancer; 2) *NUDT5* for prostate cancer; 3) *VARS2* for lung cancer and 4) *NSUN4* for both breast and prostate cancers. Our results show that genetic determinants of mitochondrial dysfunction were associated with the risk of cancer in a cancer type-specific manner, which provides robust evidence for underlying mechanisms linking the genetic loci, gene expression, and methylation with multiple cancers.

The FDPS is a key enzyme that is involved in the mevalonate pathway to catalyze the biosynthesis of cholesterol and sterol, and to isoprenylate cellular metabolites such as Ras, Rac, Rab and Rho for membrane anchorage and cellular signaling.^22^ *FDPS* has been investigated over decades for its physiological function and was found to be associated with leukemia growth,^23^ the progression of prostate cancer,^24^ the poor breast cancer prognosis,^25^ and directly involved in glioblastoma drug resistance^26^ and pancreatic cancer radioresistance.^27^ However, its causal relationship with cancer is unclear. In this study, we show that gene expression of *FDPS* has a causal relationship with breast cancer. Studies have shown that knockdown *FDPS* enhanced apoptosis and ectopic overexpression of *FDPS* promoted cancer colony growth and proliferation by affecting STAT3, AKT and ERK pathways.^24^ Prenylation is important for exerting the activity of oncogenic proteins, thus prenylation inhibitors have been widely applied in clinical trials for cancer treatment.^28^ FDPS was shown to be a key target of nitrogen-containing bisphosphonates and it is already a clinical drug target by Zoledronic acid.^29^ According to the DRUGBANK database (https://go.drugbank.com/), more drugs have been investigated such as Ibandronate, Minodronic acid and Incadronic acid that target FDPS.

*NSUN4* is an rRNA m^5^C methyltransferase that can induce the methylation of the 12S rRNA of the small ribosomal subunit joining in mitochondria and promote rRNA rearrangements to form peptidyl transferase center.^30^^,^^31^ A previous study showed that breast cancer and prostate cancer shared a common risk locus (rs5013329) and indicated that *NSUN4* is the strongest shared functional candidate at 1p34.^32^ However, our results showed that rs5013329 related to *NSUN4* methylation was associated with decreased risk of breast cancer only. Importantly, we identified additional 1 genetic locus related to *NSUN4* expression and 2 loci related to *NSUN4* methylation that were causally associated with both breast and prostate cancer (Supplementary Fig. S7). Furthermore, our phenome-wide scan analysis showed that the causal relationship between *NSUN4* and both breast and prostate cancers was not caused by horizontal pleiotropy. Together, these results emphasize the potentially important role of *NSUN4* in carcinogenesis.

*NUDT5* is differentially expressed in different types of cancer and positively correlated with aggressive cancer disease phenotype, knockdown of which can suppress the proliferation of cancer cells without inducing DNA oxidative lesion.^33^ In our study, we show that the methylation level of *NUDT5* has a strong causal effect on prostate cancer. Those findings highlight that NUDT5 may represent a promising drug target for cancer prevention and treatment. More studies now focus on the identification of NUDT5 inhibitors from approved drugs and small molecules, and a potent TH5427 was tested and shown to block hormone signaling and disrupt the proliferation of breast cancer cells.^34^

Several mutations in *VARS2* have been associated with mitochondrial diseases such as complex I defect, early onset of mitochondrial encephalomyopathies and encephalocardiomyopathies,^35^ and cancer risks including breast cancer, colon and lung cancer.^36^, ^37^, ^38^ Here, we propose *VARS2* as a causal gene only for lung cancer. The mechanisms of *VARS2* in lung cancer carcinogenesis need further evaluation by experimental studies.

The main strength of the present study is that we performed a comprehensive MR analysis between mitochondrial dysfunction, characterized by genetic predisposition in all known mitochondrial-related genes, and their causal relationship with cancers. The inclusion of all genes related to mitochondria eliminates the selection bias in previous studies and might be able to address mitochondrial dysfunction directly. Secondly, we have included a very large sample size and 18 different cancer outcomes from GWAS summarized statistics, which allowed us to gain sufficient power to elucidate causal relationships and make conclusive estimations for several cancer types. Thirdly, we used SMR as the primary analysis and performed a sensitivity analysis using 5 additional MR approaches and colocalization analysis, which shows the robustness of our findings. Finally, we only included samples of European ancestry, thus, we minimized the biases caused by different genetic backgrounds.

This study has several limitations as well. Although we drew on the large available GWAS data sources, no genetic variants were obtained that represent the mitochondrial protein expression and the available eQTL and mQTL datasets did not have information on genetic variants that were associated with gene expression or methylation level in the X chromosome, Y chromosome and mitochondrial genome; the mitochondrial genome-wide associated genetic variants in this study mainly laid on the mitochondrial-related nuclear genome rather than the mitochondrial genome itself because a mitochondrial genome-specific QTL dataset has not yet been developed. Moreover, GWAS dataset that directly reflected on mitochondrial dysfunction is not available, hence we cannot assess the direction of causal relationship by using bi-directional MR based on current software resources. In this study, we showed that causal effects of mtDNA copy number variations and cancers were bi-directional in a cancer-specific manner. However, GWAS summary statistics of mtDNA copy number variation are potentially underpowered to detect the direction of causal association between mitochondrial dysfunction and cancers. Furthermore, univariable MR estimates the total effect of exposure on the outcome. As an extension, multivariable MR simultaneously estimates several potentially related exposures with a shared set of SNPs on the outcome using GWAS summary statistics, allowing for the assessment of the direct causal effect of a single exposure on the outcome. In this study, the exposure was mitochondrial dysfunction characterized by predisposition in the mitochondrial-related gene, which only can be retrieved from the QTL datasets other than the GWAS dataset. Thus, we are unable to perform multivariable MR to estimate the direct causal effects of mitochondrial dysfunction on cancers. Further studies should be conducted on the question of whether mitochondrial dysfunction is causally associated with cancer when GWAS or more advanced methods are available.

This study leverages MR to examine the potential causal relationship between mitochondrial dysfunction characterized by genetic predisposition in mitochondrial-related genes and cancer, and demonstrates the importance of mitochondrial dysfunction in the pathogenesis of multiple cancer types. The identified putative genes can function as potential pharmacological targets for cancer treatment and prevention, further research could explore details of the underlying biological mechanisms.

## Contributors

YL designed the study and performed the statistical analysis; YL, KS, NZ, XW and AAM participated in data interpretation; YL and AAM wrote the first draft, and KS, NZ, XW and JS revised the article. YL and NZ had direct access and responsibility for verifying all data reported in the manuscript. All authors read and approved the submitted version of the manuscript.

## Data sharing statement

Data used in this study are available from the referenced peer-reviewed studies and listed in Supplementary Table S1. Summary statistics for GWAS are publicly available for download. The statistical code needed to reproduce the results in the article is available upon request.

## Declaration of interests

The authors declare that there is no conflict of interest associated with this manuscript.
