# Supplementary material for: Mitochondrial related genome-wide Mendelian randomization identifies putatively causal genes for multiple cancer types
Source: eBioMedicine. 2023 Jan 10;88:104432. doi: 10.1016/j.ebiom.2022.104432 (PMC9841346; doi:10.1016/j.ebiom.2022.104432)
Supplement: Supplementary Material [file mmc1.docx]

**Supplementary material**

**Contents**

**Supplementary methods**

**Supplementary tables**

**STROBE-MR checklist table**

**Supplementary Table 1.** Information of QTL and GWAS datasets.

**Supplementary Table 2.** Sensitivity analysis used TwoSampleMR package on the association between expression of mitochondrial-related genes and cancer outcomes.

**Supplementary Table 3.** SMR and colocalization results of the association between expression of mitochondrial-related genes and cancer outcomes.

**Supplementary Table 4.** SMR and colocalization results of the association between expression of mitochondrial-related genes and breast cancer risk classified by intrinsic–like subtypes.

**Supplementary Table 5.** SMR and colocalization results of the association between DNA methylation of mitochondrial-related genes and cancer outcomes.

**Supplementary Table 6.** Sensitivity analysis used TwoSampleMR package on the association between DNA methylation of mitochondrial-related genes and cancer outcomes.

**Supplementary Table 7.** SMR and colocalization results of the association between DNA methylation of mitochondrial-related genes and luminal A-like breast cancer risk.

**Supplementary Table 8.** SMR results of the association between DNA methylation and expression of mitochondrial-related genes.

**Supplementary Table 9.** Phenome-wide scan of the association between identified SNPs with other diseases traits using PhenoScanner.

**Supplementary Table 10.** MR analysis used TwoSampleMR package on the causal effect of mtDNA copy number variation on cancer outcomes.

**Supplementary Table 11.** MR analysis used TwoSampleMR package on the causal effect of cancers on mtDNA copy number variation.

**Supplementary figures**

**Supplementary Figure 1.** Leave-one-out sensitivity analyses of the SNPs represented the expression of mitochondrial-related genes and breast cancer outcome.

**Supplementary Figure 2.** Leave-one-out sensitivity analyses of the SNPs represented the expression of mitochondrial-related genes and prostate cancer outcome**.**

**Supplementary Figure 3.** Mendelian randomization results for the association between expression of mitochondrial-related genes and breast cancer risk classified by intrinsic–like subtypes.

**Supplementary Figure 4.** Leave-one-out sensitivity analyses of the SNPs represented the DNA methylation of mitochondrial-related genes and breast cancer outcome.

**Supplementary Figure 5.** Leave-one-out sensitivity analyses of the SNPs represented the DNA methylation of mitochondrial-related genes and prostate cancer outcome.

**Supplementary Figure 6.** Mendelian randomization results for the association between DNA methylation of mitochondrial-related genes and luminal A-like breast cancer risk.

**Supplementary Figure 7.** Results of SNPs and SMR associations across mQTL, eQTL and breast and prostate cancer GWAS.

**Supplementary Figure 8.** Manhattan plots show the phenome-wide scan results of genetic variants in *NSUN4.*

**Supplementary Figure 9.** Manhattan plots show the phenome-wide scan results of genetic variants in *FDPS* and *NUDT5*.

**Supplementary Figure 10.** Manhattan plots show the phenome-wide scan results of genetic variants in *VARS2*.

**Supplementary method references.**

**Supplementary methods**

### Study outcomes

GWAS summary statistics for cancer outcomes were obtained from publicly available databases (Supplementary Table 1). A total of 18 types of cancers were included.

The variants associated with overall breast cancer risk were retrieved from the Breast Cancer Association Consortium (BCAC), which included 133,384 breast cancer cases and 113,789 controls of European ancestry from 82 studies. The genetic associations were adjusted for age, country, array-specific first ten ancestry principal components (PCs). In addition, the associations of SNPs with five surrogate molecular subtypes of breast cancer are defined by tumor grade and receptor status including estrogen receptor (ER), progesterone receptor (PR) and human epidermal growth factor receptor (HER2), as luminal A-like, luminal B/HER2-negative-like, luminal B/HER2-positive and triple-negative and these were involved for further subgroup analysis (1).

The SNPs associated with the risk of bladder cancer (cases: 2670, controls: 385,138), corpus uteri cancer (cases: 1515, controls: 210,164), esophagus cancer (cases: 992, controls: 386,907) and non-Hodgkin lymphoma (cases: 1579, controls: 386,126), that were adjusted for age, sex, array-specific 10 ancestry PCs, exome sequencing batch and sequencing specific 20 PCs, were obtained from a published study of 454,787 UK Biobank participants (2).

The SNPs associated with the risk of cervical cancer and melanoma, and were adjusted for age, sex and genotype measurement batch, were downloaded from the IEU OpenGWAS project as the outputs from a GWAS pipeline analyzing variables from the UK Biobank. Cervical cancer comprised 1889 cases and 461,044 controls and melanoma comprised 3598 cases and 459,335 controls (3).

The SNPs associated with the risk of colorectal cancer (cases: 6581, controls: 463,421), gastric cancer (cases: 1029, controls: 475,087), pancreatic cancer (cases: 1196, controls: 475,049) and thyroid cancer (cases: 1054, controls: 490,920) were obtained from a large cross-population study that involved 220 deep-phenotype genome-wide associations from BioBank Japan, UK Biobank and FinnGen. The genetic associations were adjusted for age, sex, top 20 genotype PCs, time from sampling to biobanking (4). However, in this study, we only retrieved the SNPs from samples of mainly European ancestry.

The SNPs associated with the overall endometrial cancer risk, sub-grouped with either endometrioid or non-endometrioid histology were retrieved from a meta-analysis of 17 studies containing up to 12,906 cases and 108, 979 controls identified via UK Biobank, the Epidemiology of Endometrial Cancer Consortium (E2C2) and the Endometrial Cancer Association Consortium (ECAC). The SNP-cancer associations were adjusted for corresponding PCs in each study (5).

The SNPs associated with the risk of kidney cancer, with age, study center and the significant eigenvectors as covariates, were obtained from a sex-specific GWAS study analysis of renal cell carcinoma for men (cases: 3227, controls: 4916) and women (cases: 1992, controls: 3095) of European origin (6).

The SNPs associated with the risk of liver cancer were obtained from a case-control GWAS study of alcohol-related hepatocellular carcinoma, corresponding to 775 cases with alcohol-related liver cancer and 1332 controls with alcohol-related liver disease only. The genetic associations were adjusted for first 10 genotype PCs, age, sex, and liver fibrosis (7).

The SNPs associated with the risk of lung cancer were obtained from a meta-analysis of 4 GWAS studies, resources from the MD Anderson Cancer Center (MDACC), the Institute of Cancer Research (ICR), the National Cancer Institute (NCI) and the International Agency for Research on Cancer (IARC) of 11,348 cases and 15,861 controls of European ancestry with the adjustment for age, sex, histology and smoking status (8).

The SNPs associated with the risk of the oral cavity and pharyngeal cancer (with 2497 cases and 2928 controls of European ancestry) that were adjusted for age, sex and PCs were obtained from a GWAS study of the International Head and Neck Cancer Epidemiology Consortium (INHANCE). Cases and controls were also included from the European cohort study (EPIC) and UK Biobank (9).

The SNPs associated with the overall ovarian cancer risk and different histotypes of epithelial ovarian cancer including mucinous ovarian cancer, low-grade serous and serous ovarian cancer, endometrioid and clear cell carcinoma were obtained from the Ovarian Cancer Association Consortium (OCAC) with a total of 25,509 cases and 40,941 controls. The SNP-cancer associations were adjusted for the eigenvectors of project-specific PCs (10).

The SNPs associated with the risk of prostate cancer were obtained from a meta-analysis of 79,148 prostate cancers and 61,106 controls from the Prostate Cancer Association Group to Investigate Cancer-Associated Alterations in the Genome (PRACTICAL) Consortium, the genetic associations were adjusted for PCs and study specific covariate through logistic regression (11).

GWAS summary statistics for genetic variations associated with mtDNA copy number, with the adjustment for age, sex, 40 ancestry PCs and exome sequencing batch, were obtained from a published study generated from UK biobank-scale whole exome sequencing of 415,422 individuals by the Arking lab (12).

### Summary-data-based Mendelian randomization analyses

Mendelian randomization requires meeting three core assumptions. Assumption 1: the genetic instruments are robustly associated with the exposure. Assumption 2: the genetic instruments are not associated with any major confounders. Assumption 3: the genetic instruments affect the outcome only through exposure (13). SMR applied a two-step least-squares (2SLS) approach to estimate the effect size of an exposure on an outcome by using a genetic variant that is significantly associated with the exposure as an instrument (14). To meet MR assumptions in our study, SNPs with *P*_snp-mitodys_<5 × 10^−8^ were further extracted and included (assumption 1), and all SNPs with linkage disequilibrium (LD) r-squared >0·90 or <0·05 were excluded (assumption 2 and 3) and the causal associations were calculated as:

β_mitodys-cancer_ = β_SNP-cancer_ / β_SNP-mitodys_

β_mitodys-cancer_ is calculated as the estimated effect size of mitochondrial dysfunction on cancer, where β_SNP-mitodys_ is the estimated effect size of SNP on mitochondrial dysfunction (a genetic variant – exposure trait association) and β_SNP-cancer_ is the estimated effect size of SNP on cancer (the same genetic variant – outcome trait association).

To control the rate of genome-wide type I error, we adjusted the SMR *P*-value with Bonferroni correction to account for multiple testing. A *P-value* threshold of 0·05 / the number of probes (1013) = 4·936 × 10^−5^ was defined to assess the statistical significance of the association between mitochondrial-related RNA expression and cancer outcomes. A *P-value* threshold of 0·05 / 2550 = 1·961 × 10^−5^ was set for the statistical significance of the association between mitochondrial-related DNA methylation and cancer outcomes. A *P-value* threshold of 0·05 / 23 ≈ 0·002 was set for the association between mitochondrial-related protein expression and cancer outcomes.

SMR also implemented the heterogeneity in dependent instruments (HEIDI) test to exploit if the observed association was due to vertical pleiotropy rather than the LD with the causal variant. The LD estimation was performed by using genomes of European ancestry obtained from the 1000 Genomes Project Consortium as reference (15). Association with the HEIDI test of *P* <0·01 was implied probably due to linkage rather than pleiotropy (the same variant controls both outcome and exposure independently) and should thus be discarded from the analysis (16).

### Sensitivity analyses

Sensitivity analyses were conducted after completing the primary SMR analyses with 5 additional MR methods, including MR Egger, weighted median, inverse variance weighting (IVW), simple mode and weighted mode by using the TwoSampleMR R package. We tested for heterogeneity across the individual causal effects using Cochran Q statistic implemented in both MR Egger and IVW method, where the *P-value* of Cochran's Q test <0·05 indicates the existence of heterogeneity (17). We further examined the presence of horizontal pleiotropy using MR Egger regression and MR-PRESSO (Pleiotropy Residual Sum and Outlier). MR Egger regression with an intercept assumed that the pleiotropic effects of all identified genetic variants are independent of their instrument strength (InSIDE assumption), which means the effect of horizontal pleiotropy and genetic variants – exposure associations are uncorrelated (18). The intercept of MR Egger can be used to indicate whether directional horizontal pleiotropy is driving the results of MR analysis; there are no directional pleiotropies if an intercept is close to zero, where the *P-value* is >0·05 (19). MR-PRESSO can detect and adjust for any outliers reflecting horizontal pleiotropic biases, where the *P-value* for the Global test >0·05 indicates no horizontal pleiotropic outliers (20). The leave-one-out sensitivity tests were applied to remove one genetic variant at a time to confirm the results were not driven by any single variant, the estimate of the rest variants >0 indicates the robustness of the results (21). The Wald ratio estimate is the simplest way to obtain the causal estimate for a genetic variant which represented the exposure on the outcome when there was only a single genetic variant that was included in the analysis (22). F-statistic was used to calculate the strength of the variants and a SNP with a value >10 was considered as a strong MR instrument. We also used the PheWAS dataset to examine the identified genetic variants and their associations with other disease traits in GWASATLAS (<https://atlas.ctglab.nl/PheWAS>) and Phenoscanner (<http://www.phenoscanner.medschl.cam.ac.uk/>) database to detect possible concurrent risk factors (pleiotropy) of investigated cancers. All analyses in this part were performed using R software (version 4.1.2, [www.r-project.org](http://www.r-project.org)).

### Colocalization Analysis

Colocalization is a method to assess the presence of a shared causal variant in the region for two traits. The basic hypothesis for colocalization in the same genomic location is:

H0: neither trait has a causal genetic variant

H1: only trait 1 has a causal genetic variant

H2: only trait 2 has a causal genetic variant

H3: both traits have a causal genetic variant, but not the same variant

H4: both traits share the same causal variant

HEIDI test is one of the colocalization methods that use external reference to estimate the LD. To refine the results, we performed another Bayesian test for colocalization of two traits using the coloc R package (<https://chr1swallace.github.io/coloc/>, version 5.1.0) to estimate the posterior probability of shared variants (23). For each leading SNP in the investigated cancer GWAS database, all SNPs within 100 kb up and downstream of the leading SNPs were retrieved for colocalization analysis to analyze the posterior probability of H4 (PP.H4), with its default priors that the probability of a shared causal genetic variant for trait 1 (P1) and trait 2 (P2) is = 10^−4^ and the probability of a shared causal genetic variant across both traits is P12 = 5 × 10^−5^. Although the threshold for PP.H4 can be modified for multiple hypothesis testing, PP.H4 >0·8 is the well-applied cut-off for the evidence of colocalization of the GWAS and QTL association.

### Statistical power

To obtain sufficient power ( >80%) for Mendelian randomization, the sample size was calculated using the mRnd power calculator (<http://cnsgenomics.com/shiny/mRnd/>). All of our samples included for MR analysis in this study reached enough statistical power.

**STROBE-MR checklist table**

| **Item No.** | **Section** | **Checklist item** | **Relevant text from manuscript** |
| --- | --- | --- | --- |
| 1 | **TITLE and ABSTRACT** | Indicate Mendelian randomization (MR) as the study’s design in the title and/or the abstract if that is a main purpose of the study | Detailed in the Title and Abstract sections |
|  | **INTRODUCTION** |  |  |
| 2 | **Background** | Explain the scientific background and rationale for the reported study. What is the exposure? Is a potential causal relationship between exposure and outcome plausible? Justify why MR is a helpful method to address the study question | Detailed in the Introduction section |
| 3 | **Objectives** | State specific objectives clearly, including pre-specified causal hypotheses (if any). State that MR is a method that, under specific assumptions, intends to estimate causal effects | Detailed in the Introduction section |
|  | **METHODS** |  |  |
| 4 | **Study design and data sources** | Present key elements of the study design early in the article. Consider including a table listing sources of data for all phases of the study. For each data source contributing to the analysis, describe the following: |  |
|  | a) | Setting: Describe the study design and the underlying population, if possible. Describe the setting, locations, and relevant dates, including periods of recruitment, exposure, follow-up, and data collection, when available. | Detailed in the Methods: Study Design, Supplementary methods: Study outcomes and Supplementary Table 1 |
|  | b) | Participants: Give the eligibility criteria, and the sources and methods of selection of participants. Report the sample size, and whether any power or sample size calculations were carried out prior to the main analysis |  |
|  | c) | Describe measurement, quality control and selection of genetic variants | Detailed in the Methods: Study Design, Supplementary methods: Study outcomes |
|  | d) | For each exposure, outcome, and other relevant variables, describe methods of assessment and diagnostic criteria for diseases |  |
|  | e) | Provide details of ethics committee approval and participant informed consent, if relevant | Detailed in the Methods: Ethics and Supplementary Table 1 |
| 5 | **Assumptions** | Explicitly state the three core IV assumptions for the main analysis (relevance, independence and exclusion restriction) as well assumptions for any additional or sensitivity analysis | Detailed in the Methods: Statistical analysis and Supplementary methods: Summary-data-based Mendelian randomization analyses |
| 6 | **Statistical methods: main analysis** | Describe statistical methods and statistics used | Detailed in the Methods and Supplementary methods sections |
|  | a) | Describe how quantitative variables were handled in the analyses (i.e., scale, units, model) |  |
|  | b) | Describe how genetic variants were handled in the analyses and, if applicable, how their weights were selected |  |
|  | c) | Describe the MR estimator (e.g. two-stage least squares, Wald ratio) and related statistics. Detail the included covariates and, in case of two-sample MR, whether the same covariate set was used for adjustment in the two samples |  |
|  | d) | Explain how missing data were addressed |  |
|  | e) | If applicable, indicate how multiple testing was addressed |  |
| 7 | **Assessment of assumptions** | Describe any methods or prior knowledge used to assess the assumptions or justify their validity | Detailed in the Methods, Supplementary methods and Discusion sections |
| 8 | **Sensitivity analyses and additional analyses** | Describe any sensitivity analyses or additional analyses performed (e.g. comparison of effect estimates from different approaches, independent replication, bias analytic techniques, validation of instruments, simulations) | Detailed in the Methods: Statistical analysis and Supplementary methods: Sensitivity analyses and Colocolization analyses |
| 9 | **Software and pre-registration** |  |  |
|  | a) | Name statistical software and package(s), including version and settings used | Detailed in the Methods and Supplementary methods sections |
|  | b) | State whether the study protocol and details were pre-registered (as well as when and where) | Detailed in the Methods: Ethics and Supplementary Table 1 |
|  | **RESULTS** |  |  |
| 10 | **Descriptive data** |  |  |
|  | a) | Report the numbers of individuals at each stage of included studies and reasons for exclusion. Consider use of a flow diagram | Detailed in the Methods, Supplementary methods and Supplementary Table 1 |
|  | b) | Report summary statistics for phenotypic exposure(s), outcome(s), and other relevant variables (e.g. means, SDs, proportions) |  |
|  | c) | If the data sources include meta-analyses of previous studies, provide the assessments of heterogeneity across these studies |  |
|  | d) | For two-sample MR:  i.  Provide justification of the similarity of the genetic variant-exposure associations between the exposure and outcome samples  ii.  Provide information on the number of individuals who overlap between the exposure and outcome studies |  |
| 11 | **Main results** |  |  |
|  | a) | Report the associations between genetic variant and exposure, and between genetic variant and outcome, preferably on an interpretable scale | Detailed in the Results, Supplementary tables and Supplementary figures sections. |
|  | b) | Report MR estimates of the relationship between exposure and outcome, and the measures of uncertainty from the MR analysis, on an interpretable scale, such as odds ratio or relative risk per SD difference |  |
|  | c) | If relevant, consider translating estimates of relative risk into absolute risk for a meaningful time period |  |
|  | d) | Consider plots to visualize results (e.g. forest plot, scatterplot of associations between genetic variants and outcome versus between genetic variants and exposure) |  |
| 12 | **Assessment of assumptions** |  |  |
|  | a) | Report the assessment of the validity of the assumptions | Detailed in the Results and Supplementary tables sections. |
|  | b) | Report any additional statistics (e.g., assessments of heterogeneity across genetic variants, such as *I^2^*, Q statistic or E-value) |  |
| 13 | **Sensitivity analyses and additional analyses** |  |  |
|  | a) | Report any sensitivity analyses to assess the robustness of the main results to violations of the assumptions | Detailed in the Methods, Results, Supplementary methods and Supplementary tables |
|  | b) | Report results from other sensitivity analyses or additional analyses |  |
|  | c) | Report any assessment of direction of causal relationship (e.g., bidirectional MR) | Detailed in the Discussion section |
|  | d) | When relevant, report and compare with estimates from non-MR analyses |  |
|  | e) | Consider additional plots to visualize results (e.g., leave-one-out analyses) | Detailed in the Supplementary figures |
|  | **DISCUSSION** |  |  |
| 14 | **Key results** | Summarize key results with reference to study objectives | Detailed in the Discussion section |
| 15 | **Limitations** | Discuss limitations of the study, taking into account the validity of the IV assumptions, other sources of potential bias, and imprecision. Discuss both direction and magnitude of any potential bias and any efforts to address them |  |
| 16 | **Interpretation** |  |  |
|  | a) | Meaning: Give a cautious overall interpretation of results in the context of their limitations and in comparison with other studies |  |
|  | b) | Mechanism: Discuss underlying biological mechanisms that could drive a potential causal relationship between the investigated exposure and the outcome, and whether the gene-environment equivalence assumption is reasonable. Use causal language carefully, clarifying that IV estimates may provide causal effects only under certain assumptions |  |
|  | c) | Clinical relevance: Discuss whether the results have clinical or public policy relevance, and to what extent they inform effect sizes of possible interventions |  |
| 17 | **Generalizability** | Discuss the generalizability of the study results (a) to other populations, (b) across other exposure periods/timings, and (c) across other levels of exposure |  |
|  | **OTHER INFORMATION** |  |  |
| 18 | **Funding** | Describe sources of funding and the role of funders in the present study and, if applicable, sources of funding for the databases and original study or studies on which the present study is based | Detailed in the Abstract section |
| 19 | **Data and data sharing** | Provide the data used to perform all analyses or report where and how the data can be accessed, and reference these sources in the article. Provide the statistical code needed to reproduce the results in the article, or report whether the code is publicly accessible and if so, where | Detailed in the Data sharing section and Supplementary Table 1 |
| 20 | **Conflicts of Interest** | All authors should declare all potential conflicts of interest | Detailed in the Declaration of interests section |

**Supplementary Table 1.** Information of QTL and GWAS datasets.

| **Type of dataset** | **Data subtype** | **Resource** | **Sample size** | **Population** | **Reference** | **Download Site** |
| --- | --- | --- | --- | --- | --- | --- |
| **QTL** | **cis-eQTL** | eQTLGen Consortium | 31,684 | European | [Võsa U, et al. Nat Genet. 2021. PMID: 34475573. Large-scale cis- and trans-eQTL analyses identify thousands of genetic loci and polygenic scores that regulate blood gene expression.](https://www.nature.com/articles/s41588-021-00913-z) | <https://www.eqtlgen.org/cis-eqtls.html> |
|  | **cis-mQTL** | McRae et al. mQTL summary data | 1980 | European | [Wu Y, et al. Nat Commun. 2018. PMID: 29500431. Integrative analysis of omics summary data reveals putative mechanisms underlying complex traits.](https://www.nature.com/articles/s41467-018-03371-0) | <https://yanglab.westlake.edu.cn/data/SMR/LBC_BSGS_meta_lite.tar.gz> |
|  | **cis-pQTL** | Published paper | 1000 | European | [Suhre K, et al. Nat Commun. 2017. PMID: 28240269. Connecting genetic risk to disease end points through the human blood plasma proteome.](https://www.nature.com/articles/ncomms14357) | <https://www.nature.com/articles/ncomms14357#MOESM1569> |
|  |  |  | 3200 | European | [Emilsson V, et al. Science. 2018. PMID: 30072576. Co-regulatory networks of human serum proteins link genetics to disease.](https://www.science.org/doi/10.1126/science.aaq1327?url_ver=Z39.88-2003&rfr_id=ori:rid:crossref.org&rfr_dat=cr_pub%20%200pubmed) | https://www.science.org/doi/10.1126/science.aaq1327?url_ver=Z39.882003&rfr_id=ori:rid:crossref.org&rfr_dat=cr_pub%20%200pubmed |
|  |  |  | 3301 | European | [Sun BB, et al. Nature. 2018. PMID: 29875488. Genomic atlas of the human plasma proteome.](https://www.nature.com/articles/s41586-018-0175-2) | <http://www.phpc.cam.ac.uk/ceu/proteins/> |
|  |  |  | 6861 | European | [Yao C, et al. Nat Commun. 2018. PMID: 30111768. Genome-wide mapping of plasma protein QTLs identifies putatively causal genes and pathways for cardiovascular disease.](https://www.nature.com/articles/s41467-018-05512-x) | <https://www.nature.com/articles/s41467-018-05512-x#additional-information> |
|  |  |  | 21,758 | European | [Folkersen L, et al. Nat Metab. 2020. PMID: 33067605. Genomic and drug target evaluation of 90 cardiovascular proteins in 30,931 individuals.](https://www.nature.com/articles/s42255-020-00287-2) | <https://www.nature.com/articles/s42255-020-00287-2#Sec35> |
| **GWAS summary** | **Breast cancer** | Breast Cancer Association Consortium (BCAC) | case: 133,384, control: 113,789 | European | [Zhang H, et al. Nat Genet. 2020. PMID: 32424353. Genome-wide association study identifies 32 novel breast cancer susceptibility loci from overall and subtype-specific analyses.](https://www.nature.com/articles/s41588-020-0609-2) | <https://bcac.ccge.medschl.cam.ac.uk/bcacdata/oncoarray/oncoarray-and-combined-summary-result/gwas-summary-associations-breast-cancer-risk-2020/> |
|  | **Bladder cancer** | GWAS Catalog | case: 2670, control: 385,138 | European | [Backman JD, et al. Nature. 2021. PMID: 34662886. Exome sequencing and analysis of 454,787 UK Biobank participants.](https://www.nature.com/articles/s41586-021-04103-z) | <https://www.ebi.ac.uk/gwas/studies/GCST90079612> |
|  | **Cervical cancer** | IEU OpenGWAS project | case: 1889, control: 461,044 | European | [Lyon MS, et al. Genome Biol. 2021. PMID: 33441155. The variant call format provides efficient and robust storage of GWAS summary statistics.](https://genomebiology.biomedcentral.com/articles/10.1186/s13059-020-02248-0) | <https://gwas.mrcieu.ac.uk/datasets/ukb-b-8777/> |
|  | **Colorectal cancer** | GWAS Catalog | case: 6581, control: 463,421 | European | [Sakaue S, et al. Nat Genet. 2021. PMID: 34594039. A cross-population atlas of genetic associations for 220 human phenotypes.](https://www.nature.com/articles/s41588-021-00931-x) | <https://www.ebi.ac.uk/gwas/studies/GCST90018808> |
|  | **Corpus uteri cancer** | GWAS Catalog | case: 1515, control: 210,164 | European | [Backman JD, et al. Nature. 2021. PMID: 34662886. Exome sequencing and analysis of 454,787 UK Biobank participants.](https://www.nature.com/articles/s41586-021-04103-z) | <https://www.ebi.ac.uk/gwas/studies/GCST90079608> |
|  | **Endometrial cancer** | GWAS Catalog | case: 12,906, control: 108,979 | European | [O'Mara TA, et al. Nat Commun. 2018. PMID: 30093612. Identification of nine new susceptibility loci for endometrial cancer.](https://www.nature.com/articles/s41467-018-05427-7) | [https://www.ebi.ac.uk/gwas/studies/GCST006464, https://www.ebi.ac.uk/gwas/studies/GCST006466, https://www.ebi.ac.uk/gwas/studies/GCST006465](https://www.ebi.ac.uk/gwas/studies/GCST006464,%20https://www.ebi.ac.uk/gwas/studies/GCST006466,%20https://www.ebi.ac.uk/gwas/studies/GCST006465) |
|  | **Esophagus cancer** | GWAS Catalog | case: 992, control: 386,907 | European | [Backman JD, et al. Nature. 2021. PMID: 34662886. Exome sequencing and analysis of 454,787 UK Biobank participants.](https://www.nature.com/articles/s41586-021-04103-z) | <https://www.ebi.ac.uk/gwas/studies/GCST90079575> |
|  | **Gastric cancer** | GWAS Catalog | case: 1029, control: 475,087 | European | [Sakaue S, et al. Nat Genet. 2021. PMID: 34594039. A cross-population atlas of genetic associations for 220 human phenotypes.](https://www.nature.com/articles/s41588-021-00931-x) | <https://www.ebi.ac.uk/gwas/studies/GCST90018849> |
|  | **Kidney cancer** | GWAS Catalog | case: 5219, control: 8011 | European | [Laskar RS, et al. Eur J Hum Genet. 2019. PMID: 31231134. Sex specific associations in genome wide association analysis of renal cell carcinoma.](https://www.nature.com/articles/s41431-019-0455-9) | [https://www.ebi.ac.uk/gwas/studies/GCST008225, https://www.ebi.ac.uk/gwas/studies/GCST008226](https://www.ebi.ac.uk/gwas/studies/GCST008225,%20https://www.ebi.ac.uk/gwas/studies/GCST008226) |
|  | **Liver cancer** | GWAS Catalog | case: 775, control: 1332 | European | [Trépo E, et al. Lancet Oncol. 2022. PMID: 34902334. Common genetic variation in alcohol-related hepatocellular carcinoma: a case-control genome-wide association study.](https://www.thelancet.com/journals/lanonc/article/PIIS1470-2045(21)00603-3/fulltext) | <https://www.ebi.ac.uk/gwas/studies/GCST90092003> |
|  | **Lung cancer** | IEU OpenGWAS project | case: 11,348, control: 15,861 | European | [Wang Y, et al. Nat Genet. 2014. PMID: 24880342. Rare variants of large effect in BRCA2 and CHEK2 affect risk of lung cancer.](https://www.nature.com/articles/ng.3002) | <https://gwas.mrcieu.ac.uk/datasets/ieu-a-966/> |
|  | **Melanoma** | IEU OpenGWAS project | case: 3598, control: 459,335 | European | [Lyon MS, et al. Genome Biol. 2021. PMID: 33441155. The variant call format provides efficient and robust storage of GWAS summary statistics.](https://genomebiology.biomedcentral.com/articles/10.1186/s13059-020-02248-0) | <https://gwas.mrcieu.ac.uk/datasets/ukb-b-12915/> |
|  | **Non-Hodgkin lymphoma** | GWAS Catalog | case: 1579, control: 386,126 | European | [Backman JD, et al. Nature. 2021. PMID: 34662886. Exome sequencing and analysis of 454,787 UK Biobank participants.](https://www.nature.com/articles/s41586-021-04103-z) | <https://www.ebi.ac.uk/gwas/studies/GCST90079638> |
|  | **Oral cavity pharyngeal** | GWAS Catalog | case: 6034, control: 6585 | European | [Lesseur C, et al. Nat Genet. 2016. PMID: 27749845. Genome-wide association analyses identify new susceptibility loci for oral cavity and pharyngeal cancer.](https://www.nature.com/articles/ng.3685) | <https://www.ebi.ac.uk/gwas/studies/GCST012235>, <https://www.ebi.ac.uk/gwas/studies/GCST012237>, <https://www.ebi.ac.uk/gwas/studies/GCST012241> |
|  | **Ovarian cancer** | GWAS Catalog | case: 22,406, control: 40,941 | European | [Phelan CM, et al. Nat Genet. 2017. PMID: 28346442. Identification of 12 new susceptibility loci for different histotypes of epithelial ovarian cancer.](https://www.nature.com/articles/ng.3826) | <https://www.ebi.ac.uk/gwas/studies/GCST004415> |
|  | **Pancreatic cancer** | GWAS Catalog | case: 1196, control: 475,049 | European | [Sakaue S, et al. Nat Genet. 2021. PMID: 34594039. A cross-population atlas of genetic associations for 220 human phenotypes.](https://www.nature.com/articles/s41588-021-00931-x) | <https://www.ebi.ac.uk/gwas/studies/GCST90018893> |
|  | **Prostate cancer** | GWAS Catalog | case: 79,148, control: 61,106 | European | [Schumacher FR, et al. Nat Genet. 2018. PMID: 29892016. Association analyses of more than 140,000 men identify 63 new prostate cancer susceptibility loci.](https://www.nature.com/articles/s41588-018-0142-8) | <https://www.ebi.ac.uk/gwas/studies/GCST006085> |
|  | **Thyroid cancer** | GWAS Catalog | case: 1054, control: 490,920 | European | [Sakaue S, et al. Nat Genet. 2021. PMID: 34594039. A cross-population atlas of genetic associations for 220 human phenotypes.](https://www.nature.com/articles/s41588-021-00931-x) | <https://www.ebi.ac.uk/gwas/studies/GCST90018929> |
|  | **MtDNA copy number** | The Arking Lab | 415,422 | European | [Vamsee P, et al. HGG Adv. 2022. PMID: 36311265. Whole-exome sequencing in 415,422 individuals identifies rare variants associated with mitochondrial DNA copy number.](https://www.sciencedirect.com/science/article/pii/S2666247722000641?via%3Dihub) | https://www.arkinglab.org/resources/ |

**Supplementary Table 2.** Sensitivity analysis used TwoSampleMR package on the association between expression of mitochondrial-related genes and cancer outcomes.

| **Type of cancer** | **MR method** | **No. of SNP** | **OR (95% CI)** | ***P*-value** | **Heterogeneity test** | | **Pleiotropy test** | |
| --- | --- | --- | --- | --- | --- | --- | --- | --- |
|  |  |  |  |  | **Cochran's Q** | ***P*** | **intercept** | ***P*** |
| **Breast cancer** | MR Egger | 7 | 2·72 (1·73-3·71) | 0·104270489456752 | 8·36E-11 | 1 | 8·69E-09 | 1·00 |
|  | Weighted median | 7 | 2·72 (2·47-2·95) | 2·57269830603022E-17 | NA | NA | NA | NA |
|  | Inverse variance weighted | 7 | 2·72 (2·59-2·86) | 1·82493015640925E-44 | 8·38E-11 | 1 | NA | NA |
|  | Simple mode | 7 | 2·72 (2·40-3·04) | 0·000875950662219123 | NA | NA | NA | NA |
|  | Weighted mode | 7 | 2·72 (2·41-3·03) | 0·000767926753069368 | NA | NA | NA | NA |
|  | MR-PRESSO | NA | NA | 1 | NA | NA | NA | NA |
|  |  |  |  |  |  |  |  |  |
| **Prostate cancer** | MR Egger | 4 | 2·72 (1·79-3·64) | 0·16781644931633 | 9·86E-32 | 1 | -2·26E-17 | 1·00 |
|  | Weighted median | 4 | 2·72 (2·43-3·01) | 2·27010381403222E-11 | NA | NA | NA | NA |
|  | Inverse variance weighted | 4 | 2·72 (NA) | NA | 0·00 | 1 | NA | NA |
|  | Simple mode | 4 | 2·72 (2·35-3·09) | 0·0131976089170576 | NA | NA | NA | NA |
|  | Weighted mode | 4 | 2·72 (2·38-3·06) | 0·0102326976144679 | NA | NA | NA | NA |
|  | MR-PRESSO | NA | NA | 1 | NA | NA | NA | NA |
|  |  |  |  |  |  |  |  |  |
| **Gastric cancer** | Wald ratio | 1 | 2·72 (2·33-3·11) | 5·89418294051511E-07 | NA | NA | NA | NA |

NA: not applicable.

Cochran Q statistic implemented in MR Egger and IVW method, *P*>0·05 indicates no heterogeneity exists.

The intercept of MR Egger can be used to indicate whether directional horizontal pleiotropy is driving the results of MR analysis, there are no directional pleiotropies if *P*>0·05.

MR-PRESSO can detect and adjust for any outliers reflecting horizontal pleiotropic biases, where *p* value for Global test > 0·05 indicates no horizontal pleiotropic outliers.

**Supplementary Table 3.** SMR and colocalization results of the association between expression of mitochondrial-related genes and cancer outcomes.

| **Type of Cancer** | **Gene** | **Gene Chr.** | **Probe** | **Probe base pair** | **topSNP** | **SNP Chr.** | **SNP base pair** | **Effect allele** | **Other allele** | **Effect allele frequence** | **GWAS association** | | | **eQTL association** | | | **SMR association** | | | **HEIDI Test** | | **PP.H4** | **PP.H4/PP.H3** | **F-statistic** |
| --- | --- | --- | --- | --- | --- | --- | --- | --- | --- | --- | --- | --- | --- | --- | --- | --- | --- | --- | --- | --- | --- | --- | --- | --- |
|  |  |  |  |  |  |  |  |  |  |  | **β** | **SE** | ***P*** | **β** | **SE** | ***P*** | **β** | **SE** | ***P*** | ***P*** | **No. of SNPs** |  |  |  |
| **Breast cancer** | *PARK7* | 1 | ENSG00000116288 | 8029958 | rs35675666 | 1 | 8021973 | T | G | 0·162577 | -0·0383703 | 0·00803449 | 1·7909E-06 | 0·409378 | 0·0107042 | 0 | -0·0937283 | 0·0197785 | 2·1489E-06 | 0·4684101 | 20 | 0·00329552 | 0·003495486 | 1462·65052 |
|  | *NSUN4* | 1 | ENSG00000117481 | 46818336 | rs41293273 | 1 | 46806393 | C | T | 0·278119 | 0·029888 | 0·00655185 | 5·0724E-06 | 0·663303 | 0·00798164 | 0 | 0·0450594 | 0·00989248 | 5·2407E-06 | 0·01846121 | 20 | 0·8956758 | 8·725554 | 6906·20796 |
|  | *FDPS* | 1 | ENSG00000160752 | 155284498 | rs6677385 | 1 | 155304581 | A | C | 0·268916 | 0·0391088 | 0·00688306 | 1·3321E-08 | -0·0930443 | 0·0096413 | 4·8869E-22 | -0·420324 | 0·0858455 | 9·7662E-07 | 0·02626774 | 20 | 0·8872654 | 7·870584 | 93·1340207 |
|  | *MPC2* | 1 | ENSG00000143158 | 167896122 | rs203777 | 1 | 167876616 | T | C | 0·344581 | -0·0328859 | 0·00678946 | 1·2747E-06 | -0·0991542 | 0·00941492 | 6·1775E-26 | 0·331664 | 0·0753685 | 1·0797E-05 | 0·1475124 | 20 | 0·004180694 | 0·004817406 | 110·914662 |
|  | *MRPS18C* | 4 | ENSG00000163319 | 84383986 | rs1565909 | 4 | 84400330 | C | T | 0·51227 | 0·0414694 | 0·00598823 | 4·3549E-12 | 0·141556 | 0·00794821 | 5·9323E-71 | 0·292954 | 0·0453884 | 1·0866E-10 | 0·3399873 | 20 | 2·227986E-08 | 2·227986E-08 | 317·18884 |
|  | *YBEY* | 21 | ENSG00000182362 | 47711958 | rs62224180 | 21 | 47726332 | C | G | 0·217791 | 0·0310521 | 0·00743739 | 2·978E-05 | 0·775379 | 0·00919112 | 0 | 0·0400477 | 0·00960368 | 3·0455E-05 | 0·03135123 | 20 | 8·230211E-05 | 8·273579E-05 | 7116·90546 |
|  | *HSCB* | 22 | ENSG00000100209 | 29145761 | rs6519752 | 22 | 28904181 | G | A | 0·151329 | 0·0454959 | 0·00817993 | 2·6687E-08 | 0·0785753 | 0·0114645 | 7·1914E-12 | 0·57901 | 0·134068 | 1·5691E-05 | 0·06252503 | 20 | 0·7353553 | 2·778717 | 46·9744667 |
|  |  |  |  |  |  |  |  |  |  |  |  |  |  |  |  |  |  |  |  |  |  |  |  |  |
| **Prostate cancer** | *CASP8* | 2 | ENSG00000064012 | 202125300 | rs7560328 | 2 | 202164837 | A | C | 0·387526 | -0·0429 | 0·0082 | 1·802E-07 | -0·21219 | 0·00808379 | 7·371E-152 | 0·202177 | 0·0394047 | 2·8853E-07 | 0·2166339 | 20 | 0·000331968 | 0·0003339396 | 689·000885 |
|  | *UQCC1* | 20 | ENSG00000101019 | 33945156 | rs4911178 | 20 | 33952620 | G | A | 0·371166 | -0·0405 | 0·0082 | 7·924E-07 | -0·456025 | 0·0083381 | 0 | 0·0888109 | 0·0180546 | 8·6984E-07 | 0·2524193 | 20 | 0·004498653 | 0·004573994 | 2991·18384 |
|  | *NSUN4* | 1 | ENSG00000117481 | 46818336 | rs41293273 | 1 | 46806393 | C | T | 0·278119 | 0·0389 | 0·0088 | 9·643E-06 | 0·663303 | 0·00798164 | 0 | 0·0586459 | 0·0132857 | 1·0138E-05 | 0·1409375 | 20 | 0·9433629 | 17·42376 | 6906·20796 |
|  | *SLC25A37* | 8 | ENSG00000147454 | 23409647 | rs2928682 | 8 | 23435117 | A | G | 0·290389 | -0·0619 | 0·0088 | 1·91E-12 | -0·49277 | 0·00829328 | 0 | 0·125616 | 0·0179829 | 2·8422E-12 | 0·03240798 | 20 | 3·224604E-45 | 3·224604E-45 | 3530·49713 |
|  |  |  |  |  |  |  |  |  |  |  |  |  |  |  |  |  |  |  |  |  |  |  |  |  |
| **Gastric cancer** | *BAK1* | 6 | ENSG00000030110 | 33544174 | rs210143 | 6 | 33546930 | T | C | 0·275051 | -0·0934 | 0·0187 | 5·664E-07 | 0·668346 | 0·0121344 | 0 | -0·139748 | 0·0280943 | 6·5503E-07 | 0·05710889 | 20 | 3·881417E-05 | 3·885343E-05 | 3033·65432 |

GWAS: genome-wide association study.

eQTL: expression quantitative trait loci.

SMR: summary-data-based Mendelian randomization.

HEIDI: heterogeneity in dependent instruments.

Only genome-wide significant eQTLs (*P*<5E-8) are taken into the analysis. We report SNP-gene combinations with *P*_SMR_ < genome-wide significance Bonferonni correction threshold of 0·05/nprobe (1013), and survived after the heterogeneity test (*P*_HEIDI_≥ 0·01).

β in GWAS association, regression coefficient of cancer on SNP, log(OR).

SE, standard error.

β in eQTL association, regression coefficient of gene expression on SNP.

β in SMR association, regression coefficient of cancer on gene expression.

PP.H4, posterior probability of H4 ,PP.H4 >0·8 as the cut-off for the evidence of colocalization of cancer GWAS and eQTL association.

PP.H3, posterior probability of H3.

PP.H4/PP.H3 >5 as strong evidence of colocalization.

**Supplementary Table 4.** SMR and colocalization results of the association between expression of mitochondrial-related genes and breast cancer risk classified by intrinsic–like subtypes.

| **Breast cancer subtype** | **Gene** | **Gene Chr.** | **Probe** | **Probe base pair** | **topSNP** | **SNP Chr.** | **SNP base pair** | **Effect allele** | **Other allele** | **Effect allele frequence** | **GWAS association** | | | **eQTL association** | | | **SMR association** | | | **HEIDI Test** | | **PP.H4** | **PP.H4/PP.H3** | **F-statistic** |
| --- | --- | --- | --- | --- | --- | --- | --- | --- | --- | --- | --- | --- | --- | --- | --- | --- | --- | --- | --- | --- | --- | --- | --- | --- |
|  |  |  |  |  |  |  |  |  |  |  | **β** | **SE** | ***P*** | **β** | **SE** | ***P*** | **β** | **SE** | ***P*** | ***P*** | **No. of SNPs** |  |  |  |
| **Luminal A-like** | *PARK7* | 1 | ENSG00000116288 | 8029958 | rs35675666 | 1 | 8021973 | T | G | 0·162577 | -0·0483038 | 0·0106126 | 5·3256E-06 | 0·409378 | 0·0107042 | 0 | -0·117993 | 0·0261068 | 6·1944E-06 | 0·01743469 | 20 | 0·01443609 | 0·01572557 | 20·4270289 |
|  | *MTX1* | 1 | ENSG00000173171 | 155181052 | rs370545 | 1 | 155175390 | A | G | 0·458078 | -0·047013 | 0·00790137 | 2·6818E-09 | -0·2981 | 0·0172663 | 8·6605E-67 | 0·157709 | 0·0280357 | 1·852E-08 | 0·03159388 | 20 | 0·9771768 | 42·81544 | 31·6439105 |
|  | *FDPS* | 1 | ENSG00000160752 | 155284498 | rs6677385 | 1 | 155304581 | A | C | 0·268916 | 0·0458709 | 0·00893957 | 2·8787E-07 | -0·0930443 | 0·0096413 | 4·8869E-22 | -0·493001 | 0·108815 | 5·8812E-06 | 0·08420154 | 20 | 0·855553 | 5·924082 | 20·5266495 |
|  | *MPC2* | 1 | ENSG00000143158 | 167896122 | rs203777 | 1 | 167876616 | T | C | 0·344581 | -0·0432845 | 0·00899372 | 1·4887E-06 | -0·0991542 | 0·00941492 | 6·1775E-26 | 0·436537 | 0·0997266 | 1·2014E-05 | 0·3284888 | 20 | 0·01215136 | 0·0143014 | 19·1610848 |
|  | *MRPS18C* | 4 | ENSG00000163319 | 84383986 | rs1565909 | 4 | 84400330 | C | T | 0·51227 | 0·0412441 | 0·00787341 | 1·6197E-07 | 0·141556 | 0,00794821 | 5·9323E-71 | 0·291363 | 0·0579765 | 5·0205E-07 | 0·4593665 | 20 | 0·0002474415 | 0·0002481989 | 25·2560142 |
|  | *HSCB* | 22 | ENSG00000100209 | 29145761 | rs6519752 | 22 | 28904181 | G | A | 0·151329 | 0·0584001 | 0·0106349 | 3·9884E-08 | 0·0785753 | 0·0114645 | 7·1914E-12 | 0·743237 | 0·173431 | 1·8233E-05 | 0·02447861 | 20 | 0·2640101 | 0·3587143 | 18·3654336 |
|  |  |  |  |  |  |  |  |  |  |  |  |  |  |  |  |  |  |  |  |  |  |  |  |  |
| **Luminal B-like/Her2-positive** | *MRPS18C* | 4 | ENSG00000163319 | 84383986 | rs1565909 | 4 | 84400330 | C | T | 0·51227 | 0·0829843 | 0·0175093 | 2·143E-06 | 0·141556 | 0·00794821 | 5·9323E-71 | 0·586229 | 0·127996 | 4·6486E-06 | 0·05397803 | 20 | 0·09895463 | 2·538312 | 20·9769239 |
|  |  |  |  |  |  |  |  |  |  |  |  |  |  |  |  |  |  |  |  |  |  |  |  |  |
| **Luminal B-like/Her2-negative** | *COX11* | 17 | ENSG00000166260 | 53037704 | rs1802212 | 17 | 53038654 | C | A | 0·273006 | -0·0712297 | 0·0169266 | 2·5747E-05 | -0·310731 | 0·00884191 | 1·489E-270 | 0·229233 | 0·0548627 | 2·937E-05 | 0·03670377 | 20 | 0·985343 | 89·03358 | 17·4582184 |

GWAS: genome-wide association study.

eQTL: expression quantitative trait loci.

SMR: summary-data-based Mendelian randomization.

HEIDI: heterogeneity in dependent instruments.

Only genome-wide significant eQTLs (*P*<5E-8) are taken into the analysis. We report SNP-gene combinations with *P*_SMR_ < genome-wide significance Bonferonni correction threshold of 0·05/nprobe (1013), and survived after the heterogeneity test (*P*_HEIDI_ ≥ 0·01).

β in GWAS association, regression coefficient of cancer on SNP, log(OR).

SE, standard error.

β in eQTL association, regression coefficient of gene expression on SNP.

β in SMR association, regression coefficient of cancer on gene expression.

PP.H4, posterior probability of H4 ,PP.H4 >0·8 as the cut-off for the evidence of colocalization of cancer GWAS and eQTL association.

PP.H3, posterior probability of H3.

PP.H4/PP.H3 >5 as strong evidence of colocalization.

**Supplementary Table 5.** SMR and colocalization results of the association between DNA methylation of mitochondrial-related genes and cancer outcomes.

| **Type of Cancer** | **Gene** | **Gene Chr.** | **Probe** | **Probe base pair** | **topSNP** | **SNP Chr.** | **SNP base pair** | **Effect allele** | **Other allele** | **Effect allele frequence** | **GWAS association** | | | **mQTL association** | | | **SMR association** | | | **HEIDI Test** | | **PP.H4** | **PP.H4/PP.H3** | **F-statistic** |
| --- | --- | --- | --- | --- | --- | --- | --- | --- | --- | --- | --- | --- | --- | --- | --- | --- | --- | --- | --- | --- | --- | --- | --- | --- |
|  |  |  |  |  |  |  |  |  |  |  | **β** | **SE** | ***P*** | **β** | **SE** | ***P*** | **β** | **SE** | ***P*** | ***P*** | **No. of SNPs** |  |  |  |
| **Breast cancer** | *NSUN4* | 1 | cg14993813 | 46806288 | rs6682266 | 1 | 46820419 | C | T | 0·273006 | 0·0313956 | 0·00655956 | 1·70E-06 | -0·351591 | 0·0346711 | 3·64E-24 | -0·0892957 | 0·0206304 | 1·50E-05 | 0·3784151 | 20 | 0·9616779 | 25·51203 | 102·834836 |
|  |  |  | cg17875957 | 46806823 | rs5013329 |  | 46815091 | T | C | 0·277096 | 0·0315499 | 0·00655667 | 1·50E-06 | -0·396092 | 0·0354094 | 4·77E-29 | -0·0796531 | 0·01802 | 9·86E-06 | 0·5884129 | 20 | 0·9616726 | 25·50829 | 125·128145 |
|  |  |  | cg17806798 | 46807113 | rs41293277 |  | 46806550 | T | C | 0·278119 | 0·0300078 | 0·00655178 | 4·65E-06 | -1·1206 | 0·0277209 | 0 | -0·0267783 | 0·00588408 | 5·34E-06 | 0·0435169 | 20 | 0·9616701 | 25·50658 | 1634·12993 |
|  |  |  | cg04241075 | 46807263 | rs41293277 |  | 46806550 | T | C | 0·278119 | 0·0300078 | 0·00655178 | 4·65E-06 | -1·03836 | 0·0297271 | 2·63E-267 | -0·0288992 | 0·00636375 | 5·59E-06 | 0·06870347 | 20 | 0·9616701 | 25·50658 | 1220·08703 |
|  |  |  | cg06741803 | 46807522 | rs56063031 |  | 46825433 | T | C | 0·273006 | 0·0314002 | 0·00655962 | 1·69E-06 | -0·356653 | 0·035562 | 1·14E-23 | -0·0880412 | 0·0203798 | 1·56E-05 | 0·5486061 | 20 | 0·9616798 | 25·51335 | 100·581801 |
|  |  |  | cg00530320 | 46809349 | rs6681857 |  | 46850124 | C | T | 0·269939 | 0·0317324 | 0·00658318 | 1·43E-06 | 0·471867 | 0·0353831 | 1·43E-40 | 0·0672486 | 0·0148347 | 5·81E-06 | 0·3668974 | 20 | 0·9617792 | 25·5835 | 177·847372 |
|  |  |  | cg15580309 | 46814106 | rs111226885 |  | 46814273 | C | T | 0·274029 | 0·0315579 | 0·00655995 | 1·50E-06 | 1·18987 | 0·0263506 | 0 | 0·0265221 | 0·00554436 | 1·72E-06 | 0·1344093 | 20 | 0·9616726 | 25·5083 | 2039·00369 |
|  | *SLC25A44* | 1 | cg19263494 | 156181616 | rs72708291 | 1 | 156177285 | T | A | 0·295501 | 0·0302793 | 0·00661688 | 4·74E-06 | -0·838381 | 0·0322159 | 2·66E-149 | -0·0361163 | 0·00801354 | 6·58E-06 | 0·01266423 | 20 | 0·0001574607 | 0·0001588429 | 677·239558 |
|  | *BCL2L11* | 2 | cg09907170 | 111877177 | rs73954926 | 8 | 111877175 | G | T | 0·0705521 | 0·058064 | 0·0123562 | 2·61E-06 | 0·816196 | 0·0651452 | 5·19E-36 | 0·0711397 | 0·0161686 | 1·08E-05 | 0·1647621 | 9 | 0·003485996 | 0·003970584 | 156·972683 |
|  |  |  | cg27608154 | 111881810 | rs73954941 |  | 111890379 | G | T | 0·0705521 | 0·0591282 | 0·0123417 | 1·66E-06 | -1·50991 | 0·0610033 | 3·00E-135 | -0·0391601 | 0·00832554 | 2·56E-06 | 0·07891563 | 12 | 0·003476838 | 0·003958707 | 612·626057 |
|  | *SLC25A22* | 11 | cg23587532 | 790669 | rs7928917 | 11 | 819464 | T | G | 0·449898 | -0·0365 | 0·00620721 | 4·10E-09 | 0·230005 | 0·0327618 | 2·21E-12 | -0·158692 | 0·0352031 | 6·55E-06 | 0·03096379 | 20 | 2·488377E-09 | 2·488377E-09 | 49·2877559 |
|  |  |  | cg11475788 | 790761 | rs61876744 | 11 | 820754 | T | C | 0·425358 | -0·039641 | 0·00639289 | 5·62E-10 | 0·212819 | 0·0333349 | 1·72E-10 | -0·186266 | 0·0418757 | 8·66E-06 | 0·01403131 | 6 | 2·488377E-09 | 2·488377E-09 | 40·7589027 |
|  | *MRPL23* | 11 | cg07977153 | 1967958 | rs4929956 | 11 | 1981785 | G | T | 0·490798 | -0·0425554 | 0·00599906 | 1·31E-12 | 0·263918 | 0·0334368 | 2·95E-15 | -0·161245 | 0·0305618 | 1·32E-07 | 0·4185561 | 20 | 1·05079E-30 | 1·05079E-30 | 62·3000805 |
|  | *TRMT1* | 19 | cg22237401 | 13228070 | rs74569397 | 19 | 13239298 | T | C | 0·0501022 | 0·0764907 | 0·0138189 | 3·11E-08 | 0·660504 | 0·0670614 | 6·91E-23 | 0·115807 | 0·0239994 | 1·40E-06 | 0·1458096 | 11 | 0·006813221 | 0·00686513 | 97·0075805 |
|  |  |  | cg12014333 | 13228096 | rs16995252 | 19 | 13243304 | G | A | 0·0501022 | 0·0760938 | 0·0138134 | 3·62E-08 | 0·586768 | 0·067351 | 2·98E-18 | 0·129683 | 0·0278529 | 3·22E-06 | 0·09108908 | 8 | 0·0068135 | 0·006865413 | 75·900519 |
|  |  |  |  |  |  |  |  |  |  |  |  |  |  |  |  |  |  |  |  |  |  |  |  |  |
| **Prostate Cancer** | *BIK* | 22 | cg07972488 | 43506347 | rs2294413 | 22 | 43471386 | C | G | 0·271984 | 0·0791 | 0·0089 | 6·837E-19 | 0·200209 | 0·0362848 | 3·43435E-08 | 0·395087 | 0·0842803 | 2·76188E-06 | 0·1348615 | 4 | 8·196814E-65 | 8·196814E-65 | 30·4451225 |
|  | *NSUN4* | 1 | cg04241075 | 46807263 | rs41293277 | 1 | 46806550 | T | C | 0·278119 | 0·0389 | 0·0088 | 0·00000966 | -1·03836 | 0·0297271 | 2·6293E-267 | -0·0374629 | 0·0085425 | 1·15732E-05 | 0·390968 | 20 | 0·9631414 | 27·35524 | 1220·08703 |
|  |  |  | cg15580309 | 46814106 | rs111226885 | 1 | 46814273 | C | T | 0·274029 | 0·0395 | 0·0088 | 0·00000707 | 1·18987 | 0·0263506 | 0 | 0·0331969 | 0·00743222 | 7·94641E-06 | 0·3263274 | 20 | 0·9631398 | 27·35394 | 2039·00369 |
|  |  |  | cg17806798 | 46807113 | rs41293277 | 1 | 46806550 | T | C | 0·278119 | 0·0389 | 0·0088 | 0·00000966 | -1·1206 | 0·0277209 | 0 | -0·0347135 | 0·00789975 | 1·11151E-05 | 0·3724526 | 20 | 0·9631414 | 27·35524 | 1634·12993 |
|  | *NUDT5* | 10 | cg22687873 | 12211796 | rs4750175 | 10 | 12200125 | T | C | 0·40184 | 0·0359 | 0·0081 | 9·282E-06 | -0·879303 | 0·0284161 | 3·0758E-210 | -0·0408278 | 0·00930585 | 1·14754E-05 | 0·6092653 | 20 | 0·9911689 | 151·9916 | 957·520685 |
|  | *TSPO* | 22 | cg13160331 | 43547217 | rs138909 | 22 | 43549739 | A | T | 0·371166 | 0·0654 | 0·0082 | 1·127E-15 | 0·193332 | 0·0333336 | 6·63496E-09 | 0·338278 | 0·0721161 | 2·72209E-06 | 0·1768039 | 5 | 1·381557E-65 | 1·381557E-65 | 33·6389978 |
|  | *VARS2* | 6 | cg02186769 | 30881645 | rs2233959 | 6 | 31081065 | C | T | 0·416155 | -0·0426 | 0·0081 | 0·00000016 | 0·242893 | 0·0322969 | 5·45156E-14 | -0·175386 | 0·0406933 | 1·63294E-05 | 0·03362298 | 20 | 0·008666735 | 0·008742504 | 56·5598593 |
|  |  |  | cg05103231 | 30881664 | rs2524108 | 6 | 31232451 | G | C | 0·247444 | -0·0523 | 0·0091 | 1·078E-08 | 0·238925 | 0·0355083 | 1·71174E-11 | -0·218897 | 0·0500895 | 1·24179E-05 | 0·05304481 | 20 | 4·972874E-09 | 4·972874E-09 | 45·2755181 |
|  |  |  | cg12457901 | 30882671 | rs2233980 | 6 | 31079644 | A | G | 0·0838446 | -0·0607 | 0·0125 | 0·00000115 | 0·858528 | 0·0402543 | 6·2923E-101 | -0·0707024 | 0·0149324 | 2·19248E-06 | 0·04378673 | 20 | 0·008666735 | 0·008742504 | 454·866936 |
|  |  |  | cg16113650 | 30883959 | rs2249935 | 6 | 31327178 | A | G | 0·174847 | -0·0721 | 0·01 | 4·735E-13 | 0·201305 | 0·0366363 | 3·914E-08 | -0·358163 | 0·0819547 | 1·24102E-05 | 0·2379885 | 19 | 2·629957E-09 | 2·629958E-09 | 30·1915848 |
|  |  |  | cg26467571 | 30882355 | rs9264490 | 6 | 31232578 | G | A | 0·225971 | -0·0538 | 0·0093 | 7·104E-09 | 0·282467 | 0·0345632 | 3·02113E-16 | -0·190465 | 0·040338 | 2·3386E-06 | 0·6083029 | 20 | 4·972874E-09 | 4·972874E-09 | 66·7894006 |
|  |  |  |  |  |  |  |  |  |  |  |  |  |  |  |  |  |  |  |  |  |  |  |  |  |
| **Gastric cancer** | *BAK1* | 6 | cg00700324 | 33548127 | rs511515 | 6 | 33541507 | A | G | 0·279141 | -0·0938 | 0·0186 | 4·887E-07 | -0·27958 | 0·0327629 | 1·42059E-17 | 0·335503 | 0·0772774 | 1·41484E-05 | 0·03645502 | 11 | 0·0004891538 | 0·0004898692 | 72·8194725 |
|  |  |  |  |  |  |  |  |  |  |  |  |  |  |  |  |  |  |  |  |  |  |  |  |  |
| **Lung cancer** | *VARS2* | 6 | cg12457901 | 30882671 | rs2523593 | 6 | 31326703 | C | T | 0·0817996 | 0·164368 | 0·032813 | 9·6301E-09 | 0·837244 | 0·0418738 | 6·15371E-89 | 0·19632 | 0·0404029 | 1·17943E-06 | 0·01778986 | 20 | 0·9821793 | 61·31067 | 399·778412 |
|  |  |  | cg14935711 | 30882694 | rs2523593 | 6 | 31326703 | C | T | 0·0817996 | 0·164368 | 0·032813 | 9·6301E-09 | 0·76331 | 0·0426531 | 1·27203E-71 | 0·215336 | 0·0446401 | 1·40832E-06 | 0·01347948 | 20 | 0·9821793 | 61·31067 | 320·258527 |
|  |  |  | cg15848685 | 30882641 | rs2596500 | 6 | 31321267 | C | A | 0·0828221 | 0·163304 | 0·033766 | 3·23E-08 | 0·797449 | 0·0423235 | 3·43521E-79 | 0·204783 | 0·0437152 | 2·80665E-06 | 0·01645825 | 20 | 0·9820769 | 60·95388 | 355·011716 |
|  |  |  | cg16958594 | 30882708 | rs2596495 | 6 | 31323416 | C | G | 0·0940695 | 0·150983 | 0·032566 | 1·62E-07 | 0·753746 | 0·0423127 | 5·53093E-71 | 0·20031 | 0·0446448 | 7·23183E-06 | 0·03534323 | 20 | 0·9820769 | 60·95388 | 317·328123 |
|  |  |  |  |  |  |  |  |  |  |  |  |  |  |  |  |  |  |  |  |  |  |  |  |  |
| **Melanoma** | *SPG7* | 16 | cg09560549 | 89573369 | rs8060502 | 16 | 89589408 | G | A | 0·350716 | 0·00120683 | 0·00018773 | 1·3E-10 | 0·385641 | 0·0339469 | 6·60424E-30 | 0·00312941 | 0·00055935 | 2·20904E-08 | 0·01973762 | 20 | 5·532095E-20 | 5·532095E-20 | 129·052422 |
|  |  |  | cg15206445 | 89573312 | rs8060502 | 16 | 89589408 | G | A | 0·350716 | 0·00120683 | 0·00018773 | 1·3E-10 | 0·444993 | 0·0332731 | 8·58333E-41 | 0·00271202 | 0·00046808 | 6·87807E-09 | 0·01286307 | 20 | 5·532095E-20 | 5·532095E-20 | 178,862719 |

GWAS: genome-wide association study.

mQTL: methylation quantitative trait loci.

SMR: summary-data-based Mendelian randomization.

HEIDI: heterogeneity in dependent instruments.

Only genome-wide significant mQTLs (*P*<5E-8) are taken into the analysis. We report SNP-gene combinations with *P*_SMR_ < genome-wide significance Bonferonni correction threshold of 0·05/nprobe (2550), and survived after the heterogeneity test (*P*_HEIDI_ ≥ 0·01).

β in GWAS association, regression coefficient of cancer on SNP, log(OR).

SE, standard error.

β in mQTL association, regression coefficient of DNA methylation on SNP.

β in SMR association, regression coefficient of cancer on DNA methylation.

PP.H4, posterior probability of H4 ,PP.H4 >0·8 as the cut-off for the evidence of colocalization of cancer GWAS and mQTL association.

PP.H3, posterior probability of H3.

PP.H4/PP.H3 >5 as strong evidence of colocalization.

**Supplementary Table 6.** Sensitivity analysis used TwoSampleMR package on the association between DNA methylation of mitochondrial-related genes and cancer outcomes.

| **Type of cancer** | **MR method** | **No. of SNP** | **OR (95% CI)** | ***P*-value** | **Heterogeneity test** | | **Pleiotropy test** | |
| --- | --- | --- | --- | --- | --- | --- | --- | --- |
|  |  |  |  |  | **Cochran's Q** | ***P*** | **intercept** | ***P*** |
| **Breast cancer** | MR Egger | 14 | 2·72 (2·35-3·08) | 0·000164698838047467 | 1·90E-10 | 1 | -3·00E-08 | 1·00 |
|  | Weighted median | 14 | 2·72 (2·54-2·89) | 7·51804195952311E-29 | NA | NA | NA | NA |
|  | Inverse variance weighted | 14 | 2·72 (2·81-2·61) | 4·46448442617676E-86 | 2·08E-10 | 1 | NA | NA |
|  | Simple mode | 14 | 2·72 (2·45-2·98) | 5·08685683311402E-06 | NA | NA | NA | NA |
|  | Weighted mode | 14 | 2·72 (2·45-2·99) | 5·99683071933978E-06 | NA | NA | NA | NA |
|  | MR-PRESSO | NA | NA | 1 | NA | NA | NA | NA |
|  |  |  |  |  |  |  |  |  |
| **Prostate cancer** | MR Egger | 10 | 2·72 (2·33-3·10) | 0·000936604462163083 | 1·96E-30 | 1 | 2·02E-17 | 1·00 |
|  | Weighted median | 10 | 2·72 (2·54-2·89) | 1·56240620034785E-29 | NA | NA | NA | NA |
|  | Inverse variance weighted | 10 | 2·72 (2·62-2·82) | 8·1694216761376E-83 | 2·37E-30 | 1 | - | - |
|  | Simple mode | 10 | 2·72 (2·49-2·95) | 0·0000122280803646251 | NA | NA | NA | NA |
|  | Weighted mode | 10 | 2·72 (2·48-2·95) | 0·0000164228109845806 | NA | NA | NA | NA |
|  | MR-PRESSO | NA | NA | 1 | NA | NA | NA | NA |
|  |  |  |  |  |  |  |  |  |
| **Gastric cancer** | Wald ratio | 1 | 2·72 (2·33-3·11) | 4·58263488815543E-07 | NA | NA | NA | NA |
|  |  |  |  |  |  |  |  |  |
| **Lung cancer** | MR Egger | 3 | 2·72 (2·11-3·33) | 0·80221698961478 | 1·93E-34 | 1 | 1·02E-16 | 1·00 |
|  | Weighted median | 3 | 2·72 (2·31-3·13) | 1·94781286030451E-06 | NA | NA | NA | NA |
|  | Inverse variance weighted | 3 | 2·72 (NA) | NA | 0 | 1 | NA | NA |
|  | Simple mode | 3 | 2·72 (2·31-3·13) | 0·0413179056908491 | NA | NA | NA | NA |
|  | Weighted mode | 3 | 2·72 (2·28-3·15) | 0·0458119658393948 | NA | NA | NA | NA |
|  | MR-PRESSO | NA | NA | 1 | NA | NA | NA | NA |
|  |  |  |  |  |  |  |  |  |
| **Melanoma** | Wald ratio | 1 | 2·72 (2·41-3·02) | 1·289216E-10 | NA | NA | NA | NA |

NA: not applicable.

Cochran Q statistic implemented in MR Egger and IVW method, *P*>0·05 indicates no heterogeneity exists.

The intercept of MR Egger can be used to indicate whether directional horizontal pleiotropy is driving the results of MR analysis, there are no directional pleiotropies if *P* >0·05.

MR-PRESSO can detect and adjust for any outliers reflecting horizontal pleiotropic biases, where *p* value for Global test >0·05 indicates no horizontal pleiotropic outliers.

**Supplementary Table 7.** SMR and colocalization results of the association between DNA methylation of mitochondrial-related genes and luminal A-like breast cancer risk.

| **Gene** | **Gene Chr.** | **Probe** | **Probe base pair** | **topSNP** | **SNP Chr.** | **SNP base pair** | **Effect allele** | **Other allele** | **Effect allele frequence** | **GWAS association** | | | **mQTL association** | | | **SMR association** | | | **HEIDI Test** | | **PP.H4** | **PP.H4/PP.H3** | **F-statistic** |
| --- | --- | --- | --- | --- | --- | --- | --- | --- | --- | --- | --- | --- | --- | --- | --- | --- | --- | --- | --- | --- | --- | --- | --- |
|  |  |  |  |  |  |  |  |  |  | **β** | **SE** | ***P*** | **β** | **SE** | ***P*** | **β** | **SE** | ***P*** | ***P*** | **No. of SNPs** |  |  |  |
| *NSUN4* | 1 | cg00530320 | 46809349 | rs6681857 | 1 | 46850124 | C | T | 0·269939 | 0·046647 | 0·008637 | 6·63E-08 | 0·471867 | 0·035383 | 1·43E-40 | 0·098856 | 0·019747 | 5·56E-07 | 0·210661 | 20 | 0·9588827 | 23·33967 | 177·848377 |
|  |  | cg02459555 | 46806155 | rs12137934 |  | 46728913 | G | A | 0·282209 | 0·04623 | 0·008582 | 7·16E-08 | 0·273732 | 0·036386 | 5·35E-14 | 0·168888 | 0·03856 | 0·0000119 | 0·076727 | 14 | 5·707147E-05 | 5·713086E-05 | 56·5955794 |
|  |  | cg04241075 | 46807263 | rs41293277 |  | 46806550 | T | C | 0·278119 | 0·044065 | 0·008605 | 3·04E-07 | -1·03836 | 0·029727 | 2·6E-267 | -0·04244 | 0·008375 | 4·04E-07 | 0·039662 | 20 | 0·9585626 | 23·15148 | 1220·09524 |
|  |  | cg06741803 | 46807522 | rs56063031 |  | 46825433 | T | C | 0·273006 | 0·045898 | 0·008608 | 9·71E-08 | -0·35665 | 0·035562 | 1·14E-23 | -0·12869 | 0·027334 | 0·0000025 | 0·295729 | 20 | 0·9585668 | 23·15393 | 100·580109 |
|  |  | cg08259313 | 46806025 | rs6662982 |  | 46877180 | A | G | 0·286299 | 0·0444092 | 0·008516 | 1·8402E-07 | 0·310969 | 0·0353937 | 1·5497E-18 | 0·142809 | 0·0318458 | 7·3122E-06 | 0·1842912 | 11 | 0·9589455 | 23·37692 | 77·1937721 |
|  |  | cg14993813 | 46806288 | rs6682266 |  | 46820419 | C | T | 0·273006 | 0·0459011 | 0·0086078 | 9·6871E-08 | -0·351591 | 0·0346711 | 3·6425E-24 | -0·130552 | 0·027661 | 2·3615E-06 | 0·3041494 | 20 | 0·9585655 | 23·15319 | 102·834836 |
|  |  | cg15580309 | 46814106 | rs111226885 |  | 46814273 | C | T | 0·274029 | 0·0459566 | 0·00860855 | 9·3728E-08 | 1·18987 | 0·0263506 | 0 | 0·0386232 | 0·00728525 | 1·1482E-07 | 0·09481344 | 20 | 0·9585647 | 23·15269 | 2039·00369 |
|  |  | cg17806798 | 46807113 | rs41293277 |  | 46806550 | T | C | 0·278119 | 0·044065 | 0·0086046 | 3·0376E-07 | -1·1206 | 0·0277209 | 0 | -0·0393227 | 0·00773993 | 3·7645E-07 | 0·0154515 | 20 | 0·9585626 | 23·15148 | 1634·12993 |
|  |  | cg17875957 | 46806823 | rs5013329 |  | 46815091 | T | C | 0·277096 | 0·0465052 | 0·00860214 | 6·4364E-08 | -0·396092 | 0·0354094 | 4·7719E-29 | -0·11741 | 0·0241209 | 1·1299E-06 | 0·518721 | 20 | 0·958565 | 23·1529 | 125·128145 |
| *MRPL23* | 11 | cg07578618 | 1968453 | rs9735581 | 11 | 1976041 | G | A | 0·481595 | -0·0382687 | 0·00800948 | 1·7711E-06 | 0·817707 | 0·0288757 | 2·065E-176 | -0·0468 | 0·00993349 | 2·461E-06 | 0·0281693 | 20 | 7·422245E-22 | 7·422245E-22 | 801·918788 |
|  |  | cg07977153 | 1967958 | rs4929956 |  | 1981785 | G | T | 0·490798 | -0·0424522 | 0·00788002 | 7·1514E-08 | 0·263918 | 0·0334368 | 2·949E-15 | -0·160854 | 0·0361497 | 8·6007E-06 | 0·2146185 | 20 | 7·422245E-22 | 7·422245E-22 | 62·3000805 |
| *SLC25A22* | 11 | cg09336922 | 790795 | rs4963156 | 11 | 780827 | T | C | 0·435583 | 0·0390652 | 0·00812739 | 1·5351E-06 | -0·49226 | 0·0314805 | 4·0754E-55 | -0·0793589 | 0·0172728 | 4·3388E-06 | 0·01080128 | 20 | 0·000981894 | 0·001009489 | 244·515194 |
| *BCL2L11* | 2 | cg09907170 | 111877177 | rs73954926 | 2 | 111877175 | G | T | 0·0705521 | 0·0769372 | 0·0161119 | 1·7954E-06 | 0·816196 | 0·0651452 | 5·1897E-36 | 0·0942631 | 0·0211254 | 8·1167E-06 | 0·2262169 | 9 | 0·002152175 | 0·002337057 | 156·972683 |
|  |  | cg27608154 | 111881810 | rs73954941 |  | 111890379 | G | T | 0·0705521 | 0·0785443 | 0·0160912 | 1·0545E-06 | -1·50991 | 0·0610033 | 3·004E-135 | -0·0520192 | 0·0108623 | 1·6766E-06 | 0·1369691 | 12 | 0·002151483 | 0·002336241 | 612·626057 |
| *TRMT1* | 19 | cg12014333 | 13228096 | rs16995252 | 19 | 13243304 | G | A | 0·0501022 | 0·116153 | 0·0180297 | 1·1766E-10 | 0·586768 | 0·067351 | 2·9833E-18 | 0·197953 | 0·0382155 | 2·2198E-07 | 0·01324548 | 8 | 2·816313E-05 | 2·816392E-05 | 75·900519 |
|  |  | cg22237401 | 13228070 | rs74569397 |  | 13239298 | T | C | 0·0501022 | 0·116772 | 0·0180371 | 9·5453E-11 | 0·660504 | 0·0670614 | 6·9063E-23 | 0·176792 | 0·0326792 | 6·3048E-08 | 0·02282565 | 11 | 2·816312E-05 | 2·816392E-05 | 97·0075805 |

GWAS: genome-wide association study.

mQTL: methylation quantitative trait loci.

SMR: summary-data-based Mendelian randomization.

HEIDI: heterogeneity in dependent instruments.

Only genome-wide significant mQTLs (*P*<5E-8) are taken into the analysis. We report SNP-gene combinations with *P*_SMR_ < genome-wide significance Bonferonni correction threshold of 0·05/nprobe (2550), and survived after the heterogeneity test (*P*_HEIDI_ ≥ 0·01).

β in GWAS association, regression coefficient of cancer on SNP, log(OR).

SE, standard error.

β in mQTL association, regression coefficient of DNA methylation on SNP.

β in SMR association, regression coefficient of cancer on DNA methylation.

PP.H4, posterior probability of H4 ,PP.H4 >0·8 as the cut-off for the evidence of colocalization of cancer GWAS and mQTL association.

PP.H3, posterior probability of H3.

PP.H4/PP.H3 >5 as strong evidence of colocalization.

**Supplementary Table 8.** SMR results of the association between DNA methylation and expression of mitochondrial-related genes.

| **Expo Gene** | **Expo Chr.** | **Expo Probe** | **Expo base pair** | **Outco Gene** | **Outco Chr.** | **Outco Probe** | **Outco base pair** | **topSNP** | **SNP Chr.** | **SNP base pair** | **Effect allele** | **Other allele** | **Effect allele frequence** | **eQTL association** | | | **mQTL association** | | | **SMR association** | | | **HEIDI Test** | |
| --- | --- | --- | --- | --- | --- | --- | --- | --- | --- | --- | --- | --- | --- | --- | --- | --- | --- | --- | --- | --- | --- | --- | --- | --- |
|  |  |  |  |  |  |  |  |  |  |  |  |  |  | **β** | **SE** | ***P*** | **β** | **SE** | ***P*** | **β** | **SE** | ***P*** | ***P*** | **No. of SNPs** |
| *LDHD* | 16 | cg00004883 | 75148460 | *LDHD* | 16 | ENSG00000166816 | 75148213 | rs117601275 | 16 | 75091078 | T | C | 0·0255624 | -0·242199 | 0·0361556 | 2·1014E-11 | -1·39513 | 0·136189 | 1·258E-24 | 0·173603 | 0·0309646 | 2·0645E-08 | 0·0153974 | 3 |
| *METTL5* | 2 | cg00010932 | 171000000 | *METTL5* | 2 | ENSG00000138382 | 170674016 | rs72878187 | 2 | 170669593 | T | A | 0·129857 | 0·0834173 | 0·0115409 | 4·9016E-13 | -0·254241 | 0·0458273 | 2·8929E-08 | -0·328103 | 0·0745536 | 1·078E-05 | 0·1309293 | 9 |
| *METTL5* | 2 | cg00010932 | 171000000 | *FASTKD1* | 2 | ENSG00000138399 | 170408322 | rs72878187 | 2 | 170669593 | T | A | 0·129857 | 0·193603 | 0·0170608 | 7·6052E-30 | -0·254241 | 0·0458273 | 2·8929E-08 | -0·761494 | 0·152786 | 6·2264E-07 | 0·02782183 | 15 |
| *CBR4* | 4 | cg00020991 | 170000000 | *CBR4* | 4 | ENSG00000145439 | 169858173 | rs28421942 | 4 | 169810818 | C | G | 0·135992 | 0·542963 | 0·0119583 | 0 | -0·420903 | 0·0497539 | 2·6806E-17 | -1·29 | 0·155111 | 9·0537E-17 | 0·01569198 | 14 |
| *LONP1* | 19 | cg00032366 | 5692159 | *LONP1* | 19 | ENSG00000196365 | 5706214 | rs2184854 | 19 | 5638815 | T | C | 0·349693 | -0·386646 | 0·00809983 | 0 | -0·221587 | 0·0338172 | 5·6582E-11 | 1·74489 | 0·268792 | 8·4915E-11 | 0·07257188 | 5 |
| *LETMD1* | 12 | cg00097794 | 51442472 | *COX14* | 12 | ENSG00000178449 | 50532482 | rs55864652 | 12 | 51490334 | T | C | 0·138037 | -0·0803011 | 0·0127067 | 2·6224E-10 | -0·287908 | 0·0480464 | 2·0691E-09 | 0·278912 | 0·0641429 | 1·372E-05 | 0·07369838 | 10 |
| *COMT* | 22 | cg00107488 | 19930437 | *RTL10* | 22 | ENSG00000215012 | 19838040 | rs2020917 | 22 | 19928884 | T | C | 0·281186 | -0·0771245 | 0·00984308 | 4·6735E-15 | -0·261874 | 0·0357713 | 2·4653E-13 | 0·29451 | 0·0550562 | 8·8315E-08 | 0·07923261 | 10 |
| *TXNRD2* | 22 | cg00107488 | 19930437 | *RTL10* | 22 | ENSG00000215012 | 19838040 | rs2020917 | 22 | 19928884 | T | C | 0·281186 | -0·0771245 | 0·00984308 | 4·6735E-15 | -0·261874 | 0·0357713 | 2·4653E-13 | 0·29451 | 0·0550562 | 8·8315E-08 | 0·07923261 | 10 |
| *SLC25A30* | 13 | cg00139037 | 45992673 | *SLC25A30* | 13 | ENSG00000174032 | 45980020 | rs9534108 | 13 | 45919515 | G | T | 0·465235 | -0·0821375 | 0·00874231 | 5·7001E-21 | -0·227459 | 0·0313472 | 3·9837E-13 | 0·361109 | 0·06288 | 9·3109E-09 | 0·01714581 | 18 |
| *PRDX2* | 19 | cg00155609 | 12912527 | *PRDX2* | 19 | ENSG00000167815 | 12910164 | rs722572 | 19 | 12811045 | C | T | 0·388548 | 0·177642 | 0·00984876 | 9·982E-73 | -0·189193 | 0·0333847 | 1·4527E-08 | -0·938946 | 0·17367 | 6·4274E-08 | 0·04985988 | 7 |
| *MTHFS* | 15 | cg00225070 | 80189496 | *BCL2A1* | 15 | ENSG00000140379 | 80258509 | rs8039962 | 15 | 80197122 | T | C | 0·46319 | -0·13001 | 0·0120648 | 4·469E-27 | 0·210047 | 0·0324968 | 1·0223E-10 | -0·618957 | 0·111666 | 2·9741E-08 | 0·7026741 | 6 |
| *NT5DC2* | 3 | cg00317010 | 52569752 | *NT5DC2* | 3 | ENSG00000168268 | 52563728 | rs72947580 | 3 | 52615483 | A | G | 0·00920245 | 0·594198 | 0·0297539 | 9·9652E-89 | -0·674176 | 0·100378 | 1·8631E-11 | -0·881369 | 0·13845 | 1·9404E-10 | 0·3417587 | 9 |
| *MRPL24* | 1 | cg00346446 | 157000000 | *MRPL24* | 1 | ENSG00000143314 | 156709238 | rs12140437 | 1 | 156686005 | C | A | 0·322086 | 0·0510066 | 0·00849063 | 1·8852E-09 | 0·217665 | 0·0349052 | 4·4924E-10 | 0·234335 | 0·0541641 | 1·5157E-05 | 0·323358 | 5 |
| *TUFM* | 16 | cg00348858 | 28858442 | *TUFM* | 16 | ENSG00000178952 | 28855730 | rs62037363 | 16 | 28865042 | C | T | 0·332311 | 0·832711 | 0·00671294 | 0 | -0·285293 | 0·0336199 | 2·1418E-17 | -2·91879 | 0·344764 | 2·5386E-17 | 0·03693695 | 20 |
| *GLS2* | 12 | cg00377653 | 56882535 | *GLS2* | 12 | ENSG00000135423 | 56873467 | rs2657909 | 12 | 56895503 | T | C | 0·337423 | 0·0639721 | 0·00846326 | 4·068E-14 | 0·5698 | 0·032949 | 5·2771E-67 | 0·112271 | 0·0162099 | 4·3264E-12 | 0·01372705 | 12 |
| *ACSM3* | 16 | cg00394823 | 20775166 | *ACSM3* | 16 | ENSG00000005187 | 20715234 | rs10852246 | 16 | 20733134 | A | G | 0·126789 | -0·218156 | 0·0115184 | 5·36E-80 | 0·38032 | 0·0480088 | 2·3395E-15 | -0·573612 | 0·0784872 | 2·7045E-13 | 0·1568541 | 18 |
| *ACSM3* | 16 | cg00394823 | 20775166 | *ACSM1* | 16 | ENSG00000166743 | 20672385 | rs10852246 | 16 | 20733134 | A | G | 0·126789 | 0·16318 | 0·0115867 | 4·8042E-45 | 0·38032 | 0·0480088 | 2·3395E-15 | 0·42906 | 0·0621418 | 5·0372E-12 | 0·1410977 | 18 |
| *PPOX* | 1 | cg00397740 | 161000000 | *PPOX* | 1 | ENSG00000143224 | 161142001 | rs148385119 | 1 | 161071090 | T | C | 0·0184049 | -0·186885 | 0·0281665 | 3·245E-11 | 1·04965 | 0·114425 | 4·5908E-20 | -0·178045 | 0·0331179 | 7·6115E-08 | 0·05091882 | 8 |
| *SPG7* | 16 | cg00439318 | 89601909 | *SPG7* | 16 | ENSG00000197912 | 89590750 | rs382745 | 16 | 89603586 | G | A | 0·485685 | 0·183672 | 0·00790409 | 1·899E-119 | -0·313204 | 0·0333143 | 5·3791E-21 | -0·586429 | 0·0672878 | 2·9015E-18 | 0·0125824 | 20 |
| *SPG7* | 16 | cg00439318 | 89601909 | *LOC101930112* | 16 | ENSG00000197912 | 89590750 | rs382745 | 16 | 89603586 | G | A | 0·485685 | 0·183672 | 0·00790409 | 1·899E-119 | -0·313204 | 0·0333143 | 5·3791E-21 | -0·586429 | 0·0672878 | 2·9015E-18 | 0·0125824 | 20 |
| *TIMM44* | 19 | cg00466492 | 7991731 | *TIMM44* | 19 | ENSG00000104980 | 8000204 | rs35810163 | 19 | 7999491 | A | G | 0·0869121 | 0·373668 | 0·015828 | 3·19E-123 | -0·615951 | 0·0592106 | 2·4096E-25 | -0·606652 | 0·0637273 | 1·7401E-21 | 0·3215036 | 14 |
| *NME3* | 16 | cg00485296 | 1821559 | *MRPS34* | 16 | ENSG00000074071 | 1822523 | rs2575369 | 16 | 1817431 | C | T | 0·445808 | 0·129004 | 0·00802773 | 4·1542E-58 | -0·213984 | 0·0324573 | 4·3166E-11 | -0·602868 | 0·09884 | 1·0645E-09 | 0·2447749 | 20 |
| *MMADHC* | 2 | cg00486525 | 150000000 | *MMADHC* | 2 | ENSG00000168288 | 150435239 | rs4667420 | 2 | 150443689 | C | G | 0·115542 | 0·135205 | 0·0150526 | 2·6541E-19 | -0·304989 | 0·0556898 | 4·3366E-08 | -0·443311 | 0·0948064 | 2·9258E-06 | 0·482229 | 5 |
| *NSUN4* | 1 | cg00530320 | 46809349 | *NSUN4* | 1 | ENSG00000117481 | 46818336 | rs6681857 | 1 | 46850124 | C | T | 0·269939 | 0·648365 | 0·00803049 | 0 | 0·471867 | 0·0353831 | 1·4302E-40 | 1·37404 | 0·104429 | 1·5376E-39 | 0·3311124 | 20 |
| *NDUFS6* | 5 | cg00561739 | 1807733 | *NDUFS6* | 5 | ENSG00000145494 | 1809116 | rs13187162 | 5 | 1799865 | A | G | 0·125767 | -0·1029 | 0·01754 | 4·4482E-09 | -0·35179 | 0·0484942 | 4·0382E-13 | 0·292504 | 0·0641231 | 5·0765E-06 | 0·4033808 | 4 |
| *ABHD10* | 3 | cg00569620 | 112000000 | *ABHD10* | 3 | ENSG00000144827 | 111705033 | rs9288933 | 3 | 111698898 | T | C | 0·221881 | 0·0972293 | 0·0100562 | 4·0995E-22 | 0·238622 | 0·0381368 | 3·9244E-10 | 0·407462 | 0·0775676 | 1·4965E-07 | 0·6308358 | 3 |
| *SCP2* | 1 | cg00581603 | 53442535 | *SCP2* | 1 | ENSG00000116171 | 53455138 | rs56222568 | 1 | 53432786 | C | T | 0·351738 | -0·721517 | 0·00745799 | 0 | -0·343514 | 0·033666 | 1·9108E-24 | 2·1004 | 0·206991 | 3·4064E-24 | 0·2650841 | 20 |
| *SCP2* | 1 | cg00581603 | 53442535 | *ECHDC2* | 1 | ENSG00000121310 | 53377270 | rs56222568 | 1 | 53432786 | C | T | 0·351738 | -0·56109 | 0·00788291 | 0 | -0·343514 | 0·033666 | 1·9108E-24 | 1·63338 | 0·161716 | 5·5054E-24 | 0·1034205 | 20 |
| *PUS1* | 12 | cg00588090 | 132000000 | *PUS1* | 12 | ENSG00000177192 | 132421075 | rs6598176 | 12 | 132425448 | A | G | 0·113497 | 0·0621385 | 0·0114723 | 6·0811E-08 | 0·469773 | 0·0440816 | 1·6192E-26 | 0·132273 | 0·0273941 | 1·3754E-06 | 0·03955732 | 9 |
| *LAP3* | 4 | cg00598235 | 17580680 | *LAP3* | 4 | ENSG00000002549 | 17594205 | rs62296304 | 4 | 17578253 | G | A | 0·348671 | 0·781981 | 0·00744299 | 0 | -0·383243 | 0·0321994 | 1·1529E-32 | -2·04043 | 0·17253 | 2·8466E-32 | 0·1206794 | 20 |
| *CYP11A1* | 15 | cg00615746 | 74658981 | *CYP11A1* | 15 | ENSG00000140459 | 74645090 | rs41556118 | 15 | 74633003 | A | C | 0·273006 | 0·0631945 | 0·00901465 | 2·3798E-12 | 0·321514 | 0·0366495 | 1·7446E-18 | 0·196553 | 0·0358905 | 4·3391E-08 | 0·03230388 | 5 |
| *MSRA* | 8 | cg00629382 | 10268916 | *MSRA* | 8 | ENSG00000175806 | 10099089 | rs12541491 | 8 | 10250048 | T | C | 0·317996 | -0·282966 | 0·00817741 | 2·248E-262 | 0·235296 | 0·0333254 | 1·6584E-12 | -1·2026 | 0·173835 | 4·5803E-12 | 0·2202544 | 20 |
| *ECHDC2* | 1 | cg00653085 | 53385434 | *SCP2* | 1 | ENSG00000116171 | 53455138 | rs1242331 | 1 | 53392908 | A | G | 0·349693 | -0·724308 | 0·00742631 | 0 | 0·293063 | 0·0331153 | 8·7688E-19 | -2·47151 | 0·280421 | 1·2121E-18 | 0·04790471 | 20 |
| *COASY* | 17 | cg00685795 | 40713781 | *COASY* | 17 | ENSG00000068120 | 40715890 | rs4793056 | 17 | 40748737 | G | C | 0·477505 | -0·0523296 | 0·00803587 | 7·4157E-11 | -0·208985 | 0·0326665 | 1·579E-10 | 0·250399 | 0·0548678 | 5·0267E-06 | 0·02907828 | 11 |
| *BAK1* | 6 | cg00700324 | 33548127 | *BAK1* | 6 | ENSG00000030110 | 33544174 | rs511515 | 6 | 33541507 | A | G | 0·279141 | 0·645085 | 0·0122857 | 0 | -0·27958 | 0·0327629 | 1·4206E-17 | -2·30734 | 0·273935 | 3·6719E-17 | 0·575253 | 8 |
| *MLYCD* | 16 | cg00739973 | 83937326 | *MLYCD* | 16 | ENSG00000103150 | 83941259 | rs28519996 | 16 | 83951462 | G | C | 0·0429448 | -0·173804 | 0·0190725 | 8·0283E-20 | -0·575238 | 0·0771884 | 9·1668E-14 | 0·302143 | 0·0523742 | 7·9778E-09 | 0·6416 | 15 |
| *HADHB* | 2 | cg00747065 | 26467663 | *HADHA* | 2 | ENSG00000084754 | 26440549 | rs11126405 | 2 | 26446695 | A | G | 0·209611 | -0·237564 | 0·0099915 | 5·833E-125 | -0·334471 | 0·0399499 | 5·6523E-17 | 0·710268 | 0·0899416 | 2·8572E-15 | 0·04680708 | 20 |
| *HADHA* | 2 | cg00747065 | 26467663 | *HADHA* | 2 | ENSG00000084754 | 26440549 | rs11126405 | 2 | 26446695 | A | G | 0·209611 | -0·237564 | 0·0099915 | 5·833E-125 | -0·334471 | 0·0399499 | 5·6523E-17 | 0·710268 | 0·0899416 | 2·8572E-15 | 0·04680708 | 20 |
| *HADHB* | 2 | cg00747065 | 26467663 | *HADHB* | 2 | ENSG00000138029 | 26489687 | rs11126405 | 2 | 26446695 | A | G | 0·209611 | 0·151916 | 0·0100868 | 2·9322E-51 | -0·334471 | 0·0399499 | 5·6523E-17 | -0·454198 | 0·0620691 | 2·5243E-13 | 0·03427468 | 20 |
| *HADHA* | 2 | cg00747065 | 26467663 | *HADHB* | 2 | ENSG00000138029 | 26489687 | rs11126405 | 2 | 26446695 | A | G | 0·209611 | 0·151916 | 0·0100868 | 2·9322E-51 | -0·334471 | 0·0399499 | 5·6523E-17 | -0·454198 | 0·0620691 | 2·5243E-13 | 0·03427468 | 20 |
| *LIG3* | 17 | cg00762550 | 33306307 | *LIG3* | 17 | ENSG00000005156 | 33319798 | rs17668722 | 17 | 33406488 | C | T | 0·0869121 | 0·393001 | 0·0130618 | 6·995E-199 | 0·387395 | 0·0594903 | 7·4203E-11 | 1·01447 | 0·159394 | 1·9587E-10 | 0·029698 | 7 |
| *PRELID1* | 5 | cg00848461 | 177000000 | *PRELID1* | 5 | ENSG00000169230 | 176732367 | rs6879874 | 5 | 176730775 | A | T | 0·256646 | -0·747919 | 0·0172373 | 0 | 0·236623 | 0·0359434 | 4·6041E-11 | -3·1608 | 0·485626 | 7·5796E-11 | 0·5687681 | 19 |
| *RAB24* | 5 | cg00848461 | 177000000 | *PRELID1* | 5 | ENSG00000169230 | 176732367 | rs6879874 | 5 | 176730775 | A | T | 0·256646 | -0·747919 | 0·0172373 | 0 | 0·236623 | 0·0359434 | 4·6041E-11 | -3·1608 | 0·485626 | 7·5796E-11 | 0·5687681 | 19 |
| *DNLZ* | 9 | cg00853853 | 139000000 | *PMPCA* | 9 | ENSG00000165688 | 139311661 | rs11145750 | 9 | 139259249 | G | A | 0·433538 | 0·048566 | 0·00847219 | 9·9019E-09 | -0·266869 | 0·0331815 | 8·7879E-16 | -0·181984 | 0·0389852 | 3·0408E-06 | 0·4104678 | 11 |
| *CISD1* | 10 | cg00871019 | 60028461 | *CISD1* | 10 | ENSG00000122873 | 60039082 | rs61366424 | 10 | 60004851 | A | T | 0·206544 | -0·794616 | 0·00917315 | 0 | 0·210045 | 0·0384758 | 4·7842E-08 | -3·78308 | 0·694354 | 5·0842E-08 | 0·08732663 | 14 |
| *SND1* | 7 | cg00893242 | 127000000 | *ARF5* | 7 | ENSG00000004059 | 127230079 | rs10229583 | 7 | 127246903 | A | G | 0·236196 | 0·0557208 | 0·00915948 | 1·1766E-09 | -0·421274 | 0·0377745 | 6·9754E-29 | -0·132267 | 0·0247667 | 9·2676E-08 | 0·150957 | 18 |
| *NSUN4* | 1 | cg00937489 | 46806552 | *NSUN4* | 1 | ENSG00000117481 | 46818336 | rs6678997 | 1 | 46825677 | G | A | 0·273006 | 0·630386 | 0·0090552 | 0 | -0·220814 | 0·0378705 | 5·5174E-09 | -2·85483 | 0·491329 | 6·2317E-09 | 0·04614982 | 4 |
| *CLYBL* | 13 | cg00988050 | 101000000 | *CLYBL* | 13 | ENSG00000125246 | 100404153 | rs8000435 | 13 | 100310215 | C | G | 0·108384 | -0·48704 | 0·0127476 | 0 | -0·375209 | 0·0510635 | 2·0135E-13 | 1·29805 | 0·179893 | 5·3673E-13 | 0·2100816 | 18 |
| *CHPT1* | 12 | cg01020980 | 102000000 | *CHPT1* | 12 | ENSG00000111666 | 102114321 | rs10778139 | 12 | 102083202 | A | G | 0·392638 | -0·638804 | 0·00724391 | 0 | 0·179571 | 0·0324588 | 3·161E-08 | -3·55739 | 0·644289 | 3·3627E-08 | 0·1645283 | 3 |
| *CYB5R3* | 22 | cg01031670 | 43014920 | *CYB5R3* | 22 | ENSG00000100243 | 43029710 | rs137100 | 22 | 42990074 | T | C | 0·226994 | -0·123703 | 0·00990353 | 8·3806E-36 | -0·211412 | 0·0351354 | 1·7761E-09 | 0·585128 | 0·10794 | 5·9306E-08 | 0·01756486 | 13 |
| *CYB5R3* | 22 | cg01031670 | 43014920 | *SERHL2* | 22 | ENSG00000183569 | 42960005 | rs137100 | 22 | 42990074 | T | C | 0·226994 | -0·254702 | 0·0169098 | 2·8621E-51 | -0·211412 | 0·0351354 | 1·7761E-09 | 1·20477 | 0·21561 | 2·3008E-08 | 0·0906407 | 18 |
| *TMEM70* | 8 | cg01041367 | 74888080 | *TMEM70* | 8 | ENSG00000175606 | 74889845 | rs4434614 | 8 | 74886732 | A | G | 0·151329 | -0·192526 | 0·0114332 | 1·2593E-63 | 0·500887 | 0·0463826 | 3·4788E-27 | -0·38437 | 0·0422834 | 9·8741E-20 | 0·05894839 | 20 |
| *NUDT8* | 11 | cg01069141 | 67398826 | *NUDT8* | 11 | ENSG00000167799 | 67396405 | rs1531514 | 11 | 67398862 | A | T | 0·439673 | 0·0355745 | 0·00804707 | 9·8336E-06 | -0·578056 | 0·0304655 | 2·7924E-80 | -0·0615416 | 0·0142938 | 1·6662E-05 | 0·01019749 | 5 |
| *MRPL41* | 9 | cg01111842 | 140000000 | *MRPL41* | 9 | ENSG00000182154 | 140446329 | rs2501556 | 9 | 140577178 | A | G | 0·552147 | -0·0595478 | 0·00795711 | 7·2314E-14 | -0·196339 | 0·0311299 | 2·8435E-10 | 0·303291 | 0·0628877 | 1·416E-06 | 0·01608143 | 3 |
| *ACSF3* | 16 | cg01134309 | 89190525 | *ACSF3* | 16 | ENSG00000176715 | 89188518 | rs55756754 | 16 | 89179363 | T | C | 0·0480573 | -0·118633 | 0·0207017 | 1·0008E-08 | -2·76462 | 0·0533134 | 0 | 0·0429111 | 0·00753367 | 1·2271E-08 | 0·0762158 | 20 |
| *HADHA* | 2 | cg01188578 | 26464058 | *HADHB* | 2 | ENSG00000138029 | 26489687 | rs12185726 | 2 | 26464059 | G | A | 0·291411 | 0·174067 | 0·0091084 | 2·061E-81 | 1·4306 | 0·0170402 | 0 | 0·121674 | 0·00652971 | 1·7053E-77 | 0·1304576 | 20 |
| *SLC25A46* | 5 | cg01227078 | 110000000 | *SLC25A46* | 5 | ENSG00000164209 | 110087347 | rs17446534 | 5 | 110079450 | C | T | 0·0920245 | -0·334945 | 0·0141652 | 1·31E-123 | -0·449556 | 0·0557216 | 7·1519E-16 | 0·745057 | 0·0975759 | 2·2466E-14 | 0·09057348 | 20 |
| *QRSL1* | 6 | cg01239717 | 107000000 | *QRSL1* | 6 | ENSG00000130348 | 107096872 | rs79735382 | 6 | 107023226 | A | G | 0·0511247 | 0·202681 | 0·0189065 | 8·1827E-27 | 0·434879 | 0·0725355 | 2·0299E-09 | 0·466063 | 0·089068 | 1·6708E-07 | 0·1087261 | 7 |
| *RTN4IP1* | 6 | cg01239717 | 107000000 | *QRSL1* | 6 | ENSG00000130348 | 107096872 | rs79735382 | 6 | 107023226 | A | G | 0·0511247 | 0·202681 | 0·0189065 | 8·1827E-27 | 0·434879 | 0·0725355 | 2·0299E-09 | 0·466063 | 0·089068 | 1·6708E-07 | 0·1087261 | 7 |
| *NDUFS6* | 5 | cg01294811 | 1806625 | *MRPL36* | 5 | ENSG00000171421 | 1799990 | rs11953620 | 5 | 1818630 | C | T | 0·364008 | 0·588166 | 0·0168424 | 3·478E-267 | 0·217124 | 0·0331614 | 5·8512E-11 | 2·70889 | 0·420939 | 1·2318E-10 | 0·150161 | 7 |
| *PNKD* | 2 | cg01329789 | 219000000 | *PNKD* | 2 | ENSG00000127838 | 219173315 | rs9751327 | 2 | 219179995 | G | A | 0·370143 | -0·474213 | 0·00784959 | 0 | -0·207125 | 0·0335779 | 6·8948E-10 | 2·2895 | 0·373091 | 8·4318E-10 | 0·05824822 | 11 |
| *MRPL4* | 19 | cg01409448 | 10362061 | *MRPL4* | 19 | ENSG00000105364 | 10366649 | rs8108218 | 19 | 10361065 | C | T | 0·299591 | -0·0927414 | 0·00970545 | 1·2287E-21 | 0·25734 | 0·0344253 | 7·7021E-14 | -0·360385 | 0·0612094 | 3·9152E-09 | 0·2362393 | 3 |
| *MRPL24* | 1 | cg01416295 | 157000000 | *MRPL24* | 1 | ENSG00000143314 | 156709238 | rs12036794 | 1 | 156706259 | T | C | 0·233129 | -0·167283 | 0·00948833 | 1·4395E-69 | 0·357253 | 0·0378757 | 4·0135E-21 | -0·468248 | 0·0563013 | 9·0355E-17 | 0·0205442 | 13 |
| *ACSF3* | 16 | cg01427313 | 89162196 | *ACSF3* | 16 | ENSG00000176715 | 89188518 | rs4302032 | 16 | 89212200 | A | G | 0·165644 | 0·115356 | 0·0105367 | 6·7931E-28 | -0·294969 | 0·0402718 | 2·3988E-13 | -0·391078 | 0·0642409 | 1·1455E-09 | 0·03981829 | 8 |
| *MRPS15* | 1 | cg01430725 | 36930006 | *MRPS15* | 1 | ENSG00000116898 | 36925678 | rs2884612 | 1 | 36895790 | T | C | 0·0899796 | 0·225736 | 0·0133079 | 1·5532E-64 | -0·682242 | 0·0530685 | 7·9709E-38 | -0·330874 | 0·0322938 | 1·2367E-24 | 0·2969826 | 17 |
| *ECSIT* | 19 | cg01436526 | 11641099 | *ECSIT* | 19 | ENSG00000130159 | 11628360 | rs113259217 | 19 | 11642086 | T | C | 0·0388548 | -0·212241 | 0·0231785 | 5·3459E-20 | -0·610663 | 0·0934782 | 6·4602E-11 | 0·347558 | 0·0653547 | 1·049E-07 | 0·9038587 | 14 |
| *SLC25A29* | 14 | cg01437482 | 101000000 | *SLC25A29* | 14 | ENSG00000197119 | 100765166 | rs10134767 | 14 | 100763954 | T | G | 0·365031 | 0·592597 | 0·00812026 | 0 | -0·221471 | 0·0343149 | 1·0888E-10 | -2·67573 | 0·416198 | 1·2846E-10 | 0·09853166 | 7 |
| *MTG1* | 10 | cg01444801 | 135000000 | *MTG1* | 10 | ENSG00000148824 | 135221204 | rs11101744 | 10 | 135226041 | C | G | 0·249489 | 0·352187 | 0·00939789 | 2·343E-301 | -0·708443 | 0·0378815 | 4·8087E-78 | -0·497128 | 0·0297084 | 7·4589E-63 | 0·02526359 | 20 |
| *MRPL34* | 19 | cg01445659 | 17414977 | *GTPBP3* | 19 | ENSG00000130299 | 17449636 | rs8100448 | 19 | 17422402 | A | C | 0·42638 | 0·133527 | 0·00930619 | 1·0934E-46 | 0·391815 | 0·0329103 | 1·1076E-32 | 0·340791 | 0·0371954 | 5·0863E-20 | 0·01886703 | 20 |
| *ACOT2* | 14 | cg01498900 | 74035742 | *ISCA2* | 14 | ENSG00000165898 | 74962116 | rs7155613 | 14 | 74087235 | C | A | 0·497955 | -0·037151 | 0·00817476 | 5·504E-06 | -0·493979 | 0·0323241 | 1·007E-52 | 0·0752077 | 0·017265 | 1·3242E-05 | 0·02978593 | 5 |
| *HSD17B8* | 6 | cg01502872 | 33173119 | *HSD17B8* | 6 | ENSG00000204228 | 33173513 | rs114171462 | 6 | 33083793 | A | T | 0·0439673 | -0·433588 | 0·029908 | 1·2583E-47 | 0·757623 | 0·0909627 | 8·1566E-17 | -0·5723 | 0·0792448 | 5·1254E-13 | 0·2744713 | 6 |
| *TIMM13* | 19 | cg01543300 | 2426874 | *TIMM13* | 19 | ENSG00000099800 | 2426757 | rs4806849 | 19 | 2447215 | A | C | 0·167689 | 0·182063 | 0·0110146 | 2·2612E-61 | -0·243812 | 0·0428492 | 1·2704E-08 | -0·746735 | 0·138794 | 7·4424E-08 | 0·2281253 | 7 |
| *HMGCL* | 1 | cg01604210 | 24129185 | *HMGCL* | 1 | ENSG00000117305 | 24146742 | rs2501370 | 1 | 24206053 | T | C | 0·407975 | 0·0862597 | 0·00827209 | 1·8512E-25 | -0·23389 | 0·0323006 | 4·4527E-13 | -0·368805 | 0·0620079 | 2·7193E-09 | 0·2020613 | 4 |
| *FAHD1* | 16 | cg01637482 | 1880436 | *HAGH* | 16 | ENSG00000063854 | 1861408 | rs238678 | 16 | 1872722 | A | G | 0·150307 | 0·359247 | 0·0117152 | 1·666E-206 | -0·313807 | 0·0496216 | 2·5488E-10 | -1·1448 | 0·184835 | 5·8783E-10 | 0·01283971 | 11 |
| *FAHD1* | 16 | cg01637482 | 1880436 | *NME3* | 16 | ENSG00000103024 | 1821009 | rs238678 | 16 | 1872722 | A | G | 0·150307 | 0·118142 | 0·0119205 | 3·7364E-23 | -0·313807 | 0·0496216 | 2·5488E-10 | -0·37648 | 0·070619 | 9·76E-08 | 0·1145383 | 11 |
| *BCKDHA* | 19 | cg01655341 | 41903697 | *DMAC2* | 19 | ENSG00000105341 | 41941922 | rs45500792 | 19 | 41903699 | G | T | 0·133947 | -0·154082 | 0·0127458 | 1·2104E-33 | 0·285343 | 0·0475114 | 1·9042E-09 | -0·539989 | 0·100396 | 7·5074E-08 | 0·01666421 | 4 |
| *NUDT13* | 10 | cg01701415 | 74870311 | *NUDT13* | 10 | ENSG00000166321 | 74880901 | rs12268338 | 10 | 75286806 | C | T | 0·0644172 | -0·499727 | 0·0145814 | 2·084E-257 | 0·384794 | 0·0644271 | 2·3357E-09 | -1·29869 | 0·22072 | 4·0078E-09 | 0·7115912 | 12 |
| *NUDT13* | 10 | cg01701415 | 74870311 | *MRPS16* | 10 | ENSG00000182180 | 75009480 | rs12268338 | 10 | 75286806 | C | T | 0·0644172 | 0·311375 | 0·0147476 | 5·965E-99 | 0·384794 | 0·0644271 | 2·3357E-09 | 0·809199 | 0·140803 | 9·0821E-09 | 0·4093001 | 12 |
| *PDK2* | 17 | cg01717201 | 48174262 | *PDK2* | 17 | ENSG00000005882 | 48180808 | rs8071734 | 17 | 48179321 | T | C | 0·308793 | 0·146018 | 0·010732 | 3·6991E-42 | 0·228229 | 0·0341177 | 2·24E-11 | 0·639787 | 0·106576 | 1·9356E-09 | 0·3048449 | 3 |
| *MRPS34* | 16 | cg01729862 | 1824041 | *MRPS34* | 16 | ENSG00000074071 | 1822523 | rs1178435 | 16 | 1830621 | T | C | 0·189162 | 0·111915 | 0·0102364 | 8·0154E-28 | -0·318856 | 0·0425857 | 7·0253E-14 | -0·350989 | 0·0568166 | 6·5092E-10 | 0·06994595 | 20 |
| *MRPS34* | 16 | cg01729862 | 1824041 | *NME3* | 16 | ENSG00000103024 | 1821009 | rs1178435 | 16 | 1830621 | T | C | 0·189162 | 0·18722 | 0·010229 | 7·8602E-75 | -0·318856 | 0·0425857 | 7·0253E-14 | -0·587162 | 0·0847281 | 4·2098E-12 | 0·01482415 | 20 |
| *SLC25A41* | 19 | cg01756647 | 6434432 | *SLC25A23* | 19 | ENSG00000125648 | 6450652 | rs2305058 | 19 | 6416123 | G | A | 0·50818 | 0·0726742 | 0·0118262 | 7·9863E-10 | -0·247719 | 0·0325465 | 2·7151E-14 | -0·293374 | 0·0613583 | 1·7415E-06 | 0·4609887 | 8 |
| *TK2* | 16 | cg01758899 | 66583237 | *TK2* | 16 | ENSG00000166548 | 66564176 | rs61066285 | 16 | 66631542 | G | A | 0·137014 | 0·705711 | 0·0116947 | 0 | -0·441203 | 0·0507351 | 3·4315E-18 | -1·59952 | 0·185833 | 7·4805E-18 | 0·08849502 | 20 |
| *ALDH7A1* | 5 | cg01814098 | 126000000 | *ALDH7A1* | 5 | ENSG00000164904 | 125904321 | rs62391691 | 5 | 126078913 | A | G | 0·0552147 | -0·27652 | 0·0264147 | 1·2067E-25 | 0·599112 | 0·0723322 | 1·2033E-16 | -0·46155 | 0·0710569 | 8·2759E-11 | 0·03661845 | 5 |
| *CAT* | 11 | cg01847719 | 34460557 | *CAT* | 11 | ENSG00000121691 | 34477040 | rs11604331 | 11 | 34460769 | G | A | 0·351738 | 0·678855 | 0·00748166 | 0 | -0·189372 | 0·0337266 | 1·9666E-08 | -3·58477 | 0·639659 | 2·0923E-08 | 0·3592719 | 4 |
| *CYP27A1* | 2 | cg01872077 | 220000000 | *CYP27A1* | 2 | ENSG00000135929 | 219663244 | rs691140 | 2 | 219627004 | G | A | 0·42638 | -0·766623 | 0·0073476 | 0 | 0·20947 | 0·0322264 | 8·0348E-11 | -3·65982 | 0·564146 | 8·7347E-11 | 0·1010344 | 12 |
| *TK2* | 16 | cg01931893 | 66584178 | *TK2* | 16 | ENSG00000166548 | 66564176 | rs725131 | 16 | 66585710 | G | C | 0·134969 | 0·712605 | 0·0115019 | 0 | -0·487863 | 0·0498778 | 1·3565E-22 | -1·46067 | 0·151184 | 4·394E-22 | 0·06750765 | 20 |
| *SERHL2* | 22 | cg02051290 | 42967981 | *BIK* | 22 | ENSG00000100290 | 43516236 | rs2071729 | 22 | 43567789 | C | T | 0·0306748 | 0·106268 | 0·0167626 | 2·3036E-10 | -0·590711 | 0·0960398 | 7·7148E-10 | -0·179898 | 0·040752 | 1·0126E-05 | 0·348995 | 3 |
| *AGPAT4* | 6 | cg02054200 | 162000000 | *AGPAT4* | 6 | ENSG00000026652 | 161623052 | rs150720261 | 6 | 161565967 | T | C | 0·0623722 | 0·192005 | 0·0163153 | 5·6792E-32 | -0·34632 | 0·0634489 | 4·8084E-08 | -0·554415 | 0·111967 | 7·3609E-07 | 0·271468 | 3 |
| *CAT* | 11 | cg02109652 | 34460386 | *CAT* | 11 | ENSG00000121691 | 34477040 | rs12793666 | 11 | 34444031 | A | C | 0·349693 | 0·680619 | 0·00746814 | 0 | -0·205797 | 0·0335118 | 8·1984E-10 | -3·30723 | 0·539768 | 8·947E-10 | 0·9397033 | 10 |
| *METTL8* | 2 | cg02115781 | 172000000 | *METTL8* | 2 | ENSG00000123600 | 172235536 | rs34096402 | 2 | 172151436 | C | G | 0·273006 | -0·25037 | 0·00891435 | 1·445E-173 | -0·21825 | 0·0369394 | 3·456E-09 | 1·14717 | 0·198411 | 7·3914E-09 | 0·1596164 | 8 |
| *BCO2* | 11 | cg02119229 | 112000000 | *BCO2* | 11 | ENSG00000197580 | 112070806 | rs11214124 | 11 | 112061361 | T | C | 0·331288 | 0·133835 | 0·00841655 | 6·1963E-57 | 0·371104 | 0·0340336 | 1·1024E-27 | 0·36064 | 0·0401031 | 2·4096E-19 | 0·04120703 | 20 |
| *MSRA* | 8 | cg02131230 | 10283275 | *MSRA* | 8 | ENSG00000175806 | 10099089 | rs9657519 | 8 | 10945439 | A | G | 0·489775 | -0·19536 | 0·00789563 | 3·706E-135 | 0·188206 | 0·0308227 | 1·021E-09 | -1·03801 | 0·175096 | 3·0621E-09 | 0·5009485 | 20 |
| *AURKAIP1* | 1 | cg02144516 | 1311909 | *PUSL1* | 1 | ENSG00000169972 | 1245502 | rs78446752 | 1 | 1183397 | T | C | 0·0654397 | -0·211083 | 0·0213431 | 4·6003E-23 | 0·426657 | 0·0599257 | 1·0811E-12 | -0·494737 | 0·085621 | 7·5495E-09 | 0·07175135 | 20 |
| *AURKAIP1* | 1 | cg02144516 | 1311909 | *MRPL20* | 1 | ENSG00000242485 | 1339990 | rs78446752 | 1 | 1183397 | T | C | 0·0654397 | 0·285143 | 0·0215766 | 7·1505E-40 | 0·426657 | 0·0599257 | 1·0811E-12 | 0·668319 | 0·106624 | 3·6572E-10 | 0·1313663 | 20 |
| *VARS2* | 6 | cg02149965 | 30883203 | *C6orf136* | 6 | ENSG00000204564 | 30617901 | rs1265093 | 6 | 31107187 | A | G | 0·276074 | 0·0525938 | 0·0092647 | 1·3724E-08 | -0·353036 | 0·0358378 | 6·79E-23 | -0·148976 | 0·0302885 | 8·7187E-07 | 0·01153812 | 3 |
| *HTRA2* | 2 | cg02154074 | 74756234 | *MTHFD2* | 2 | ENSG00000065911 | 74435190 | rs10185658 | 2 | 74542691 | G | C | 0·140082 | -0·0581902 | 0·0115525 | 4·7288E-07 | -0·586038 | 0·0468878 | 7·5844E-36 | 0·0992942 | 0·0212536 | 2·9844E-06 | 0·01939758 | 7 |
| *PDF* | 16 | cg02192472 | 69364945 | *DUS2* | 16 | ENSG00000167264 | 68067436 | rs7202398 | 16 | 69006797 | G | A | 0·183027 | 0·0764659 | 0·0119928 | 1·8177E-10 | 0·324346 | 0·0418837 | 9·6361E-15 | 0·235754 | 0·0478955 | 8·555E-07 | 0·4883334 | 6 |
| *PDF* | 16 | cg02192472 | 69364945 | *PDF* | 16 | ENSG00000258429 | 69363511 | rs2062547 | 16 | 69374461 | C | G | 0·284254 | -0·185424 | 0·00990502 | 3·3888E-78 | 0·696542 | 0·032603 | 2·866E-101 | -0·266206 | 0·018907 | 5·0572E-45 | 0·01085537 | 20 |
| *ACSF3* | 16 | cg02193283 | 89164953 | *ACSF3* | 16 | ENSG00000176715 | 89188518 | rs12917851 | 16 | 89208407 | A | T | 0·134969 | 0·0770714 | 0·0125018 | 7·0572E-10 | 0·492912 | 0·0461539 | 1·266E-26 | 0·156359 | 0·0292856 | 9·3396E-08 | 0·02663128 | 20 |
| *SPG7* | 16 | cg02244288 | 89573955 | *SPG7* | 16 | ENSG00000197912 | 89590750 | rs3935312 | 16 | 89592077 | A | G | 0·489775 | 0·188414 | 0·00791742 | 3·553E-125 | -0·59971 | 0·0321626 | 1·3558E-77 | -0·314175 | 0·0214055 | 9·005E-49 | 0·05372809 | 20 |
| *SPG7* | 16 | cg02244288 | 89573955 | *LOC101930112* | 16 | ENSG00000197912 | 89590750 | rs3935312 | 16 | 89592077 | A | G | 0·489775 | 0·188414 | 0·00791742 | 3·553E-125 | -0·59971 | 0·0321626 | 1·3558E-77 | -0·314175 | 0·0214055 | 9·005E-49 | 0·05372809 | 20 |
| *NUDT19* | 19 | cg02283691 | 33182526 | *NUDT19* | 19 | ENSG00000213965 | 33193784 | rs8108621 | 19 | 33183291 | A | G | 0·175869 | -0·0935096 | 0·0118604 | 3·1656E-15 | -0·403378 | 0·0423911 | 1·8062E-21 | 0·231816 | 0·0381838 | 1·2707E-09 | 0·1382889 | 11 |
| *TK2* | 16 | cg02295216 | 66585210 | *DUS2* | 16 | ENSG00000167264 | 68067436 | rs189527310 | 16 | 67447829 | A | G | 0·0143149 | 0·522308 | 0·0474748 | 3·7458E-28 | -0·616948 | 0·110969 | 2·7031E-08 | -0·8466 | 0·170615 | 6·9749E-07 | 0·4512175 | 16 |
| *OXCT1* | 5 | cg02389942 | 41869679 | *OXCT1* | 5 | ENSG00000083720 | 41800394 | rs11750607 | 5 | 41889050 | A | C | 0·239264 | 0·132736 | 0·0098599 | 2·6103E-41 | -0·269407 | 0·0413976 | 7·6261E-11 | -0·492697 | 0·0840908 | 4·6536E-09 | 0·1512402 | 20 |
| *MRPL34* | 19 | cg02403349 | 17416356 | *MRPL34* | 19 | ENSG00000130312 | 17410535 | rs8100232 | 19 | 17422951 | A | G | 0·42638 | 0·50404 | 0·00876183 | 0 | 0·780473 | 0·0288961 | 1·139E-160 | 0·645814 | 0·0264148 | 5·166E-132 | 0·288684 | 20 |
| *ACSF3* | 16 | cg02441474 | 89165300 | *ACSF3* | 16 | ENSG00000176715 | 89188518 | rs2287353 | 16 | 89262431 | A | G | 0·091002 | 0·146597 | 0·0128495 | 3·7791E-30 | -0·3822 | 0·0489354 | 5·7053E-15 | -0·383561 | 0·0595151 | 1·1578E-10 | 0·01771406 | 20 |
| *NSUN4* | 1 | cg02459555 | 46806155 | *NSUN4* | 1 | ENSG00000117481 | 46818336 | rs12137934 | 1 | 46728913 | G | A | 0·282209 | 0·616143 | 0·00816897 | 0 | 0·273732 | 0·0363858 | 5·3515E-14 | 2·2509 | 0·300685 | 7·1058E-14 | 0·02874914 | 14 |
| *SLC25A34* | 1 | cg02485345 | 16061554 | *AGMAT* | 1 | ENSG00000116771 | 15905226 | rs6701500 | 1 | 16063698 | T | G | 0·267894 | 0·0511945 | 0·00866939 | 3·5222E-09 | 0·285871 | 0·0359572 | 1·8604E-15 | 0·179083 | 0·0377765 | 2·1311E-06 | 0·3867272 | 3 |
| *SLC25A34* | 1 | cg02485345 | 16061554 | *SLC25A34* | 1 | ENSG00000162461 | 16065395 | rs35352784 | 1 | 16061966 | A | G | 0·207566 | -0·49595 | 0·00920702 | 0 | 0·340638 | 0·0388033 | 1·6554E-18 | -1·45594 | 0·16804 | 4·5435E-18 | 0·6050841 | 17 |
| *GRPEL1* | 4 | cg02503808 | 7069936 | *GRPEL1* | 4 | ENSG00000109519 | 7065278 | rs3796908 | 4 | 7061378 | G | A | 0·220859 | -0·0958309 | 0·0100471 | 1·4533E-21 | -0·611581 | 0·0366837 | 2·1031E-62 | 0·156694 | 0·0189266 | 1·2419E-16 | 0·01041565 | 20 |
| *GPT2* | 16 | cg02506360 | 46963989 | *GPT2* | 16 | ENSG00000166123 | 46941749 | rs11540355 | 16 | 46964022 | G | A | 0·0480573 | 0·101143 | 0·0203172 | 6·4178E-07 | 0·683706 | 0·0715544 | 1·2353E-21 | 0·147933 | 0·0335075 | 1·0104E-05 | 0·06452878 | 9 |
| *BDH1* | 3 | cg02569554 | 197000000 | *BDH1* | 3 | ENSG00000161267 | 197268424 | rs71325620 | 3 | 197273992 | T | C | 0·291411 | 0·1574 | 0·00963835 | 5·9793E-60 | -0·242108 | 0·0360421 | 1·8503E-11 | -0·650123 | 0·10465 | 5·22E-10 | 0·0141041 | 13 |
| *VARS2* | 6 | cg02601318 | 30882384 | *VARS2* | 6 | ENSG00000137411 | 30885127 | rs2596495 | 6 | 31323416 | C | G | 0·0940695 | -0·283682 | 0·0191682 | 1·4736E-49 | 0·33604 | 0·0440886 | 2·4993E-14 | -0·844191 | 0·124584 | 1·2347E-11 | 0·1246991 | 20 |
| *MOCS1* | 6 | cg02680909 | 39899321 | *MOCS1* | 6 | ENSG00000124615 | 39884822 | rs2504116 | 6 | 39899372 | C | T | 0·43456 | -0·0390214 | 0·00811458 | 1·5184E-06 | -0·331509 | 0·0329 | 7·0354E-24 | 0·117708 | 0·0271224 | 1·4255E-05 | 0·03278454 | 10 |
| *THNSL1* | 10 | cg02690159 | 25305391 | *THNSL1* | 10 | ENSG00000185875 | 25310590 | rs4748996 | 10 | 25311890 | C | T | 0·283231 | -0·132106 | 0·0087086 | 5·6217E-52 | 0·709629 | 0·0329571 | 7·829E-103 | -0·186162 | 0·0150118 | 2·5795E-35 | 0·01542055 | 20 |
| *IMMP2L* | 7 | cg02729030 | 111000000 | *IMMP2L* | 7 | ENSG00000184903 | 110752841 | rs6466376 | 7 | 110959881 | T | C | 0·505112 | 0·518879 | 0·00808372 | 0 | 0·269806 | 0·0318253 | 2·2964E-17 | 1·92316 | 0·228818 | 4·2883E-17 | 0·1886166 | 20 |
| *PDF* | 16 | cg02802420 | 69363236 | *PDF* | 16 | ENSG00000258429 | 69363511 | rs1424114 | 16 | 69224615 | T | C | 0·340491 | -0·14998 | 0·00965508 | 2·0488E-54 | 0·219978 | 0·0340873 | 1·0939E-10 | -0·681795 | 0·114404 | 2·5293E-09 | 0·01518132 | 8 |
| *SLC25A30* | 13 | cg02828514 | 45993412 | *SLC25A30* | 13 | ENSG00000174032 | 45980020 | rs9316140 | 13 | 46006171 | A | G | 0·47955 | -0·0829338 | 0·00867998 | 1·2406E-21 | -0·182701 | 0·0316657 | 7·9424E-09 | 0·453932 | 0·0919073 | 7·8517E-07 | 0·1325943 | 10 |
| *OGG1* | 3 | cg02860797 | 9791144 | *RPUSD3* | 3 | ENSG00000156990 | 9882909 | rs3219008 | 3 | 9795543 | G | A | 0·188139 | -0·0624752 | 0·00987875 | 2·5455E-10 | 0·167837 | 0·024691 | 1·0645E-11 | -0·372237 | 0·0803938 | 3·6536E-06 | 0·02693115 | 5 |
| *PITRM1* | 10 | cg02899903 | 3216063 | *PITRM1* | 10 | ENSG00000107959 | 3197461 | rs7098000 | 10 | 3214739 | A | G | 0·248466 | 0·55981 | 0·00855222 | 0 | -0·197916 | 0·0356007 | 2·7081E-08 | -2·82852 | 0·51062 | 3·0353E-08 | 0·1593049 | 10 |
| *HTATIP2* | 11 | cg02900213 | 20385756 | *HTATIP2* | 11 | ENSG00000109854 | 20395280 | rs10437608 | 11 | 20385606 | G | A | 0·464213 | -0·46468 | 0·00756487 | 0 | 0·507424 | 0·0317256 | 1·404E-57 | -0·915763 | 0·0591653 | 4·8805E-54 | 0·02594254 | 20 |
| *MCL1* | 1 | cg02961109 | 151000000 | *MCL1* | 1 | ENSG00000143384 | 150549549 | rs6587756 | 1 | 150546865 | A | T | 0·347648 | 0·09329 | 0·00871315 | 9·4574E-27 | 0·218117 | 0·0345848 | 2·85E-10 | 0·427706 | 0·0787082 | 5·5082E-08 | 0·03404054 | 10 |
| *MCL1* | 1 | cg02961109 | 151000000 | *ADAMTSL4-AS1* | 1 | ENSG00000143384 | 150549549 | rs6587756 | 1 | 150546865 | A | T | 0·347648 | 0·09329 | 0·00871315 | 9·4574E-27 | 0·218117 | 0·0345848 | 2·85E-10 | 0·427706 | 0·0787082 | 5·5082E-08 | 0·03404054 | 10 |
| *TRIT1* | 1 | cg02965157 | 40350001 | *TRIT1* | 1 | ENSG00000043514 | 40327945 | rs114129095 | 1 | 40300843 | A | T | 0·0449898 | 0·591878 | 0·0172995 | 1·518E-256 | 0·447557 | 0·0793825 | 1·7206E-08 | 1·32246 | 0·237727 | 2·6525E-08 | 0·8016888 | 8 |
| *COX16* | 14 | cg03055888 | 70826472 | *COX16* | 14 | ENSG00000133983 | 70809123 | rs17566814 | 14 | 70837256 | A | C | 0·106339 | -0·151598 | 0·014198 | 1·2986E-26 | 0·43588 | 0·0523509 | 8·3528E-17 | -0·347798 | 0·0529708 | 5·174E-11 | 0·02515517 | 14 |
| *COX16* | 14 | cg03055888 | 70826472 | *SYNJ2BP* | 14 | ENSG00000213463 | 70860963 | rs11622440 | 14 | 70815162 | A | C | 0·158487 | -0·0644748 | 0·0105863 | 1·1263E-09 | 0·279185 | 0·0424309 | 4·7123E-11 | -0·230939 | 0·0516694 | 7·8381E-06 | 0·4795615 | 5 |
| *NME3* | 16 | cg03080965 | 1821577 | *MRPS34* | 16 | ENSG00000074071 | 1822523 | rs2575329 | 16 | 1819433 | G | A | 0·428425 | 0·137722 | 0·00807744 | 3·4833E-65 | -0·215159 | 0·0324244 | 3·2293E-11 | -0·640094 | 0·10351 | 6·2539E-10 | 0·3316466 | 20 |
| *NME3* | 16 | cg03080965 | 1821577 | *NME3* | 16 | ENSG00000103024 | 1821009 | rs2575329 | 16 | 1819433 | G | A | 0·428425 | 0·168599 | 0·0080807 | 1·128E-96 | -0·215159 | 0·0324244 | 3·2293E-11 | -0·783602 | 0·123917 | 2·5553E-10 | 0·01358684 | 20 |
| *LDHD* | 16 | cg03085549 | 75150819 | *LDHD* | 16 | ENSG00000166816 | 75148213 | rs7193344 | 16 | 75191702 | G | T | 0·370143 | -0·0867271 | 0·00825202 | 7·7858E-26 | 0·196429 | 0·0334085 | 4·1121E-09 | -0·441519 | 0·0860455 | 2·8786E-07 | 0·7650812 | 14 |
| *CMC1* | 3 | cg03144753 | 28286464 | *CMC1* | 3 | ENSG00000187118 | 28324854 | rs10510607 | 3 | 28286261 | T | C | 0·172802 | 0·295664 | 0·0112849 | 2·66E-151 | -0·232928 | 0·0415098 | 2·0071E-08 | -1·26934 | 0·231337 | 4·0892E-08 | 0·1315476 | 3 |
| *MRPL23* | 11 | cg03157283 | 1975380 | *MRPL23* | 11 | ENSG00000214026 | 1987130 | rs75676658 | 11 | 1988737 | G | A | 0·0235174 | -0·217599 | 0·0330808 | 4·7746E-11 | 0·866901 | 0·0902689 | 7·7241E-22 | -0·251008 | 0·0462528 | 5·7349E-08 | 0·01653461 | 11 |
| *MRPL23* | 11 | cg03157283 | 1975380 | *LOC107987373* | 11 | ENSG00000214026 | 1987130 | rs75676658 | 11 | 1988737 | G | A | 0·0235174 | -0·217599 | 0·0330808 | 4·7746E-11 | 0·866901 | 0·0902689 | 7·7241E-22 | -0·251008 | 0·0462528 | 5·7349E-08 | 0·01653461 | 11 |
| *MUL1* | 1 | cg03182908 | 20833814 | *MUL1* | 1 | ENSG00000090432 | 20830298 | rs6700034 | 1 | 20834610 | A | C | 0·119632 | 0·635123 | 0·0118342 | 0 | 0·571549 | 0·0487929 | 1·0835E-31 | 1·11123 | 0·0970987 | 2·5098E-30 | 0·03450358 | 14 |
| *NDUFA13* | 19 | cg03233793 | 19626605 | *NDUFA13* | 19 | ENSG00000186010 | 19635415 | rs2965201 | 19 | 19478051 | C | T | 0·171779 | 0·187679 | 0·0128519 | 2·6797E-48 | -0·392335 | 0·0423631 | 2·0205E-20 | -0·478364 | 0·0611637 | 5·2388E-15 | 0·3299631 | 20 |
| *DNAJC4* | 11 | cg03236948 | 63997492 | *DNAJC4* | 11 | ENSG00000110011 | 63999754 | rs1860368 | 11 | 63994692 | T | C | 0·311861 | -0·0551624 | 0·00912831 | 1·5127E-09 | 0·410233 | 0·0329853 | 1·6492E-35 | -0·134466 | 0·0247392 | 5·4691E-08 | 0·1366237 | 10 |
| *ALDH1B1* | 9 | cg03237166 | 38393112 | *ALDH1B1* | 9 | ENSG00000137124 | 38395659 | rs4646770 | 9 | 38393171 | G | A | 0·302658 | -0·0581441 | 0·00992051 | 4·6009E-09 | 0·371445 | 0·0371859 | 1·705E-23 | -0·156535 | 0·0309659 | 4·3024E-07 | 0·01015031 | 3 |
| *NDUFS6* | 5 | cg03243450 | 1800782 | *MRPL36* | 5 | ENSG00000171421 | 1799990 | rs2270826 | 5 | 1799737 | C | T | 0·368098 | 0·609088 | 0·0281649 | 1·027E-103 | -0·237247 | 0·0326766 | 3·8577E-13 | -2·56732 | 0·372999 | 5·8642E-12 | 0·02941302 | 16 |
| *MRPL36* | 5 | cg03243450 | 1800782 | *MRPL36* | 5 | ENSG00000171421 | 1799990 | rs2270826 | 5 | 1799737 | C | T | 0·368098 | 0·609088 | 0·0281649 | 1·027E-103 | -0·237247 | 0·0326766 | 3·8577E-13 | -2·56732 | 0·372999 | 5·8642E-12 | 0·02941302 | 16 |
| *MRPL12* | 17 | cg03243595 | 79673106 | *OXLD1* | 17 | ENSG00000204237 | 79632865 | rs72860537 | 17 | 79667304 | T | C | 0·0705521 | -0·768919 | 0·0193391 | 0 | -0·557897 | 0·0706153 | 2·7775E-15 | 1·37825 | 0·177861 | 9·2609E-15 | 0·5499226 | 12 |
| *FBXL4* | 6 | cg03306368 | 99396091 | *FBXL4* | 6 | ENSG00000112234 | 99358591 | rs35727107 | 6 | 99301012 | T | C | 0·126789 | 0·118087 | 0·01272 | 1·6385E-20 | 0·346478 | 0·0501549 | 4·91E-12 | 0·340821 | 0·0614966 | 2·9884E-08 | 0·1947065 | 5 |
| *TXNRD1* | 12 | cg03331715 | 105000000 | *TXNRD1* | 12 | ENSG00000198431 | 104676809 | rs10861185 | 12 | 104676946 | A | C | 0·440695 | 0·186705 | 0·00801123 | 3·908E-120 | 0·276424 | 0·032335 | 1·2442E-17 | 0·67543 | 0·0841569 | 1·0083E-15 | 0·04068087 | 20 |
| *SFXN5* | 2 | cg03344820 | 73297746 | *SFXN5* | 2 | ENSG00000144040 | 73235956 | rs56088054 | 2 | 73297743 | A | C | 0·0572597 | -0·293197 | 0·0161844 | 2·383E-73 | 0·356438 | 0·0644144 | 3·1388E-08 | -0·822575 | 0·155433 | 1·2089E-07 | 0·138937 | 3 |
| *NDUFC2* | 11 | cg03353394 | 77791298 | *NDUFC2* | 11 | ENSG00000151366 | 77785307 | rs7125563 | 11 | 77829010 | A | G | 0·275051 | 0·153723 | 0·00884686 | 1·254E-67 | 0·222216 | 0·0348903 | 1·9029E-10 | 0·691773 | 0·115682 | 2·2323E-09 | 0·265227 | 10 |
| *PRODH* | 22 | cg03407228 | 18921705 | *SLC25A1* | 22 | ENSG00000100075 | 19164719 | rs2008903 | 22 | 18910307 | G | A | 0·440695 | -0·0502606 | 0·00905825 | 2·8797E-08 | -0·360877 | 0·0327973 | 3·6854E-28 | 0·139273 | 0·0281115 | 7·258E-07 | 0·0204357 | 11 |
| *ALDH7A1* | 5 | cg03409151 | 126000000 | *ALDH7A1* | 5 | ENSG00000164904 | 125904321 | rs7719712 | 5 | 125910169 | C | T | 0·0940695 | 0·468218 | 0·0188712 | 6·787E-136 | -1·02977 | 0·0544159 | 7·2266E-80 | -0·454682 | 0·0302177 | 3·6199E-51 | 0·01436272 | 20 |
| *IFI27* | 14 | cg03447547 | 94577039 | *IFI27* | 14 | ENSG00000165949 | 94577107 | rs2896258 | 14 | 94564035 | A | G | 0·488753 | -0·0865421 | 0·00827932 | 1·4226E-25 | 0·203844 | 0·0317786 | 1·4129E-10 | -0·424551 | 0·0776547 | 4·5731E-08 | 0·09438699 | 11 |
| *TK2* | 16 | cg03448915 | 66583078 | *TK2* | 16 | ENSG00000166548 | 66564176 | rs2290183 | 16 | 66613841 | C | G | 0·132924 | 0·719804 | 0·0116359 | 0 | -1·35417 | 0·042724 | 1·777E-220 | -0·531546 | 0·0188434 | 4·592E-175 | 0·01159313 | 20 |
| *CPOX* | 3 | cg03483464 | 98312971 | *CPOX* | 3 | ENSG00000080819 | 98276271 | rs1381090 | 3 | 98319863 | T | A | 0·294479 | -0·172937 | 0·00936161 | 3·4062E-76 | 0·288891 | 0·0361555 | 1·3468E-15 | -0·598624 | 0·0816274 | 2·2402E-13 | 0·4396578 | 18 |
| *UQCRC1* | 3 | cg03536664 | 48636487 | *UQCRC1* | 3 | ENSG00000010256 | 48642422 | rs138792863 | 3 | 49455678 | T | C | 0·0541922 | 0·344849 | 0·0299634 | 1·1885E-30 | -0·654241 | 0·0729963 | 3·1695E-19 | -0·527098 | 0·0745399 | 1·5343E-12 | 0·6319711 | 13 |
| *COX4I1* | 16 | cg03563694 | 85832315 | *COX4I1* | 16 | ENSG00000131143 | 85836444 | rs301152 | 16 | 85805894 | G | C | 0·0480573 | 0·302446 | 0·0174012 | 1·1533E-67 | -0·489141 | 0·0727677 | 1·7931E-11 | -0·618321 | 0·0986249 | 3·624E-10 | 0·01691649 | 7 |
| *GRPEL1* | 4 | cg03567896 | 7067375 | *GRPEL1* | 4 | ENSG00000109519 | 7065278 | rs4689589 | 4 | 7077428 | T | C | 0·0388548 | -0·137808 | 0·0204069 | 1·4482E-11 | 1·0351 | 0·0883681 | 1·0869E-31 | -0·133135 | 0·0227566 | 4·9043E-09 | 0·03338079 | 3 |
| *PDF* | 16 | cg03603530 | 69365009 | *DUS2* | 16 | ENSG00000167264 | 68067436 | rs78235713 | 16 | 68966406 | G | A | 0·179959 | 0·0774131 | 0·0121326 | 1·764E-10 | 0·342965 | 0·0423713 | 5·7607E-16 | 0·225717 | 0·0450451 | 5·4172E-07 | 0·04586666 | 11 |
| *SCP2* | 1 | cg03609269 | 53393023 | *SCP2* | 1 | ENSG00000116171 | 53455138 | rs11585570 | 1 | 53438130 | C | T | 0·364008 | -0·68845 | 0·0074077 | 0 | 0·220042 | 0·0326256 | 1·5361E-11 | -3·12872 | 0·465116 | 1·7348E-11 | 0·3910822 | 19 |
| *SCP2* | 1 | cg03609269 | 53393023 | *ECHDC2* | 1 | ENSG00000121310 | 53377270 | rs11585570 | 1 | 53438130 | C | T | 0·364008 | -0·538773 | 0·00778918 | 0 | 0·220042 | 0·0326256 | 1·5361E-11 | -2·4485 | 0·364761 | 1·9117E-11 | 0·04083812 | 19 |
| *PPIF* | 10 | cg03648155 | 81106771 | *PPIF* | 10 | ENSG00000108179 | 81111159 | rs2105461 | 10 | 81101679 | T | C | 0·531697 | -0·134937 | 0·00796606 | 2·3204E-64 | 0·189286 | 0·0334333 | 1·4996E-08 | -0·712874 | 0·132761 | 7·8903E-08 | 0·0491774 | 8 |
| *NDUFA9* | 12 | cg03680150 | 4758201 | *NDUFA9* | 12 | ENSG00000139180 | 4756282 | rs714621 | 12 | 4758188 | G | C | 0·445808 | 0·150109 | 0·0118246 | 6·3354E-37 | 0·322669 | 0·0312105 | 4·7205E-25 | 0·46521 | 0·0580325 | 1·0891E-15 | 0·3128275 | 16 |
| *LDHD* | 16 | cg03743982 | 75150833 | *LDHD* | 16 | ENSG00000166816 | 75148213 | rs9927029 | 16 | 75172340 | A | G | 0·406953 | -0·0907657 | 0·0080566 | 1·9315E-29 | 0·202982 | 0·0332271 | 1·0031E-09 | -0·447161 | 0·0832667 | 7·8637E-08 | 0·6869732 | 14 |
| *CBR4* | 4 | cg03845745 | 170000000 | *CBR4* | 4 | ENSG00000145439 | 169858173 | rs6839909 | 4 | 169850155 | C | A | 0·124744 | 0·569007 | 0·0123041 | 0 | -0·291071 | 0·0498159 | 5·129E-09 | -1·95487 | 0·337231 | 6·7574E-09 | 0·2312254 | 4 |
| *GRPEL1* | 4 | cg03847636 | 7070632 | *GRPEL1* | 4 | ENSG00000109519 | 7065278 | rs11945642 | 4 | 7051065 | C | G | 0·197342 | -0·104819 | 0·0103621 | 4·7112E-24 | 0·239185 | 0·0393467 | 1·21E-09 | -0·438234 | 0·0841067 | 1·8838E-07 | 0·7577259 | 8 |
| *NIPSNAP1* | 22 | cg03857553 | 29976613 | *NIPSNAP1* | 22 | ENSG00000184117 | 29964061 | rs71640299 | 22 | 29977229 | A | G | 0·0439673 | -0·407942 | 0·0182355 | 7·577E-111 | 0·465818 | 0·076397 | 1·0785E-09 | -0·875754 | 0·148868 | 4·0352E-09 | 0·2888299 | 5 |
| *PPTC7* | 12 | cg03926050 | 111000000 | *PPTC7* | 12 | ENSG00000196850 | 110995122 | rs28562888 | 12 | 110818442 | T | C | 0·110429 | -0·224587 | 0·0159544 | 5·2744E-45 | -0·372248 | 0·0555334 | 2·0399E-11 | 0·603326 | 0·0996901 | 1·4304E-09 | 0·2021851 | 20 |
| *HDHD3* | 9 | cg03984733 | 116000000 | *HDHD3* | 9 | ENSG00000119431 | 116137489 | rs3747815 | 9 | 116137673 | C | A | 0·246421 | -0·499805 | 0·00884892 | 0 | -0·215718 | 0·0367647 | 4·4233E-09 | 2·31694 | 0·396999 | 5·3429E-09 | 0·2004687 | 6 |
| *LDHD* | 16 | cg03991512 | 75150456 | *LDHD* | 16 | ENSG00000166816 | 75148213 | rs9927029 | 16 | 75172340 | A | G | 0·406953 | -0·0907657 | 0·0080566 | 1·9315E-29 | 0·231046 | 0·0334181 | 4·7182E-12 | -0·392847 | 0·0666672 | 3·8003E-09 | 0·4055906 | 16 |
| *POLG2* | 17 | cg04009666 | 62493127 | *POLG2* | 17 | ENSG00000256525 | 62483528 | rs9902065 | 17 | 62632349 | T | C | 0·0889571 | -0·119318 | 0·022275 | 8·4803E-08 | 0·923629 | 0·0667841 | 1·6783E-43 | -0·129184 | 0·0258625 | 5·8828E-07 | 0·5561572 | 5 |
| *GATM* | 15 | cg04025675 | 45671028 | *GATM* | 15 | ENSG00000171766 | 45673869 | rs12593371 | 15 | 45640084 | A | G | 0·283231 | -0·570475 | 0·00829354 | 0 | 0·683809 | 0·0346216 | 7·8779E-87 | -0·834261 | 0·0439459 | 2·3209E-80 | 0·02928795 | 20 |
| *ACSF3* | 16 | cg04069539 | 89168963 | *ACSF3* | 16 | ENSG00000176715 | 89188518 | rs9939105 | 16 | 89132772 | G | C | 0·388548 | -0·11916 | 0·0176706 | 1·5472E-11 | 0·249428 | 0·0338335 | 1·6782E-13 | -0·477733 | 0·0960116 | 6·4984E-07 | 0·07686431 | 20 |
| *NAGS* | 17 | cg04196345 | 42084953 | *NAGS* | 17 | ENSG00000161653 | 42084172 | rs228771 | 17 | 42084097 | G | C | 0·433538 | 0·0544441 | 0·00832912 | 6·2933E-11 | 0·328511 | 0·0335311 | 1·1575E-22 | 0·16573 | 0·0304793 | 5·4044E-08 | 0·06652962 | 14 |
| *NIPSNAP3B* | 9 | cg04239375 | 108000000 | *NIPSNAP3B* | 9 | ENSG00000165028 | 107533088 | rs62565988 | 9 | 107526619 | A | G | 0·128834 | 0·319896 | 0·012386 | 4·389E-147 | -0·558563 | 0·0485396 | 1·2112E-30 | -0·572712 | 0·0544857 | 7·6678E-26 | 0·1289313 | 14 |
| *TOP3A* | 17 | cg04261877 | 18218829 | *ATPAF2* | 17 | ENSG00000171953 | 17911623 | rs1563632 | 17 | 18220770 | G | A | 0·343558 | -0·0721459 | 0·00847379 | 1·6803E-17 | 0·26999 | 0·0345301 | 5·3254E-15 | -0·267217 | 0·0464006 | 8·4657E-09 | 0·02659592 | 14 |
| *ACSF3* | 16 | cg04308346 | 89203063 | *ACSF3* | 16 | ENSG00000176715 | 89188518 | rs34022105 | 16 | 89229598 | A | G | 0·134969 | 0·0811924 | 0·0122859 | 3·8797E-11 | -0·345108 | 0·0453454 | 2·7273E-14 | -0·235267 | 0·0471484 | 6·0404E-07 | 0·1664197 | 20 |
| *RHOT2* | 16 | cg04365973 | 717556 | *RHOT2* | 16 | ENSG00000140983 | 721130 | rs3743912 | 16 | 720960 | T | C | 0·107362 | -0·106073 | 0·0143145 | 1·2611E-13 | -0·511755 | 0·0521859 | 1·057E-22 | 0·207273 | 0·0350592 | 3·3781E-09 | 0·03349062 | 7 |
| *COX4I1* | 16 | cg04399085 | 85832271 | *COX4I1* | 16 | ENSG00000131143 | 85836444 | rs76341119 | 16 | 85809290 | A | G | 0·0521472 | 0·310423 | 0·0174807 | 1·4947E-70 | -0·621013 | 0·0689356 | 2·0872E-19 | -0·499866 | 0·0622192 | 9·439E-16 | 0·2952821 | 12 |
| *CYB5B* | 16 | cg04406620 | 69499075 | *CYB5B* | 16 | ENSG00000103018 | 69479298 | rs153051 | 16 | 69447446 | T | A | 0·45501 | -0·0867867 | 0·00807378 | 5·9778E-27 | 0·253717 | 0·0327416 | 9·2559E-15 | -0·342061 | 0·0544166 | 3·2582E-10 | 0·01581399 | 8 |
| *CYB5B* | 16 | cg04406620 | 69499075 | *PDF* | 16 | ENSG00000258429 | 69363511 | rs153051 | 16 | 69447446 | T | A | 0·45501 | 0·067105 | 0·00932791 | 6·292E-13 | 0·253717 | 0·0327416 | 9·2559E-15 | 0·264488 | 0·050166 | 1·3476E-07 | 0·0437829 | 9 |
| *TRAP1* | 16 | cg04439028 | 3722002 | *TRAP1* | 16 | ENSG00000126602 | 3734619 | rs1053874 | 16 | 3707747 | A | G | 0·302658 | 0·141636 | 0·0128515 | 3·0268E-28 | -0·230059 | 0·0344378 | 2·3826E-11 | -0·615651 | 0·107766 | 1·111E-08 | 0·01255546 | 19 |
| *SLC25A18* | 22 | cg04460557 | 18042646 | *BCL2L13* | 22 | ENSG00000099968 | 18162504 | rs174351 | 22 | 18038786 | A | G | 0·288344 | 0·0903023 | 0·00842592 | 8·4522E-27 | 0·27569 | 0·0346994 | 1·9404E-15 | 0·32755 | 0·05132 | 1·7421E-10 | 0·1274288 | 5 |
| *SDHAF1* | 19 | cg04579398 | 36485360 | *SDHAF1* | 19 | ENSG00000205138 | 36486655 | rs4805149 | 19 | 36483832 | T | C | 0·162577 | -0·259919 | 0·0124906 | 3·5727E-96 | -0·346142 | 0·0496796 | 3·2266E-12 | 0·750903 | 0·113653 | 3·9227E-11 | 0·08941666 | 5 |
| *NDUFA11* | 19 | cg04582374 | 5904794 | *NDUFA11* | 19 | ENSG00000174886 | 5897652 | rs12984793 | 19 | 5870832 | T | A | 0·320041 | -0·102784 | 0·0107288 | 9·6857E-22 | 0·261437 | 0·036985 | 1·5635E-12 | -0·39315 | 0·0691193 | 1·2854E-08 | 0·1602016 | 11 |
| *MACROD1* | 11 | cg04582871 | 63827695 | *BAD* | 11 | ENSG00000002330 | 64044739 | rs2282492 | 11 | 63918589 | C | T | 0·329243 | 0·0446763 | 0·0090124 | 7·1516E-07 | 0·276128 | 0·0325295 | 2·0924E-17 | 0·161796 | 0·0377965 | 1·863E-05 | 0·01142492 | 12 |
| *MACROD1* | 11 | cg04582871 | 63827695 | *DNAJC4* | 11 | ENSG00000110011 | 63999754 | rs2282492 | 11 | 63918589 | C | T | 0·329243 | -0·0516647 | 0·00901087 | 9·8321E-09 | 0·276128 | 0·0325295 | 2·0924E-17 | -0·187104 | 0·0393796 | 2·0212E-06 | 0·1856369 | 9 |
| *AMACR* | 5 | cg04596544 | 33997325 | *AMACR* | 5 | ENSG00000242110 | 33997251 | rs40507 | 5 | 34003361 | C | T | 0·180982 | 0·31061 | 0·0106305 | 1·12E-187 | -0·236077 | 0·0408798 | 7·6998E-09 | -1·31571 | 0·232241 | 1·4676E-08 | 0·04428028 | 5 |
| *ACSF3* | 16 | cg04753738 | 89160056 | *ACSF3* | 16 | ENSG00000176715 | 89188518 | rs4782474 | 16 | 89157547 | G | A | 0·45501 | 0·0631159 | 0·00896609 | 1·9307E-12 | 0·236165 | 0·0321084 | 1·9059E-13 | 0·267253 | 0·052551 | 3·6647E-07 | 0·04638757 | 16 |
| *ACACB* | 12 | cg04777824 | 110000000 | *UNG* | 12 | ENSG00000076248 | 109542088 | rs34770418 | 12 | 109592852 | G | C | 0·169734 | 0·139433 | 0·0101617 | 7·5592E-43 | -0·229337 | 0·0398707 | 8·8184E-09 | -0·607983 | 0·114611 | 1·1281E-07 | 0·1294298 | 8 |
| *ACACB* | 12 | cg04777824 | 110000000 | *ACACB* | 12 | ENSG00000076555 | 109630215 | rs34770418 | 12 | 109592852 | G | C | 0·169734 | -0·555327 | 0·00957367 | 0 | -0·229337 | 0·0398707 | 8·8184E-09 | 2·42145 | 0·423038 | 1·0408E-08 | 0·3059455 | 9 |
| *L2HGDH* | 14 | cg04800684 | 50778323 | *DMAC2L* | 14 | ENSG00000125375 | 50790660 | rs4901011 | 14 | 50768483 | A | G | 0·45092 | 0·223304 | 0·0079559 | 2·428E-173 | -0·269634 | 0·0320986 | 4·4581E-17 | -0·828174 | 0·102911 | 8·4504E-16 | 0·1945207 | 20 |
| *NUDT13* | 10 | cg04833713 | 74871078 | *MICU1* | 10 | ENSG00000107745 | 74256498 | rs7090105 | 10 | 75131545 | T | C | 0·245399 | 0·154819 | 0·0208735 | 1·198E-13 | 0·314493 | 0·0384283 | 2·7485E-16 | 0·492281 | 0·0895744 | 3·8898E-08 | 0·08141648 | 6 |
| *NUDT13* | 10 | cg04833713 | 74871078 | *NUDT13* | 10 | ENSG00000166321 | 74880901 | rs12258130 | 10 | 75005420 | C | T | 0·0633947 | -0·508552 | 0·0145767 | 1·13E-266 | 1·04867 | 0·0630985 | 5·0288E-62 | -0·484949 | 0·0323211 | 6·9004E-51 | 0·01392417 | 6 |
| *NUDT13* | 10 | cg04833713 | 74871078 | *MRPS16* | 10 | ENSG00000182180 | 75009480 | rs12258130 | 10 | 75005420 | C | T | 0·0633947 | 0·317667 | 0·0147483 | 6·685E-103 | 1·04867 | 0·0630985 | 5·0288E-62 | 0·302924 | 0·023022 | 1·5305E-39 | 0·06409857 | 6 |
| *MRPL16* | 11 | cg04862679 | 59578540 | *MRPL16* | 11 | ENSG00000166902 | 59575976 | rs11230085 | 11 | 59578322 | A | G | 0·0838446 | -0·102641 | 0·0161284 | 1·9657E-10 | 1·15292 | 0·0592298 | 2·1666E-84 | -0·089027 | 0·0147178 | 1·4582E-09 | 0·9495129 | 7 |
| *SPG7* | 16 | cg04864846 | 89614532 | *SPG7* | 16 | ENSG00000197912 | 89590750 | rs80324518 | 16 | 89614534 | T | C | 0·0807771 | -0·196704 | 0·0153175 | 9·5625E-38 | 0·293956 | 0·0529875 | 2·8953E-08 | -0·669161 | 0·131395 | 3·529E-07 | 0·05754517 | 4 |
| *SPG7* | 16 | cg04864846 | 89614532 | *LOC101930112* | 16 | ENSG00000197912 | 89590750 | rs80324518 | 16 | 89614534 | T | C | 0·0807771 | -0·196704 | 0·0153175 | 9·5625E-38 | 0·293956 | 0·0529875 | 2·8953E-08 | -0·669161 | 0·131395 | 3·529E-07 | 0·05754517 | 4 |
| *ATPAF1* | 1 | cg04879861 | 47134311 | *ATPAF1* | 1 | ENSG00000123472 | 47118974 | rs1371834 | 1 | 47176894 | G | A | 0·213701 | 0·141324 | 0·010252 | 3·1377E-43 | -0·421383 | 0·0362984 | 3·7155E-31 | -0·335381 | 0·0377698 | 6·7098E-19 | 0·3415901 | 6 |
| *PNKD* | 2 | cg04880052 | 219000000 | *PNKD* | 2 | ENSG00000127838 | 219173315 | rs3731861 | 2 | 219191256 | C | T | 0·343558 | -0·498378 | 0·00784161 | 0 | -0·384029 | 0·0328473 | 1·4117E-31 | 1·29776 | 0·112865 | 1·3439E-30 | 0·02187555 | 20 |
| *MGST1* | 12 | cg04885072 | 16513009 | *MGST1* | 12 | ENSG00000008394 | 16631134 | rs10505798 | 12 | 16340120 | G | A | 0·46728 | 0·064853 | 0·00795801 | 3·6573E-16 | 0·20631 | 0·0325534 | 2·334E-10 | 0·314347 | 0·0628339 | 5·6494E-07 | 0·435982 | 14 |
| *MACROD1* | 11 | cg04935121 | 63775413 | *COX8A* | 11 | ENSG00000176340 | 63743047 | rs4980517 | 11 | 63745677 | T | C | 0·396728 | -0·159966 | 0·0152027 | 6·8261E-26 | -0·454975 | 0·0318888 | 3·4871E-46 | 0·351593 | 0·0415186 | 2·4894E-17 | 0·5651549 | 20 |
| *MACROD1* | 11 | cg05016508 | 63871570 | *DNAJC4* | 11 | ENSG00000110011 | 63999754 | rs11603384 | 11 | 63949095 | A | G | 0·305726 | -0·0559175 | 0·00918729 | 1·1548E-09 | 0·436777 | 0·0330867 | 8·6608E-40 | -0·128023 | 0·0231623 | 3·2536E-08 | 0·05490042 | 11 |
| *SLC25A29* | 14 | cg05043627 | 101000000 | *SLC25A29* | 14 | ENSG00000197119 | 100765166 | rs55663021 | 14 | 100763862 | A | G | 0·0439673 | -0·310737 | 0·0221578 | 1·1148E-44 | -1·0596 | 0·065724 | 1·788E-58 | 0·293259 | 0·0277158 | 3·6538E-26 | 0·04171688 | 19 |
| *GPX1* | 3 | cg05055782 | 49396193 | *GPX1* | 3 | ENSG00000233276 | 49395321 | rs4855855 | 3 | 49559984 | T | C | 0·433538 | 0·0840087 | 0·00839835 | 1·4785E-23 | 0·206358 | 0·0323941 | 1·8873E-10 | 0·407102 | 0·0757655 | 7·7358E-08 | 0·1506319 | 10 |
| *RHOT2* | 16 | cg05135288 | 717707 | *MCRIP2* | 16 | ENSG00000172366 | 695143 | rs4984681 | 16 | 719933 | C | T | 0·379346 | -0·0552778 | 0·00936626 | 3·5956E-09 | 0·460185 | 0·0327768 | 8·8773E-45 | -0·120121 | 0·0220784 | 5·3084E-08 | 0·6771693 | 5 |
| *SLC25A28* | 10 | cg05149213 | 101000000 | *SLC25A28* | 10 | ENSG00000155287 | 101375324 | rs2052318 | 10 | 101381519 | T | C | 0·468303 | 0·129616 | 0·00793166 | 4·9925E-60 | -0·551572 | 0·031652 | 5·2283E-68 | -0·234994 | 0·0197139 | 9·2854E-33 | 0·1052235 | 20 |
| *SDSL* | 12 | cg05149586 | 114000000 | *SDSL* | 12 | ENSG00000139410 | 113868061 | rs35764330 | 12 | 113832378 | C | T | 0·0378323 | 0·797313 | 0·0229567 | 2·668E-264 | -0·571976 | 0·0980429 | 5·4134E-09 | -1·39396 | 0·242288 | 8·7499E-09 | 0·8998604 | 11 |
| *AIFM3* | 22 | cg05156805 | 21318995 | *AIFM3* | 22 | ENSG00000183773 | 21327522 | rs61588756 | 22 | 21316557 | A | C | 0·170757 | -0·238483 | 0·0139021 | 5·8269E-66 | 0·510249 | 0·0464827 | 4·92E-28 | -0·467386 | 0·0505491 | 2·3268E-20 | 0·6223683 | 4 |
| *ALDH6A1* | 14 | cg05179529 | 74551516 | *ISCA2* | 14 | ENSG00000165898 | 74962116 | rs118177643 | 14 | 74911219 | T | C | 0·0347648 | -0·143566 | 0·0223533 | 1·3397E-10 | 0·664072 | 0·102136 | 7·9347E-11 | -0·21619 | 0·0473145 | 4·8955E-06 | 0·03211987 | 7 |
| *ACADM* | 1 | cg05258079 | 76189192 | *CRYZ* | 1 | ENSG00000116791 | 75185131 | rs7518038 | 1 | 76154430 | A | G | 0·306748 | 0·0455458 | 0·00972121 | 2·7969E-06 | 0·417236 | 0·0346335 | 2·0068E-33 | 0·109161 | 0·024999 | 1·2619E-05 | 0·0112858 | 3 |
| *GATM* | 15 | cg05280133 | 45670068 | *GATM* | 15 | ENSG00000171766 | 45673869 | rs56850226 | 15 | 45633118 | C | T | 0·279141 | -0·572783 | 0·00830097 | 0 | 0·509406 | 0·0355932 | 1·8438E-46 | -1·12441 | 0·080237 | 1·2862E-44 | 0·04612817 | 20 |
| *BCKDHA* | 19 | cg05360949 | 41903737 | *DMAC2* | 19 | ENSG00000105341 | 41941922 | rs111867059 | 19 | 41900809 | T | C | 0·122699 | -0·177358 | 0·0135481 | 3·7069E-39 | 0·434053 | 0·0468354 | 1·9033E-20 | -0·408609 | 0·0540201 | 3·9081E-14 | 0·2008125 | 3 |
| *DHX30* | 3 | cg05411186 | 47890988 | *NME6* | 3 | ENSG00000172113 | 48338964 | rs111499603 | 3 | 47588649 | T | C | 0·264826 | -0·0977318 | 0·0101745 | 7·5716E-22 | 0·200288 | 0·0364602 | 3·9441E-08 | -0·487956 | 0·102327 | 1·855E-06 | 0·3903939 | 3 |
| *ACSF3* | 16 | cg05445348 | 89219472 | *ACSF3* | 16 | ENSG00000176715 | 89188518 | rs117716265 | 16 | 89260678 | G | C | 0·107362 | 0·0971036 | 0·0136141 | 9·849E-13 | 0·315555 | 0·0498037 | 2·3584E-10 | 0·307723 | 0·0649627 | 2·1699E-06 | 0·5655103 | 16 |
| *ACSF3* | 16 | cg05445348 | 89219472 | *SPG7* | 16 | ENSG00000197912 | 89590750 | rs34387772 | 16 | 89213433 | T | C | 0·278119 | -0·052583 | 0·00942652 | 2·4302E-08 | 0·281926 | 0·0349948 | 7·8693E-16 | -0·186513 | 0·040669 | 4·5153E-06 | 0·5746682 | 5 |
| *ACSF3* | 16 | cg05445348 | 89219472 | *LOC101930112* | 16 | ENSG00000197912 | 89590750 | rs34387772 | 16 | 89213433 | T | C | 0·278119 | -0·052583 | 0·00942652 | 2·4302E-08 | 0·281926 | 0·0349948 | 7·8693E-16 | -0·186513 | 0·040669 | 4·5153E-06 | 0·5746682 | 5 |
| *CYP11A1* | 15 | cg05454635 | 74657911 | *CYP11A1* | 15 | ENSG00000140459 | 74645090 | rs12898858 | 15 | 74641464 | A | G | 0·274029 | 0·0634392 | 0·00901356 | 1·9474E-12 | 0·24426 | 0·0372417 | 5·4251E-11 | 0·25972 | 0·0541275 | 1·6002E-06 | 0·03426694 | 5 |
| *MFN2* | 1 | cg05523254 | 12040215 | *MFN2* | 1 | ENSG00000116688 | 12056904 | rs3766746 | 1 | 12031866 | T | C | 0·458078 | -0·500909 | 0·00745002 | 0 | 0·188329 | 0·0319339 | 3·6915E-09 | -2·65975 | 0·452732 | 4·231E-09 | 0·01100666 | 6 |
| *GPX1* | 3 | cg05551922 | 49396232 | *GPX1* | 3 | ENSG00000233276 | 49395321 | rs4241406 | 3 | 49600426 | T | C | 0·435583 | 0·0826477 | 0·00839762 | 7·4368E-23 | 0·192934 | 0·032526 | 2·9983E-09 | 0·428373 | 0·0843202 | 3·7681E-07 | 0·1937824 | 7 |
| *COQ3* | 6 | cg05567646 | 99842244 | *COQ3* | 6 | ENSG00000132423 | 99829678 | rs17179548 | 6 | 99842889 | G | A | 0·183027 | 0·0516731 | 0·0108507 | 1·9149E-06 | -0·42759 | 0·0426415 | 1·1534E-23 | -0·120847 | 0·0280927 | 1·6946E-05 | 0·02251153 | 5 |
| *SPATA20* | 17 | cg05652817 | 48626506 | *SPATA20* | 17 | ENSG00000006282 | 48626816 | rs80277878 | 17 | 48624045 | G | A | 0·099182 | -0·872319 | 0·0127358 | 0 | -0·331079 | 0·050043 | 3·6931E-11 | 2·63478 | 0·400103 | 4·5413E-11 | 0·04645814 | 12 |
| *ALDH7A1* | 5 | cg05689600 | 126000000 | *ALDH7A1* | 5 | ENSG00000164904 | 125904321 | rs62391691 | 5 | 126078913 | A | G | 0·0552147 | -0·27652 | 0·0264147 | 1·2067E-25 | 0·887853 | 0·0729318 | 4·2897E-34 | -0·311448 | 0·0392385 | 2·0661E-15 | 0·1129547 | 11 |
| *ME3* | 11 | cg05705813 | 86383809 | *ME3* | 11 | ENSG00000151376 | 86267914 | rs12098937 | 11 | 86384063 | G | C | 0·126789 | 0·649796 | 0·0113153 | 0 | -0·50165 | 0·0483287 | 3·0591E-25 | -1·29532 | 0·126812 | 1·7086E-24 | 0·04214348 | 20 |
| *LDHD* | 16 | cg05820066 | 75145843 | *LDHD* | 16 | ENSG00000166816 | 75148213 | rs1043503 | 16 | 75144297 | A | G | 0·129857 | -0·0943054 | 0·011526 | 2·7917E-16 | 0·319942 | 0·0477324 | 2·0442E-11 | -0·294758 | 0·0568474 | 2·1593E-07 | 0·01790475 | 12 |
| *MRPL27* | 17 | cg05868183 | 48450068 | *MRPL27* | 17 | ENSG00000108826 | 48447896 | rs11871126 | 17 | 48450227 | T | C | 0·440695 | -0·164725 | 0·00806069 | 8·0694E-93 | -0·430345 | 0·0311018 | 1·5315E-43 | 0·382774 | 0·0334085 | 2·1588E-30 | 0·02006142 | 20 |
| *THEM4* | 1 | cg05927068 | 152000000 | *TDRKH* | 1 | ENSG00000182134 | 151753237 | rs74940264 | 1 | 151833396 | T | C | 0·0265849 | -0·175587 | 0·0260368 | 1·543E-11 | 0·779665 | 0·109266 | 9·6432E-13 | -0·225208 | 0·0459495 | 9·5246E-07 | 0·691315 | 7 |
| *ALKBH7* | 19 | cg05968233 | 6372277 | *ALKBH7* | 19 | ENSG00000125652 | 6373743 | rs74525335 | 19 | 6372431 | G | T | 0·0920245 | 0·324543 | 0·0262215 | 3·4811E-35 | -0·978641 | 0·0539027 | 1·1579E-73 | -0·331626 | 0·0324275 | 1·5055E-24 | 0·357122 | 20 |
| *CHCHD6* | 3 | cg05977955 | 127000000 | *CHCHD6* | 3 | ENSG00000159685 | 126551156 | rs58383276 | 3 | 126635468 | T | G | 0·122699 | -0·0793612 | 0·0118747 | 2·3378E-11 | 0·331401 | 0·0484391 | 7·8313E-12 | -0·239472 | 0·0500908 | 1·7464E-06 | 0·0617252 | 16 |
| *PNKD* | 2 | cg05991184 | 219000000 | *PNKD* | 2 | ENSG00000127838 | 219173315 | rs2015863 | 2 | 219185161 | T | C | 0·339468 | -0·501558 | 0·00786795 | 0 | 0·231007 | 0·0333565 | 4·3477E-12 | -2·17118 | 0·315355 | 5·7836E-12 | 0·5106879 | 20 |
| *IMMT* | 2 | cg06002975 | 86422967 | *IMMT* | 2 | ENSG00000132305 | 86396974 | rs13403186 | 2 | 86405016 | A | G | 0·49591 | 0·509352 | 0·00746744 | 0 | -0·425282 | 0·0321333 | 5·5162E-40 | -1·19768 | 0·0921818 | 1·3478E-38 | 0·02655064 | 20 |
| *MRPS35* | 12 | cg06024135 | 27863332 | *MRPS35* | 12 | ENSG00000061794 | 27886467 | rs7307900 | 12 | 27851134 | C | A | 0·368098 | -0·132558 | 0·00812283 | 7·2076E-60 | -0·205544 | 0·0325262 | 2·6269E-10 | 0·644913 | 0·109438 | 3·7938E-09 | 0·05456059 | 6 |
| *DNA2* | 10 | cg06032048 | 70231928 | *SLC25A16* | 10 | ENSG00000122912 | 70264876 | rs10998210 | 10 | 70237160 | C | T | 0·145194 | 0·585147 | 0·0104386 | 0 | -0·666018 | 0·0477008 | 2·643E-44 | -0·878575 | 0·0648469 | 8·0902E-42 | 0·02068239 | 20 |
| *DNM1L* | 12 | cg06077899 | 32832894 | *YARS2* | 12 | ENSG00000139131 | 32894630 | rs10844299 | 12 | 32844234 | A | G | 0·161554 | 0·106642 | 0·0174703 | 1·0332E-09 | 0·707965 | 0·0435973 | 2·6847E-59 | 0·150632 | 0·0263626 | 1·1046E-08 | 0·4066454 | 5 |
| *PYCR2* | 1 | cg06086141 | 226000000 | *PYCR2* | 1 | ENSG00000143811 | 226109778 | rs77157663 | 1 | 226111945 | C | T | 0·0613497 | 0·640337 | 0·0189449 | 1·975E-250 | -0·488837 | 0·0691933 | 1·6084E-12 | -1·30992 | 0·189422 | 4·6667E-12 | 0·3445231 | 4 |
| *NDUFB10* | 16 | cg06141561 | 2011215 | *NDUFB10* | 16 | ENSG00000140990 | 2010742 | rs72764891 | 16 | 2013588 | C | T | 0·127812 | 0·126526 | 0·0129019 | 1·0525E-22 | -0·326257 | 0·0461633 | 1·578E-12 | -0·387811 | 0·0676375 | 9·8282E-09 | 0·1166533 | 16 |
| *MRPL16* | 11 | cg06171787 | 59578490 | *MRPL16* | 11 | ENSG00000166902 | 59575976 | rs11230067 | 11 | 59516411 | T | G | 0·0838446 | -0·109791 | 0·0157818 | 3·4809E-12 | 0·448455 | 0·0619355 | 4·4644E-13 | -0·244821 | 0·0488025 | 5·2606E-07 | 0·7498665 | 7 |
| *C16orf91* | 16 | cg06180061 | 1473469 | *MRPS34* | 16 | ENSG00000074071 | 1822523 | rs4786363 | 16 | 1643247 | T | C | 0·0531697 | 0·10973 | 0·0188358 | 5·6908E-09 | -0·566644 | 0·0752814 | 5·1915E-14 | -0·193649 | 0·042034 | 4·0859E-06 | 0·1286342 | 3 |
| *C16orf91* | 16 | cg06180061 | 1473469 | *NME3* | 16 | ENSG00000103024 | 1821009 | rs4786363 | 16 | 1643247 | T | C | 0·0531697 | 0·139577 | 0·0188852 | 1·4595E-13 | -0·566644 | 0·0752814 | 5·1915E-14 | -0·246322 | 0·0467087 | 1·3379E-07 | 0·04241323 | 5 |
| *MACROD1* | 11 | cg06268327 | 63885740 | *BAD* | 11 | ENSG00000002330 | 64044739 | rs6591838 | 11 | 63959356 | G | A | 0·214724 | 0·0526213 | 0·00920435 | 1·0842E-08 | -0·318679 | 0·0373438 | 1·4179E-17 | -0·165123 | 0·0347653 | 2·0376E-06 | 0·02250065 | 12 |
| *SLC25A10* | 17 | cg06380072 | 79687834 | *MRPL12* | 17 | ENSG00000262814 | 79672471 | rs62077190 | 17 | 79676109 | A | C | 0·174847 | -0·141453 | 0·0133597 | 3·3875E-26 | -0·645718 | 0·0466239 | 1·2811E-43 | 0·219063 | 0·0260434 | 4·0488E-17 | 0·265662 | 20 |
| *DLAT* | 11 | cg06437417 | 112000000 | *BCO2* | 11 | ENSG00000197580 | 112070806 | rs11214048 | 11 | 111814258 | G | C | 0·328221 | 0·0563206 | 0·00832677 | 1·3442E-11 | 0·215665 | 0·0312619 | 5·2497E-12 | 0·261149 | 0·0540714 | 1·3674E-06 | 0·4335713 | 11 |
| *PC* | 11 | cg06467504 | 66650500 | *NDUFS8* | 11 | ENSG00000110717 | 67801097 | rs72936340 | 11 | 67470895 | A | G | 0·0725971 | 0·0766894 | 0·0149848 | 3·0912E-07 | -0·580236 | 0·0611008 | 2·1733E-21 | -0·132169 | 0·029337 | 6·6308E-06 | 0·02687657 | 4 |
| *PC* | 11 | cg06467504 | 66650500 | *NUDT8* | 11 | ENSG00000167799 | 67396405 | rs7108149 | 11 | 67362363 | T | C | 0·349693 | 0·0446705 | 0·00840177 | 1·0561E-07 | -0·330392 | 0·0335852 | 7·7665E-23 | -0·135205 | 0·0289061 | 2·9058E-06 | 0·4931031 | 5 |
| *ACSM3* | 16 | cg06478823 | 20774873 | *LYRM1* | 16 | ENSG00000102897 | 20923759 | rs11642550 | 16 | 20854647 | A | G | 0·320041 | 0·099737 | 0·00856597 | 2·4793E-31 | 0·428703 | 0·034897 | 1·093E-34 | 0·232648 | 0·0275298 | 2·8935E-17 | 0·01413541 | 12 |
| *SUOX* | 12 | cg06495347 | 56391451 | *SUOX* | 12 | ENSG00000139531 | 56395694 | rs1702877 | 12 | 56427808 | T | C | 0·328221 | -0·325323 | 0·0084006 | 0 | -0·18407 | 0·0329835 | 2·396E-08 | 1·76739 | 0·31997 | 3·3211E-08 | 0·1814138 | 5 |
| *MACROD1* | 11 | cg06497934 | 63899087 | *DNAJC4* | 11 | ENSG00000110011 | 63999754 | rs7112960 | 11 | 63907079 | G | A | 0·322086 | -0·0528835 | 0·00901004 | 4·3738E-09 | -0·556859 | 0·0313835 | 1·929E-70 | 0·0949675 | 0·0170424 | 2·512E-08 | 0·1910383 | 9 |
| *MACROD1* | 11 | cg06497934 | 63899087 | *COX8A* | 11 | ENSG00000176340 | 63743047 | rs503735 | 11 | 63797679 | C | T | 0·47546 | -0·115404 | 0·0150583 | 1·8051E-14 | 0·210314 | 0·0323232 | 7·6875E-11 | -0·548722 | 0·110628 | 7·0469E-07 | 0·01392771 | 17 |
| *ACACB* | 12 | cg06516150 | 110000000 | *ACACB* | 12 | ENSG00000076555 | 109630215 | rs2268405 | 12 | 109589593 | G | A | 0·192229 | -0·56872 | 0·00916456 | 0 | -0·436719 | 0·0383337 | 4·5545E-30 | 1·30226 | 0·116218 | 3·8409E-29 | 0·02334134 | 20 |
| *OXCT1* | 5 | cg06537708 | 41871170 | *OXCT1* | 5 | ENSG00000083720 | 41800394 | rs12186512 | 5 | 41802771 | G | A | 0·327198 | 0·0934816 | 0·0088034 | 2·4367E-26 | -0·201917 | 0·0348763 | 7·0589E-09 | -0·46297 | 0·0910802 | 3·7131E-07 | 0·07820079 | 3 |
| *PCCA* | 13 | cg06568323 | 101000000 | *PCCA* | 13 | ENSG00000175198 | 100961977 | rs59406319 | 13 | 100773120 | C | T | 0·116564 | -0·151626 | 0·0137899 | 4·0212E-28 | -0·317777 | 0·0530168 | 2·0488E-09 | 0·477146 | 0·090665 | 1·4193E-07 | 0·3190763 | 4 |
| *ECHDC2* | 1 | cg06600287 | 53387719 | *SCP2* | 1 | ENSG00000116171 | 53455138 | rs12405280 | 1 | 53423528 | G | T | 0·364008 | -0·690832 | 0·00739269 | 0 | 0·611575 | 0·0320507 | 3·5975E-81 | -1·12959 | 0·06042 | 5·3636E-78 | 0·01883952 | 20 |
| *ACSF3* | 16 | cg06615999 | 89178870 | *ACSF3* | 16 | ENSG00000176715 | 89188518 | rs4782329 | 16 | 89178839 | T | C | 0·182004 | 0·0645444 | 0·0101195 | 1·7918E-10 | -0·268575 | 0·0395957 | 1·1775E-11 | -0·240322 | 0·0517202 | 3·375E-06 | 0·06846653 | 7 |
| *GRPEL1* | 4 | cg06697600 | 7070879 | *GRPEL1* | 4 | ENSG00000109519 | 7065278 | rs4689586 | 4 | 7067413 | A | G | 0·231084 | -0·110207 | 0·00982307 | 3·2823E-29 | 0·739443 | 0·0378643 | 6·2554E-85 | -0·149041 | 0·0153206 | 2·2881E-22 | 0·3858561 | 20 |
| *DMPK* | 19 | cg06710981 | 46282571 | *DMPK* | 19 | ENSG00000104936 | 46279392 | rs572634 | 19 | 46282503 | C | A | 0·115542 | 0·11896 | 0·0117183 | 3·2597E-24 | -0·319553 | 0·0496932 | 1·2717E-10 | -0·37227 | 0·0685285 | 5·5619E-08 | 0·1977282 | 4 |
| *NSUN4* | 1 | cg06741803 | 46807522 | *NSUN4* | 1 | ENSG00000117481 | 46818336 | rs56063031 | 1 | 46825433 | T | C | 0·273006 | 0·65276 | 0·0084938 | 0 | -0·356653 | 0·035562 | 1·136E-23 | -1·83024 | 0·184041 | 2·6589E-23 | 0·4997512 | 20 |
| *NDUFS2* | 1 | cg06760507 | 161000000 | *NIT1* | 1 | ENSG00000158793 | 161091555 | rs11587213 | 1 | 161184875 | G | A | 0·180982 | -0·0704898 | 0·0105895 | 2·8023E-11 | -0·497357 | 0·0412421 | 1·7291E-33 | 0·141729 | 0·0243197 | 5·6184E-09 | 0·2143319 | 5 |
| *CASP3* | 4 | cg06796944 | 186000000 | *PRIMPOL* | 4 | ENSG00000164306 | 185593442 | rs4861629 | 4 | 185573312 | C | G | 0·166667 | 0·251071 | 0·0190269 | 9·3011E-40 | 0·360797 | 0·045876 | 3·7024E-15 | 0·695879 | 0·103006 | 1·4211E-11 | 0·4638237 | 20 |
| *FASTK* | 7 | cg06807926 | 151000000 | *FASTK* | 7 | ENSG00000164896 | 150775832 | rs34308016 | 7 | 150773664 | T | C | 0·176892 | 0·107889 | 0·0112904 | 1·2263E-21 | 0·410185 | 0·0383846 | 1·1811E-26 | 0·263025 | 0·0369251 | 1·0544E-12 | 0·7452818 | 16 |
| *COX7A2* | 6 | cg06809298 | 75953853 | *COX7A2* | 6 | ENSG00000112695 | 75953715 | rs9360898 | 6 | 75953705 | G | T | 0·198364 | 0·0668398 | 0·0145519 | 4·365E-06 | -0·862497 | 0·0369002 | 7·902E-121 | -0·0774957 | 0·0171945 | 6·575E-06 | 0·02672628 | 3 |
| *EFHD1* | 2 | cg06842071 | 233000000 | *EFHD1* | 2 | ENSG00000115468 | 233509129 | rs17297115 | 2 | 233510979 | C | T | 0·361963 | -0·0547453 | 0·00827368 | 3·6706E-11 | -0·268088 | 0·033496 | 1·2085E-15 | 0·204206 | 0·0400429 | 3·4021E-07 | 0·03216328 | 8 |
| *SLC25A29* | 14 | cg06846458 | 101000000 | *SLC25A29* | 14 | ENSG00000197119 | 100765166 | rs1059264 | 14 | 100769163 | T | C | 0·365031 | 0·630903 | 0·00818619 | 0 | -0·243283 | 0·034079 | 9·4158E-13 | -2·59329 | 0·364822 | 1·1743E-12 | 0·2480393 | 6 |
| *COMT* | 22 | cg06860277 | 19930072 | *RTL10* | 22 | ENSG00000215012 | 19838040 | rs2078748 | 22 | 19925414 | G | A | 0·281186 | -0·0769887 | 0·00985317 | 5·5577E-15 | -0·217785 | 0·0348136 | 3·9561E-10 | 0·353508 | 0·0723892 | 1·0425E-06 | 0·02566727 | 7 |
| *TXNRD2* | 22 | cg06860277 | 19930072 | *RTL10* | 22 | ENSG00000215012 | 19838040 | rs2078748 | 22 | 19925414 | G | A | 0·281186 | -0·0769887 | 0·00985317 | 5·5577E-15 | -0·217785 | 0·0348136 | 3·9561E-10 | 0·353508 | 0·0723892 | 1·0425E-06 | 0·02566727 | 7 |
| *SND1* | 7 | cg06903658 | 128000000 | *ARF5* | 7 | ENSG00000004059 | 127230079 | rs68030304 | 7 | 127694031 | G | A | 0·275051 | 0·0499685 | 0·00884742 | 1·6251E-08 | -0·311897 | 0·0359875 | 4·444E-18 | -0·160208 | 0·033858 | 2·2256E-06 | 0·07796778 | 10 |
| *CAT* | 11 | cg06908474 | 34460516 | *CAT* | 11 | ENSG00000121691 | 34477040 | rs10836229 | 11 | 34447300 | G | A | 0·392638 | 0·647557 | 0·0073557 | 0 | -0·20419 | 0·0332526 | 8·2223E-10 | -3·17135 | 0·517712 | 9·029E-10 | 0·4928143 | 15 |
| *RPUSD4* | 11 | cg06912824 | 126000000 | *RPUSD4* | 11 | ENSG00000165526 | 126076790 | rs527051 | 11 | 126066948 | T | C | 0·204499 | -0·472715 | 0·00926748 | 0 | -0·258579 | 0·0401741 | 1·2229E-10 | 1·82813 | 0·286279 | 1·7048E-10 | 0·01597498 | 11 |
| *MRPL16* | 11 | cg06925083 | 59578514 | *MRPL16* | 11 | ENSG00000166902 | 59575976 | rs11230085 | 11 | 59578322 | A | G | 0·0838446 | -0·102641 | 0·0161284 | 1·9657E-10 | 1·14355 | 0·0596947 | 8·5177E-82 | -0·0897565 | 0·0148617 | 1·5464E-09 | 0·5577593 | 7 |
| *OSBPL1A* | 18 | cg06997114 | 21851455 | *OSBPL1A* | 18 | ENSG00000141447 | 21859926 | rs66851072 | 18 | 22002305 | G | A | 0·0766871 | 0·0762875 | 0·013871 | 3·8022E-08 | 0·383339 | 0·0548714 | 2·8257E-12 | 0·199008 | 0·046052 | 1·5506E-05 | 0·1855625 | 3 |
| *MRPS17* | 7 | cg07023327 | 56019384 | *NIPSNAP2* | 7 | ENSG00000146729 | 56043680 | rs4275190 | 7 | 56104388 | C | T | 0·331288 | 0·102389 | 0·00863532 | 1·9794E-32 | -0·330523 | 0·0353813 | 9·4769E-21 | -0·309779 | 0·0422163 | 2·1697E-13 | 0·05337737 | 20 |
| *CLPB* | 11 | cg07062262 | 72142353 | *CLPB* | 11 | ENSG00000162129 | 72074580 | rs12295464 | 11 | 72153384 | C | T | 0·0501022 | -0·147856 | 0·0186404 | 2·1564E-15 | -1·08729 | 0·0762293 | 3·699E-46 | 0·135986 | 0·0196166 | 4·1437E-12 | 0·2666774 | 20 |
| *DCXR* | 17 | cg07073120 | 79994884 | *FASN* | 17 | ENSG00000169710 | 80046211 | rs74512221 | 17 | 79992508 | G | C | 0·122699 | -0·173359 | 0·0182337 | 1·9511E-21 | -0·287982 | 0·0482313 | 2·3601E-09 | 0·601979 | 0·119052 | 4·2719E-07 | 0·02140116 | 10 |
| *DCXR* | 17 | cg07073120 | 79994884 | *DCXR* | 17 | ENSG00000169738 | 79994310 | rs74512221 | 17 | 79992508 | G | C | 0·122699 | 0·843972 | 0·0130301 | 0 | -0·287982 | 0·0482313 | 2·3601E-09 | -2·93064 | 0·492906 | 2·7537E-09 | 0·4257664 | 10 |
| *UCP3* | 11 | cg07091552 | 73721170 | *UCP3* | 11 | ENSG00000175564 | 73715903 | rs642154 | 11 | 73684945 | G | T | 0·135992 | -0·115399 | 0·0112523 | 1·1168E-24 | 0·255533 | 0·0462311 | 3·2519E-08 | -0·451601 | 0·0928146 | 1·1409E-06 | 0·02402418 | 5 |
| *UCP3* | 11 | cg07091552 | 73721170 | *MRPL48* | 11 | ENSG00000175581 | 73537269 | rs146217454 | 11 | 74001808 | A | G | 0·0685072 | 0·182046 | 0·0251361 | 4·4081E-13 | 0·463344 | 0·064601 | 7·3698E-13 | 0·392896 | 0·0770956 | 3·465E-07 | 0·01142709 | 14 |
| *TK2* | 16 | cg07127456 | 66583081 | *TK2* | 16 | ENSG00000166548 | 66564176 | rs2290183 | 16 | 66613841 | C | G | 0·132924 | 0·719804 | 0·0116359 | 0 | -1·29171 | 0·0442435 | 2·213E-187 | -0·557249 | 0·0211058 | 1·276E-153 | 0·01027657 | 20 |
| *MRPL51* | 12 | cg07142400 | 6602447 | *MRPL51* | 12 | ENSG00000111639 | 6602078 | rs11610712 | 12 | 6612883 | C | G | 0·307771 | -0·193128 | 0·00880488 | 1·226E-106 | 0·241889 | 0·0356291 | 1·1285E-11 | -0·798416 | 0·123107 | 8·8427E-11 | 0·4635971 | 5 |
| *HINT1* | 5 | cg07158495 | 130000000 | *ACSL6* | 5 | ENSG00000164398 | 131245309 | rs11746464 | 5 | 130857727 | T | G | 0·453988 | 0·0550182 | 0·00805939 | 8·6951E-12 | -0·190017 | 0·0329926 | 8·4421E-09 | -0·289544 | 0·0657751 | 1·0724E-05 | 0·0148942 | 20 |
| *HINT1* | 5 | cg07158495 | 130000000 | *HINT1* | 5 | ENSG00000169567 | 130501074 | rs10059807 | 5 | 130877684 | T | C | 0·262781 | -0·10212 | 0·0136601 | 7·6736E-14 | -0·216994 | 0·0395911 | 4·232E-08 | 0·470612 | 0·106469 | 9·861E-06 | 0·1530965 | 8 |
| *HINT1* | 5 | cg07158495 | 130000000 | *LYRM7* | 5 | ENSG00000186687 | 130523811 | rs11746464 | 5 | 130857727 | T | G | 0·453988 | -0·07365 | 0·00805957 | 6·35E-20 | -0·190017 | 0·0329926 | 8·4421E-09 | 0·387597 | 0·0795493 | 1·1025E-06 | 0·366909 | 20 |
| *NDUFS5* | 1 | cg07201754 | 39491318 | *NDUFS5* | 1 | ENSG00000168653 | 39496149 | rs34848020 | 1 | 39453785 | A | G | 0·44683 | -0·533192 | 0·0074418 | 0 | -0·195821 | 0·0325465 | 1·7806E-09 | 2·72285 | 0·454146 | 2·028E-09 | 0·5599874 | 4 |
| *GPX1* | 3 | cg07274523 | 49395745 | *GPX1* | 3 | ENSG00000233276 | 49395321 | rs34293138 | 3 | 49579017 | C | T | 0·333333 | -0·109836 | 0·00901078 | 3·5402E-34 | -0·688309 | 0·0337708 | 2·4281E-92 | 0·159574 | 0·0152537 | 1·3007E-25 | 0·03209629 | 20 |
| *LDHD* | 16 | cg07320140 | 75150611 | *LDHD* | 16 | ENSG00000166816 | 75148213 | rs9927029 | 16 | 75172340 | A | G | 0·406953 | -0·0907657 | 0·0080566 | 1·9315E-29 | 0·219939 | 0·0331289 | 3·1604E-11 | -0·412686 | 0·0721521 | 1·0674E-08 | 0·4403728 | 15 |
| *SLC25A45* | 11 | cg07321092 | 65144724 | *MRPL49* | 11 | ENSG00000149792 | 64892249 | rs1149569 | 11 | 64913165 | G | A | 0·0593047 | -0·0925271 | 0·0168415 | 3·9293E-08 | -0·627891 | 0·063704 | 6·4329E-23 | 0·147362 | 0·0307077 | 1·5958E-06 | 0·550472 | 5 |
| *ACSF3* | 16 | cg07356861 | 89184285 | *ACSF3* | 16 | ENSG00000176715 | 89188518 | rs7204124 | 16 | 89179567 | A | G | 0·392638 | -0·126983 | 0·00925114 | 7·0748E-43 | 0·216354 | 0·032968 | 5·2896E-11 | -0·586922 | 0·0991312 | 3·2065E-09 | 0·0164679 | 10 |
| *RHOT2* | 16 | cg07375207 | 717419 | *MCRIP2* | 16 | ENSG00000172366 | 695143 | rs3743909 | 16 | 716428 | A | G | 0·374233 | -0·0612611 | 0·00938551 | 6·7011E-11 | 0·328787 | 0·0338109 | 2·3754E-22 | -0·186325 | 0·0343802 | 5·9757E-08 | 0·1904771 | 3 |
| *SUCLG2* | 3 | cg07438401 | 67698057 | *SUCLG2* | 3 | ENSG00000172340 | 67557961 | rs6786211 | 3 | 67703360 | T | A | 0·355828 | -0·222745 | 0·00816319 | 6·132E-164 | 0·251908 | 0·0332067 | 3·2977E-14 | -0·884232 | 0·120981 | 2·6942E-13 | 0·1020638 | 19 |
| *BID* | 22 | cg07473201 | 18231406 | *BID* | 22 | ENSG00000015475 | 18237221 | rs2895951 | 22 | 18232368 | A | G | 0·45092 | 0·0776434 | 0·00804778 | 5·0222E-22 | 0·240266 | 0·0320053 | 6·0465E-14 | 0·323156 | 0·0545433 | 3·1275E-09 | 0·02048652 | 11 |
| *SPG7* | 16 | cg07474682 | 89573562 | *ACSF3* | 16 | ENSG00000176715 | 89188518 | rs57696383 | 16 | 89572665 | G | A | 0·530675 | -0·0394084 | 0·00795679 | 7·3153E-07 | -0·330211 | 0·0324102 | 2·2323E-24 | 0·119343 | 0·0267923 | 8·4137E-06 | 0·03435032 | 20 |
| *TK2* | 16 | cg07512993 | 66582592 | *TK2* | 16 | ENSG00000166548 | 66564176 | rs7193473 | 16 | 66600479 | A | G | 0·135992 | 0·696058 | 0·011434 | 0 | -0·554844 | 0·0491035 | 1·32E-29 | -1·25451 | 0·11292 | 1·1251E-28 | 0·2830356 | 20 |
| *ALAS1* | 3 | cg07598930 | 52233294 | *ALAS1* | 3 | ENSG00000023330 | 52240222 | rs614288 | 3 | 52220203 | T | C | 0·464213 | -0·0785405 | 0·00802613 | 1·2982E-22 | 0·258845 | 0·0322313 | 9·6802E-16 | -0·303427 | 0·0488773 | 5·3689E-10 | 0·4893538 | 20 |
| *ALAS1* | 3 | cg07598930 | 52233294 | *GLYCTK* | 3 | ENSG00000168237 | 52325188 | rs614288 | 3 | 52220203 | T | C | 0·464213 | 0·310673 | 0·00781649 | 0 | 0·258845 | 0·0322313 | 9·6802E-16 | 1·20023 | 0·152472 | 3·4967E-15 | 0·02078697 | 20 |
| *SLC25A46* | 5 | cg07611177 | 110000000 | *SLC25A46* | 5 | ENSG00000164209 | 110087347 | rs6886061 | 5 | 109996467 | A | G | 0·201431 | -0·148706 | 0·0099713 | 2·6966E-50 | -0·250612 | 0·0435188 | 8·4761E-09 | 0·593371 | 0·110454 | 7·7821E-08 | 0·10941 | 7 |
| *NDUFA13* | 19 | cg07624705 | 19626525 | *SLC25A42* | 19 | ENSG00000181035 | 19199252 | rs61744761 | 19 | 19656615 | T | C | 0·166667 | -0·0564005 | 0·0116636 | 1·3274E-06 | -0·459726 | 0·042246 | 1·4026E-27 | 0·122683 | 0·0277628 | 9·9173E-06 | 0·1356844 | 11 |
| *NDUFA13* | 19 | cg07624705 | 19626525 | *NDUFA13* | 19 | ENSG00000186010 | 19635415 | rs61744761 | 19 | 19656615 | T | C | 0·166667 | 0·178241 | 0·0126846 | 7·5101E-45 | -0·459726 | 0·042246 | 1·4026E-27 | -0·387711 | 0·045063 | 7·714E-18 | 0·3127615 | 20 |
| *GFM1* | 3 | cg07654934 | 158000000 | *GFM1* | 3 | ENSG00000168827 | 158386215 | rs7624771 | 3 | 158335409 | T | C | 0·218814 | 0·0885709 | 0·0096453 | 4·2002E-20 | -0·285026 | 0·0404176 | 1·7633E-12 | -0·310747 | 0·0555596 | 2·2313E-08 | 0·02255216 | 11 |
| *CYB5B* | 16 | cg07707379 | 69458383 | *CYB5B* | 16 | ENSG00000103018 | 69479298 | rs153053 | 16 | 69446319 | A | G | 0·157464 | 0·137266 | 0·0105558 | 1·1641E-38 | -0·285583 | 0·0424991 | 1·8204E-11 | -0·480652 | 0·0805141 | 2·3756E-09 | 0·0309095 | 5 |
| *CYB5B* | 16 | cg07713807 | 69458177 | *PDF* | 16 | ENSG00000258429 | 69363511 | rs12927133 | 16 | 69438091 | T | G | 0·0879346 | -0·142294 | 0·0151939 | 7·5906E-21 | 0·311096 | 0·0549201 | 1·4744E-08 | -0·457396 | 0·094369 | 1·2541E-06 | 0·5460652 | 3 |
| *MRPS33* | 7 | cg07735777 | 141000000 | *NDUFB2* | 7 | ENSG00000090266 | 140406583 | rs73498965 | 7 | 140755848 | A | G | 0·0562372 | -0·30296 | 0·016264 | 1·9193E-77 | 0·397256 | 0·0720985 | 3·5903E-08 | -0·762632 | 0·144339 | 1·2666E-07 | 0·03728216 | 7 |
| *MRPS33* | 7 | cg07735777 | 141000000 | *ADCK2* | 7 | ENSG00000133597 | 140384507 | rs73498965 | 7 | 140755848 | A | G | 0·0562372 | 0·215456 | 0·0162532 | 4·1542E-40 | 0·397256 | 0·0720985 | 3·5903E-08 | 0·542361 | 0·106598 | 3·6204E-07 | 0·01179605 | 7 |
| *CAT* | 11 | cg07768201 | 34460336 | *CAT* | 11 | ENSG00000121691 | 34477040 | rs11604331 | 11 | 34460769 | G | A | 0·351738 | 0·678855 | 0·00748166 | 0 | -0·184682 | 0·033838 | 4·8198E-08 | -3·67581 | 0·674709 | 5·0943E-08 | 0·7533985 | 11 |
| *SPG7* | 16 | cg07778534 | 89601834 | *ACSF3* | 16 | ENSG00000176715 | 89188518 | rs57696383 | 16 | 89572665 | G | A | 0·530675 | -0·0394084 | 0·00795679 | 7·3153E-07 | 0·296248 | 0·032745 | 1·4677E-19 | -0·133025 | 0·0306199 | 1·3966E-05 | 0·1282733 | 12 |
| *NAGS* | 17 | cg07803108 | 42081927 | *NAGS* | 17 | ENSG00000161653 | 42084172 | rs186636 | 17 | 42085972 | C | T | 0·360941 | -0·0560292 | 0·00830629 | 1·5261E-11 | -0·253905 | 0·0348658 | 3·2805E-13 | 0·22067 | 0·0445918 | 7·4723E-07 | 0·4469583 | 15 |
| *ISCA1* | 9 | cg07824177 | 88896457 | *ISCA1* | 9 | ENSG00000135070 | 88888568 | rs1547629 | 9 | 88870898 | C | T | 0·368098 | 0·505449 | 0·0130295 | 0 | 0·317556 | 0·0339727 | 8·9836E-21 | 1·59168 | 0·175155 | 1·0152E-19 | 0·01051763 | 17 |
| *MRPS21* | 1 | cg07843065 | 150000000 | *TARS2* | 1 | ENSG00000143374 | 150469982 | rs4926419 | 1 | 150264714 | T | C | 0·45092 | -0·0838823 | 0·00798864 | 8·6197E-26 | -0·232032 | 0·0327993 | 1·502E-12 | 0·361512 | 0·061618 | 4·438E-09 | 0·6804736 | 17 |
| *MRPS21* | 1 | cg07843065 | 150000000 | *MCL1* | 1 | ENSG00000143384 | 150549549 | rs4926419 | 1 | 150264714 | T | C | 0·45092 | 0·0780574 | 0·00801789 | 2·1298E-22 | -0·232032 | 0·0327993 | 1·502E-12 | -0·336408 | 0·0587826 | 1·0471E-08 | 0·2823422 | 18 |
| *MRPS21* | 1 | cg07843065 | 150000000 | *ADAMTSL4-AS1* | 1 | ENSG00000143384 | 150549549 | rs4926419 | 1 | 150264714 | T | C | 0·45092 | 0·0780574 | 0·00801789 | 2·1298E-22 | -0·232032 | 0·0327993 | 1·502E-12 | -0·336408 | 0·0587826 | 1·0471E-08 | 0·2823422 | 18 |
| *SLC25A10* | 17 | cg07845392 | 79678158 | *MRPL12* | 17 | ENSG00000262814 | 79672471 | rs62077189 | 17 | 79673088 | A | G | 0·201431 | -0·124953 | 0·0122129 | 1·4373E-24 | -0·462593 | 0·0438281 | 4·8312E-26 | 0·270114 | 0·036769 | 2·0383E-13 | 0·01136503 | 14 |
| *VARS2* | 6 | cg07906263 | 30893133 | *MRPS18B* | 6 | ENSG00000204568 | 30589829 | rs28732099 | 6 | 31095892 | A | G | 0·0511247 | -0·136222 | 0·0290539 | 2·7508E-06 | -1·11982 | 0·0659361 | 1·0894E-64 | 0·121646 | 0·0269157 | 6·1975E-06 | 0·03506231 | 7 |
| *ACSL1* | 4 | cg07942479 | 186000000 | *ACSL1* | 4 | ENSG00000151726 | 185712360 | rs12645247 | 4 | 185749420 | C | T | 0·435583 | -0·0446019 | 0·00803261 | 2·8145E-08 | 0·270092 | 0·0324961 | 9·4474E-17 | -0·165136 | 0·0357664 | 3·8919E-06 | 0·128585 | 7 |
| *TSTD1* | 1 | cg07967677 | 161000000 | *PPOX* | 1 | ENSG00000143224 | 161142001 | rs4396136 | 1 | 161022380 | G | A | 0·209611 | -0·0701041 | 0·00943528 | 1·086E-13 | -0·256609 | 0·0408868 | 3·472E-10 | 0·273194 | 0·0569805 | 1·6306E-06 | 0·3689048 | 12 |
| *COX6B2* | 19 | cg08024114 | 55865076 | *COX6B2* | 19 | ENSG00000160471 | 55863428 | rs11084396 | 19 | 55865527 | C | T | 0·457055 | -0·0855448 | 0·00959345 | 4·7909E-19 | 0·347141 | 0·0312138 | 9·8715E-29 | -0·246427 | 0·0354217 | 3·4775E-12 | 0·02177628 | 8 |
| *MRPL16* | 11 | cg08066376 | 59577361 | *MRPL16* | 11 | ENSG00000166902 | 59575976 | rs11230085 | 11 | 59578322 | A | G | 0·0838446 | -0·102641 | 0·0161284 | 1·9657E-10 | 1·01685 | 0·0606265 | 3·8877E-63 | -0·10094 | 0·0169645 | 2·6801E-09 | 0·7459918 | 7 |
| *ACAT1* | 11 | cg08152564 | 108000000 | *ACAT1* | 11 | ENSG00000075239 | 108005373 | rs10890813 | 11 | 107987784 | A | G | 0·336401 | -0·27642 | 0·0123635 | 1·014E-110 | 0·219439 | 0·0350476 | 3·8211E-10 | -1·25967 | 0·208927 | 1·6477E-09 | 0·01369934 | 7 |
| *MRPL55* | 1 | cg08158976 | 228000000 | *GUK1* | 1 | ENSG00000143774 | 228332174 | rs1620734 | 1 | 228297613 | G | A | 0·0613497 | -0·134101 | 0·0213652 | 3·4606E-10 | 0·524005 | 0·0593225 | 1·0176E-18 | -0·255915 | 0·0500182 | 3·1134E-07 | 0·01222273 | 16 |
| *MRPL55* | 1 | cg08158976 | 228000000 | *COQ8A* | 1 | ENSG00000163050 | 227130241 | rs80096292 | 1 | 228125436 | T | G | 0·0705521 | -0·155135 | 0·0155088 | 1·4785E-23 | 0·38234 | 0·0547304 | 2·8307E-12 | -0·405751 | 0·0708437 | 1·0197E-08 | 0·01200607 | 7 |
| *PARS2* | 1 | cg08168431 | 55230707 | *PARS2* | 1 | ENSG00000162396 | 55226379 | rs1180942 | 1 | 55231955 | T | C | 0·423313 | 0·28385 | 0·00787526 | 1·759E-284 | -0·390012 | 0·0323144 | 1·5344E-33 | -0·727798 | 0·0635926 | 2·4996E-30 | 0·06688135 | 20 |
| *PDE2A* | 11 | cg08182160 | 72385307 | *PDE2A* | 11 | ENSG00000186642 | 72336410 | rs932074 | 11 | 72365663 | C | G | 0·484663 | -0·0576574 | 0·00795318 | 4·18E-13 | -0·20063 | 0·0319217 | 3·2773E-10 | 0·287382 | 0·0605157 | 2·0455E-06 | 0·01535747 | 10 |
| *BCL2* | 18 | cg08223235 | 60903834 | *BCL2* | 18 | ENSG00000171791 | 60888970 | rs3810027 | 18 | 60903978 | G | C | 0·345603 | 0·0494444 | 0·00836566 | 3·4128E-09 | -0·277025 | 0·0340318 | 3·9465E-16 | -0·178484 | 0·0373188 | 1·7298E-06 | 0·05226414 | 6 |
| *BCL2L1* | 20 | cg08257293 | 30308440 | *BCL2L1* | 20 | ENSG00000171552 | 30282023 | rs6058460 | 20 | 30363071 | A | G | 0·233129 | -0·0657921 | 0·00965713 | 9·5717E-12 | 0·443691 | 0·0402636 | 3·0721E-28 | -0·148284 | 0·0255892 | 6·8411E-09 | 0·06235446 | 20 |
| *NSUN4* | 1 | cg08259313 | 46806025 | *NSUN4* | 1 | ENSG00000117481 | 46818336 | rs6662982 | 1 | 46877180 | A | G | 0·286299 | 0·604985 | 0·00797711 | 0 | 0·310969 | 0·0353937 | 1·5497E-18 | 1·94548 | 0·222911 | 2·6005E-18 | 0·1713156 | 11 |
| *ABCB6* | 2 | cg08290072 | 220000000 | *CYP27A1* | 2 | ENSG00000135929 | 219663244 | rs3731894 | 2 | 220136371 | T | C | 0·0787321 | -0·0795348 | 0·0148535 | 8·5746E-08 | 0·489464 | 0·0528249 | 1·9366E-20 | -0·162494 | 0·0350494 | 3·5498E-06 | 0·2051079 | 3 |
| *ACSF3* | 16 | cg08316009 | 89165765 | *ACSF3* | 16 | ENSG00000176715 | 89188518 | rs12445614 | 16 | 89166832 | G | A | 0·185072 | 0·0536914 | 0·010029 | 8·6221E-08 | 0·305978 | 0·0407853 | 6·2775E-14 | 0·175475 | 0·0402668 | 1·3138E-05 | 0·01916282 | 17 |
| *NDUFA13* | 19 | cg08331981 | 19626599 | *NDUFA13* | 19 | ENSG00000186010 | 19635415 | rs8102502 | 19 | 19652746 | C | T | 0·176892 | 0·162259 | 0·0116565 | 4·789E-44 | -0·35623 | 0·0411097 | 4·5016E-18 | -0·455489 | 0·0619173 | 1·8891E-13 | 0·1227691 | 20 |
| *AGPAT4* | 6 | cg08362144 | 162000000 | *AGPAT4* | 6 | ENSG00000026652 | 161623052 | rs56073227 | 6 | 161556411 | A | T | 0·187117 | -0·0620314 | 0·0102984 | 1·7079E-09 | 0·285985 | 0·0402816 | 1·2508E-12 | -0·216904 | 0·0472243 | 4·3677E-06 | 0·3717461 | 5 |
| *TMEM70* | 8 | cg08526319 | 74888271 | *TMEM70* | 8 | ENSG00000175606 | 74889845 | rs7838336 | 8 | 74896677 | C | T | 0·167689 | -0·201713 | 0·0107111 | 4·1132E-79 | -0·491738 | 0·0433918 | 9·0579E-30 | 0·410204 | 0·0422456 | 2·7338E-22 | 0·1711311 | 18 |
| *ALDH4A1* | 1 | cg08538976 | 19229774 | *ALDH4A1* | 1 | ENSG00000159423 | 19213600 | rs9426727 | 1 | 19214597 | G | A | 0·0408998 | -0·373365 | 0·0276513 | 1·5095E-41 | -0·556442 | 0·0921917 | 1·5827E-09 | 0·670986 | 0·121771 | 3·5832E-08 | 0·03542339 | 3 |
| *NDUFS2* | 1 | cg08545169 | 161000000 | *NIT1* | 1 | ENSG00000158793 | 161091555 | rs4656994 | 1 | 161179877 | A | G | 0·207566 | -0·0614935 | 0·00972906 | 2·6055E-10 | 0·552394 | 0·0381257 | 1·427E-47 | -0·111322 | 0·0192155 | 6·9001E-09 | 0·09132597 | 4 |
| *RHOT2* | 16 | cg08616681 | 718778 | *RHOT2* | 16 | ENSG00000140983 | 721130 | rs11866949 | 16 | 716273 | C | T | 0·118609 | -0·107766 | 0·0139832 | 1·2901E-14 | 0·91787 | 0·0492794 | 1·9842E-77 | -0·117409 | 0·0164871 | 1·0693E-12 | 0·04248581 | 8 |
| *RHOT2* | 16 | cg08616681 | 718778 | *MCRIP2* | 16 | ENSG00000172366 | 695143 | rs3177338 | 16 | 722331 | T | C | 0·381391 | -0·0530969 | 0·00947855 | 2·1214E-08 | 0·37319 | 0·0338759 | 3·1855E-28 | -0·142278 | 0·0284938 | 5·9352E-07 | 0·1144395 | 5 |
| *ALDH7A1* | 5 | cg08675664 | 126000000 | *ALDH7A1* | 5 | ENSG00000164904 | 125904321 | rs62391691 | 5 | 126078913 | A | G | 0·0552147 | -0·27652 | 0·0264147 | 1·2067E-25 | 0·784048 | 0·0727431 | 4·3579E-27 | -0·352683 | 0·0469652 | 5·9376E-14 | 0·0848961 | 8 |
| *CYB5R3* | 22 | cg08690876 | 43040721 | *SMDT1* | 22 | ENSG00000183172 | 42497900 | rs8190441 | 22 | 43024701 | A | G | 0·0204499 | -0·225673 | 0·0298825 | 4·2862E-14 | 0·769105 | 0·11071 | 3·7309E-12 | -0·293423 | 0·0573898 | 3·1741E-07 | 0·330236 | 3 |
| *COMT* | 22 | cg08730070 | 19938378 | *RTL10* | 22 | ENSG00000215012 | 19838040 | rs5748484 | 22 | 19922585 | T | C | 0·281186 | -0·0767259 | 0·00986905 | 7·5806E-15 | -0·225367 | 0·0367475 | 8·6313E-10 | 0·340449 | 0·0707054 | 1·4718E-06 | 0·09993069 | 5 |
| *PRDX5* | 11 | cg08790734 | 64084599 | *BAD* | 11 | ENSG00000002330 | 64044739 | rs750830 | 11 | 64163446 | A | G | 0·253579 | 0·0531705 | 0·00907068 | 4·5788E-09 | 0·287889 | 0·0370567 | 7·9186E-15 | 0·184691 | 0·0394701 | 2·8791E-06 | 0·2845247 | 9 |
| *QDPR* | 4 | cg08808571 | 17513338 | *QDPR* | 4 | ENSG00000151552 | 17487870 | rs7661303 | 4 | 17518920 | T | C | 0·238241 | -0·911957 | 0·00775417 | 0 | 0·827179 | 0·0344444 | 1·943E-127 | -1·10249 | 0·0468559 | 2·042E-122 | 0·0280459 | 20 |
| *ACACB* | 12 | cg08866695 | 110000000 | *USP30* | 12 | ENSG00000135093 | 109493362 | rs2541886 | 12 | 109537564 | T | G | 0·50409 | -0·0647801 | 0·00799517 | 5·3885E-16 | 0·277191 | 0·0326731 | 2·18E-17 | -0·233702 | 0·0398846 | 4·644E-09 | 0·01290749 | 4 |
| *COX4I2* | 20 | cg08918020 | 30225706 | *BCL2L1* | 20 | ENSG00000171552 | 30282023 | rs6088856 | 20 | 30226543 | G | C | 0·206544 | -0·0721911 | 0·0104079 | 4·0279E-12 | -0·408921 | 0·0417097 | 1·0824E-22 | 0·17654 | 0·0311779 | 1·493E-08 | 0·3746711 | 20 |
| *MRPL28* | 16 | cg08923669 | 420230 | *ANTKMT* | 16 | ENSG00000103254 | 771591 | rs34626626 | 16 | 438352 | T | G | 0·432515 | 0·0456384 | 0·00938021 | 1·1423E-06 | -0·357297 | 0·0324112 | 2·9311E-28 | -0·127732 | 0·0286965 | 8·5412E-06 | 0·05013178 | 13 |
| *BIK* | 22 | cg08954856 | 43506306 | *BIK* | 22 | ENSG00000100290 | 43516236 | rs4988374 | 22 | 43506753 | A | C | 0·266871 | -0·146138 | 0·00980213 | 2·889E-50 | -0·228535 | 0·0375076 | 1·1079E-09 | 0·639456 | 0·113375 | 1·6987E-08 | 0·1423172 | 5 |
| *AGPAT4* | 6 | cg09043403 | 162000000 | *AGPAT4* | 6 | ENSG00000026652 | 161623052 | rs12524665 | 6 | 161616293 | T | C | 0·110429 | 0·172798 | 0·0125298 | 2·8873E-43 | -0·314785 | 0·0475841 | 3·707E-11 | -0·54894 | 0·0920328 | 2·4522E-09 | 0·1079188 | 13 |
| *MRPS10* | 6 | cg09055489 | 42175018 | *MRPS10* | 6 | ENSG00000048544 | 42180071 | rs3199638 | 6 | 42175221 | A | G | 0·360941 | -0·156788 | 0·0123443 | 5·823E-37 | 0·221046 | 0·0317241 | 3·2203E-12 | -0·7093 | 0·116109 | 1·0032E-09 | 0·02783488 | 8 |
| *NDUFS2* | 1 | cg09070378 | 161000000 | *NIT1* | 1 | ENSG00000158793 | 161091555 | rs4379692 | 1 | 161186313 | T | C | 0·320041 | -0·0469062 | 0·00865013 | 5·8738E-08 | 0·265016 | 0·0344882 | 1·5389E-14 | -0·176994 | 0·0399488 | 9·4006E-06 | 0·0232954 | 5 |
| *NDUFS2* | 1 | cg09070378 | 161000000 | *TSTD1* | 1 | ENSG00000215845 | 161008100 | rs4379692 | 1 | 161186313 | T | C | 0·320041 | 0·0829971 | 0·0105845 | 4·4555E-15 | 0·265016 | 0·0344882 | 1·5389E-14 | 0·313178 | 0·0570627 | 4·0581E-08 | 0·0850058 | 6 |
| *NTHL1* | 16 | cg09123625 | 2097210 | *NTHL1* | 16 | ENSG00000065057 | 2093841 | rs2369090 | 16 | 2091600 | T | C | 0·160532 | 0·100676 | 0·0130359 | 1·1362E-14 | -0·287797 | 0·045532 | 2·6028E-10 | -0·349816 | 0·0715166 | 1·0012E-06 | 0·250693 | 8 |
| *ECHDC2* | 1 | cg09128567 | 53387203 | *SCP2* | 1 | ENSG00000116171 | 53455138 | rs11206056 | 1 | 53444836 | C | T | 0·371166 | -0·666727 | 0·00739435 | 0 | 0·329292 | 0·0316935 | 2·7567E-25 | -2·02473 | 0·196164 | 5·6282E-25 | 0·1115432 | 20 |
| *ECHDC2* | 1 | cg09128567 | 53387203 | *ECHDC2* | 1 | ENSG00000121310 | 53377270 | rs11206056 | 1 | 53444836 | C | T | 0·371166 | -0·524836 | 0·00775177 | 0 | 0·329292 | 0·0316935 | 2·7567E-25 | -1·59383 | 0·155198 | 9·6531E-25 | 0·05657747 | 20 |
| *MACROD1* | 11 | cg09182533 | 63775071 | *COX8A* | 11 | ENSG00000176340 | 63743047 | rs11605797 | 11 | 63743420 | G | A | 0·396728 | -0·161765 | 0·0153192 | 4·5861E-26 | -0·283005 | 0·0325344 | 3·359E-18 | 0·571598 | 0·0851357 | 1·8941E-11 | 0·2124586 | 20 |
| *TK2* | 16 | cg09238666 | 66584358 | *DUS2* | 16 | ENSG00000167264 | 68067436 | rs2242140 | 16 | 67421798 | G | A | 0·0398773 | 0·436997 | 0·0266549 | 2·0901E-60 | -0·472219 | 0·0837088 | 1·6886E-08 | -0·925412 | 0·173485 | 9·5934E-08 | 0·3982504 | 18 |
| *HAGH* | 16 | cg09274113 | 1875967 | *MRPS34* | 16 | ENSG00000074071 | 1822523 | rs11863172 | 16 | 1909083 | G | C | 0·192229 | 0·0882188 | 0·010286 | 9·7719E-18 | -0·243492 | 0·0398598 | 1·0044E-09 | -0·362307 | 0·0728161 | 6·5032E-07 | 0·04737065 | 20 |
| *FAHD1* | 16 | cg09274113 | 1875967 | *MRPS34* | 16 | ENSG00000074071 | 1822523 | rs11863172 | 16 | 1909083 | G | C | 0·192229 | 0·0882188 | 0·010286 | 9·7719E-18 | -0·243492 | 0·0398598 | 1·0044E-09 | -0·362307 | 0·0728161 | 6·5032E-07 | 0·04737065 | 20 |
| *METTL8* | 2 | cg09281979 | 172000000 | *METAP1D* | 2 | ENSG00000172878 | 172905824 | rs79670648 | 2 | 172359279 | C | T | 0·0674847 | -0·0982614 | 0·0182955 | 7·8388E-08 | -0·458037 | 0·064891 | 1·6824E-12 | 0·214527 | 0·0501913 | 1·9183E-05 | 0·4408729 | 3 |
| *NAGS* | 17 | cg09300856 | 42083686 | *NAGS* | 17 | ENSG00000161653 | 42084172 | rs228771 | 17 | 42084097 | G | C | 0·433538 | 0·0544441 | 0·00832912 | 6·2933E-11 | -0·229483 | 0·0323628 | 1·3319E-12 | -0·237247 | 0·0493635 | 1·5389E-06 | 0·2230861 | 4 |
| *MRPL43* | 10 | cg09319822 | 103000000 | *MRPL43* | 10 | ENSG00000055950 | 102738243 | rs112309064 | 10 | 102751706 | A | G | 0·185072 | -0·424099 | 0·00957577 | 0 | 0·278134 | 0·0414685 | 1·9852E-11 | -1·5248 | 0·229933 | 3·323E-11 | 0·2107825 | 7 |
| *MRPL43* | 10 | cg09319822 | 103000000 | *TWNK* | 10 | ENSG00000107815 | 102750641 | rs112309064 | 10 | 102751706 | A | G | 0·185072 | -0·133653 | 0·00980839 | 2·7901E-42 | 0·278134 | 0·0414685 | 1·9852E-11 | -0·480535 | 0·0798543 | 1·7697E-09 | 0·6096112 | 7 |
| *ACCS* | 11 | cg09339476 | 44087396 | *ACCS* | 11 | ENSG00000110455 | 44096623 | rs2074038 | 11 | 44087989 | T | G | 0·119632 | 1·31917 | 0·0131363 | 0 | -0·842381 | 0·0516605 | 8·9307E-60 | -1·566 | 0·0972956 | 2·7523E-58 | 0·01414468 | 20 |
| *MACROD1* | 11 | cg09375205 | 63885665 | *BAD* | 11 | ENSG00000002330 | 64044739 | rs677447 | 11 | 63885704 | C | T | 0·303681 | 0·0483554 | 0·00838863 | 8·1949E-09 | -0·244149 | 0·0342419 | 1·0029E-12 | -0·198057 | 0·0441827 | 7·371E-06 | 0·09065416 | 16 |
| *MRPL55* | 1 | cg09462576 | 228000000 | *GUK1* | 1 | ENSG00000143774 | 228332174 | rs1620734 | 1 | 228297613 | G | A | 0·0613497 | -0·134101 | 0·0213652 | 3·4606E-10 | 0·563469 | 0·0594696 | 2·6698E-21 | -0·237992 | 0·0454823 | 1·6712E-07 | 0·01464335 | 17 |
| *GTPBP3* | 19 | cg09793057 | 17448797 | *MRPL34* | 19 | ENSG00000130312 | 17410535 | rs117419016 | 19 | 17443453 | A | G | 0·0235174 | -0·232127 | 0·0289276 | 1·0202E-15 | 1·01947 | 0·118834 | 9·5738E-18 | -0·227694 | 0·0388532 | 4·6187E-09 | 0·1651574 | 4 |
| *NDUFA7* | 19 | cg09799873 | 8385500 | *NDUFA7* | 19 | ENSG00000267855 | 8379885 | rs4147647 | 19 | 8386059 | G | T | 0·0429448 | 0·15793 | 0·0212924 | 1·1962E-13 | -1·15537 | 0·0907163 | 3·7262E-37 | -0·136692 | 0·0213265 | 1·4601E-10 | 0·9971442 | 3 |
| *PGAM5* | 12 | cg09802308 | 133000000 | *PGAM5* | 12 | ENSG00000247077 | 133293316 | rs11147050 | 12 | 133335243 | C | G | 0·41411 | 0·1092 | 0·00820645 | 2·1191E-40 | -0·185046 | 0·0314958 | 4·2219E-09 | -0·590124 | 0·109797 | 7·672E-08 | 0·6431265 | 15 |
| *MFN2* | 1 | cg09820729 | 12039712 | *MFN2* | 1 | ENSG00000116688 | 12056904 | rs2236053 | 1 | 12040203 | G | A | 0·441718 | -0·519687 | 0·00742857 | 0 | -0·217641 | 0·0328778 | 3·5992E-11 | 2·38782 | 0·362325 | 4·3904E-11 | 0·06361389 | 17 |
| *FAHD1* | 16 | cg09830162 | 1889614 | *NDUFB10* | 16 | ENSG00000140990 | 2010742 | rs2754184 | 16 | 2007391 | T | G | 0·157464 | 0·117625 | 0·0132089 | 5·3386E-19 | -0·257864 | 0·0452525 | 1·2098E-08 | -0·456151 | 0·0950363 | 1·5886E-06 | 0·2927977 | 6 |
| *TXNRD1* | 12 | cg09884423 | 105000000 | *NT5DC3* | 12 | ENSG00000111696 | 104199603 | rs4592489 | 12 | 104619699 | C | T | 0·221881 | -0·0859238 | 0·00936601 | 4·5579E-20 | 0·208844 | 0·0381329 | 4·3322E-08 | -0·411426 | 0·0874906 | 2·5698E-06 | 0·06998762 | 7 |
| *TXNRD1* | 12 | cg09884423 | 105000000 | *TXNRD1* | 12 | ENSG00000198431 | 104676809 | rs4592489 | 12 | 104619699 | C | T | 0·221881 | 0·157004 | 0·0093689 | 4·95E-63 | 0·208844 | 0·0381329 | 4·3322E-08 | 0·751776 | 0·144412 | 1·9317E-07 | 0·08254981 | 10 |
| *LDHD* | 16 | cg09899215 | 75150799 | *LDHD* | 16 | ENSG00000166816 | 75148213 | rs9927029 | 16 | 75172340 | A | G | 0·406953 | -0·0907657 | 0·0080566 | 1·9315E-29 | 0·210748 | 0·0333712 | 2·6971E-10 | -0·430684 | 0·0781812 | 3·6131E-08 | 0·7865067 | 14 |
| *GRPEL1* | 4 | cg09948192 | 7069943 | *GRPEL1* | 4 | ENSG00000109519 | 7065278 | rs6825954 | 4 | 7074226 | T | C | 0·219836 | -0·09469 | 0·0101108 | 7·5906E-21 | -0·393737 | 0·0382044 | 6·6162E-25 | 0·24049 | 0·0346978 | 4·1786E-12 | 0·05568608 | 20 |
| *ACSM3* | 16 | cg10078415 | 20775011 | *ACSM3* | 16 | ENSG00000005187 | 20715234 | rs3815020 | 16 | 20916757 | G | T | 0·134969 | -0·207936 | 0·0111474 | 1·185E-77 | 0·421705 | 0·0461408 | 6·2732E-20 | -0·493084 | 0·0600786 | 2·262E-16 | 0·4258292 | 20 |
| *ACSM3* | 16 | cg10078415 | 20775011 | *ACSM1* | 16 | ENSG00000166743 | 20672385 | rs3815020 | 16 | 20916757 | G | T | 0·134969 | 0·145759 | 0·0112165 | 1·3061E-38 | 0·421705 | 0·0461408 | 6·2732E-20 | 0·345642 | 0·0462352 | 7·6771E-14 | 0·01752256 | 19 |
| *SPG7* | 16 | cg10091053 | 89603535 | *SPG7* | 16 | ENSG00000197912 | 89590750 | rs8059968 | 16 | 89589125 | A | G | 0·490798 | 0·188755 | 0·00791724 | 1·255E-125 | -0·379629 | 0·0323736 | 9·3249E-32 | -0·497209 | 0·0472519 | 6·8031E-26 | 0·1083875 | 20 |
| *SPG7* | 16 | cg10091053 | 89603535 | *LOC101930112* | 16 | ENSG00000197912 | 89590750 | rs8059968 | 16 | 89589125 | A | G | 0·490798 | 0·188755 | 0·00791724 | 1·255E-125 | -0·379629 | 0·0323736 | 9·3249E-32 | -0·497209 | 0·0472519 | 6·8031E-26 | 0·1083875 | 20 |
| *MRPL38* | 17 | cg10108402 | 73901394 | *MRPL38* | 17 | ENSG00000204316 | 73900311 | rs55872768 | 17 | 73892414 | G | T | 0·350716 | -0·0522975 | 0·00859987 | 1·1929E-09 | -0·447388 | 0·0348028 | 8·0687E-38 | 0·116895 | 0·0212648 | 3·8603E-08 | 0·02301825 | 17 |
| *LAP3* | 4 | cg10189774 | 17578691 | *LAP3* | 4 | ENSG00000002549 | 17594205 | rs60777810 | 4 | 17643170 | T | C | 0·377301 | 0·716629 | 0·00754168 | 0 | -0·214314 | 0·0318216 | 1·641E-11 | -3·34383 | 0·497742 | 1·8424E-11 | 0·09612384 | 18 |
| *NSUN4* | 1 | cg10215817 | 46806136 | *NSUN4* | 1 | ENSG00000117481 | 46818336 | rs5013329 | 1 | 46815091 | T | C | 0·277096 | 0·634139 | 0·00796633 | 0 | 0·211409 | 0·0343858 | 7·8396E-10 | 2·99958 | 0·489338 | 8·7943E-10 | 0·0307169 | 10 |
| *IDH3B* | 20 | cg10475341 | 2644958 | *IDH3B* | 20 | ENSG00000101365 | 2641953 | rs55856704 | 20 | 2654966 | T | C | 0·111452 | 0·087902 | 0·0133128 | 4·0346E-11 | -0·33553 | 0·0513562 | 6·4299E-11 | -0·26198 | 0·0564107 | 3·4148E-06 | 0·01188145 | 3 |
| *BCO2* | 11 | cg10503334 | 112000000 | *BCO2* | 11 | ENSG00000197580 | 112070806 | rs2115763 | 11 | 112051169 | T | A | 0·335378 | 0·133938 | 0·00851276 | 8·8723E-56 | 0·232286 | 0·0329026 | 1·6673E-12 | 0·576608 | 0·0895199 | 1·186E-10 | 0·1273654 | 6 |
| *BIK* | 22 | cg10513709 | 43506327 | *TSPO* | 22 | ENSG00000100300 | 43553384 | rs5759168 | 22 | 43500435 | G | A | 0·162577 | -0·0861 | 0·0109347 | 3·4348E-15 | 0·254742 | 0·0462416 | 3·6101E-08 | -0·337989 | 0·0748779 | 6·3656E-06 | 0·2430321 | 4 |
| *ECHDC2* | 1 | cg10554624 | 53386618 | *SCP2* | 1 | ENSG00000116171 | 53455138 | rs11206055 | 1 | 53436632 | T | A | 0·372188 | -0·667608 | 0·00783258 | 0 | 0·296858 | 0·0333385 | 5·3696E-19 | -2·24891 | 0·253938 | 8·2816E-19 | 0·08328993 | 20 |
| *MRPS6* | 21 | cg10599571 | 35445161 | *ATP5PO* | 21 | ENSG00000241837 | 35282020 | rs2834330 | 21 | 35366219 | G | A | 0·163599 | -0·107271 | 0·010833 | 4·0722E-23 | 0·245845 | 0·0430554 | 1·13E-08 | -0·436336 | 0·0882109 | 7·5557E-07 | 0·07683103 | 5 |
| *MRPS6* | 21 | cg10599571 | 35445161 | *MRPS6* | 21 | ENSG00000243927 | 35480429 | rs2834330 | 21 | 35366219 | G | A | 0·163599 | -0·103591 | 0·0107975 | 8·4735E-22 | 0·245845 | 0·0430554 | 1·13E-08 | -0·421367 | 0·0858758 | 9·262E-07 | 0·01013693 | 6 |
| *MACROD1* | 11 | cg10612274 | 63827432 | *BAD* | 11 | ENSG00000002330 | 64044739 | rs11601522 | 11 | 63958409 | T | C | 0·302658 | 0·0533878 | 0·00921464 | 6·8811E-09 | 0·306379 | 0·0334089 | 4·7047E-20 | 0·174254 | 0·0355755 | 9·6745E-07 | 0·1099596 | 14 |
| *MACROD1* | 11 | cg10612274 | 63827432 | *DNAJC4* | 11 | ENSG00000110011 | 63999754 | rs11601522 | 11 | 63958409 | T | C | 0·302658 | -0·0576877 | 0·00921323 | 3·8154E-10 | 0·306379 | 0·0334089 | 4·7047E-20 | -0·188289 | 0·0364121 | 2·3279E-07 | 0·4633468 | 10 |
| *VARS2* | 6 | cg10661769 | 30881484 | *VARS2* | 6 | ENSG00000137411 | 30885127 | rs2233956 | 6 | 31081205 | C | T | 0·149284 | -0·19038 | 0·0165833 | 1·659E-30 | 0·225469 | 0·0386473 | 5·4107E-09 | -0·844373 | 0·162349 | 1·9824E-07 | 0·01391189 | 16 |
| *C12orf65* | 12 | cg10672416 | 124000000 | *MTRFR* | 12 | ENSG00000130921 | 123729984 | rs10744151 | 12 | 123723735 | G | C | 0·280164 | 0·0662361 | 0·00917016 | 5·0853E-13 | -0·415061 | 0·0371907 | 6·3751E-29 | -0·159582 | 0·026317 | 1·3293E-09 | 0·01225969 | 17 |
| *PICK1* | 22 | cg10778862 | 38453075 | *GCAT* | 22 | ENSG00000100116 | 38208547 | rs7289911 | 22 | 38468632 | A | G | 0·0194274 | 0·370576 | 0·0243348 | 2·2983E-52 | 0·594071 | 0·105868 | 2·0065E-08 | 0·623791 | 0·118471 | 1·3993E-07 | 0·119214 | 10 |
| *PICK1* | 22 | cg10778862 | 38453075 | *PICK1* | 22 | ENSG00000100151 | 38462013 | rs7289911 | 22 | 38468632 | A | G | 0·0194274 | 0·414843 | 0·0243956 | 7·5664E-65 | 0·594071 | 0·105868 | 2·0065E-08 | 0·698305 | 0·131044 | 9·8866E-08 | 0·8705461 | 9 |
| *LYRM7* | 5 | cg10838757 | 131000000 | *HINT1* | 5 | ENSG00000169567 | 130501074 | rs74905787 | 5 | 131403532 | A | G | 0·0327198 | -0·684778 | 0·0418461 | 3·4453E-60 | 0·611303 | 0·0924022 | 3·6986E-11 | -1·12019 | 0·182638 | 8·6014E-10 | 0·466435 | 20 |
| *GATM* | 15 | cg11032707 | 45671246 | *GATM* | 15 | ENSG00000171766 | 45673869 | rs2172874 | 15 | 45665452 | G | T | 0·280164 | -0·576243 | 0·00825717 | 0 | 0·548726 | 0·0354891 | 6·2788E-54 | -1·05015 | 0·0695657 | 1·7268E-51 | 0·01543855 | 20 |
| *PARS2* | 1 | cg11084629 | 55230770 | *PARS2* | 1 | ENSG00000162396 | 55226379 | rs1180959 | 1 | 55240823 | C | A | 0·433538 | 0·274744 | 0·00787335 | 8·718E-267 | -0·282433 | 0·0321745 | 1·6618E-18 | -0·972776 | 0·11427 | 1·6959E-17 | 0·5822372 | 14 |
| *DMPK* | 19 | cg11101109 | 46274119 | *DMPK* | 19 | ENSG00000104936 | 46279392 | rs4802276 | 19 | 46260748 | C | T | 0·468303 | 0·279289 | 0·00780585 | 2·306E-280 | 0·49705 | 0·0302109 | 8·0238E-61 | 0·561893 | 0·0375898 | 1·6042E-50 | 0·0171115 | 20 |
| *NT5DC2* | 3 | cg11126497 | 52558566 | *ALAS1* | 3 | ENSG00000023330 | 52240222 | rs1010553 | 3 | 52540773 | C | T | 0·464213 | -0·0514362 | 0·00797387 | 1·1141E-10 | 0·191896 | 0·0327592 | 4·691E-09 | -0·268042 | 0·06181 | 1·4474E-05 | 0·113071 | 18 |
| *NT5DC2* | 3 | cg11126497 | 52558566 | *GLYCTK* | 3 | ENSG00000168237 | 52325188 | rs1010553 | 3 | 52540773 | C | T | 0·464213 | 0·295488 | 0·00777711 | 0 | 0·191896 | 0·0327592 | 4·691E-09 | 1·53983 | 0·265976 | 7·0648E-09 | 0·1802013 | 19 |
| *NT5DC2* | 3 | cg11126497 | 52558566 | *NT5DC2* | 3 | ENSG00000168268 | 52563728 | rs1010553 | 3 | 52540773 | C | T | 0·464213 | 0·299292 | 0·00777251 | 0 | 0·191896 | 0·0327592 | 4·691E-09 | 1·55966 | 0·269317 | 6·9905E-09 | 0·02400527 | 19 |
| *ECSIT* | 19 | cg11224407 | 11617055 | *ECSIT* | 19 | ENSG00000130159 | 11628360 | rs4520944 | 19 | 11614987 | A | C | 0·0388548 | -0·204354 | 0·022609 | 1·5869E-19 | -0·593269 | 0·0904312 | 5·3647E-11 | 0·344454 | 0·0648772 | 1·1003E-07 | 0·8783316 | 10 |
| *NIF3L1* | 2 | cg11229815 | 202000000 | *NIF3L1* | 2 | ENSG00000196290 | 201761352 | rs7559150 | 2 | 201754063 | C | T | 0·259714 | -0·101529 | 0·0093767 | 2·5419E-27 | -0·327748 | 0·0389668 | 4·0668E-17 | 0·309778 | 0·0466366 | 3·0866E-11 | 0·438531 | 16 |
| *MTX3* | 5 | cg11264499 | 79286765 | *MTX3* | 5 | ENSG00000177034 | 79281333 | rs12054701 | 5 | 79295326 | A | T | 0·176892 | 0·159213 | 0·0192509 | 1·3351E-16 | 0·243497 | 0·0438986 | 2·9094E-08 | 0·65386 | 0·141938 | 4·0918E-06 | 0·1422821 | 4 |
| *SERHL2* | 22 | cg11341438 | 42949526 | *SMDT1* | 22 | ENSG00000183172 | 42497900 | rs137116 | 22 | 43011674 | C | T | 0·326176 | -0·0462219 | 0·00923109 | 5·5228E-07 | 0·338567 | 0·0341863 | 4·0163E-23 | -0·136522 | 0·0305519 | 7·8759E-06 | 0·01097251 | 4 |
| *SERHL2* | 22 | cg11341438 | 42949526 | *SERHL2* | 22 | ENSG00000183569 | 42960005 | rs137100 | 22 | 42990074 | T | C | 0·226994 | -0·254702 | 0·0169098 | 2·8621E-51 | 0·403757 | 0·0362567 | 8·3731E-29 | -0·63083 | 0·0704483 | 3·4124E-19 | 0·01542743 | 20 |
| *GCAT* | 22 | cg11368946 | 38204391 | *GCAT* | 22 | ENSG00000100116 | 38208547 | rs5750499 | 22 | 38210424 | C | T | 0·123722 | 0·758217 | 0·0110513 | 0 | 0·318547 | 0·0459978 | 4·3517E-12 | 2·38024 | 0·34545 | 5·5694E-12 | 0·2176315 | 10 |
| *LIPT2* | 11 | cg11417029 | 74204987 | *LIPT2* | 11 | ENSG00000175536 | 74203767 | rs7121457 | 11 | 74205004 | G | A | 0·398773 | 0·0705529 | 0·00905825 | 6·7649E-15 | -0·247385 | 0·0319643 | 9·987E-15 | -0·285195 | 0·0519483 | 4·02E-08 | 0·7976756 | 8 |
| *ATPAF1* | 1 | cg11528978 | 47134349 | *ATPAF1* | 1 | ENSG00000123472 | 47118974 | rs35050319 | 1 | 47103886 | A | C | 0·212679 | 0·142137 | 0·0102935 | 2·268E-43 | -0·34245 | 0·0367049 | 1·0606E-20 | -0·415059 | 0·0536902 | 1·0702E-14 | 0·05543896 | 4 |
| *SLC25A10* | 17 | cg11609462 | 79682615 | *MRPL12* | 17 | ENSG00000262814 | 79672471 | rs62080220 | 17 | 79644695 | A | G | 0·166667 | -0·133387 | 0·0132237 | 6·3068E-24 | 0·373261 | 0·0459607 | 4·6118E-16 | -0·357356 | 0·0564916 | 2·5188E-10 | 0·3903943 | 20 |
| *MACROD1* | 11 | cg11642412 | 63768412 | *COX8A* | 11 | ENSG00000176340 | 63743047 | rs1055933 | 11 | 63724175 | A | G | 0·279141 | -0·129493 | 0·0169259 | 2·0004E-14 | 0·201984 | 0·0358433 | 1·7486E-08 | -0·641105 | 0·141299 | 5·6995E-06 | 0·0273886 | 7 |
| *TXNRD1* | 12 | cg11659394 | 105000000 | *TXNRD1* | 12 | ENSG00000198431 | 104676809 | rs12811618 | 12 | 104677589 | G | A | 0·447853 | 0·19289 | 0·00800706 | 3·182E-128 | -0·373529 | 0·032924 | 7·8361E-30 | -0·516399 | 0·0503121 | 1·0248E-24 | 0·01535655 | 20 |
| *MACROD1* | 11 | cg11659663 | 63768794 | *COX8A* | 11 | ENSG00000176340 | 63743047 | rs4980512 | 11 | 63716716 | G | C | 0·440695 | -0·159472 | 0·0148806 | 8·4889E-27 | 0·278887 | 0·0322882 | 5·7487E-18 | -0·571816 | 0·0850275 | 1·7551E-11 | 0·6939426 | 20 |
| *COX4I2* | 20 | cg11704513 | 30225851 | *BCL2L1* | 20 | ENSG00000171552 | 30282023 | rs6060474 | 20 | 30236557 | G | A | 0·204499 | -0·0664355 | 0·010022 | 3·3797E-11 | -0·465386 | 0·0417371 | 7·1272E-29 | 0·142754 | 0·0250529 | 1·2116E-08 | 0·02036997 | 20 |
| *AGMAT* | 1 | cg11706911 | 15910989 | *AGMAT* | 1 | ENSG00000116771 | 15905226 | rs61103742 | 1 | 15909153 | C | A | 0·232106 | 0·0549794 | 0·00908691 | 1·4449E-09 | 0·304764 | 0·0356852 | 1·3383E-17 | 0·1804 | 0·0365404 | 7·9333E-07 | 0·09665844 | 6 |
| *TSTD1* | 1 | cg11731300 | 161000000 | *TSTD1* | 1 | ENSG00000215845 | 161008100 | rs10908821 | 1 | 161008535 | G | C | 0·128834 | 0·173743 | 0·0144754 | 3·4431E-33 | -0·368857 | 0·0528097 | 2·8559E-12 | -0·471031 | 0·0780256 | 1·5713E-09 | 0·01199373 | 12 |
| *NDUFS2* | 1 | cg11801851 | 161000000 | *TSTD1* | 1 | ENSG00000215845 | 161008100 | rs4656994 | 1 | 161179877 | A | G | 0·207566 | 0·0725486 | 0·0119135 | 1·1319E-09 | -0·249913 | 0·0388031 | 1·1905E-10 | -0·290295 | 0·0656055 | 9·6503E-06 | 0·05626223 | 3 |
| *COX4I2* | 20 | cg12033622 | 30225573 | *BCL2L1* | 20 | ENSG00000171552 | 30282023 | rs6060425 | 20 | 30224355 | T | C | 0·207566 | -0·0709434 | 0·0104785 | 1·2843E-11 | -0·310149 | 0·0404239 | 1·6879E-14 | 0·22874 | 0·0450586 | 3·8446E-07 | 0·31475 | 20 |
| *NDUFS8* | 11 | cg12070987 | 67804055 | *MRPL21* | 11 | ENSG00000197345 | 68665023 | rs4930227 | 11 | 67840160 | A | T | 0·378323 | 0·0569659 | 0·00833737 | 8·3389E-12 | 0·20391 | 0·0339093 | 1·8169E-09 | 0·279368 | 0·0618878 | 6·3586E-06 | 0·7823186 | 4 |
| *TMEM177* | 2 | cg12108912 | 120000000 | *TMEM177* | 2 | ENSG00000144120 | 120440413 | rs6708153 | 2 | 120462953 | A | G | 0·143149 | -0·195526 | 0·0120577 | 3·8997E-59 | -0·286346 | 0·0442082 | 9·3428E-11 | 0·682831 | 0·113519 | 1·7976E-09 | 0·2802997 | 11 |
| *TSFM* | 12 | cg12113251 | 58176230 | *ATP23* | 12 | ENSG00000166896 | 58343188 | rs35091613 | 12 | 58270680 | T | C | 0·0552147 | -0·441932 | 0·0275742 | 8·2783E-58 | 0·450733 | 0·0803063 | 1·9923E-08 | -0·980474 | 0·185092 | 1·1757E-07 | 0·1595464 | 7 |
| *SUCLG1* | 2 | cg12158889 | 84663044 | *SUCLG1* | 2 | ENSG00000163541 | 84668908 | rs6719880 | 2 | 84673355 | A | G | 0·0562372 | 0·18838 | 0·019172 | 8·7179E-23 | -0·476708 | 0·0747889 | 1·841E-10 | -0·395169 | 0·0738986 | 8·9205E-08 | 0·2015413 | 9 |
| *D2HGDH* | 2 | cg12163823 | 243000000 | *D2HGDH* | 2 | ENSG00000180902 | 242691112 | rs13421651 | 2 | 242469811 | T | C | 0·154397 | -0·133442 | 0·0116137 | 1·4811E-30 | 0·370892 | 0·0450448 | 1·8129E-16 | -0·359787 | 0·0537573 | 2·1895E-11 | 0·04300033 | 18 |
| *SLC25A1* | 22 | cg12167239 | 19166808 | *SLC25A1* | 22 | ENSG00000100075 | 19164719 | rs5746711 | 22 | 19264625 | G | A | 0·00613497 | -0·415876 | 0·0751221 | 3·0946E-08 | 1·38865 | 0·175783 | 2·7934E-15 | -0·299482 | 0·0660582 | 5·7983E-06 | 0·03679196 | 12 |
| *HPDL* | 1 | cg12178578 | 45792714 | *NSUN4* | 1 | ENSG00000117481 | 46818336 | rs61782881 | 1 | 46516038 | C | A | 0·0276074 | -0·237036 | 0·0250508 | 3·0153E-21 | 0·480679 | 0·080175 | 2·0301E-09 | -0·493127 | 0·097372 | 4·0976E-07 | 0·4172719 | 6 |
| *HPDL* | 1 | cg12178578 | 45792714 | *MUTYH* | 1 | ENSG00000132781 | 45800488 | rs140723719 | 1 | 45306595 | A | G | 0·0408998 | -0·187759 | 0·0227135 | 1·3806E-16 | 0·516566 | 0·0731251 | 1·6162E-12 | -0·363475 | 0·067682 | 7·8588E-08 | 0·2188316 | 13 |
| *SCP2* | 1 | cg12220753 | 53393030 | *SCP2* | 1 | ENSG00000116171 | 53455138 | rs56222568 | 1 | 53432786 | C | T | 0·351738 | -0·721517 | 0·00745799 | 0 | 0·289942 | 0·03094 | 7·1779E-21 | -2·48849 | 0·266792 | 1·0845E-20 | 0·4714132 | 20 |
| *SCP2* | 1 | cg12220753 | 53393030 | *ECHDC2* | 1 | ENSG00000121310 | 53377270 | rs56222568 | 1 | 53432786 | C | T | 0·351738 | -0·56109 | 0·00788291 | 0 | 0·289942 | 0·03094 | 7·1779E-21 | -1·93518 | 0·208287 | 1·5295E-20 | 0·06639788 | 20 |
| *DCAKD* | 17 | cg12223258 | 43117026 | *DCAKD* | 17 | ENSG00000172992 | 43119590 | rs59246405 | 17 | 43123625 | T | C | 0·359918 | 0·369662 | 0·00845953 | 0 | -0·213712 | 0·0321217 | 2·8677E-11 | -1·72972 | 0·262979 | 4·7872E-11 | 0·01811925 | 17 |
| *ARF5* | 7 | cg12288726 | 127000000 | *ARF5* | 7 | ENSG00000004059 | 127230079 | rs712699 | 7 | 127250597 | G | A | 0·220859 | -0·0612407 | 0·00931417 | 4·8653E-11 | -0·239965 | 0·040264 | 2·5257E-09 | 0·255207 | 0·057795 | 1·0067E-05 | 0·4462892 | 4 |
| *SND1* | 7 | cg12345672 | 128000000 | *ARF5* | 7 | ENSG00000004059 | 127230079 | rs3823994 | 7 | 127669857 | T | A | 0·271984 | 0·0517651 | 0·00881348 | 4·2695E-09 | -0·355015 | 0·0368541 | 5·8021E-22 | -0·145811 | 0·0290763 | 5·3097E-07 | 0·1120589 | 20 |
| *SDHAF1* | 19 | cg12416033 | 36484731 | *SDHAF1* | 19 | ENSG00000205138 | 36486655 | rs7251575 | 19 | 36488986 | C | T | 0·230061 | -0·236708 | 0·0105753 | 5·738E-111 | -0·356269 | 0·0422242 | 3·2403E-17 | 0·664408 | 0·084153 | 2·8977E-15 | 0·8986492 | 8 |
| *TSTD1* | 1 | cg12426196 | 161000000 | *PPOX* | 1 | ENSG00000143224 | 161142001 | rs2990706 | 1 | 160973693 | T | G | 0·210634 | -0·0717661 | 0·00969576 | 1·3435E-13 | -0·234142 | 0·0400296 | 4·9387E-09 | 0·306507 | 0·0667882 | 4·4487E-06 | 0·5622477 | 9 |
| *VARS2* | 6 | cg12433575 | 30881464 | *VARS2* | 6 | ENSG00000137411 | 30885127 | rs2242955 | 6 | 31474280 | A | G | 0·108384 | 0·106374 | 0·0177396 | 2·0174E-09 | -0·352132 | 0·0532648 | 3·818E-11 | -0·302086 | 0·0680141 | 8·9328E-06 | 0·01118508 | 8 |
| *ATAD3B* | 1 | cg12439834 | 1410849 | *MRPL20* | 1 | ENSG00000242485 | 1339990 | rs143070056 | 1 | 1294375 | T | G | 0·0173824 | 0·302448 | 0·0493841 | 9·1026E-10 | -0·892736 | 0·130503 | 7·8792E-12 | -0·338788 | 0·0742481 | 5·0449E-06 | 0·6005748 | 5 |
| *PRDX5* | 11 | cg12453748 | 64085041 | *PRDX5* | 11 | ENSG00000126432 | 64087421 | rs4930698 | 11 | 64085063 | C | G | 0·0633947 | -0·622443 | 0·0159941 | 0 | 0·389994 | 0·0694003 | 1·9152E-08 | -1·59603 | 0·286963 | 2·67E-08 | 0·9736674 | 4 |
| *HEBP1* | 12 | cg12479139 | 13154094 | *HEBP1* | 12 | ENSG00000013583 | 13140502 | rs67498669 | 12 | 13154077 | C | T | 0·458078 | -0·274437 | 0·00860333 | 2·763E-223 | -0·195763 | 0·0308245 | 2·1408E-10 | 1·40188 | 0·225071 | 4·7051E-10 | 0·0281605 | 14 |
| *PRODH* | 22 | cg12512005 | 18924935 | *SLC25A1* | 22 | ENSG00000100075 | 19164719 | rs9604911 | 22 | 18924956 | T | G | 0·246421 | 0·0574391 | 0·0113894 | 4·5781E-07 | 0·339503 | 0·0388615 | 2·4102E-18 | 0·169186 | 0·0387358 | 1·2557E-05 | 0·01018529 | 6 |
| *ABCB8* | 7 | cg12513221 | 151000000 | *FASTK* | 7 | ENSG00000164896 | 150775832 | rs2069456 | 7 | 150752608 | G | T | 0·249489 | -0·0662525 | 0·0107822 | 8·0165E-10 | 0·354934 | 0·0375845 | 3·6011E-21 | -0·186661 | 0·0362425 | 2·6001E-07 | 0·06616289 | 17 |
| *TIMM8B* | 11 | cg12532791 | 112000000 | *BCO2* | 11 | ENSG00000197580 | 112070806 | rs12790529 | 11 | 111974107 | T | C | 0·325153 | 0·0551429 | 0·00831417 | 3·3027E-11 | 0·207126 | 0·0336824 | 7·7788E-10 | 0·266229 | 0·059039 | 6·5021E-06 | 0·1937071 | 8 |
| *SDHD* | 11 | cg12532791 | 112000000 | *BCO2* | 11 | ENSG00000197580 | 112070806 | rs12790529 | 11 | 111974107 | T | C | 0·325153 | 0·0551429 | 0·00831417 | 3·3027E-11 | 0·207126 | 0·0336824 | 7·7788E-10 | 0·266229 | 0·059039 | 6·5021E-06 | 0·1937071 | 8 |
| *SAMM50* | 22 | cg12712746 | 44351370 | *SAMM50* | 22 | ENSG00000100347 | 44378856 | rs2294920 | 22 | 44351351 | C | T | 0·132924 | -0·843045 | 0·0112827 | 0 | 0·433676 | 0·0502341 | 5·9711E-18 | -1·94395 | 0·226672 | 9·8189E-18 | 0·9255705 | 8 |
| *SPATA20* | 17 | cg12765405 | 48628455 | *ACSF2* | 17 | ENSG00000167107 | 48527862 | rs739924 | 17 | 48631607 | C | T | 0·277096 | -0·112523 | 0·00930634 | 1·1784E-33 | 0·332381 | 0·0365616 | 9·8152E-20 | -0·338536 | 0·0465904 | 3·6968E-13 | 0·03582273 | 8 |
| *GUK1* | 1 | cg12796841 | 228000000 | *GUK1* | 1 | ENSG00000143774 | 228332174 | rs10916271 | 1 | 228372546 | T | C | 0·368098 | -0·0702266 | 0·0121135 | 6·7351E-09 | -0·301003 | 0·0315104 | 1·2661E-21 | 0·233309 | 0·0470752 | 7·1929E-07 | 0·08890747 | 14 |
| *GUK1* | 1 | cg12796841 | 228000000 | *MRPL55* | 1 | ENSG00000162910 | 228295696 | rs10916271 | 1 | 228372546 | T | C | 0·368098 | -0·147896 | 0·00813671 | 7·9378E-74 | -0·301003 | 0·0315104 | 1·2661E-21 | 0·491344 | 0·0581069 | 2·7702E-17 | 0·01557424 | 20 |
| *GPD2* | 2 | cg12807206 | 157000000 | *GPD2* | 2 | ENSG00000115159 | 157381024 | rs298309 | 2 | 157288358 | C | T | 0·449898 | 0·387214 | 0·00781419 | 0 | -0·334386 | 0·0324477 | 6·6634E-25 | -1·15799 | 0·114771 | 6·1487E-24 | 0·402057 | 20 |
| *MSRA* | 8 | cg12810313 | 10192475 | *MSRA* | 8 | ENSG00000175806 | 10099089 | rs11249985 | 8 | 10171312 | A | G | 0·320041 | 0·330184 | 0·00884369 | 4·351E-301 | -0·256197 | 0·0345015 | 1·1221E-13 | -1·28879 | 0·176958 | 3·2642E-13 | 0·1641905 | 20 |
| *BCL2L1* | 20 | cg12873919 | 30309627 | *BCL2L1* | 20 | ENSG00000171552 | 30282023 | rs1075698 | 20 | 30387992 | C | G | 0·232106 | -0·0668016 | 0·00966319 | 4·7451E-12 | 0·328922 | 0·0404874 | 4·5087E-16 | -0·203093 | 0·038575 | 1·4029E-07 | 0·07868744 | 20 |
| *MACROD1* | 11 | cg12874671 | 63772824 | *COX8A* | 11 | ENSG00000176340 | 63743047 | rs320156 | 11 | 63768397 | C | T | 0·444785 | -0·163508 | 0·0149366 | 6·8837E-28 | 0·601681 | 0·0304656 | 8·1029E-87 | -0·271752 | 0·0283832 | 1·0246E-21 | 0·8625421 | 20 |
| *GFM1* | 3 | cg12896421 | 158000000 | *GFM1* | 3 | ENSG00000168827 | 158386215 | rs7628497 | 3 | 158384068 | G | A | 0·217791 | 0·0902732 | 0·00959088 | 4·8494E-21 | -0·261958 | 0·0404515 | 9·4274E-11 | -0·344609 | 0·0645929 | 9·5492E-08 | 0·1307676 | 9 |
| *TSTD1* | 1 | cg12898512 | 161000000 | *PPOX* | 1 | ENSG00000143224 | 161142001 | rs6662441 | 1 | 161030974 | T | G | 0·208589 | -0·0772154 | 0·00964542 | 1·1908E-15 | -0·39249 | 0·0399032 | 7·8746E-23 | 0·196732 | 0·0316855 | 5·3359E-10 | 0·02935671 | 13 |
| *MRPL23* | 11 | cg12921275 | 1969496 | *MRPL23* | 11 | ENSG00000214026 | 1987130 | rs75676658 | 11 | 1988737 | G | A | 0·0235174 | -0·217599 | 0·0330808 | 4·7746E-11 | -0·665819 | 0·0887116 | 6·1224E-14 | 0·326814 | 0·066065 | 7·5423E-07 | 0·01187666 | 8 |
| *MRPL23* | 11 | cg12921275 | 1969496 | *LOC107987373* | 11 | ENSG00000214026 | 1987130 | rs75676658 | 11 | 1988737 | G | A | 0·0235174 | -0·217599 | 0·0330808 | 4·7746E-11 | -0·665819 | 0·0887116 | 6·1224E-14 | 0·326814 | 0·066065 | 7·5423E-07 | 0·01187666 | 8 |
| *BLOC1S1* | 12 | cg12926596 | 56111944 | *SUOX* | 12 | ENSG00000139531 | 56395694 | rs73119231 | 12 | 56126308 | A | G | 0·0378323 | 0·184304 | 0·0325844 | 1·5476E-08 | -0·967974 | 0·116972 | 1·2817E-16 | -0·190402 | 0·0407745 | 3·0174E-06 | 0·04770426 | 7 |
| *CYP24A1* | 20 | cg12978433 | 52789956 | *CYP24A1* | 20 | ENSG00000019186 | 52780250 | rs2585421 | 20 | 52807023 | C | T | 0·142127 | -0·0743721 | 0·0117169 | 2·1898E-10 | 0·292391 | 0·0447998 | 6·7271E-11 | -0·254358 | 0·0558989 | 5·3562E-06 | 0·0431615 | 12 |
| *NMNAT3* | 3 | cg12987403 | 139000000 | *NMNAT3* | 3 | ENSG00000163864 | 139337940 | rs6785099 | 3 | 139392951 | C | T | 0·256646 | 0·40811 | 0·00906406 | 0 | 0·228131 | 0·0382016 | 2·3469E-09 | 1·78893 | 0·302187 | 3·2208E-09 | 0·6133804 | 4 |
| *MRPL12* | 17 | cg12996171 | 79674399 | *MRPL12* | 17 | ENSG00000262814 | 79672471 | rs57366978 | 17 | 79658280 | A | G | 0·175869 | 0·0850614 | 0·0123471 | 5·6107E-12 | 0·646146 | 0·040717 | 1·0358E-56 | 0·131644 | 0·0208318 | 2·6258E-10 | 0·04349975 | 11 |
| *SCP2* | 1 | cg13078931 | 53397458 | *SCP2* | 1 | ENSG00000116171 | 53455138 | rs550561 | 1 | 53375134 | C | T | 0·369121 | -0·673068 | 0·00734106 | 0 | -0·307279 | 0·0322236 | 1·4867E-21 | 2·19041 | 0·230942 | 2·4312E-21 | 0·01498712 | 20 |
| *SCP2* | 1 | cg13078931 | 53397458 | *ECHDC2* | 1 | ENSG00000121310 | 53377270 | rs550561 | 1 | 53375134 | C | T | 0·369121 | -0·537478 | 0·00768713 | 0 | -0·307279 | 0·0322236 | 1·4867E-21 | 1·74915 | 0·185127 | 3·4413E-21 | 0·03830457 | 20 |
| *COX16* | 14 | cg13099429 | 70827109 | *COX16* | 14 | ENSG00000133983 | 70809123 | rs35697534 | 14 | 70764266 | C | T | 0·106339 | -0·146089 | 0·0144405 | 4·6633E-24 | -0·375959 | 0·0566255 | 3·1499E-11 | 0·388577 | 0·0700044 | 2·8444E-08 | 0·02616736 | 20 |
| *NUDT19* | 19 | cg13157960 | 33183277 | *NUDT19* | 19 | ENSG00000213965 | 33193784 | rs7246451 | 19 | 33209614 | G | C | 0·177914 | -0·0975112 | 0·0118511 | 1·9037E-16 | 0·492072 | 0·0400046 | 9·0137E-35 | -0·198165 | 0·0289757 | 7·9758E-12 | 0·2596297 | 13 |
| *BNIP3L* | 8 | cg13160058 | 26243215 | *BNIP3L* | 8 | ENSG00000104765 | 26301783 | rs7818293 | 8 | 26264116 | A | C | 0·243354 | -0·05089 | 0·00977563 | 1·9316E-07 | 0·651434 | 0·036813 | 4·5191E-70 | -0·07812 | 0·0156422 | 5·9087E-07 | 0·2024725 | 5 |
| *MCL1* | 1 | cg13175981 | 151000000 | *MCL1* | 1 | ENSG00000143384 | 150549549 | rs11204679 | 1 | 150583670 | G | C | 0·474438 | 0·0982566 | 0·00802108 | 1·6841E-34 | -0·430974 | 0·0305392 | 3·1957E-45 | -0·227987 | 0·0246452 | 2·2283E-20 | 0·2202998 | 20 |
| *MCL1* | 1 | cg13175981 | 151000000 | *ADAMTSL4-AS1* | 1 | ENSG00000143384 | 150549549 | rs11204679 | 1 | 150583670 | G | C | 0·474438 | 0·0982566 | 0·00802108 | 1·6841E-34 | -0·430974 | 0·0305392 | 3·1957E-45 | -0·227987 | 0·0246452 | 2·2283E-20 | 0·2202998 | 20 |
| *OXA1L* | 14 | cg13182010 | 23235291 | *OXA1L* | 14 | ENSG00000155463 | 23238369 | rs35875924 | 14 | 23227561 | T | C | 0·476483 | -0·0872086 | 0·0141123 | 6·4264E-10 | -0·271784 | 0·0329142 | 1·4893E-16 | 0·320875 | 0·0648554 | 7·5158E-07 | 0·01218505 | 7 |
| *BAD* | 11 | cg13221074 | 64051814 | *PRDX5* | 11 | ENSG00000126432 | 64087421 | rs34882006 | 11 | 64051823 | T | C | 0·0306748 | 0·8351 | 0·0227144 | 6·642E-296 | 0·499328 | 0·0761547 | 5·499E-11 | 1·67245 | 0·259097 | 1·0828E-10 | 0·01934168 | 3 |
| *ALAS1* | 3 | cg13241645 | 52235959 | *ALAS1* | 3 | ENSG00000023330 | 52240222 | rs614288 | 3 | 52220203 | T | C | 0·464213 | -0·0785405 | 0·00802613 | 1·2982E-22 | 0·239176 | 0·0322418 | 1·1873E-13 | -0·32838 | 0·0555486 | 3·3886E-09 | 0·8519707 | 16 |
| *ATAD3A* | 1 | cg13321900 | 1455303 | *MRPL20* | 1 | ENSG00000242485 | 1339990 | rs3128342 | 1 | 1486834 | A | C | 0·458078 | 0·0768282 | 0·0118016 | 7·5151E-11 | 0·322251 | 0·0328286 | 9·5914E-23 | 0·238411 | 0·043944 | 5·7846E-08 | 0·2853222 | 10 |
| *ECHDC2* | 1 | cg13461509 | 53387576 | *SCP2* | 1 | ENSG00000116171 | 53455138 | rs12040433 | 1 | 53232766 | G | A | 0·337423 | -0·696558 | 0·00763783 | 0 | 0·356889 | 0·0335844 | 2·2404E-26 | -1·95175 | 0·184908 | 4·8049E-26 | 0·1914068 | 20 |
| *TSTD1* | 1 | cg13466383 | 161000000 | *TSTD1* | 1 | ENSG00000215845 | 161008100 | rs10908821 | 1 | 161008535 | G | C | 0·128834 | 0·173743 | 0·0144754 | 3·4431E-33 | -0·359345 | 0·0525583 | 8·0824E-12 | -0·483499 | 0·0813857 | 2·8357E-09 | 0·02084541 | 10 |
| *DNA2* | 10 | cg13484341 | 70232027 | *SLC25A16* | 10 | ENSG00000122912 | 70264876 | rs10998210 | 10 | 70237160 | C | T | 0·145194 | 0·585147 | 0·0104386 | 0 | -0·633474 | 0·0482162 | 1·9893E-39 | -0·923711 | 0·0722126 | 1·8278E-37 | 0·08666372 | 20 |
| *DNA2* | 10 | cg13484341 | 70232027 | *DNA2* | 10 | ENSG00000138346 | 70202850 | rs10998210 | 10 | 70237160 | C | T | 0·145194 | -0·247954 | 0·0118991 | 1·9585E-96 | -0·633474 | 0·0482162 | 1·9893E-39 | 0·391419 | 0·0352197 | 1·0767E-28 | 0·0115158 | 20 |
| *ECSIT* | 19 | cg13520520 | 11616957 | *ECSIT* | 19 | ENSG00000130159 | 11628360 | rs113259217 | 19 | 11642086 | T | C | 0·0388548 | -0·212241 | 0·0231785 | 5·3459E-20 | -1·19655 | 0·0931279 | 8·7747E-38 | 0·177377 | 0·0237871 | 8·8607E-14 | 0·06475316 | 20 |
| *MTHFD1L* | 6 | cg13602242 | 151000000 | *MTHFD1L* | 6 | ENSG00000120254 | 151304854 | rs9397361 | 6 | 151187349 | G | C | 0·444785 | 0·101121 | 0·012084 | 5·8505E-17 | -0·203034 | 0·0322538 | 3·0763E-10 | -0·49805 | 0·099006 | 4·8921E-07 | 0·04807304 | 6 |
| *PRDX2* | 19 | cg13618045 | 12913155 | *GCDH* | 19 | ENSG00000105607 | 13013430 | rs3786712 | 19 | 12923725 | A | G | 0·377301 | -0·277782 | 0·00877069 | 3·822E-220 | -0·209378 | 0·0348276 | 1·8344E-09 | 1·3267 | 0·224622 | 3·4973E-09 | 0·02546639 | 12 |
| *BAD* | 11 | cg13620770 | 64051738 | *PRDX5* | 11 | ENSG00000126432 | 64087421 | rs4672 | 11 | 64009879 | A | G | 0·0725971 | 0·792628 | 0·0164483 | 0 | 0·318262 | 0·0575608 | 3·218E-08 | 2·49049 | 0·453385 | 3·9493E-08 | 0·4245943 | 3 |
| *SCP2* | 1 | cg13685833 | 53393034 | *SCP2* | 1 | ENSG00000116171 | 53455138 | rs12040433 | 1 | 53232766 | G | A | 0·337423 | -0·696558 | 0·00763783 | 0 | 0·42126 | 0·0323123 | 7·5217E-39 | -1·65351 | 0·12812 | 4·1667E-38 | 0·04169786 | 20 |
| *TTC19* | 17 | cg13736128 | 15901788 | *TTC19* | 17 | ENSG00000011295 | 15925511 | rs62072427 | 17 | 15674784 | G | A | 0·281186 | 0·0580786 | 0·00963544 | 1·6641E-09 | -0·248807 | 0·0366578 | 1·1427E-11 | -0·233428 | 0·0517934 | 6·5778E-06 | 0·01696045 | 11 |
| *ACSF2* | 17 | cg13764778 | 48546503 | *RSAD1* | 17 | ENSG00000136444 | 48559748 | rs11869714 | 17 | 48587307 | G | A | 0·236196 | -0·200255 | 0·00972168 | 2·8132E-94 | 0·231235 | 0·0396961 | 5·7066E-09 | -0·866024 | 0·1545 | 2·0787E-08 | 0·1262477 | 6 |
| *LIPT1* | 2 | cg13789186 | 99771248 | *LIPT1* | 2 | ENSG00000144182 | 99775519 | rs13023501 | 2 | 99793070 | G | A | 0·387526 | -0·494035 | 0·0076599 | 0 | -0·377904 | 0·0331362 | 3·968E-30 | 1·3073 | 0·116408 | 2·8937E-29 | 0·1777698 | 20 |
| *NDUFAF1* | 15 | cg13897914 | 41695294 | *RMDN3* | 15 | ENSG00000137824 | 41038065 | rs11858278 | 15 | 41416276 | C | T | 0·325153 | 0·053496 | 0·00977631 | 4·4498E-08 | 0·245999 | 0·0348946 | 1·7922E-12 | 0·217464 | 0·0503081 | 1·5417E-05 | 0·07312268 | 16 |
| *TSTD1* | 1 | cg13906823 | 161000000 | *PPOX* | 1 | ENSG00000143224 | 161142001 | rs10797091 | 1 | 161027665 | A | G | 0·209611 | -0·0692632 | 0·00946943 | 2·5853E-13 | -0·224192 | 0·0409179 | 4·2757E-08 | 0·308946 | 0·0704521 | 1·1588E-05 | 0·2260954 | 7 |
| *TSTD1* | 1 | cg13906823 | 161000000 | *TSTD1* | 1 | ENSG00000215845 | 161008100 | rs10908821 | 1 | 161008535 | G | C | 0·128834 | 0·173743 | 0·0144754 | 3·4431E-33 | -0·362783 | 0·0526814 | 5·724E-12 | -0·478917 | 0·0801793 | 2·3282E-09 | 0·01011154 | 12 |
| *ACADS* | 12 | cg13914990 | 121000000 | *SIRT4* | 12 | ENSG00000089163 | 120745585 | rs55647329 | 12 | 121155966 | T | C | 0·153374 | 0·0558999 | 0·0116619 | 1·6398E-06 | 0·477704 | 0·0479249 | 2·1093E-23 | 0·117018 | 0·0270884 | 1·5613E-05 | 0·04877748 | 8 |
| *SPIRE1* | 18 | cg13924974 | 12656824 | *PRELID3A* | 18 | ENSG00000141391 | 12420066 | rs12959998 | 18 | 12485188 | T | C | 0·157464 | 0·099292 | 0·0120272 | 1·5114E-16 | -0·46605 | 0·0471157 | 4·5265E-23 | -0·21305 | 0·0336139 | 2·3252E-10 | 0·04051243 | 12 |
| *HPDL* | 1 | cg13951491 | 45793032 | *MUTYH* | 1 | ENSG00000132781 | 45800488 | rs3219487 | 1 | 45798555 | T | C | 0·0797546 | -0·25817 | 0·0141648 | 3·1977E-74 | 0·432258 | 0·0568294 | 2·8219E-14 | -0·597259 | 0·0850857 | 2·2266E-12 | 0·01516602 | 20 |
| *SND1* | 7 | cg13969327 | 128000000 | *ARF5* | 7 | ENSG00000004059 | 127230079 | rs327521 | 7 | 127249143 | G | A | 0·231084 | -0·0575513 | 0·00917942 | 3·6198E-10 | -0·287113 | 0·0394584 | 3·4298E-13 | 0·200448 | 0·0422026 | 2·0376E-06 | 0·637477 | 5 |
| *SLC25A25* | 9 | cg13983182 | 131000000 | *PTGES2* | 9 | ENSG00000148334 | 130886856 | rs10760536 | 9 | 130854984 | G | T | 0·135992 | -0·147932 | 0·0111495 | 3·5493E-40 | 0·408191 | 0·043133 | 2·9768E-21 | -0·362409 | 0·0470384 | 1·3132E-14 | 0·01225237 | 15 |
| *SLC25A25* | 9 | cg13983182 | 131000000 | *SLC25A25* | 9 | ENSG00000148339 | 130851002 | rs10760536 | 9 | 130854984 | G | T | 0·135992 | 0·163603 | 0·0112399 | 5·3813E-48 | 0·408191 | 0·043133 | 2·9768E-21 | 0·4008 | 0·0505165 | 2·1211E-15 | 0·02112432 | 15 |
| *BCL2L1* | 20 | cg13989999 | 30309717 | *BCL2L1* | 20 | ENSG00000171552 | 30282023 | rs8118150 | 20 | 30436088 | A | G | 0·240286 | -0·0663687 | 0·00966375 | 6·52E-12 | 0·272739 | 0·0402864 | 1·2878E-11 | -0·243341 | 0·050472 | 1·4261E-06 | 0·1954313 | 20 |
| *GFM1* | 3 | cg14019186 | 158000000 | *GFM1* | 3 | ENSG00000168827 | 158386215 | rs7628497 | 3 | 158384068 | G | A | 0·217791 | 0·0902732 | 0·00959088 | 4·8494E-21 | -0·246865 | 0·0404567 | 1·0478E-09 | -0·365678 | 0·0714196 | 3·0531E-07 | 0·07589225 | 6 |
| *PANK2* | 20 | cg14025831 | 3873404 | *PANK2* | 20 | ENSG00000125779 | 3888545 | rs115017522 | 20 | 3873195 | T | G | 0·0470348 | 0·144182 | 0·0183676 | 4·167E-15 | -0·47064 | 0·0803478 | 4·6979E-09 | -0·306353 | 0·0652569 | 2·6717E-06 | 0·5590588 | 3 |
| *DNLZ* | 9 | cg14035368 | 139000000 | *PMPCA* | 9 | ENSG00000165688 | 139311661 | rs11145750 | 9 | 139259249 | G | A | 0·433538 | 0·048566 | 0·00847219 | 9·9019E-09 | -0·261225 | 0·0331228 | 3·1061E-15 | -0·185916 | 0·0400948 | 3·5364E-06 | 0·2947845 | 11 |
| *SLC25A25* | 9 | cg14039237 | 131000000 | *PTGES2* | 9 | ENSG00000148334 | 130886856 | rs35830594 | 9 | 130835135 | G | A | 0·169734 | -0·130755 | 0·0103701 | 1·8885E-36 | -0·234508 | 0·0422824 | 2·9188E-08 | 0·557572 | 0·109827 | 3·8385E-07 | 0·1457428 | 11 |
| *SLC25A25* | 9 | cg14039237 | 131000000 | *SLC25A25* | 9 | ENSG00000148339 | 130851002 | rs35830594 | 9 | 130835135 | G | A | 0·169734 | 0·155146 | 0·0104489 | 7·1675E-50 | -0·234508 | 0·0422824 | 2·9188E-08 | -0·661581 | 0·127335 | 2·0406E-07 | 0·4189412 | 11 |
| *MACROD1* | 11 | cg14085017 | 63775226 | *COX8A* | 11 | ENSG00000176340 | 63743047 | rs11231663 | 11 | 63738769 | C | A | 0·396728 | -0·161047 | 0·0151825 | 2·753E-26 | -0·273804 | 0·0326945 | 5·5404E-17 | 0·588183 | 0·089485 | 4·9316E-11 | 0·4430727 | 20 |
| *AGPAT4* | 6 | cg14170337 | 162000000 | *AGPAT4* | 6 | ENSG00000026652 | 161623052 | rs150633624 | 6 | 161597476 | A | G | 0·193252 | 0·106371 | 0·0101499 | 1·0674E-25 | -0·222054 | 0·0403747 | 3·8016E-08 | -0·479032 | 0·0983648 | 1·1162E-06 | 0·05787296 | 10 |
| *CHCHD2* | 7 | cg14187409 | 56174376 | *CHCHD2* | 7 | ENSG00000106153 | 56171765 | rs6964563 | 7 | 55957708 | T | C | 0·156442 | -0·686841 | 0·0115443 | 0 | -0·290349 | 0·0402281 | 5·2925E-13 | 2·36557 | 0·330155 | 7·7764E-13 | 0·173905 | 20 |
| *CHCHD2* | 7 | cg14187409 | 56174376 | *NIPSNAP2* | 7 | ENSG00000146729 | 56043680 | rs6964563 | 7 | 55957708 | T | C | 0·156442 | -0·112594 | 0·010643 | 3·7212E-26 | -0·290349 | 0·0402281 | 5·2925E-13 | 0·387788 | 0·0650414 | 2·489E-09 | 0·128814 | 20 |
| *NDUFA13* | 19 | cg14188428 | 19625761 | *NDUFA13* | 19 | ENSG00000186010 | 19635415 | rs74950305 | 19 | 19639448 | T | C | 0·170757 | 0·174462 | 0·0126175 | 1·7518E-43 | 0·322755 | 0·0418582 | 1·2515E-14 | 0·54054 | 0·0802663 | 1·6468E-11 | 0·968774 | 20 |
| *CAT* | 11 | cg14316565 | 34460789 | *CAT* | 11 | ENSG00000121691 | 34477040 | rs35960263 | 11 | 34510012 | A | G | 0·400818 | 0·60539 | 0·00741092 | 0 | -0·195667 | 0·0321992 | 1·2263E-09 | -3·09398 | 0·510556 | 1·3611E-09 | 0·3638277 | 12 |
| *BAD* | 11 | cg14391469 | 64051725 | *PRDX5* | 11 | ENSG00000126432 | 64087421 | rs34882006 | 11 | 64051823 | T | C | 0·0306748 | 0·8351 | 0·0227144 | 6·642E-296 | 0·430855 | 0·075976 | 1·4202E-08 | 1·93824 | 0·345827 | 2·0867E-08 | 0·07850984 | 4 |
| *NIPSNAP3B* | 9 | cg14392031 | 108000000 | *NIPSNAP3A* | 9 | ENSG00000136783 | 107516186 | rs62569025 | 9 | 107409559 | T | C | 0·121677 | 0·325039 | 0·0130297 | 2·36E-137 | -0·365112 | 0·0504408 | 4·5392E-13 | -0·890245 | 0·128062 | 3·6093E-12 | 0·09058587 | 8 |
| *NIPSNAP3B* | 9 | cg14392031 | 108000000 | *NIPSNAP3B* | 9 | ENSG00000165028 | 107533088 | rs62569025 | 9 | 107409559 | T | C | 0·121677 | 0·272459 | 0·0128906 | 3·697E-99 | -0·365112 | 0·0504408 | 4·5392E-13 | -0·746234 | 0·108971 | 7·4899E-12 | 0·4919751 | 7 |
| *NDUFB6* | 9 | cg14472148 | 32573691 | *NDUFB6* | 9 | ENSG00000165264 | 32563078 | rs7874777 | 9 | 32565245 | G | C | 0·256646 | 0·084859 | 0·00947108 | 3·2527E-19 | 0·415305 | 0·037949 | 7·1184E-28 | 0·204329 | 0·0294733 | 4·1289E-12 | 0·01220796 | 13 |
| *GRHPR* | 9 | cg14519323 | 37432946 | *GRHPR* | 9 | ENSG00000137106 | 37429825 | rs59967810 | 9 | 37420311 | T | C | 0·349693 | 0·854447 | 0·00702188 | 0 | 0·27545 | 0·0342274 | 8·4401E-16 | 3·102 | 0·386297 | 9·739E-16 | 0·6207224 | 6 |
| *SPTLC2* | 14 | cg14544289 | 78051204 | *ALKBH1* | 14 | ENSG00000100601 | 78156555 | rs2272587 | 14 | 78023519 | C | G | 0·429448 | -0·04061 | 0·00804095 | 4·4089E-07 | -0·280324 | 0·0330044 | 2·0043E-17 | 0·144868 | 0·0333724 | 1·4186E-05 | 0·02107494 | 6 |
| *SLC25A25* | 9 | cg14554539 | 131000000 | *PTGES2* | 9 | ENSG00000148334 | 130886856 | rs7851605 | 9 | 130841917 | T | C | 0·138037 | -0·13871 | 0·0120314 | 9·4231E-31 | 0·276254 | 0·0451819 | 9·7004E-10 | -0·50211 | 0·0929551 | 6·6033E-08 | 0·07658714 | 11 |
| *SLC25A25* | 9 | cg14554539 | 131000000 | *SLC25A25* | 9 | ENSG00000148339 | 130851002 | rs7851605 | 9 | 130841917 | T | C | 0·138037 | 0·164805 | 0·0121391 | 5·5278E-42 | 0·276254 | 0·0451819 | 9·7004E-10 | 0·596571 | 0·107009 | 2·4756E-08 | 0·2247811 | 11 |
| *MTX2* | 2 | cg14575602 | 177000000 | *MTX2* | 2 | ENSG00000128654 | 177168438 | rs10170769 | 2 | 177132655 | T | C | 0·206544 | 0·158205 | 0·00934996 | 3·1826E-64 | -0·684843 | 0·0358931 | 3·6966E-81 | -0·231009 | 0·0182478 | 9·9094E-37 | 0·1859129 | 20 |
| *PMPCA* | 9 | cg14631541 | 139000000 | *DNLZ* | 9 | ENSG00000213221 | 139256086 | rs45464397 | 9 | 139315527 | T | C | 0·156442 | -0·135136 | 0·0110387 | 1·8541E-34 | 0·232001 | 0·0412267 | 1·829E-08 | -0·58248 | 0·113919 | 3·1692E-07 | 0·4489033 | 4 |
| *UQCRB* | 8 | cg14651183 | 97248129 | *UQCRB* | 8 | ENSG00000156467 | 97243005 | rs10955067 | 8 | 97230982 | A | G | 0·469325 | 0·103928 | 0·00796652 | 6·7325E-39 | -0·180914 | 0·0325985 | 2·8605E-08 | -0·574461 | 0·112488 | 3·2755E-07 | 0·0338285 | 3 |
| *MACROD1* | 11 | cg14842237 | 63784102 | *COX8A* | 11 | ENSG00000176340 | 63743047 | rs523586 | 11 | 63784090 | T | C | 0·403885 | -0·152568 | 0·0151541 | 7·6674E-24 | 0·975708 | 0·0267952 | 2·599E-290 | -0·156366 | 0·0161141 | 2·9066E-22 | 0·4365758 | 20 |
| *MRPL43* | 10 | cg14905631 | 103000000 | *TWNK* | 10 | ENSG00000107815 | 102750641 | rs12253241 | 10 | 102733779 | C | T | 0·183027 | -0·132232 | 0·010058 | 1·7706E-39 | 0·66041 | 0·0410742 | 3·6135E-58 | -0·200227 | 0·0196731 | 2·4932E-24 | 0·08412474 | 20 |
| *GATM* | 15 | cg14910265 | 45670406 | *GATM* | 15 | ENSG00000171766 | 45673869 | rs56850226 | 15 | 45633118 | C | T | 0·279141 | -0·572783 | 0·00830097 | 0 | 0·424852 | 0·0355205 | 5·7035E-33 | -1·34819 | 0·114399 | 4·6629E-32 | 0·3675967 | 20 |
| *HPDL* | 1 | cg14913111 | 45792733 | *MUTYH* | 1 | ENSG00000132781 | 45800488 | rs115623213 | 1 | 45404313 | A | G | 0·0398773 | -0·188563 | 0·0227283 | 1·0731E-16 | 0·454688 | 0·0752366 | 1·5086E-09 | -0·414709 | 0·0848972 | 1·0352E-06 | 0·3877575 | 5 |
| *ALKBH7* | 19 | cg14963062 | 6371673 | *SLC25A41* | 19 | ENSG00000181240 | 6429919 | rs61003547 | 19 | 6373687 | T | C | 0·0725971 | -0·0950595 | 0·0162812 | 5·2641E-09 | -0·48053 | 0·0727271 | 3·9138E-11 | 0·197822 | 0·0452148 | 1·2134E-05 | 0·09242841 | 5 |
| *NSUN4* | 1 | cg14993813 | 46806288 | *NSUN4* | 1 | ENSG00000117481 | 46818336 | rs6682266 | 1 | 46820419 | C | T | 0·273006 | 0·650098 | 0·00805595 | 0 | -0·351591 | 0·0346711 | 3·6425E-24 | -1·84902 | 0·183769 | 8·1635E-24 | 0·01083925 | 20 |
| *ACAT1* | 11 | cg14994056 | 108000000 | *ACAT1* | 11 | ENSG00000075239 | 108005373 | rs10890813 | 11 | 107987784 | A | G | 0·336401 | -0·27642 | 0·0123635 | 1·014E-110 | 0·200488 | 0·0349075 | 9·2789E-09 | -1·37874 | 0·247849 | 2·6549E-08 | 0·4231289 | 5 |
| *MACROD1* | 11 | cg15037583 | 63767176 | *COX8A* | 11 | ENSG00000176340 | 63743047 | rs7936614 | 11 | 63728137 | A | G | 0·394683 | -0·161996 | 0·0152126 | 1·7663E-26 | 0·314338 | 0·0328246 | 1·006E-21 | -0·515356 | 0·0723761 | 1·0751E-12 | 0·8258597 | 20 |
| *CHCHD4* | 3 | cg15084803 | 14160960 | *CHCHD4* | 3 | ENSG00000163528 | 14159975 | rs2129886 | 3 | 14071228 | A | G | 0·269939 | 0·0745663 | 0·0136643 | 4·8425E-08 | -0·399558 | 0·036689 | 1·2806E-27 | -0·186622 | 0·0382518 | 1·0675E-06 | 0·07165521 | 11 |
| *NUDT19* | 19 | cg15248132 | 33182730 | *NUDT19* | 19 | ENSG00000213965 | 33193784 | rs71351189 | 19 | 33176508 | C | T | 0·213701 | -0·0726543 | 0·0111488 | 7·1823E-11 | 0·32262 | 0·0389043 | 1·1075E-16 | -0·225201 | 0·0439508 | 2·9922E-07 | 0·02702437 | 10 |
| *MGST1* | 12 | cg15284911 | 16512994 | *MGST1* | 12 | ENSG00000008394 | 16631134 | rs2331818 | 12 | 16351775 | G | A | 0·46728 | 0·0641875 | 0·0079568 | 7·2047E-16 | 0·260696 | 0·031788 | 2·3825E-16 | 0·246216 | 0·0428124 | 8·8693E-09 | 0·3967909 | 17 |
| *ADCK1* | 14 | cg15388570 | 78328609 | *ADCK1* | 14 | ENSG00000063761 | 78333890 | rs3783967 | 14 | 78333139 | T | C | 0·0449898 | 1·31127 | 0·0175772 | 0 | 0·51278 | 0·0661809 | 9·3236E-15 | 2·55718 | 0·331812 | 1·2911E-14 | 0·0609917 | 20 |
| *ADCK1* | 14 | cg15388570 | 78328609 | *GSTZ1* | 14 | ENSG00000100577 | 77792583 | rs3783967 | 14 | 78333139 | T | C | 0·0449898 | 0·165026 | 0·019194 | 8·1259E-18 | 0·51278 | 0·0661809 | 9·3236E-15 | 0·321826 | 0·0559135 | 8·6241E-09 | 0·01023657 | 20 |
| *ADCK1* | 14 | cg15388570 | 78328609 | *SPTLC2* | 14 | ENSG00000100596 | 78027728 | rs77135431 | 14 | 78101720 | G | A | 0·095092 | 0·0835541 | 0·0131943 | 2·4106E-10 | 0·292898 | 0·0491063 | 2·4528E-09 | 0·285267 | 0·0657014 | 1·4128E-05 | 0·01892988 | 6 |
| *ADCK1* | 14 | cg15388570 | 78328609 | *ALKBH1* | 14 | ENSG00000100601 | 78156555 | rs3783967 | 14 | 78333139 | T | C | 0·0449898 | -0·197914 | 0·019114 | 3·9968E-25 | 0·51278 | 0·0661809 | 9·3236E-15 | -0·385963 | 0·062216 | 5·5188E-10 | 0·06599345 | 20 |
| *MRPS26* | 20 | cg15419313 | 3026568 | *MRPS26* | 20 | ENSG00000125901 | 3027745 | rs16988242 | 20 | 3026531 | C | A | 0·266871 | -0·0606753 | 0·00935366 | 8·7679E-11 | -0·255125 | 0·037537 | 1·071E-11 | 0·237826 | 0·0506814 | 2·698E-06 | 0·01152595 | 7 |
| *SPG7* | 16 | cg15473617 | 89593170 | *SPG7* | 16 | ENSG00000197912 | 89590750 | rs141882852 | 16 | 89593170 | T | C | 0·0388548 | -0·111679 | 0·0210184 | 1·076E-07 | -0·976769 | 0·0847851 | 1·0397E-30 | 0·114335 | 0·0236966 | 1·4004E-06 | 0·09982641 | 3 |
| *SPG7* | 16 | cg15473617 | 89593170 | *LOC101930112* | 16 | ENSG00000197912 | 89590750 | rs141882852 | 16 | 89593170 | T | C | 0·0388548 | -0·111679 | 0·0210184 | 1·076E-07 | -0·976769 | 0·0847851 | 1·0397E-30 | 0·114335 | 0·0236966 | 1·4004E-06 | 0·09982641 | 3 |
| *ACOT11* | 1 | cg15490897 | 55038020 | *PARS2* | 1 | ENSG00000162396 | 55226379 | rs452605 | 1 | 55032420 | T | G | 0·385481 | -0·0423295 | 0·00945193 | 7·5205E-06 | 0·451669 | 0·0318412 | 1·1336E-45 | -0·093718 | 0·0219448 | 1·9493E-05 | 0·02997893 | 8 |
| *SDHAF1* | 19 | cg15584071 | 36484913 | *SDHAF1* | 19 | ENSG00000205138 | 36486655 | rs7247214 | 19 | 36489149 | A | G | 0·162577 | -0·257649 | 0·0124161 | 1·1957E-95 | -0·401112 | 0·0491259 | 3·2148E-16 | 0·642337 | 0·0845404 | 3·0078E-14 | 0·9272788 | 10 |
| *COX6B2* | 19 | cg15681628 | 55866305 | *COX6B2* | 19 | ENSG00000160471 | 55863428 | rs2298885 | 19 | 55876240 | T | C | 0·308793 | -0·0768828 | 0·011342 | 1·2135E-11 | -0·203034 | 0·0342049 | 2·9238E-09 | 0·37867 | 0·0847956 | 7·9817E-06 | 0·2201298 | 3 |
| *ABHD10* | 3 | cg15684481 | 112000000 | *ABHD10* | 3 | ENSG00000144827 | 111705033 | rs9288933 | 3 | 111698898 | T | C | 0·221881 | 0·0972293 | 0·0100562 | 4·0995E-22 | -0·2692 | 0·0385646 | 2·9416E-12 | -0·361179 | 0·063817 | 1·5173E-08 | 0·08135054 | 7 |
| *ATAD3B* | 1 | cg15688683 | 1425815 | *PUSL1* | 1 | ENSG00000169972 | 1245502 | rs2765021 | 1 | 1297422 | C | T | 0·0787321 | 0·180387 | 0·0191819 | 5·2527E-21 | -0·380956 | 0·0631735 | 1·6364E-09 | -0·473511 | 0·0932792 | 3·849E-07 | 0·227623 | 12 |
| *ALAS1* | 3 | cg15698299 | 52233019 | *ALAS1* | 3 | ENSG00000023330 | 52240222 | rs181274 | 3 | 52233861 | T | A | 0·462168 | -0·0785179 | 0·00799392 | 9·0351E-23 | 0·353866 | 0·0321936 | 4·1849E-28 | -0·221886 | 0·0302954 | 2·4056E-13 | 0·9572633 | 20 |
| *GATM* | 15 | cg15744692 | 45671195 | *GATM* | 15 | ENSG00000171766 | 45673869 | rs2172874 | 15 | 45665452 | G | T | 0·280164 | -0·576243 | 0·00825717 | 0 | 0·641325 | 0·0347138 | 3·3032E-76 | -0·898519 | 0·0503106 | 2·4406E-71 | 0·0114995 | 20 |
| *NIPSNAP3A* | 9 | cg15754405 | 108000000 | *NIPSNAP3A* | 9 | ENSG00000136783 | 107516186 | rs12351524 | 9 | 107531127 | T | C | 0·095092 | -0·35853 | 0·0134248 | 3·951E-157 | 0·3547 | 0·0557181 | 1·9406E-10 | -1·0108 | 0·16323 | 5·9226E-10 | 0·01318879 | 3 |
| *POLDIP2* | 17 | cg15792713 | 26674270 | *POLDIP2* | 17 | ENSG00000004142 | 26679102 | rs59046213 | 17 | 26706435 | T | G | 0·0797546 | 0·32927 | 0·0136503 | 1·476E-128 | -0·398141 | 0·0566234 | 2·0449E-12 | -0·827019 | 0·122513 | 1·4739E-11 | 0·7623731 | 6 |
| *POLDIP2* | 17 | cg15792713 | 26674270 | *ERAL1* | 17 | ENSG00000132591 | 27185020 | rs59046213 | 17 | 26706435 | T | G | 0·0797546 | -0·0897892 | 0·0137663 | 6·9191E-11 | -0·398141 | 0·0566234 | 2·0449E-12 | 0·225521 | 0·0471618 | 1·7368E-06 | 0·767733 | 6 |
| *FASN* | 17 | cg15818577 | 80040107 | *DCXR* | 17 | ENSG00000169738 | 79994310 | rs1140616 | 17 | 80039481 | G | A | 0·522495 | -0·171748 | 0·00971348 | 5·8329E-70 | -0·272472 | 0·0335829 | 4·9221E-16 | 0·630333 | 0·085479 | 1·6544E-13 | 0·01618108 | 20 |
| *OXA1L* | 14 | cg15862452 | 23235611 | *OXA1L* | 14 | ENSG00000155463 | 23238369 | rs1957374 | 14 | 23235808 | A | G | 0·466258 | -0·0932554 | 0·0119292 | 5·3926E-15 | 0·311657 | 0·0327921 | 2·0193E-21 | -0·299224 | 0·0495616 | 1·5659E-09 | 0·0756566 | 8 |
| *ACSF2* | 17 | cg15880760 | 48546290 | *ACSF2* | 17 | ENSG00000167107 | 48527862 | rs9916635 | 17 | 48528962 | G | A | 0·308793 | -0·165584 | 0·00864452 | 8·8266E-82 | -0·291474 | 0·0351966 | 1·2183E-16 | 0·568092 | 0·0747359 | 2·9312E-14 | 0·1194218 | 6 |
| *ACCS* | 11 | cg15894661 | 44088124 | *ACCS* | 11 | ENSG00000110455 | 44096623 | rs2074038 | 11 | 44087989 | T | G | 0·119632 | 1·31917 | 0·0131363 | 0 | -0·721414 | 0·0510778 | 2·7089E-45 | -1·82859 | 0·130743 | 1·8934E-44 | 0·0270076 | 20 |
| *GUK1* | 1 | cg16000360 | 228000000 | *GUK1* | 1 | ENSG00000143774 | 228332174 | rs9970544 | 1 | 228348680 | A | G | 0·0337423 | -0·231639 | 0·032998 | 2·2219E-12 | -0·523412 | 0·0773529 | 1·3189E-11 | 0·442556 | 0·0908414 | 1·1062E-06 | 0·8217638 | 8 |
| *SPATA20* | 17 | cg16020904 | 48624201 | *SPATA20* | 17 | ENSG00000006282 | 48626816 | rs8076632 | 17 | 48625928 | G | C | 0·350716 | -1·01527 | 0·00680025 | 0 | 0·20007 | 0·033886 | 3·5433E-09 | -5·07457 | 0·860155 | 3·6437E-09 | 0·01526375 | 7 |
| *SPATA20* | 17 | cg16020904 | 48624201 | *ACSF2* | 17 | ENSG00000167107 | 48527862 | rs8076632 | 17 | 48625928 | G | C | 0·350716 | -0·0958872 | 0·0091396 | 9·4616E-26 | 0·20007 | 0·033886 | 3·5433E-09 | -0·479268 | 0·0931453 | 2·6698E-07 | 0·4373362 | 3 |
| *ACOT7* | 1 | cg16049283 | 6419606 | *ACOT7* | 1 | ENSG00000097021 | 6389392 | rs67984858 | 1 | 6340557 | C | T | 0·119632 | 0·209987 | 0·0136972 | 4·7755E-53 | -0·364383 | 0·053773 | 1·2328E-11 | -0·576281 | 0·0929806 | 5·7236E-10 | 0·01560714 | 10 |
| *VARS2* | 6 | cg16113650 | 30883959 | *VARS2* | 6 | ENSG00000137411 | 30885127 | rs2249935 | 6 | 31327178 | A | G | 0·174847 | -0·24644 | 0·0152994 | 2·2488E-58 | 0·201305 | 0·0366363 | 3·914E-08 | -1·22421 | 0·235405 | 1·9881E-07 | 0·08091317 | 16 |
| *SLC25A16* | 10 | cg16214034 | 70255978 | *SLC25A16* | 10 | ENSG00000122912 | 70264876 | rs10823210 | 10 | 70237246 | T | C | 0·182004 | 0·482638 | 0·00972657 | 0 | -0·307258 | 0·0428078 | 7·0936E-13 | -1·57079 | 0·221123 | 1·2147E-12 | 0·133106 | 9 |
| *SLC25A16* | 10 | cg16214034 | 70255978 | *DNA2* | 10 | ENSG00000138346 | 70202850 | rs10823210 | 10 | 70237246 | T | C | 0·182004 | -0·205509 | 0·0109797 | 3·5851E-78 | -0·307258 | 0·0428078 | 7·0936E-13 | 0·668848 | 0·099802 | 2·0594E-11 | 0·08994114 | 9 |
| *FAHD1* | 16 | cg16270890 | 1875963 | *MRPS34* | 16 | ENSG00000074071 | 1822523 | rs1657138 | 16 | 1957643 | G | A | 0·392638 | 0·0989072 | 0·00814936 | 6·7411E-34 | -0·264897 | 0·034089 | 7·8024E-15 | -0·37338 | 0·0570542 | 5·9776E-11 | 0·02319216 | 20 |
| *HAGH* | 16 | cg16270890 | 1875963 | *MRPS34* | 16 | ENSG00000074071 | 1822523 | rs1657138 | 16 | 1957643 | G | A | 0·392638 | 0·0989072 | 0·00814936 | 6·7411E-34 | -0·264897 | 0·034089 | 7·8024E-15 | -0·37338 | 0·0570542 | 5·9776E-11 | 0·02319216 | 20 |
| *BLOC1S1* | 12 | cg16337574 | 56109265 | *BLOC1S1* | 12 | ENSG00000135441 | 56111845 | rs2231462 | 12 | 56123365 | A | G | 0·0419223 | 0·339827 | 0·0460083 | 1·5108E-13 | -0·619294 | 0·113446 | 4·7905E-08 | -0·548733 | 0·124994 | 1·1332E-05 | 0·05172843 | 3 |
| *LAP3* | 4 | cg16339924 | 17578868 | *LAP3* | 4 | ENSG00000002549 | 17594205 | rs62296304 | 4 | 17578253 | G | A | 0·348671 | 0·781981 | 0·00744299 | 0 | -0·287429 | 0·0308349 | 1·1465E-20 | -2·72061 | 0·293008 | 1·6159E-20 | 0·2852197 | 20 |
| *NDUFA12* | 12 | cg16372047 | 95397610 | *NDUFA12* | 12 | ENSG00000184752 | 95344188 | rs835046 | 12 | 95398159 | T | G | 0·148262 | -0·422138 | 0·0111301 | 0 | 0·224908 | 0·0408884 | 3·7863E-08 | -1·87694 | 0·344798 | 5·2218E-08 | 0·443096 | 7 |
| *ISCA2* | 14 | cg16374328 | 74960395 | *ISCA2* | 14 | ENSG00000165898 | 74962116 | rs7148018 | 14 | 74957153 | T | C | 0·352761 | 0·545942 | 0·00754056 | 0 | 0·496175 | 0·0324309 | 7·7077E-53 | 1·1003 | 0·0735058 | 1·1723E-50 | 0·07467112 | 20 |
| *TK2* | 16 | cg16376828 | 66584228 | *TK2* | 16 | ENSG00000166548 | 66564176 | rs725131 | 16 | 66585710 | G | C | 0·134969 | 0·712605 | 0·0115019 | 0 | -0·521057 | 0·0498694 | 1·4897E-25 | -1·36761 | 0·13274 | 6·8335E-25 | 0·1095421 | 20 |
| *SPG7* | 16 | cg16428612 | 89573535 | *SPG7* | 16 | ENSG00000197912 | 89590750 | rs997212 | 16 | 89616039 | G | A | 0·484663 | 0·182747 | 0·00796061 | 1·272E-116 | -0·284123 | 0·0334502 | 1·9978E-17 | -0·643197 | 0·0807416 | 1·6374E-15 | 0·4191447 | 20 |
| *SPG7* | 16 | cg16428612 | 89573535 | *LOC101930112* | 16 | ENSG00000197912 | 89590750 | rs997212 | 16 | 89616039 | G | A | 0·484663 | 0·182747 | 0·00796061 | 1·272E-116 | -0·284123 | 0·0334502 | 1·9978E-17 | -0·643197 | 0·0807416 | 1·6374E-15 | 0·4191447 | 20 |
| *NME3* | 16 | cg16445708 | 1822320 | *MRPS34* | 16 | ENSG00000074071 | 1822523 | rs2575329 | 16 | 1819433 | G | A | 0·428425 | 0·137722 | 0·00807744 | 3·4833E-65 | -0·50033 | 0·0326288 | 4·5335E-53 | -0·275262 | 0·0241429 | 4·1154E-30 | 0·03035763 | 20 |
| *MRPS34* | 16 | cg16445708 | 1822320 | *MRPS34* | 16 | ENSG00000074071 | 1822523 | rs2575329 | 16 | 1819433 | G | A | 0·428425 | 0·137722 | 0·00807744 | 3·4833E-65 | -0·50033 | 0·0326288 | 4·5335E-53 | -0·275262 | 0·0241429 | 4·1154E-30 | 0·03035763 | 20 |
| *MRPL18* | 6 | cg16489826 | 160000000 | *MRPL18* | 6 | ENSG00000112110 | 160215156 | rs1128661 | 6 | 160211554 | T | G | 0·242331 | 0·60625 | 0·00958112 | 0 | 0·466656 | 0·0384399 | 6·4919E-34 | 1·29914 | 0·108966 | 9·0424E-33 | 0·01906966 | 20 |
| *MMAB* | 12 | cg16497413 | 110000000 | *MMAB* | 12 | ENSG00000139428 | 110002467 | rs7970557 | 12 | 110002133 | T | C | 0·176892 | -0·228764 | 0·0105844 | 1·344E-103 | 0·400983 | 0·0425083 | 3·983E-21 | -0·570508 | 0·0659889 | 5·3564E-18 | 0·0743708 | 20 |
| *STARD7* | 2 | cg16512708 | 96874307 | *STARD7* | 2 | ENSG00000084090 | 96862580 | rs2969473 | 2 | 96689334 | C | G | 0·300613 | 0·0478065 | 0·0103298 | 3·6921E-06 | 0·734334 | 0·032213 | 5·004E-115 | 0·0651018 | 0·0143539 | 5·7473E-06 | 0·01363809 | 8 |
| *MRPS35* | 12 | cg16533146 | 27901072 | *MRPS35* | 12 | ENSG00000061794 | 27886467 | rs61915375 | 12 | 27899235 | C | T | 0·176892 | -0·201597 | 0·0107219 | 7·2175E-79 | -0·828649 | 0·0404418 | 2·6474E-93 | 0·243284 | 0·0175611 | 1·2109E-43 | 0·04078389 | 20 |
| *COQ2* | 4 | cg16536390 | 84205065 | *COQ2* | 4 | ENSG00000173085 | 84194378 | rs6826758 | 4 | 84222783 | A | C | 0·275051 | 0·197479 | 0·0091878 | 1·787E-102 | 0·202434 | 0·0363654 | 2·5966E-08 | 0·975523 | 0·181026 | 7·0905E-08 | 0·01641521 | 11 |
| *LDHD* | 16 | cg16540789 | 75150784 | *LDHD* | 16 | ENSG00000166816 | 75148213 | rs9927029 | 16 | 75172340 | A | G | 0·406953 | -0·0907657 | 0·0080566 | 1·9315E-29 | 0·215857 | 0·033052 | 6·5405E-11 | -0·42049 | 0·0744214 | 1·6033E-08 | 0·664772 | 16 |
| *ACSF3* | 16 | cg16555341 | 89160798 | *ACSF3* | 16 | ENSG00000176715 | 89188518 | rs4782474 | 16 | 89157547 | G | A | 0·45501 | 0·0631159 | 0·00896609 | 1·9307E-12 | 0·181339 | 0·0317847 | 1·1619E-08 | 0·348055 | 0·0785268 | 9·3232E-06 | 0·1724358 | 13 |
| *NDUFA2* | 5 | cg16577123 | 140000000 | *HARS2* | 5 | ENSG00000112855 | 140074950 | rs1962648 | 5 | 140030306 | T | C | 0·406953 | 0·106821 | 0·0080214 | 1·8437E-40 | -0·757668 | 0·0290179 | 2·781E-150 | -0·140987 | 0·0118844 | 1·8398E-32 | 0·03492136 | 20 |
| *NDUFA2* | 5 | cg16577123 | 140000000 | *NDUFA2* | 5 | ENSG00000131495 | 140022847 | rs1962648 | 5 | 140030306 | T | C | 0·406953 | -0·331931 | 0·00950874 | 5·624E-267 | -0·757668 | 0·0290179 | 2·781E-150 | 0·438096 | 0·0209529 | 4·4736E-97 | 0·05047199 | 20 |
| *SDSL* | 12 | cg16604086 | 114000000 | *SLC8B1* | 12 | ENSG00000089060 | 113766931 | rs79557981 | 12 | 113761917 | C | G | 0·0429448 | 0·379948 | 0·0285491 | 2·0631E-40 | -0·378837 | 0·0638245 | 2·9277E-09 | -1·00293 | 0·185012 | 5·9303E-08 | 0·01236732 | 3 |
| *DMPK* | 19 | cg16686429 | 46284983 | *DMPK* | 19 | ENSG00000104936 | 46279392 | rs8107744 | 19 | 46300848 | C | A | 0·205521 | -0·205756 | 0·0105492 | 1·0073E-84 | 0·318447 | 0·0431609 | 1·6052E-13 | -0·646123 | 0·0936289 | 5·1676E-12 | 0·3229709 | 4 |
| *ACSF3* | 16 | cg16692470 | 89184092 | *ACSF3* | 16 | ENSG00000176715 | 89188518 | rs4782327 | 16 | 89184135 | G | C | 0·197342 | 0·0655282 | 0·0100267 | 6·344E-11 | -0·230825 | 0·0397387 | 6·3011E-09 | -0·283887 | 0·0653877 | 1·4145E-05 | 0·1021845 | 18 |
| *PICK1* | 22 | cg16760049 | 38452866 | *GCAT* | 22 | ENSG00000100116 | 38208547 | rs76216215 | 22 | 38460760 | A | G | 0·0184049 | 0·401017 | 0·0257296 | 9·0916E-55 | 0·812614 | 0·108349 | 6·385E-14 | 0·49349 | 0·0730211 | 1·3973E-11 | 0·01003259 | 13 |
| *PICK1* | 22 | cg16760049 | 38452866 | *PICK1* | 22 | ENSG00000100151 | 38462013 | rs76216215 | 22 | 38460760 | A | G | 0·0184049 | 0·436779 | 0·0257994 | 2·713E-64 | 0·812614 | 0·108349 | 6·385E-14 | 0·537499 | 0·0783847 | 7·0227E-12 | 0·2520462 | 12 |
| *MSRA* | 8 | cg16773768 | 10253076 | *MSRA* | 8 | ENSG00000175806 | 10099089 | rs13273009 | 8 | 10234303 | C | T | 0·194274 | -0·100726 | 0·00966697 | 2·018E-25 | 0·244028 | 0·0401863 | 1·2599E-09 | -0·412764 | 0·0786745 | 1·5504E-07 | 0·08838745 | 5 |
| *HEBP1* | 12 | cg16789995 | 13152456 | *HEBP1* | 12 | ENSG00000013583 | 13140502 | rs11055208 | 12 | 13186361 | C | G | 0·41002 | -0·234129 | 0·00897837 | 6·649E-150 | 0·167538 | 0·0286173 | 4·7866E-09 | -1·39747 | 0·244644 | 1·115E-08 | 0·01321436 | 10 |
| *NDUFA12* | 12 | cg16830870 | 95397608 | *NDUFA12* | 12 | ENSG00000184752 | 95344188 | rs844333 | 12 | 95453279 | C | G | 0·344581 | 0·641282 | 0·00773342 | 0 | -0·2127 | 0·0351217 | 1·3948E-09 | -3·01496 | 0·499166 | 1·5412E-09 | 0·0176223 | 14 |
| *COMT* | 22 | cg16834011 | 19931790 | *RTL10* | 22 | ENSG00000215012 | 19838040 | rs8140265 | 22 | 19921641 | A | G | 0·281186 | -0·0751195 | 0·0098476 | 2·3805E-14 | -0·258283 | 0·0365141 | 1·5106E-12 | 0·290842 | 0·056074 | 2·14E-07 | 0·04077016 | 6 |
| *NME3* | 16 | cg16878641 | 1821720 | *MRPS34* | 16 | ENSG00000074071 | 1822523 | rs2575369 | 16 | 1817431 | C | T | 0·445808 | 0·129004 | 0·00802773 | 4·1542E-58 | -0·184808 | 0·0328359 | 1·8206E-08 | -0·698043 | 0·131412 | 1·0851E-07 | 0·02852628 | 18 |
| *CISD1* | 10 | cg16915659 | 60032665 | *CISD1* | 10 | ENSG00000122873 | 60039082 | rs1988578 | 10 | 60003200 | G | A | 0·208589 | -0·818669 | 0·00820321 | 0 | -0·352486 | 0·0378137 | 1·1455E-20 | 2·32256 | 0·250242 | 1·6751E-20 | 0·06175298 | 20 |
| *TXN2* | 22 | cg16924664 | 36878180 | *TXN2* | 22 | ENSG00000100348 | 36870580 | rs5756209 | 22 | 36878566 | A | G | 0·338446 | 0·0942506 | 0·00858009 | 4·5224E-28 | -0·221945 | 0·0345306 | 1·2972E-10 | -0·424657 | 0·076548 | 2·8962E-08 | 0·01518567 | 6 |
| *PGAM5* | 12 | cg17037406 | 133000000 | *PGAM5* | 12 | ENSG00000247077 | 133293316 | rs10781634 | 12 | 133306589 | T | C | 0·420245 | 0·100937 | 0·00822391 | 1·2554E-34 | -0·228981 | 0·0317366 | 5·3916E-13 | -0·440809 | 0·0708704 | 4·9734E-10 | 0·1312435 | 20 |
| *MACROD1* | 11 | cg17074656 | 63784126 | *COX8A* | 11 | ENSG00000176340 | 63743047 | rs523586 | 11 | 63784090 | T | C | 0·403885 | -0·152568 | 0·0151541 | 7·6674E-24 | 0·426702 | 0·0319616 | 1·1769E-40 | -0·357552 | 0·0444808 | 9·1069E-16 | 0·2141768 | 20 |
| *COX4I2* | 20 | cg17116120 | 30225681 | *BCL2L1* | 20 | ENSG00000171552 | 30282023 | rs6088856 | 20 | 30226543 | G | C | 0·206544 | -0·0721911 | 0·0104079 | 4·0279E-12 | -0·435689 | 0·0414747 | 8·197E-26 | 0·165694 | 0·0286258 | 7·1114E-09 | 0·1967382 | 20 |
| *NDUFB8* | 10 | cg17328514 | 102000000 | *NDUFB8* | 10 | ENSG00000166136 | 102278480 | rs7079756 | 10 | 102302105 | A | G | 0·190184 | -0·0777293 | 0·00964551 | 7·7173E-16 | -0·405072 | 0·0389437 | 2·4411E-25 | 0·19189 | 0·0301222 | 1·8854E-10 | 0·3968866 | 10 |
| *MRPL34* | 19 | cg17328880 | 17415155 | *GTPBP3* | 19 | ENSG00000130299 | 17449636 | rs7252571 | 19 | 17419848 | C | T | 0·42638 | 0·1336 | 0·00934083 | 2·1019E-46 | 0·310392 | 0·0331386 | 7·4985E-21 | 0·430423 | 0·0549305 | 4·6592E-15 | 0·01403018 | 20 |
| *DMPK* | 19 | cg17499826 | 46284986 | *DMPK* | 19 | ENSG00000104936 | 46279392 | rs8107744 | 19 | 46300848 | C | A | 0·205521 | -0·205756 | 0·0105492 | 1·0073E-84 | 0·278873 | 0·0434606 | 1·3928E-10 | -0·737813 | 0·121046 | 1·0923E-09 | 0·3401302 | 4 |
| *TIMM50* | 19 | cg17512742 | 39971654 | *TIMM50* | 19 | ENSG00000105197 | 39977737 | rs2060272 | 19 | 39983459 | T | C | 0·436605 | -0·0451474 | 0·00802192 | 1·8231E-08 | 0·251785 | 0·0329457 | 2·1318E-14 | -0·179309 | 0·0395671 | 5·849E-06 | 0·3175254 | 16 |
| *PRDX5* | 11 | cg17589175 | 64085337 | *BAD* | 11 | ENSG00000002330 | 64044739 | rs11231741 | 11 | 64046885 | T | C | 0·299591 | -0·0425703 | 0·00859069 | 7·2182E-07 | -0·284662 | 0·0337056 | 3·0267E-17 | 0·149547 | 0·0349899 | 1·9199E-05 | 0·1348286 | 4 |
| *MACROD1* | 11 | cg17590162 | 63827361 | *DNAJC4* | 11 | ENSG00000110011 | 63999754 | rs2282492 | 11 | 63918589 | C | T | 0·329243 | -0·0516647 | 0·00901087 | 9·8321E-09 | 0·265981 | 0·0322233 | 1·5278E-16 | -0·194242 | 0·0412489 | 2·4891E-06 | 0·07444515 | 9 |
| *TFB1M* | 6 | cg17597176 | 156000000 | *TFB1M* | 6 | ENSG00000029639 | 155607135 | rs9397803 | 6 | 155624352 | G | C | 0·435583 | 0·21675 | 0·00799251 | 5·856E-162 | 0·263199 | 0·0327295 | 8·8638E-16 | 0·823521 | 0·106815 | 1·2599E-14 | 0·02680001 | 16 |
| *C2orf69* | 2 | cg17644776 | 201000000 | *C2orf69* | 2 | ENSG00000178074 | 200798318 | rs2881593 | 2 | 201000347 | G | A | 0·210634 | 0·0458011 | 0·00967003 | 2·1755E-06 | -0·674389 | 0·0375914 | 5·7472E-72 | -0·067915 | 0·0148303 | 4·6612E-06 | 0·4250596 | 6 |
| *ALDH7A1* | 5 | cg17653368 | 126000000 | *ALDH7A1* | 5 | ENSG00000164904 | 125904321 | rs11960347 | 5 | 125945742 | C | T | 0·0777096 | -0·129853 | 0·0210445 | 6·8117E-10 | 0·574002 | 0·0609936 | 4·9213E-21 | -0·226224 | 0·0438408 | 2·4678E-07 | 0·1830847 | 6 |
| *MACROD1* | 11 | cg17681750 | 63768665 | *COX8A* | 11 | ENSG00000176340 | 63743047 | rs7111130 | 11 | 63704817 | T | C | 0·437628 | -0·152811 | 0·0151836 | 7·953E-24 | 0·234485 | 0·0322608 | 3·6369E-13 | -0·651688 | 0·110598 | 3·8061E-09 | 0·5816473 | 19 |
| *QTRT1* | 19 | cg17710535 | 10819994 | *QTRT1* | 19 | ENSG00000213339 | 10818109 | rs11085744 | 19 | 10819967 | C | T | 0·478528 | 0·039255 | 0·00811154 | 1·3023E-06 | -0·366685 | 0·0325226 | 1·7483E-29 | -0·107054 | 0·0240729 | 8·7053E-06 | 0·0287312 | 16 |
| *MCL1* | 1 | cg17724175 | 151000000 | *TDRKH* | 1 | ENSG00000182134 | 151753237 | rs12406660 | 1 | 150836439 | C | T | 0·140082 | 0·0638358 | 0·010732 | 2·7111E-09 | 0·692296 | 0·0413738 | 7·5701E-63 | 0·0922088 | 0·0164523 | 2·0873E-08 | 0·05781035 | 9 |
| *CYP11A1* | 15 | cg17790333 | 74660265 | *CYP11A1* | 15 | ENSG00000140459 | 74645090 | rs4432229 | 15 | 74666367 | G | A | 0·161554 | 0·0817596 | 0·0114078 | 7·6659E-13 | -0·268978 | 0·0470747 | 1·1044E-08 | -0·303964 | 0·0680348 | 7·9039E-06 | 0·1277564 | 4 |
| *NSUN4* | 1 | cg17875957 | 46806823 | *NSUN4* | 1 | ENSG00000117481 | 46818336 | rs5013329 | 1 | 46815091 | T | C | 0·277096 | 0·634139 | 0·00796633 | 0 | -0·396092 | 0·0354094 | 4·7719E-29 | -1·60099 | 0·14453 | 1·6182E-28 | 0·1267653 | 20 |
| *ACSF2* | 17 | cg17890928 | 48546193 | *ACSF2* | 17 | ENSG00000167107 | 48527862 | rs58383597 | 17 | 48552455 | G | C | 0·300613 | -0·165477 | 0·00883496 | 2·8299E-78 | -0·237617 | 0·0357607 | 3·0397E-11 | 0·696402 | 0·111207 | 3·7949E-10 | 0·05247238 | 4 |
| *DHRS4* | 14 | cg17919599 | 24423864 | *DHRS4* | 14 | ENSG00000157326 | 24430641 | rs6573484 | 14 | 24428285 | G | A | 0·0511247 | -0·223199 | 0·0449672 | 6·9198E-07 | 0·734023 | 0·0759505 | 4·2673E-22 | -0·304076 | 0·0688685 | 1·0086E-05 | 0·01015296 | 4 |
| *BIK* | 22 | cg17967224 | 43506194 | *BIK* | 22 | ENSG00000100290 | 43516236 | rs5759168 | 22 | 43500435 | G | A | 0·162577 | 0·0952834 | 0·010894 | 2·2027E-18 | 0·348277 | 0·0459271 | 3·3698E-14 | 0·273585 | 0·0477494 | 1·0067E-08 | 0·01219802 | 3 |
| *CYP24A1* | 20 | cg17997279 | 52791472 | *CYP24A1* | 20 | ENSG00000019186 | 52780250 | rs2756029 | 20 | 52795949 | T | C | 0·189162 | -0·0615598 | 0·0105245 | 4·9394E-09 | 0·257934 | 0·0402996 | 1·5496E-10 | -0·238665 | 0·0552753 | 1·5763E-05 | 0·05078194 | 9 |
| *AGPAT4* | 6 | cg17999280 | 162000000 | *AGPAT4* | 6 | ENSG00000026652 | 161623052 | rs150720261 | 6 | 161565967 | T | C | 0·0623722 | 0·192005 | 0·0163153 | 5·6792E-32 | -0·364704 | 0·0638324 | 1·107E-08 | -0·526468 | 0·102431 | 2·751E-07 | 0·297527 | 3 |
| *DNLZ* | 9 | cg18033770 | 139000000 | *PMPCA* | 9 | ENSG00000165688 | 139311661 | rs11145750 | 9 | 139259249 | G | A | 0·433538 | 0·048566 | 0·00847219 | 9·9019E-09 | -0·23101 | 0·0334545 | 5·0132E-12 | -0·210233 | 0·0476651 | 1·0307E-05 | 0·5992941 | 9 |
| *CYP11A1* | 15 | cg18068537 | 74660088 | *CYP11A1* | 15 | ENSG00000140459 | 74645090 | rs4363830 | 15 | 74665055 | G | A | 0·182004 | 0·0779953 | 0·0108856 | 7·7787E-13 | -0·265711 | 0·0454853 | 5·1672E-09 | -0·293534 | 0·0648324 | 5·9661E-06 | 0·3012938 | 4 |
| *RMND1* | 6 | cg18121565 | 152000000 | *RMND1* | 6 | ENSG00000155906 | 151749624 | rs7740026 | 6 | 151750763 | T | C | 0·119632 | -0·207923 | 0·0136379 | 1·7503E-52 | -0·414535 | 0·0502966 | 1·6963E-16 | 0·501581 | 0·0691815 | 4·1608E-13 | 0·03664076 | 18 |
| *MACROD1* | 11 | cg18137308 | 63775436 | *COX8A* | 11 | ENSG00000176340 | 63743047 | rs579669 | 11 | 63780468 | G | A | 0·407975 | -0·160111 | 0·015144 | 3·9927E-26 | -0·457521 | 0·0318682 | 9·677E-47 | 0·349953 | 0·041107 | 1·6915E-17 | 0·4921252 | 20 |
| *TIMM13* | 19 | cg18147048 | 2428677 | *TIMM13* | 19 | ENSG00000099800 | 2426757 | rs1049910 | 19 | 2430637 | G | C | 0·415133 | 0·179928 | 0·00964358 | 1·0913E-77 | 0·377128 | 0·0323946 | 2·5298E-31 | 0·477101 | 0·0483054 | 5·2509E-23 | 0·02987799 | 20 |
| *MRPL2* | 6 | cg18159180 | 43022213 | *MRPL2* | 6 | ENSG00000112651 | 43024655 | rs149411734 | 6 | 43041154 | T | C | 0·0276074 | 0·439859 | 0·0231732 | 2·4303E-80 | -0·533939 | 0·077948 | 7·3882E-12 | -0·8238 | 0·127855 | 1·1697E-10 | 0·02864201 | 13 |
| *SLC25A13* | 7 | cg18184880 | 95938732 | *SLC25A13* | 7 | ENSG00000004864 | 95850495 | rs11773446 | 7 | 95944485 | A | G | 0·302658 | 0·31356 | 0·0082595 | 0 | 0·189015 | 0·034648 | 4·8892E-08 | 1·65892 | 0·307217 | 6·6706E-08 | 0·2810207 | 5 |
| *GADD45GIP1* | 19 | cg18197795 | 13067554 | *PRDX2* | 19 | ENSG00000167815 | 12910164 | rs11667458 | 19 | 13019554 | T | C | 0·200409 | 0·109666 | 0·0103439 | 2·9167E-26 | 0·328318 | 0·0425893 | 1·2687E-14 | 0·334024 | 0·0535728 | 4·5193E-10 | 0·02954225 | 5 |
| *SDHA* | 5 | cg18366108 | 226170 | *CCDC127* | 5 | ENSG00000164366 | 211601 | rs10066474 | 5 | 244147 | G | T | 0·122699 | -0·275062 | 0·0132938 | 4·1748E-95 | 0·290951 | 0·0484265 | 1·8771E-09 | -0·945389 | 0·163852 | 7·9377E-09 | 0·09889799 | 19 |
| *AMACR* | 5 | cg18397975 | 33997484 | *AMACR* | 5 | ENSG00000242110 | 33997251 | rs253201 | 5 | 34013260 | C | G | 0·143149 | 0·418863 | 0·0115857 | 3·284E-286 | -0·366264 | 0·0450987 | 4·6088E-16 | -1·14361 | 0·144324 | 2·3013E-15 | 0·3924679 | 8 |
| *MMADHC* | 2 | cg18399834 | 150000000 | *MMADHC* | 2 | ENSG00000168288 | 150435239 | rs10204262 | 2 | 150423752 | G | C | 0·110429 | 0·133385 | 0·0146227 | 7·3887E-20 | -0·524668 | 0·0567733 | 2·4317E-20 | -0·254227 | 0·0391603 | 8·4717E-11 | 0·403961 | 13 |
| *NDUFC2* | 11 | cg18402101 | 77789467 | *NARS2* | 11 | ENSG00000137513 | 78216463 | rs11237414 | 11 | 77855357 | A | G | 0·241309 | 0·0659495 | 0·00931043 | 1·4066E-12 | -0·297813 | 0·0373097 | 1·4376E-15 | -0·221446 | 0·0417971 | 1·1701E-07 | 0·1029369 | 3 |
| *DMPK* | 19 | cg18504989 | 46286198 | *DMPK* | 19 | ENSG00000104936 | 46279392 | rs635299 | 19 | 46274171 | C | A | 0·46728 | 0·278547 | 0·00781738 | 4·517E-278 | 0·288272 | 0·0317134 | 9·9125E-20 | 0·966264 | 0·109705 | 1·276E-18 | 0·2829328 | 16 |
| *MACROD1* | 11 | cg18545771 | 63827879 | *DNAJC4* | 11 | ENSG00000110011 | 63999754 | rs2282492 | 11 | 63918589 | C | T | 0·329243 | -0·0516647 | 0·00901087 | 9·8321E-09 | 0·230782 | 0·0330701 | 2·9821E-12 | -0·223868 | 0·0505331 | 9·4177E-06 | 0·08712973 | 7 |
| *GPX1* | 3 | cg18642234 | 49394622 | *GPX1* | 3 | ENSG00000233276 | 49395321 | rs7637665 | 3 | 49552295 | C | T | 0·43456 | 0·0847132 | 0·00845441 | 1·245E-23 | 0·28893 | 0·0319017 | 1·3425E-19 | 0·293196 | 0·0436373 | 1·8305E-11 | 0·0889456 | 17 |
| *ALDH7A1* | 5 | cg18670373 | 126000000 | *ALDH7A1* | 5 | ENSG00000164904 | 125904321 | rs62391691 | 5 | 126078913 | A | G | 0·0552147 | -0·27652 | 0·0264147 | 1·2067E-25 | 0·629909 | 0·0727311 | 4·6848E-18 | -0·438984 | 0·0657844 | 2·505E-11 | 0·2352509 | 9 |
| *ACOT2* | 14 | cg18674267 | 74039910 | *ACOT2* | 14 | ENSG00000119673 | 74038340 | rs186191621 | 14 | 74056994 | T | G | 0·00511247 | -1·25477 | 0·0613076 | 4·2566E-93 | 0·942122 | 0·135937 | 4·1914E-12 | -1·33186 | 0·20289 | 5·2235E-11 | 0·5257516 | 4 |
| *DNAJC30* | 7 | cg18697641 | 73097722 | *DNAJC30* | 7 | ENSG00000176410 | 73097192 | rs10229375 | 7 | 73124558 | T | C | 0·0961145 | 0·262672 | 0·0254463 | 5·5694E-25 | 0·898447 | 0·0555273 | 6·9487E-59 | 0·292362 | 0·0335955 | 3·2487E-18 | 0·2469475 | 5 |
| *NDUFA9* | 12 | cg18779092 | 4757717 | *NDUFA9* | 12 | ENSG00000139180 | 4756282 | rs2270134 | 12 | 4758788 | A | G | 0·456033 | 0·154529 | 0·0118033 | 3·6585E-39 | -0·223192 | 0·0328018 | 1·0157E-11 | -0·692359 | 0·114676 | 1·5649E-09 | 0·186205 | 8 |
| *NDUFS6* | 5 | cg18800681 | 1814073 | *MRPL36* | 5 | ENSG00000171421 | 1799990 | rs11745573 | 5 | 1801432 | A | G | 0·125767 | 0·563115 | 0·0412236 | 1·7598E-42 | -0·314149 | 0·0499639 | 3·2259E-10 | -1·79251 | 0·313841 | 1·1197E-08 | 0·01172195 | 6 |
| *PTCD1* | 7 | cg18809830 | 99032528 | *PTCD1* | 7 | ENSG00000106246 | 99039091 | rs73711294 | 7 | 99088714 | A | G | 0·0316973 | 0·192644 | 0·0209856 | 4·3189E-20 | -0·976687 | 0·0840089 | 3·0404E-31 | -0·197242 | 0·0273771 | 5·8193E-13 | 0·01837395 | 20 |
| *RAB24* | 5 | cg18824775 | 177000000 | *PRELID1* | 5 | ENSG00000169230 | 176732367 | rs6885410 | 5 | 176740996 | C | A | 0·277096 | -0·685476 | 0·0170334 | 0 | 0·222368 | 0·0347261 | 1·5188E-10 | -3·08262 | 0·487454 | 2·5501E-10 | 0·2829183 | 5 |
| *PRELID1* | 5 | cg18824775 | 177000000 | *PRELID1* | 5 | ENSG00000169230 | 176732367 | rs6885410 | 5 | 176740996 | C | A | 0·277096 | -0·685476 | 0·0170334 | 0 | 0·222368 | 0·0347261 | 1·5188E-10 | -3·08262 | 0·487454 | 2·5501E-10 | 0·2829183 | 5 |
| *IMMT* | 2 | cg18825430 | 86422958 | *IMMT* | 2 | ENSG00000132305 | 86396974 | rs150159816 | 2 | 86452556 | A | G | 0·0224949 | 0·188886 | 0·023522 | 9·7314E-16 | 0·479388 | 0·0820071 | 5·0447E-09 | 0·394015 | 0·0833706 | 2·2891E-06 | 0·9831595 | 10 |
| *IMMT* | 2 | cg18825430 | 86422958 | *MRPL35* | 2 | ENSG00000132313 | 86433748 | rs150159816 | 2 | 86452556 | A | G | 0·0224949 | -0·354022 | 0·0234573 | 1·8226E-51 | 0·479388 | 0·0820071 | 5·0447E-09 | -0·738487 | 0·135476 | 5·0067E-08 | 0·5598858 | 11 |
| *ISOC2* | 19 | cg18846804 | 55966376 | *ISOC2* | 19 | ENSG00000063241 | 55969031 | rs753754 | 19 | 55974146 | T | C | 0·128834 | 0·124745 | 0·0154406 | 6·53E-16 | 0·544189 | 0·0509971 | 1·3914E-26 | 0·229231 | 0·0355883 | 1·1855E-10 | 0·96101 | 6 |
| *DMPK* | 19 | cg18861015 | 46285653 | *DMPK* | 19 | ENSG00000104936 | 46279392 | rs10401487 | 19 | 46251460 | C | T | 0·468303 | 0·276732 | 0·00781737 | 1·73E-274 | 0·419407 | 0·0317433 | 7·4338E-40 | 0·659817 | 0·053304 | 3·4216E-35 | 0·04715325 | 20 |
| *SERHL2* | 22 | cg19042136 | 42949598 | *SERHL2* | 22 | ENSG00000183569 | 42960005 | rs137100 | 22 | 42990074 | T | C | 0·226994 | -0·254702 | 0·0169098 | 2·8621E-51 | 0·229073 | 0·036549 | 3·6674E-10 | -1·11188 | 0·192148 | 7·1828E-09 | 0·5435977 | 5 |
| *ALDH2* | 12 | cg19186356 | 112000000 | *ACAD10* | 12 | ENSG00000111271 | 112159380 | rs10744773 | 12 | 112086810 | A | C | 0·167689 | 0·0989821 | 0·0102679 | 5·4188E-22 | -0·325756 | 0·0418866 | 7·4211E-15 | -0·303853 | 0·0501997 | 1·4226E-09 | 0·3545371 | 20 |
| *PITRM1* | 10 | cg19191258 | 3197650 | *PITRM1* | 10 | ENSG00000107959 | 3197461 | rs3765099 | 10 | 3197592 | A | G | 0·225971 | 0·578285 | 0·00875214 | 0 | -0·237164 | 0·0356628 | 2·9273E-11 | -2·43833 | 0·368509 | 3·6717E-11 | 0·3680024 | 14 |
| *MUTYH* | 1 | cg19235065 | 45795593 | *NSUN4* | 1 | ENSG00000117481 | 46818336 | rs12739418 | 1 | 45850319 | C | T | 0·530675 | 0·0513328 | 0·00802577 | 1·595E-10 | 0·22401 | 0·032375 | 4·5414E-12 | 0·229154 | 0·0487899 | 2·6434E-06 | 0·01468884 | 3 |
| *COX7A2* | 6 | cg19367436 | 75953935 | *COX7A2* | 6 | ENSG00000112695 | 75953715 | rs9360898 | 6 | 75953705 | G | T | 0·198364 | 0·0668398 | 0·0145519 | 4·365E-06 | -0·584766 | 0·0386226 | 8·7528E-52 | -0·114302 | 0·0260049 | 1·1058E-05 | 0·03635094 | 3 |
| *SDHAF1* | 19 | cg19389372 | 36485356 | *SDHAF1* | 19 | ENSG00000205138 | 36486655 | rs4805149 | 19 | 36483832 | T | C | 0·162577 | -0·259919 | 0·0124906 | 3·5727E-96 | -0·44391 | 0·0493939 | 2·5373E-19 | 0·585522 | 0·0709675 | 1·5765E-16 | 0·1989692 | 10 |
| *FASTKD2* | 2 | cg19455953 | 208000000 | *FASTKD2* | 2 | ENSG00000118246 | 207643657 | rs12473950 | 2 | 207623347 | G | A | 0·0582822 | -0·113252 | 0·0153089 | 1·3846E-13 | 0·389162 | 0·0523398 | 1·0433E-13 | -0·291015 | 0·0554923 | 1·5693E-07 | 0·08166229 | 8 |
| *MRPL40* | 22 | cg19498375 | 19420336 | *MRPL40* | 22 | ENSG00000185608 | 19421511 | rs9618567 | 22 | 19413706 | T | C | 0·0981595 | -0·216599 | 0·0151555 | 2·4616E-46 | 0·492187 | 0·0551765 | 4·6535E-19 | -0·440075 | 0·0581554 | 3·8128E-14 | 0·06819274 | 15 |
| *NAGS* | 17 | cg19579216 | 42081856 | *NAGS* | 17 | ENSG00000161653 | 42084172 | rs186636 | 17 | 42085972 | C | T | 0·360941 | -0·0560292 | 0·00830629 | 1·5261E-11 | -0·254873 | 0·0342673 | 1·0238E-13 | 0·219832 | 0·0439962 | 5·8346E-07 | 0·0994402 | 14 |
| *GLS2* | 12 | cg19636672 | 56881121 | *GLS2* | 12 | ENSG00000135423 | 56873467 | rs11171862 | 12 | 56871314 | A | G | 0·313906 | 0·0608574 | 0·00858235 | 1·3315E-12 | -0·529621 | 0·0343903 | 1·6289E-53 | -0·114907 | 0·01784 | 1·1868E-10 | 0·01596236 | 14 |
| *GLS2* | 12 | cg19636672 | 56881121 | *COQ10A* | 12 | ENSG00000135469 | 56662696 | rs2657878 | 12 | 56866962 | T | C | 0·206544 | -0·0535949 | 0·0104023 | 2·5745E-07 | 0·338793 | 0·0434936 | 6·7276E-15 | -0·158194 | 0·0368128 | 1·7293E-05 | 0·5408012 | 9 |
| *BCO2* | 11 | cg19680332 | 112000000 | *BCO2* | 11 | ENSG00000197580 | 112070806 | rs10891334 | 11 | 112057100 | A | G | 0·334356 | 0·134488 | 0·00839994 | 1·0777E-57 | 0·257713 | 0·0341282 | 4·3088E-14 | 0·521852 | 0·0764082 | 8·5039E-12 | 0·1441252 | 9 |
| *ECSIT* | 19 | cg19782731 | 11641309 | *ECSIT* | 19 | ENSG00000130159 | 11628360 | rs142035232 | 19 | 11633687 | G | C | 0·0388548 | -0·209048 | 0·0231447 | 1·6825E-19 | -0·734087 | 0·0922794 | 1·7907E-15 | 0·284773 | 0·0477025 | 2·376E-09 | 0·5521441 | 9 |
| *FIS1* | 7 | cg19802458 | 101000000 | *FIS1* | 7 | ENSG00000214253 | 100889168 | rs10241107 | 7 | 100883973 | T | C | 0·0593047 | -0·920236 | 0·0175222 | 0 | -0·493421 | 0·0686575 | 6·6377E-13 | 1·86501 | 0·261927 | 1·0766E-12 | 0·9580892 | 5 |
| *CCDC127* | 5 | cg19858280 | 207590 | *SDHA* | 5 | ENSG00000073578 | 237585 | rs72711339 | 5 | 287976 | T | C | 0·149284 | 0·155953 | 0·0120578 | 2·9012E-38 | 0·329569 | 0·0530807 | 5·3379E-10 | 0·473203 | 0·0845412 | 2·1771E-08 | 0·1916169 | 19 |
| *CCDC127* | 5 | cg19858280 | 207590 | *CCDC127* | 5 | ENSG00000164366 | 211601 | rs72711339 | 5 | 287976 | T | C | 0·149284 | -0·401995 | 0·0129711 | 6·998E-211 | 0·329569 | 0·0530807 | 5·3379E-10 | -1·21976 | 0·200359 | 1·1443E-09 | 0·06325488 | 19 |
| *CHCHD3* | 7 | cg19918623 | 133000000 | *CHCHD3* | 7 | ENSG00000106554 | 132618238 | rs12707063 | 7 | 132656873 | C | A | 0·44683 | 0·0755287 | 0·0119255 | 2·3982E-10 | -0·186559 | 0·0319083 | 5·0137E-09 | -0·404852 | 0·0942388 | 1·7389E-05 | 0·01498203 | 12 |
| *MACROD1* | 11 | cg19960891 | 63776653 | *COX8A* | 11 | ENSG00000176340 | 63743047 | rs11605797 | 11 | 63743420 | G | A | 0·396728 | -0·161765 | 0·0153192 | 4·5861E-26 | 0·235698 | 0·0331359 | 1·1349E-12 | -0·686323 | 0·116337 | 3·6471E-09 | 0·4389197 | 13 |
| *PGAM5* | 12 | cg19967846 | 133000000 | *PGAM5* | 12 | ENSG00000247077 | 133293316 | rs7486197 | 12 | 133293611 | T | C | 0·425358 | 0·100089 | 0·00816546 | 1·5296E-34 | -0·198115 | 0·0313612 | 2·6634E-10 | -0·505207 | 0·0899692 | 1·9619E-08 | 0·05080096 | 20 |
| *RECQL4* | 8 | cg19996418 | 146000000 | *C8orf82* | 8 | ENSG00000213563 | 145752816 | rs2721173 | 8 | 145744429 | T | C | 0·46319 | -0·0837637 | 0·00867622 | 4·709E-22 | -0·204819 | 0·0315497 | 8·4735E-11 | 0·408965 | 0·0759135 | 7·1547E-08 | 0·07659323 | 20 |
| *DHRS4* | 14 | cg20021513 | 24438136 | *DHRS4* | 14 | ENSG00000157326 | 24430641 | rs8017358 | 14 | 24525274 | G | A | 0·47137 | -0·14865 | 0·0131279 | 1·0073E-29 | -0·199267 | 0·0321136 | 5·4672E-10 | 0·745984 | 0·13709 | 5·281E-08 | 0·05396383 | 6 |
| *CARS2* | 13 | cg20124610 | 111000000 | *CARS2* | 13 | ENSG00000134905 | 111329854 | rs9555724 | 13 | 111354110 | T | C | 0·312883 | -0·353546 | 0·012807 | 9·51E-168 | 0·405177 | 0·0373133 | 1·8117E-27 | -0·872572 | 0·0863495 | 5·2435E-24 | 0·08192152 | 20 |
| *COASY* | 17 | cg20126647 | 40714003 | *ACLY* | 17 | ENSG00000131473 | 40054978 | rs2062214 | 17 | 40593005 | C | G | 0·133947 | -0·0583024 | 0·0111918 | 1·8945E-07 | -0·425238 | 0·0451325 | 4·4264E-21 | 0·137105 | 0·0300738 | 5·1405E-06 | 0·03137681 | 4 |
| *COX16* | 14 | cg20262330 | 70826997 | *SYNJ2BP* | 14 | ENSG00000213463 | 70860963 | rs35660795 | 14 | 70739690 | C | T | 0·150307 | -0·054541 | 0·0109546 | 6·398E-07 | -0·414636 | 0·0455794 | 9·2855E-20 | 0·131539 | 0·030118 | 1·2569E-05 | 0·01822491 | 14 |
| *NDUFC2* | 11 | cg20267732 | 77787848 | *NDUFC2* | 11 | ENSG00000151366 | 77785307 | rs6592765 | 11 | 77771911 | T | C | 0·165644 | -0·341357 | 0·0109398 | 9·635E-214 | 0·318287 | 0·0445318 | 8·8436E-13 | -1·07248 | 0·153938 | 3·2385E-12 | 0·5128784 | 19 |
| *MRPL21* | 11 | cg20273122 | 68671309 | *MRPL21* | 11 | ENSG00000197345 | 68665023 | rs619727 | 11 | 68627535 | C | T | 0·300613 | -0·768104 | 0·00730215 | 0 | 0·456613 | 0·0341134 | 7·3828E-41 | -1·68218 | 0·126688 | 3·1016E-40 | 0·1165362 | 20 |
| *OXCT1* | 5 | cg20405584 | 41870875 | *OXCT1* | 5 | ENSG00000083720 | 41800394 | rs11740984 | 5 | 41897928 | T | C | 0·238241 | 0·132415 | 0·00992988 | 1·4485E-40 | -0·283059 | 0·0415622 | 9·7269E-12 | -0·4678 | 0·0771279 | 1·3175E-09 | 0·1012596 | 12 |
| *ALKBH7* | 19 | cg20752312 | 6372363 | *ALKBH7* | 19 | ENSG00000125652 | 6373743 | rs74525335 | 19 | 6372431 | G | T | 0·0920245 | 0·324543 | 0·0262215 | 3·4811E-35 | -1·21185 | 0·0521232 | 1·432E-119 | -0·267808 | 0·0245126 | 8·7216E-28 | 0·3570133 | 20 |
| *PDE2A* | 11 | cg20935363 | 72295535 | *PDE2A* | 11 | ENSG00000186642 | 72336410 | rs148038373 | 11 | 72295530 | A | C | 0·0725971 | 0·105421 | 0·0152206 | 4·323E-12 | -0·384501 | 0·0594589 | 1·0018E-10 | -0·274176 | 0·0580053 | 2·2815E-06 | 0·2143997 | 8 |
| *ZADH2* | 18 | cg20936529 | 72912760 | *ZADH2* | 18 | ENSG00000180011 | 72914183 | rs634687 | 18 | 72906960 | G | T | 0·0664622 | 0·442774 | 0·0165873 | 5·592E-157 | -0·409668 | 0·0671293 | 1·0431E-09 | -1·08081 | 0·181674 | 2·6949E-09 | 0·01943671 | 4 |
| *CYB5R3* | 22 | cg20950843 | 43044387 | *TSPO* | 22 | ENSG00000100300 | 43553384 | rs5751323 | 22 | 43057833 | T | C | 0·484663 | -0·065151 | 0·00798458 | 3·3614E-16 | 0·429392 | 0·0322423 | 1·8276E-40 | -0·151728 | 0·0218078 | 3·4626E-12 | 0·06883138 | 20 |
| *CARS2* | 13 | cg21067963 | 111000000 | *CARS2* | 13 | ENSG00000134905 | 111329854 | rs2478466 | 13 | 111313297 | C | T | 0·203476 | -0·194824 | 0·0148934 | 4·2173E-39 | 0·228164 | 0·0408839 | 2·3944E-08 | -0·853877 | 0·166345 | 2·8492E-07 | 0·1257105 | 7 |
| *CARS2* | 13 | cg21067963 | 111000000 | *NAXD* | 13 | ENSG00000213995 | 111280110 | rs9588242 | 13 | 111349894 | A | C | 0·0807771 | -0·178139 | 0·0157556 | 1·2199E-29 | 0·386965 | 0·0699302 | 3·1374E-08 | -0·460349 | 0·0926209 | 6·6867E-07 | 0·3226394 | 5 |
| *CHCHD2* | 7 | cg21096502 | 56174374 | *CHCHD2* | 7 | ENSG00000106153 | 56171765 | rs192877109 | 7 | 56217893 | T | G | 0·160532 | -0·677154 | 0·0124033 | 0 | -0·317528 | 0·0388591 | 3·0516E-16 | 2·13258 | 0·263892 | 6·4104E-16 | 0·02765466 | 20 |
| *SND1* | 7 | cg21129531 | 128000000 | *ARF5* | 7 | ENSG00000004059 | 127230079 | rs6973092 | 7 | 127660620 | T | C | 0·364008 | 0·0441326 | 0·00814465 | 6·0068E-08 | 0·33473 | 0·0337302 | 3·2818E-23 | 0·131845 | 0·0277229 | 1·9764E-06 | 0·02244285 | 16 |
| *THEM5* | 1 | cg21211478 | 152000000 | *THEM4* | 1 | ENSG00000159445 | 151864172 | rs2279503 | 1 | 151824089 | T | C | 0·307771 | -0·359053 | 0·00838555 | 0 | 0·224573 | 0·0358712 | 3·8364E-10 | -1·59883 | 0·258097 | 5·8404E-10 | 0·02787276 | 5 |
| *THEM5* | 1 | cg21211478 | 152000000 | *TDRKH* | 1 | ENSG00000182134 | 151753237 | rs2279503 | 1 | 151824089 | T | C | 0·307771 | 0·0538629 | 0·00861972 | 4·1362E-10 | 0·224573 | 0·0358712 | 3·8364E-10 | 0·239846 | 0·0542305 | 9·7469E-06 | 0·8476673 | 3 |
| *FKBP10* | 17 | cg21416237 | 39970091 | *ACLY* | 17 | ENSG00000131473 | 40054978 | rs149683818 | 17 | 39964032 | A | G | 0·0920245 | -0·197449 | 0·013889 | 7·2696E-46 | 0·480203 | 0·0529965 | 1·2922E-19 | -0·411178 | 0·0538124 | 2·1563E-14 | 0·03545154 | 11 |
| *NUDT19* | 19 | cg21498471 | 33183713 | *NUDT19* | 19 | ENSG00000213965 | 33193784 | rs71351189 | 19 | 33176508 | C | T | 0·213701 | -0·0726543 | 0·0111488 | 7·1823E-11 | -0·598387 | 0·0391594 | 1·027E-52 | 0·121417 | 0·0202549 | 2·0419E-09 | 0·01301172 | 13 |
| *COX19* | 7 | cg21511321 | 1004997 | *COX19* | 7 | ENSG00000240230 | 976825 | rs80133931 | 7 | 1027286 | T | C | 0·0685072 | -0·35786 | 0·0173838 | 3·6791E-94 | -0·350513 | 0·0598631 | 4·7631E-09 | 1·02096 | 0·181283 | 1·7828E-08 | 0·952446 | 16 |
| *COX4I2* | 20 | cg21543589 | 30225685 | *BCL2L1* | 20 | ENSG00000171552 | 30282023 | rs6088856 | 20 | 30226543 | G | C | 0·206544 | -0·0721911 | 0·0104079 | 4·0279E-12 | -0·418403 | 0·0414719 | 6·1895E-24 | 0·17254 | 0·030187 | 1·0926E-08 | 0·2904903 | 20 |
| *MTFR1* | 8 | cg21628000 | 66556877 | *ADHFE1* | 8 | ENSG00000147576 | 67363128 | rs67514002 | 8 | 66622236 | T | C | 0·173824 | -0·079701 | 0·0108917 | 2·5245E-13 | -0·273374 | 0·0430344 | 2·1191E-10 | 0·291546 | 0·0607759 | 1·6101E-06 | 0·01067945 | 14 |
| *MTO1* | 6 | cg21708354 | 74171446 | *MTO1* | 6 | ENSG00000135297 | 74195130 | rs9352002 | 6 | 74220852 | C | T | 0·0715746 | -0·440635 | 0·0153831 | 1·899E-180 | 0·541214 | 0·0681299 | 1·9599E-15 | -0·81416 | 0·106358 | 1·9342E-14 | 0·1100943 | 10 |
| *OXNAD1* | 3 | cg21761922 | 16309088 | *OXNAD1* | 3 | ENSG00000154814 | 16342764 | rs842245 | 3 | 16293675 | C | T | 0·375256 | -0·128886 | 0·00813572 | 1·5966E-56 | -0·19974 | 0·0325128 | 8·0759E-10 | 0·645269 | 0·112655 | 1·0173E-08 | 0·500928 | 8 |
| *C15orf61* | 15 | cg21788615 | 67813140 | *C15orf61* | 15 | ENSG00000189227 | 67816517 | rs72749380 | 15 | 67920129 | G | C | 0·144172 | 0·213851 | 0·0121501 | 2·4287E-69 | -0·345938 | 0·047876 | 4·9852E-13 | -0·618177 | 0·0924814 | 2·3196E-11 | 0·2936074 | 14 |
| *DMPK* | 19 | cg21904251 | 46279757 | *DMPK* | 19 | ENSG00000104936 | 46279392 | rs1865116 | 19 | 46294510 | A | G | 0·46728 | 0·281665 | 0·00779054 | 3·133E-286 | -0·674399 | 0·0293578 | 8·947E-117 | -0·417653 | 0·0215407 | 9·5508E-84 | 0·01261437 | 20 |
| *MACROD1* | 11 | cg21920570 | 63766787 | *PRDX5* | 11 | ENSG00000126432 | 64087421 | rs523586 | 11 | 63784090 | T | C | 0·403885 | -0·0898115 | 0·0081897 | 5·5436E-28 | 0·221862 | 0·0333244 | 2·7823E-11 | -0·404808 | 0·0711313 | 1·263E-08 | 0·0165107 | 19 |
| *MACROD1* | 11 | cg21920570 | 63766787 | *COX8A* | 11 | ENSG00000176340 | 63743047 | rs523586 | 11 | 63784090 | T | C | 0·403885 | -0·152568 | 0·0151541 | 7·6674E-24 | 0·221862 | 0·0333244 | 2·7823E-11 | -0·687671 | 0·123832 | 2·8038E-08 | 0·5318905 | 19 |
| *BCAT2* | 19 | cg21978618 | 49298898 | *BAX* | 19 | ENSG00000087088 | 49461563 | rs17272645 | 19 | 49342366 | G | C | 0·158487 | -0·0711322 | 0·0118748 | 2·0958E-09 | 0·314634 | 0·0456365 | 5·4112E-12 | -0·226079 | 0·0499973 | 6·1309E-06 | 0·0361023 | 7 |
| *BCAT2* | 19 | cg21978618 | 49298898 | *BCAT2* | 19 | ENSG00000105552 | 49306321 | rs17272645 | 19 | 49342366 | G | C | 0·158487 | -0·24377 | 0·0119036 | 3·3411E-93 | 0·314634 | 0·0456365 | 5·4112E-12 | -0·774773 | 0·118576 | 6·4036E-11 | 0·3531792 | 7 |
| *CBR4* | 4 | cg21985951 | 170000000 | *CBR4* | 4 | ENSG00000145439 | 169858173 | rs28371817 | 4 | 169931268 | G | A | 0·144172 | -0·316879 | 0·0108924 | 4·565E-186 | 0·246231 | 0·0411616 | 2·2033E-09 | -1·28692 | 0·21963 | 4·6435E-09 | 0·01366567 | 9 |
| *HTRA2* | 2 | cg22039458 | 74756240 | *MTHFD2* | 2 | ENSG00000065911 | 74435190 | rs9309483 | 2 | 74531420 | C | T | 0·140082 | -0·0578963 | 0·0117589 | 8·4966E-07 | -0·53511 | 0·0464111 | 9·338E-31 | 0·108195 | 0·0238946 | 5·9539E-06 | 0·02541362 | 6 |
| *SCP2* | 1 | cg22082456 | 53392538 | *SCP2* | 1 | ENSG00000116171 | 53455138 | rs562641 | 1 | 53374666 | C | T | 0·369121 | -0·672346 | 0·00731034 | 0 | 0·354112 | 0·0320362 | 2·1085E-28 | -1·89868 | 0·173008 | 5·0676E-28 | 0·03887296 | 20 |
| *LAP3* | 4 | cg22109262 | 17578738 | *LAP3* | 4 | ENSG00000002549 | 17594205 | rs12233712 | 4 | 17570063 | T | A | 0·348671 | 0·77309 | 0·0074683 | 0 | -0·322001 | 0·032127 | 1·2109E-23 | -2·40089 | 0·240665 | 1·9394E-23 | 0·1603959 | 20 |
| *COX19* | 7 | cg22126965 | 1015501 | *COX19* | 7 | ENSG00000240230 | 976825 | rs10274964 | 7 | 1019784 | T | C | 0·444785 | -0·0703589 | 0·0100769 | 2·9059E-12 | 0·181352 | 0·0327936 | 3·2005E-08 | -0·387969 | 0·089495 | 1·457E-05 | 0·02359059 | 6 |
| *SLC25A32* | 8 | cg22148115 | 104000000 | *SLC25A32* | 8 | ENSG00000164933 | 104419140 | rs3134290 | 8 | 104424190 | T | C | 0·469325 | 0·0965657 | 0·00797023 | 8·7109E-34 | 0·492742 | 0·0297284 | 1·06E-61 | 0·195976 | 0·020036 | 1·3556E-22 | 0·1920508 | 20 |
| *ALDH2* | 12 | cg22158248 | 112000000 | *ALDH2* | 12 | ENSG00000111275 | 112226236 | rs17628828 | 12 | 112091356 | T | C | 0·0664622 | 0·452227 | 0·0155965 | 7·516E-185 | -0·780638 | 0·0647397 | 1·7579E-33 | -0·579304 | 0·0520315 | 8·5961E-29 | 0·05668311 | 20 |
| *CAT* | 11 | cg22159421 | 34460182 | *CAT* | 11 | ENSG00000121691 | 34477040 | rs508370 | 11 | 34488095 | T | C | 0·40184 | 0·564749 | 0·00800146 | 0 | -0·184767 | 0·0326566 | 1·5326E-08 | -3·05655 | 0·541961 | 1·7026E-08 | 0·04595098 | 9 |
| *MRPL55* | 1 | cg22163199 | 228000000 | *GUK1* | 1 | ENSG00000143774 | 228332174 | rs11582265 | 1 | 228274018 | T | C | 0·0582822 | -0·142508 | 0·0221823 | 1·3239E-10 | 0·517878 | 0·0599709 | 5·8477E-18 | -0·275177 | 0·0533863 | 2·5439E-07 | 0·04522051 | 17 |
| *MRPL55* | 1 | cg22163199 | 228000000 | *COQ8A* | 1 | ENSG00000163050 | 227130241 | rs80096292 | 1 | 228125436 | T | G | 0·0705521 | -0·155135 | 0·0155088 | 1·4785E-23 | 0·370159 | 0·054611 | 1·2177E-11 | -0·419104 | 0·0746902 | 2·009E-08 | 0·01561136 | 6 |
| *EFHD1* | 2 | cg22193436 | 233000000 | *EFHD1* | 2 | ENSG00000115468 | 233509129 | rs10173752 | 2 | 233484154 | A | G | 0·533742 | 0·073197 | 0·00796745 | 4·0395E-20 | 0·218948 | 0·032121 | 9·3383E-12 | 0·334312 | 0·0610712 | 4·3963E-08 | 0·01931699 | 9 |
| *ACADSB* | 10 | cg22262889 | 125000000 | *ACADSB* | 10 | ENSG00000196177 | 124793161 | rs2277250 | 10 | 124768292 | G | A | 0·295501 | -0·0719905 | 0·00849586 | 2·3792E-17 | 0·330439 | 0·0330654 | 1·6276E-23 | -0·217863 | 0·0337092 | 1·0263E-10 | 0·6937851 | 4 |
| *UQCRB* | 8 | cg22313024 | 97248069 | *UQCRB* | 8 | ENSG00000156467 | 97243005 | rs10112866 | 8 | 97224627 | C | G | 0·515337 | -0·10139 | 0·0080206 | 1·2513E-36 | 0·230301 | 0·0321756 | 8·2085E-13 | -0·44025 | 0·0706831 | 4·7091E-10 | 0·02295989 | 9 |
| *NDUFC2* | 11 | cg22319984 | 77791290 | *NDUFC2* | 11 | ENSG00000151366 | 77785307 | rs11237414 | 11 | 77855357 | A | G | 0·241309 | 0·158063 | 0·00927524 | 4·0493E-65 | 0·211182 | 0·0374856 | 1·764E-08 | 0·748468 | 0·139928 | 8·8458E-08 | 0·07006542 | 4 |
| *MACROD1* | 11 | cg22408430 | 63883266 | *DNAJC4* | 11 | ENSG00000110011 | 63999754 | rs2282492 | 11 | 63918589 | C | T | 0·329243 | -0·0516647 | 0·00901087 | 9·8321E-09 | -0·215159 | 0·0324627 | 3·4051E-11 | 0·240123 | 0·0553759 | 1·4494E-05 | 0·2447471 | 3 |
| *SPATA20* | 17 | cg22450693 | 48624483 | *SPATA20* | 17 | ENSG00000006282 | 48626816 | rs9890200 | 17 | 48624523 | C | A | 0·350716 | -1·00849 | 0·00674709 | 0 | -0·386873 | 0·0320579 | 1·5596E-33 | 2·60677 | 0·216711 | 2·5073E-33 | 0·03135664 | 20 |
| *DNAJC30* | 7 | cg22527142 | 73097923 | *DNAJC30* | 7 | ENSG00000176410 | 73097192 | rs189575629 | 7 | 73084909 | A | G | 0·0337423 | 0·501105 | 0·0337118 | 5·6115E-50 | 0·640225 | 0·0807436 | 2·2072E-15 | 0·782701 | 0·111879 | 2·6339E-12 | 0·3261626 | 5 |
| *ALDH7A1* | 5 | cg22547559 | 126000000 | *ALDH7A1* | 5 | ENSG00000164904 | 125904321 | rs6861395 | 5 | 125928737 | T | C | 0·093047 | 0·481576 | 0·0190988 | 2·743E-140 | -0·462178 | 0·0567842 | 3·979E-16 | -1·04197 | 0·134523 | 9·508E-15 | 0·4800527 | 12 |
| *SUOX* | 12 | cg22580629 | 56390979 | *SUOX* | 12 | ENSG00000139531 | 56395694 | rs773109 | 12 | 56374695 | A | G | 0·320041 | -0·33178 | 0·00844564 | 0 | -0·217713 | 0·0338495 | 1·2611E-10 | 1·52393 | 0·240092 | 2·1915E-10 | 0·3837377 | 12 |
| *ATPAF1* | 1 | cg22617213 | 47134329 | *ATPAF1* | 1 | ENSG00000123472 | 47118974 | rs1371834 | 1 | 47176894 | G | A | 0·213701 | 0·141324 | 0·010252 | 3·1377E-43 | -0·270273 | 0·0365849 | 1·4954E-13 | -0·522893 | 0·0803037 | 7·4428E-11 | 0·03841528 | 4 |
| *AASS* | 7 | cg22684968 | 122000000 | *AASS* | 7 | ENSG00000008311 | 121750017 | rs74937683 | 7 | 121781468 | G | A | 0·0685072 | -0·461685 | 0·0142346 | 9·107E-231 | 0·433772 | 0·0526118 | 1·6548E-16 | -1·06435 | 0·1332 | 1·3424E-15 | 0·2295653 | 12 |
| *MTHFD2* | 2 | cg22704057 | 74425085 | *MTHFD2* | 2 | ENSG00000065911 | 74435190 | rs57740554 | 2 | 74435332 | G | A | 0·0756646 | -0·191442 | 0·0134377 | 4·7064E-46 | -0·526781 | 0·0605706 | 3·4086E-18 | 0·363419 | 0·0489577 | 1·1439E-13 | 0·01716744 | 9 |
| *PNKD* | 2 | cg22712983 | 219000000 | *PNKD* | 2 | ENSG00000127838 | 219173315 | rs4672884 | 2 | 219182481 | A | G | 0·384458 | -0·527811 | 0·00761678 | 0 | -0·399164 | 0·0325683 | 1·556E-34 | 1·32229 | 0·109562 | 1·5418E-33 | 0·1328317 | 20 |
| *ACOT2* | 14 | cg22809626 | 74036661 | *ACOT2* | 14 | ENSG00000119673 | 74038340 | rs189280747 | 14 | 73800593 | A | G | 0·00613497 | -1·22936 | 0·0713094 | 1·335E-66 | 0·762519 | 0·127403 | 2·1623E-09 | -1·61224 | 0·285146 | 1·5669E-08 | 0·2706472 | 4 |
| *NUDT19* | 19 | cg22928329 | 33183273 | *NUDT19* | 19 | ENSG00000213965 | 33193784 | rs34748593 | 19 | 33240447 | C | T | 0·178937 | -0·096663 | 0·0118946 | 4·415E-16 | 0·58576 | 0·0390778 | 8·5866E-51 | -0·165022 | 0·0230986 | 9·0515E-13 | 0·1411037 | 13 |
| *C12orf65* | 12 | cg22931309 | 124000000 | *MTRFR* | 12 | ENSG00000130921 | 123729984 | rs1727295 | 12 | 123616861 | G | A | 0·299591 | 0·0630447 | 0·00878305 | 7·0739E-13 | 0·211312 | 0·0350936 | 1·7293E-09 | 0·298349 | 0·0646732 | 3·9656E-06 | 0·1700411 | 11 |
| *QTRT1* | 19 | cg23018448 | 10811855 | *TIMM29* | 19 | ENSG00000142444 | 11041810 | rs2229383 | 19 | 10794630 | G | T | 0·402863 | -0·0827294 | 0·00845092 | 1·2504E-22 | 0·244006 | 0·0335923 | 3·765E-13 | -0·339047 | 0·0581224 | 5·4336E-09 | 0·03143628 | 20 |
| *GHITM* | 10 | cg23027704 | 85902361 | *GHITM* | 10 | ENSG00000165678 | 85906098 | rs12412411 | 10 | 85906102 | A | T | 0·316973 | -0·151046 | 0·00835986 | 5·6942E-73 | -0·281589 | 0·032117 | 1·825E-18 | 0·536406 | 0·0680032 | 3·0718E-15 | 0·03428061 | 12 |
| *NDUFS5* | 1 | cg23093580 | 39491322 | *NDUFS5* | 1 | ENSG00000168653 | 39496149 | rs10888632 | 1 | 39487144 | T | G | 0·300613 | -0·580301 | 0·00887522 | 0 | -0·209912 | 0·0352172 | 2·5146E-09 | 2·7645 | 0·465726 | 2·9226E-09 | 0·4255993 | 5 |
| *SLC25A15* | 13 | cg23211791 | 41364359 | *SLC25A15* | 13 | ENSG00000102743 | 41373897 | rs9315801 | 13 | 41586054 | A | C | 0·216769 | -0·086497 | 0·0137993 | 3·6525E-10 | -0·232776 | 0·0397317 | 4·665E-09 | 0·371589 | 0·0868162 | 1·8674E-05 | 0·2057481 | 14 |
| *THEM4* | 1 | cg23231729 | 152000000 | *THEM4* | 1 | ENSG00000159445 | 151864172 | rs112245851 | 1 | 151901126 | T | C | 0·0296524 | -0·348508 | 0·0244828 | 5·5722E-46 | -0·580482 | 0·103579 | 2·0919E-08 | 0·600377 | 0·115133 | 1·8417E-07 | 0·4199601 | 5 |
| *THEM4* | 1 | cg23231729 | 152000000 | *TDRKH* | 1 | ENSG00000182134 | 151753237 | rs112245851 | 1 | 151901126 | T | C | 0·0296524 | -0·185045 | 0·0245405 | 4·6853E-14 | -0·580482 | 0·103579 | 2·0919E-08 | 0·318778 | 0·0708717 | 6·8609E-06 | 0·8934097 | 5 |
| *HMGCL* | 1 | cg23245838 | 24152049 | *HMGCL* | 1 | ENSG00000117305 | 24146742 | rs12116511 | 1 | 24162662 | G | A | 0·411043 | 0·0961123 | 0·00819707 | 9·4676E-32 | -0·22075 | 0·0318699 | 4·3109E-12 | -0·43539 | 0·0730064 | 2·4656E-09 | 0·2642545 | 8 |
| *PCCA* | 13 | cg23271530 | 101000000 | *PCCA* | 13 | ENSG00000175198 | 100961977 | rs4772302 | 13 | 101170433 | A | G | 0·234151 | 0·170966 | 0·00886799 | 8·0621E-83 | -0·238386 | 0·0377388 | 2·6715E-10 | -0·717181 | 0·119476 | 1·9402E-09 | 0·2194121 | 4 |
| *MSRA* | 8 | cg23400122 | 10283795 | *MSRA* | 8 | ENSG00000175806 | 10099089 | rs7005363 | 8 | 10283748 | C | T | 0·424335 | -0·282558 | 0·00787131 | 3·378E-282 | -0·218922 | 0·0316188 | 4·397E-12 | 1·29068 | 0·189848 | 1·0572E-11 | 0·04202476 | 20 |
| *OXCT1* | 5 | cg23434998 | 41870910 | *OXCT1* | 5 | ENSG00000083720 | 41800394 | rs12186512 | 5 | 41802771 | G | A | 0·327198 | 0·0934816 | 0·0088034 | 2·4367E-26 | -0·261051 | 0·0362032 | 5·5661E-13 | -0·358097 | 0·0600294 | 2·441E-09 | 0·03797419 | 8 |
| *IDH3B* | 20 | cg23469025 | 2645106 | *IDH3B* | 20 | ENSG00000101365 | 2641953 | rs8958 | 20 | 2637071 | T | C | 0·391616 | -0·0889952 | 0·0082199 | 2·5698E-27 | 0·3086 | 0·032738 | 4·2454E-21 | -0·288384 | 0·0405639 | 1·1658E-12 | 0·01326055 | 7 |
| *CHCHD6* | 3 | cg23542533 | 127000000 | *CHCHD6* | 3 | ENSG00000159685 | 126551156 | rs7633071 | 3 | 126561086 | T | A | 0·143149 | -0·0669633 | 0·011482 | 5·4767E-09 | -0·454454 | 0·0461235 | 6·6535E-23 | 0·147349 | 0·0293597 | 5·2011E-07 | 0·03116014 | 16 |
| *C2orf69* | 2 | cg23649088 | 201000000 | *C2orf69* | 2 | ENSG00000178074 | 200798318 | rs2881593 | 2 | 201000347 | G | A | 0·210634 | 0·0458011 | 0·00967003 | 2·1755E-06 | -0·853063 | 0·035771 | 1·066E-125 | -0·0536902 | 0·0115571 | 3·3899E-06 | 0·2882715 | 6 |
| *MACROD1* | 11 | cg23719950 | 63933701 | *DNAJC4* | 11 | ENSG00000110011 | 63999754 | rs11603192 | 11 | 63927695 | G | A | 0·323108 | -0·0540683 | 0·00899309 | 1·8302E-09 | -0·337236 | 0·0324303 | 2·5108E-25 | 0·160328 | 0·0308033 | 1·9411E-07 | 0·1813345 | 9 |
| *GDAP1* | 8 | cg23779890 | 75262522 | *GDAP1* | 8 | ENSG00000104381 | 75317236 | rs6996971 | 8 | 75292492 | T | C | 0·45501 | 0·113781 | 0·0079787 | 3·8514E-46 | -0·322318 | 0·0322407 | 1·5671E-23 | -0·353009 | 0·0431231 | 2·6992E-16 | 0·1560691 | 20 |
| *BAD* | 11 | cg23796481 | 64053134 | *DNAJC4* | 11 | ENSG00000110011 | 63999754 | rs604203 | 11 | 64016935 | C | A | 0·460123 | 0·040364 | 0·0085546 | 2·3771E-06 | -0·339026 | 0·0320886 | 4·3159E-26 | -0·119059 | 0·0276348 | 1·6453E-05 | 0·6858196 | 3 |
| *FKBP10* | 17 | cg23796525 | 39978853 | *ACLY* | 17 | ENSG00000131473 | 40054978 | rs60588087 | 17 | 39982432 | A | C | 0·269939 | -0·0933387 | 0·00983693 | 2·3416E-21 | -0·254754 | 0·0356378 | 8·7783E-13 | 0·366388 | 0·0641717 | 1·1332E-08 | 0·01239024 | 10 |
| *MRPL12* | 17 | cg23847089 | 79672787 | *MRPL12* | 17 | ENSG00000262814 | 79672471 | rs11867435 | 17 | 79655232 | A | G | 0·219836 | -0·0798394 | 0·0116646 | 7·669E-12 | 0·229401 | 0·0410123 | 2·2258E-08 | -0·348034 | 0·0803557 | 1·4832E-05 | 0·01341777 | 6 |
| *IMMT* | 2 | cg23876751 | 86422994 | *IMMT* | 2 | ENSG00000132305 | 86396974 | rs9653508 | 2 | 86422816 | T | G | 0·49591 | 0·507182 | 0·00745725 | 0 | -0·330588 | 0·032289 | 1·3342E-24 | -1·53418 | 0·151534 | 4·3092E-24 | 0·01746421 | 20 |
| *PANK2* | 20 | cg23908269 | 3868713 | *MAVS* | 20 | ENSG00000088888 | 3838383 | rs73084594 | 20 | 3916380 | G | T | 0·184049 | -0·134465 | 0·0108028 | 1·4484E-35 | 0·241725 | 0·0416777 | 6·6369E-09 | -0·556273 | 0·105812 | 1·4629E-07 | 0·0150882 | 7 |
| *PANK2* | 20 | cg23908269 | 3868713 | *PANK2* | 20 | ENSG00000125779 | 3888545 | rs73084594 | 20 | 3916380 | G | T | 0·184049 | -0·269204 | 0·0107222 | 4·15E-139 | 0·241725 | 0·0416777 | 6·6369E-09 | -1·11368 | 0·197075 | 1·5948E-08 | 0·20455 | 7 |
| *PUSL1* | 1 | cg23931819 | 1245076 | *MRPL20* | 1 | ENSG00000242485 | 1339990 | rs11804831 | 1 | 1194804 | C | T | 0·192229 | 0·207405 | 0·0135663 | 9·1646E-53 | -0·229358 | 0·0402871 | 1·2476E-08 | -0·904285 | 0·169495 | 9·5451E-08 | 0·5091009 | 20 |
| *GPX1* | 3 | cg24011261 | 49396226 | *GPX1* | 3 | ENSG00000233276 | 49395321 | rs111903592 | 3 | 49427319 | C | G | 0·336401 | -0·109219 | 0·00902219 | 9·8645E-34 | -0·242777 | 0·0351205 | 4·7554E-12 | 0·449874 | 0·0749425 | 1·938E-09 | 0·2256945 | 11 |
| *ADCK2* | 7 | cg24013954 | 140000000 | *NDUFB2* | 7 | ENSG00000090266 | 140406583 | rs2968548 | 7 | 140406499 | C | G | 0·158487 | -0·444324 | 0·0119999 | 4·165E-300 | 0·309964 | 0·0497412 | 4·6188E-10 | -1·43347 | 0·23327 | 7·9903E-10 | 0·8482926 | 6 |
| *EXOG* | 3 | cg24069376 | 38537580 | *EXOG* | 3 | ENSG00000157036 | 38560635 | rs7372545 | 3 | 38507570 | T | G | 0·324131 | 0·0446129 | 0·00935948 | 1·8736E-06 | -0·573938 | 0·0336471 | 3·0704E-65 | -0·0777312 | 0·0169322 | 4·417E-06 | 0·528992 | 3 |
| *MRPL9* | 1 | cg24153003 | 152000000 | *MRPL9* | 1 | ENSG00000143436 | 151734079 | rs11204881 | 1 | 151730369 | A | G | 0·338446 | 0·054628 | 0·00842999 | 9·1601E-11 | -0·420298 | 0·0332647 | 1·3545E-36 | -0·129974 | 0·0225413 | 8·1146E-09 | 0·1125882 | 10 |
| *TSTD1* | 1 | cg24161057 | 161000000 | *TSTD1* | 1 | ENSG00000215845 | 161008100 | rs10908821 | 1 | 161008535 | G | C | 0·128834 | 0·173743 | 0·0144754 | 3·4431E-33 | -0·3338 | 0·052569 | 2·1566E-10 | -0·5205 | 0·0927359 | 1·9917E-08 | 0·02896083 | 10 |
| *ABCD3* | 1 | cg24215727 | 94883469 | *ABCD3* | 1 | ENSG00000117528 | 94934077 | rs2786906 | 1 | 94824664 | A | G | 0·332311 | 0·149091 | 0·00813221 | 4·4802E-75 | -0·206299 | 0·034305 | 1·8136E-09 | -0·722694 | 0·126475 | 1·1027E-08 | 0·01863348 | 20 |
| *VARS2* | 6 | cg24268004 | 30882373 | *VARS2* | 6 | ENSG00000137411 | 30885127 | rs2233980 | 6 | 31079644 | A | G | 0·0838446 | -0·312535 | 0·0198475 | 7·2247E-56 | 0·240782 | 0·0434822 | 3·0685E-08 | -1·298 | 0·248474 | 1·752E-07 | 0·04080853 | 20 |
| *GPT2* | 16 | cg24306924 | 46918433 | *GPT2* | 16 | ENSG00000166123 | 46941749 | rs117939346 | 16 | 46852813 | G | A | 0·0265849 | -0·490804 | 0·030501 | 2·9313E-58 | 0·679672 | 0·104556 | 8·0021E-11 | -0·722119 | 0·119808 | 1·667E-09 | 0·2633072 | 8 |
| *SLC25A24* | 1 | cg24323958 | 109000000 | *SLC25A24* | 1 | ENSG00000085491 | 108710064 | rs2840307 | 1 | 108742952 | T | G | 0·302658 | 0·400343 | 0·00837772 | 0 | -0·35745 | 0·0343951 | 2·6835E-25 | -1·12 | 0·110289 | 3·1454E-24 | 0·09804435 | 20 |
| *GATM* | 15 | cg24328539 | 45671016 | *GATM* | 15 | ENSG00000171766 | 45673869 | rs12593371 | 15 | 45640084 | A | G | 0·283231 | -0·570475 | 0·00829354 | 0 | 0·691459 | 0·0346311 | 1·0788E-88 | -0·825031 | 0·0430265 | 5·9917E-82 | 0·01084309 | 20 |
| *CHPT1* | 12 | cg24337881 | 102000000 | *CHPT1* | 12 | ENSG00000111666 | 102114321 | rs2695287 | 12 | 102088361 | T | C | 0·390593 | -0·64575 | 0·00722715 | 0 | -0·200887 | 0·0325672 | 6·8993E-10 | 3·21449 | 0·522365 | 7·568E-10 | 0·2802047 | 3 |
| *TSTD1* | 1 | cg24411043 | 161000000 | *PPOX* | 1 | ENSG00000143224 | 161142001 | rs2247472 | 1 | 160976238 | G | A | 0·210634 | -0·0749032 | 0·0097818 | 1·8973E-14 | -0·373624 | 0·0410263 | 8·4731E-20 | 0·200477 | 0·0342059 | 4·6033E-09 | 0·3917602 | 14 |
| *TSTD1* | 1 | cg24411043 | 161000000 | *NDUFS2* | 1 | ENSG00000158864 | 161175539 | rs2247472 | 1 | 160976238 | G | A | 0·210634 | -0·0490536 | 0·00987113 | 6·716E-07 | -0·373624 | 0·0410263 | 8·4731E-20 | 0·131291 | 0·0300974 | 1·2875E-05 | 0·405499 | 8 |
| *LDHD* | 16 | cg24429836 | 75150744 | *LDHD* | 16 | ENSG00000166816 | 75148213 | rs8058619 | 16 | 75182354 | C | G | 0·296524 | -0·0966531 | 0·00874863 | 2·2465E-28 | 0·243336 | 0·0361876 | 1·7645E-11 | -0·3972 | 0·0691506 | 9·2475E-09 | 0·5983485 | 16 |
| *SPATA20* | 17 | cg24438145 | 48624694 | *SPATA20* | 17 | ENSG00000006282 | 48626816 | rs8076632 | 17 | 48625928 | G | C | 0·350716 | -1·01527 | 0·00680025 | 0 | 0·791094 | 0·0303565 | 1·032E-149 | -1·28337 | 0·0499912 | 2·402E-145 | 0·01507954 | 20 |
| *CHCHD6* | 3 | cg24448870 | 127000000 | *CHCHD6* | 3 | ENSG00000159685 | 126551156 | rs4679311 | 3 | 126669726 | G | A | 0·310838 | -0·0687757 | 0·00880002 | 5·4789E-15 | 0·234404 | 0·0361706 | 9·1409E-11 | -0·293407 | 0·0588154 | 6·0818E-07 | 0·2786056 | 8 |
| *TDRKH* | 1 | cg24503712 | 152000000 | *MRPL9* | 1 | ENSG00000143436 | 151734079 | rs12066445 | 1 | 151740420 | A | G | 0·341513 | 0·0544858 | 0·0084587 | 1·1838E-10 | -0·274473 | 0·0341706 | 9·5551E-16 | -0·198511 | 0·0395033 | 5·0299E-07 | 0·3022722 | 10 |
| *COASY* | 17 | cg24535823 | 40713676 | *COASY* | 17 | ENSG00000068120 | 40715890 | rs4792930 | 17 | 40805844 | T | G | 0·477505 | -0·0528906 | 0·00801762 | 4·2013E-11 | 0·212046 | 0·0321319 | 4·1329E-11 | -0·24943 | 0·0534626 | 3·0786E-06 | 0·04172982 | 7 |
| *SCP2* | 1 | cg24543286 | 53392881 | *SCP2* | 1 | ENSG00000116171 | 53455138 | rs1242331 | 1 | 53392908 | A | G | 0·349693 | -0·724308 | 0·00742631 | 0 | 0·292581 | 0·0313247 | 9·6098E-21 | -2·47558 | 0·266257 | 1·4349E-20 | 0·5571871 | 20 |
| *SCP2* | 1 | cg24543286 | 53392881 | *ECHDC2* | 1 | ENSG00000121310 | 53377270 | rs1242331 | 1 | 53392908 | A | G | 0·349693 | -0·564276 | 0·00785791 | 0 | 0·292581 | 0·0313247 | 9·6098E-21 | -1·92861 | 0·208223 | 2·0019E-20 | 0·04309672 | 20 |
| *CYP24A1* | 20 | cg24582168 | 52789646 | *CYP24A1* | 20 | ENSG00000019186 | 52780250 | rs2756028 | 20 | 52795655 | C | T | 0·168712 | -0·0623749 | 0·0111344 | 2·1189E-08 | 0·275353 | 0·0415531 | 3·4365E-11 | -0·226527 | 0·0529503 | 1·8849E-05 | 0·02862898 | 8 |
| *MACROD1* | 11 | cg24687543 | 63912206 | *BAD* | 11 | ENSG00000002330 | 64044739 | rs2096706 | 11 | 63924446 | G | T | 0·138037 | 0·0880072 | 0·0110346 | 1·5164E-15 | 0·408927 | 0·0423382 | 4·5205E-22 | 0·215215 | 0·0349949 | 7·7535E-10 | 0·6830327 | 20 |
| *GTPBP3* | 19 | cg24720006 | 17448183 | *MRPL34* | 19 | ENSG00000130312 | 17410535 | rs7259703 | 19 | 17462792 | A | G | 0·0214724 | -0·216187 | 0·0271414 | 1·6496E-15 | 0·779334 | 0·121924 | 1·6378E-10 | -0·2774 | 0·0556443 | 6·1889E-07 | 0·5847636 | 4 |
| *ACSL1* | 4 | cg24721647 | 186000000 | *ACSL1* | 4 | ENSG00000151726 | 185712360 | rs7676928 | 4 | 185735054 | G | A | 0·464213 | -0·0582209 | 0·0080932 | 6·3012E-13 | 0·191041 | 0·0325027 | 4·1602E-09 | -0·304756 | 0·0669557 | 5·3237E-06 | 0·7779421 | 5 |
| *ACSL1* | 4 | cg24721647 | 186000000 | *CASP3* | 4 | ENSG00000164305 | 185559756 | rs7676928 | 4 | 185735054 | G | A | 0·464213 | -0·0520998 | 0·00809431 | 1·2218E-10 | 0·191041 | 0·0325027 | 4·1602E-09 | -0·272715 | 0·0628329 | 1·4227E-05 | 0·3590715 | 6 |
| *ABCB6* | 2 | cg24775454 | 220000000 | *CYP27A1* | 2 | ENSG00000135929 | 219663244 | rs3731894 | 2 | 220136371 | T | C | 0·0787321 | -0·0795348 | 0·0148535 | 8·5746E-08 | 0·479001 | 0·0520153 | 3·2966E-20 | -0·166043 | 0·0358705 | 3·6752E-06 | 0·2595285 | 3 |
| *ECSIT* | 19 | cg24813710 | 11618984 | *ECSIT* | 19 | ENSG00000130159 | 11628360 | rs2871605 | 19 | 11621129 | C | T | 0·0388548 | -0·193526 | 0·0213855 | 1·4376E-19 | -0·842774 | 0·0865248 | 2·0303E-22 | 0·22963 | 0·0346366 | 3·364E-11 | 0·05043963 | 20 |
| *PANK2* | 20 | cg24825299 | 3896885 | *PANK2* | 20 | ENSG00000125779 | 3888545 | rs4813650 | 20 | 3886094 | T | C | 0·486708 | -0·205688 | 0·00801315 | 2·608E-145 | -0·187569 | 0·0323102 | 6·4269E-09 | 1·0966 | 0·193668 | 1·494E-08 | 0·2053733 | 3 |
| *ACADVL* | 17 | cg24825722 | 7121848 | *ACADVL* | 17 | ENSG00000072778 | 7124518 | rs41283403 | 17 | 7122828 | T | C | 0·0388548 | 0·240039 | 0·022734 | 4·6352E-26 | 0·651154 | 0·0893507 | 3·1548E-13 | 0·368636 | 0·0614628 | 2·0011E-09 | 0·1730976 | 7 |
| *ECHS1* | 10 | cg24905316 | 135000000 | *ECHS1* | 10 | ENSG00000127884 | 135181588 | rs4838722 | 10 | 135161180 | C | T | 0·0419223 | 0·264154 | 0·023888 | 2·0052E-28 | 1·22732 | 0·0760265 | 1·2654E-58 | 0·215228 | 0·023592 | 7·3116E-20 | 0·06538897 | 20 |
| *COQ2* | 4 | cg24939380 | 84189574 | *COQ2* | 4 | ENSG00000173085 | 84194378 | rs6535454 | 4 | 84191031 | A | G | 0·255624 | 0·249421 | 0·00878195 | 1·935E-177 | 0·332069 | 0·0355908 | 1·0567E-20 | 0·751112 | 0·0847359 | 7·709E-19 | 0·03798251 | 20 |
| *DNA2* | 10 | cg25000555 | 70232188 | *SLC25A16* | 10 | ENSG00000122912 | 70264876 | rs12767599 | 10 | 70247476 | C | A | 0·137014 | 0·59115 | 0·0105246 | 0 | -0·328315 | 0·048869 | 1·839E-11 | -1·80056 | 0·269919 | 2·5455E-11 | 0·4479783 | 12 |
| *DNA2* | 10 | cg25000555 | 70232188 | *DNA2* | 10 | ENSG00000138346 | 70202850 | rs12767599 | 10 | 70247476 | C | A | 0·137014 | -0·261785 | 0·011946 | 1·91E-106 | -0·328315 | 0·048869 | 1·839E-11 | 0·797359 | 0·124137 | 1·3344E-10 | 0·2244551 | 12 |
| *PARK7* | 1 | cg25007680 | 8021821 | *PARK7* | 1 | ENSG00000116288 | 8029958 | rs226253 | 1 | 8027706 | T | C | 0·403885 | -0·236169 | 0·00796181 | 2·324E-193 | -0·271107 | 0·0304986 | 6·1564E-19 | 0·871128 | 0·102305 | 1·6652E-17 | 0·06551062 | 17 |
| *HMGCL* | 1 | cg25037394 | 24152592 | *HMGCL* | 1 | ENSG00000117305 | 24146742 | rs7514394 | 1 | 24100223 | C | T | 0·404908 | 0·101084 | 0·00816735 | 3·4985E-35 | -0·407563 | 0·0321978 | 1·0093E-36 | -0·248021 | 0·0280268 | 8·7975E-19 | 0·1326162 | 12 |
| *GCAT* | 22 | cg25043629 | 38202659 | *GCAT* | 22 | ENSG00000100116 | 38208547 | rs1894644 | 22 | 38202552 | T | C | 0·124744 | 0·826882 | 0·010974 | 0 | -0·326286 | 0·0483666 | 1·5187E-11 | -2·53422 | 0·37716 | 1·8269E-11 | 0·9551705 | 4 |
| *CYB5R3* | 22 | cg25044876 | 43041146 | *TSPO* | 22 | ENSG00000100300 | 43553384 | rs130333 | 22 | 43066379 | C | T | 0·488753 | -0·0644886 | 0·00798917 | 6·9156E-16 | -0·242799 | 0·0321437 | 4·2359E-14 | 0·265605 | 0·0481574 | 3·481E-08 | 0·887736 | 15 |
| *GHITM* | 10 | cg25186695 | 85899272 | *GHITM* | 10 | ENSG00000165678 | 85906098 | rs78338638 | 10 | 85888929 | C | T | 0·0368098 | -0·311778 | 0·0202251 | 1·2897E-53 | -0·447008 | 0·0819612 | 4·9278E-08 | 0·697477 | 0·135654 | 2·7242E-07 | 0·553392 | 4 |
| *OGG1* | 3 | cg25207828 | 9791044 | *RPUSD3* | 3 | ENSG00000156990 | 9882909 | rs3219008 | 3 | 9795543 | G | A | 0·188139 | -0·0624752 | 0·00987875 | 2·5455E-10 | 0·29693 | 0·0398255 | 8·9341E-14 | -0·210404 | 0·0436263 | 1·4151E-06 | 0·01481652 | 5 |
| *MIPEP* | 13 | cg25267304 | 24462978 | *MIPEP* | 13 | ENSG00000027001 | 24383943 | rs3794340 | 13 | 24455686 | C | T | 0·255624 | -0·130128 | 0·00917415 | 1·1477E-45 | 0·346512 | 0·0359167 | 5·0298E-22 | -0·375537 | 0·0470759 | 1·4961E-15 | 0·384315 | 19 |
| *FDXR* | 17 | cg25268537 | 72860402 | *FDXR* | 17 | ENSG00000161513 | 72863887 | rs7219247 | 17 | 72847205 | T | C | 0·294479 | -0·0849485 | 0·0089752 | 2·9412E-21 | 0·200481 | 0·0353528 | 1·4208E-08 | -0·423723 | 0·0871045 | 1·1472E-06 | 0·0508858 | 8 |
| *SLC25A39* | 17 | cg25269247 | 42401439 | *SLC25A39* | 17 | ENSG00000013306 | 42399615 | rs8079946 | 17 | 42401708 | C | T | 0·408998 | 0·110103 | 0·00805105 | 1·4203E-42 | -0·329515 | 0·0337666 | 1·6946E-22 | -0·334137 | 0·0420638 | 1·9645E-15 | 0·09164301 | 14 |
| *NDUFA13* | 19 | cg25274157 | 19626576 | *NDUFA13* | 19 | ENSG00000186010 | 19635415 | rs34487417 | 19 | 19509701 | T | C | 0·171779 | 0·167527 | 0·0121352 | 2·3777E-43 | -0·329562 | 0·0426862 | 1·1581E-14 | -0·508332 | 0·0754384 | 1·6017E-11 | 0·6897467 | 20 |
| *PRDX6* | 1 | cg25302646 | 173000000 | *MRPS14* | 1 | ENSG00000120333 | 174986243 | rs2097548 | 1 | 175004730 | G | A | 0·476483 | 0·0531158 | 0·00796554 | 2·5896E-11 | -0·228592 | 0·0325202 | 2·0768E-12 | -0·232361 | 0·048031 | 1·3133E-06 | 0·1658306 | 5 |
| *MRPL4* | 19 | cg25371692 | 10362856 | *MRPL4* | 19 | ENSG00000105364 | 10366649 | rs73015182 | 19 | 10367852 | G | T | 0·293456 | -0·0877196 | 0·00973515 | 2·0493E-19 | -0·349107 | 0·0341667 | 1·6513E-24 | 0·251269 | 0·0371801 | 1·3976E-11 | 0·07198345 | 17 |
| *OGG1* | 3 | cg25415932 | 9791051 | *RPUSD3* | 3 | ENSG00000156990 | 9882909 | rs55814656 | 3 | 9795194 | A | C | 0·188139 | -0·0624917 | 0·00988199 | 2·5521E-10 | 0·287663 | 0·040067 | 6·9946E-13 | -0·217239 | 0·0457783 | 2·0803E-06 | 0·01722444 | 5 |
| *ALDH7A1* | 5 | cg25476565 | 126000000 | *ALDH7A1* | 5 | ENSG00000164904 | 125904321 | rs56397023 | 5 | 125915779 | A | G | 0·0940695 | 0·468945 | 0·0188348 | 7·86E-137 | -0·830132 | 0·0555312 | 1·5828E-50 | -0·564904 | 0·0440771 | 1·3294E-37 | 0·10288 | 20 |
| *MOCS1* | 6 | cg25571957 | 39902693 | *MOCS1* | 6 | ENSG00000124615 | 39884822 | rs2475508 | 6 | 39900020 | C | A | 0·41002 | -0·0432907 | 0·00817299 | 1·1785E-07 | 0·275382 | 0·0322018 | 1·2123E-17 | -0·157202 | 0·0349105 | 6·6998E-06 | 0·1182299 | 10 |
| *GRPEL1* | 4 | cg25660036 | 7070649 | *GRPEL1* | 4 | ENSG00000109519 | 7065278 | rs4689586 | 4 | 7067413 | A | G | 0·231084 | -0·110207 | 0·00982307 | 3·2823E-29 | 0·524432 | 0·0382178 | 7·4761E-43 | -0·210145 | 0·0241945 | 3·7649E-18 | 0·0282708 | 20 |
| *MTFMT* | 15 | cg25698089 | 65321903 | *MTFMT* | 15 | ENSG00000103707 | 65308411 | rs2946655 | 15 | 65321938 | G | A | 0·0398773 | 0·193235 | 0·0219436 | 1·2969E-18 | 0·484985 | 0·0745714 | 7·8404E-11 | 0·398435 | 0·0761603 | 1·6811E-07 | 0·2897562 | 3 |
| *ACOT7* | 1 | cg25706257 | 6390530 | *ACOT7* | 1 | ENSG00000097021 | 6389392 | rs57440563 | 1 | 6466944 | G | T | 0·102249 | 0·282018 | 0·014024 | 6·0812E-90 | -0·412587 | 0·055256 | 8·2151E-14 | -0·683536 | 0·0976498 | 2·562E-12 | 0·0708115 | 9 |
| *GFM1* | 3 | cg25727025 | 158000000 | *GFM1* | 3 | ENSG00000168827 | 158386215 | rs7624771 | 3 | 158335409 | T | C | 0·218814 | 0·0885709 | 0·0096453 | 4·2002E-20 | -0·276983 | 0·0405785 | 8·7402E-12 | -0·31977 | 0·0583717 | 4·2974E-08 | 0·04460278 | 9 |
| *NMNAT3* | 3 | cg25780109 | 139000000 | *NMNAT3* | 3 | ENSG00000163864 | 139337940 | rs35487059 | 3 | 139284004 | G | A | 0·194274 | -0·0799401 | 0·0101692 | 3·8108E-15 | -0·234103 | 0·040013 | 4·8957E-09 | 0·341474 | 0·0727558 | 2·6866E-06 | 0·08783118 | 5 |
| *ACSF2* | 17 | cg25792518 | 48545950 | *MRPL27* | 17 | ENSG00000108826 | 48447896 | rs9896330 | 17 | 48570614 | T | A | 0·375256 | 0·0640576 | 0·0087004 | 1·8036E-13 | -0·205779 | 0·0342045 | 1·7862E-09 | -0·311293 | 0·0668204 | 3·1828E-06 | 0·04193451 | 5 |
| *AIFM3* | 22 | cg25976804 | 21335632 | *AIFM3* | 22 | ENSG00000183773 | 21327522 | rs744931 | 22 | 21332409 | G | A | 0·476483 | 0·129256 | 0·00901568 | 1·2886E-46 | -0·32291 | 0·0329021 | 9·7736E-23 | -0·400285 | 0·049427 | 5·5638E-16 | 0·08591229 | 9 |
| *MRPL43* | 10 | cg26023709 | 103000000 | *SFXN3* | 10 | ENSG00000107819 | 102795994 | rs12359673 | 10 | 102711762 | G | A | 0·0582822 | 0·103646 | 0·0155191 | 2·4124E-11 | 0·498425 | 0·0591936 | 3·7568E-17 | 0·207947 | 0·0397412 | 1·6721E-07 | 0·8931329 | 4 |
| *MRPS11* | 15 | cg26025224 | 89020059 | *MRPS11* | 15 | ENSG00000181991 | 89016453 | rs11633528 | 15 | 89086392 | A | G | 0·142127 | -0·0590339 | 0·0110249 | 8·5746E-08 | -0·320718 | 0·045181 | 1·2611E-12 | 0·184068 | 0·043059 | 1·9132E-05 | 0·02899936 | 4 |
| *COX4I2* | 20 | cg26070540 | 30225666 | *BCL2L1* | 20 | ENSG00000171552 | 30282023 | rs6060425 | 20 | 30224355 | T | C | 0·207566 | -0·0709434 | 0·0104785 | 1·2843E-11 | -0·417304 | 0·0400515 | 2·0267E-25 | 0·170004 | 0·0299455 | 1·3699E-08 | 0·2950746 | 20 |
| *CARS2* | 13 | cg26090534 | 111000000 | *CARS2* | 13 | ENSG00000134905 | 111329854 | rs9588224 | 13 | 111312221 | G | A | 0·204499 | -0·195996 | 0·0149924 | 4·6976E-39 | -0·220903 | 0·0396395 | 2·5068E-08 | 0·887249 | 0·173073 | 2·9526E-07 | 0·09704237 | 3 |
| *CYP27A1* | 2 | cg26104932 | 220000000 | *CYP27A1* | 2 | ENSG00000135929 | 219663244 | rs7573075 | 2 | 219630666 | G | A | 0·468303 | 0·854256 | 0·00636721 | 0 | 0·30213 | 0·0327 | 2·4779E-20 | 2·82745 | 0·306744 | 3·038E-20 | 0·01914364 | 18 |
| *CYP27A1* | 2 | cg26175971 | 220000000 | *CYP27A1* | 2 | ENSG00000135929 | 219663244 | rs141941813 | 2 | 219632749 | C | A | 0·462168 | 0·799723 | 0·00744671 | 0 | 0·287499 | 0·0332868 | 5·769E-18 | 2·78165 | 0·323101 | 7·3547E-18 | 0·03127474 | 16 |
| *CHCHD5* | 2 | cg26236440 | 113000000 | *CHCHD5* | 2 | ENSG00000125611 | 113344334 | rs34865009 | 2 | 113340189 | T | C | 0·095092 | 0·155212 | 0·0150449 | 5·9285E-25 | -0·338923 | 0·0542059 | 4·0392E-10 | -0·457957 | 0·0856454 | 8·9362E-08 | 0·09648158 | 3 |
| *LONP1* | 19 | cg26242866 | 5711310 | *MICOS13* | 19 | ENSG00000174917 | 5679669 | rs118168835 | 19 | 5713486 | A | G | 0·0235174 | 0·263243 | 0·027676 | 1·8775E-21 | 0·803248 | 0·127647 | 3·1198E-10 | 0·327723 | 0·0624457 | 1·5365E-07 | 0·1444613 | 11 |
| *LONP1* | 19 | cg26242866 | 5711310 | *LONP1* | 19 | ENSG00000196365 | 5706214 | rs2436508 | 19 | 5711217 | C | T | 0·0889571 | 0·169429 | 0·0131884 | 8·9641E-38 | 0·837466 | 0·0563686 | 6·2697E-50 | 0·202312 | 0·020819 | 2·5357E-22 | 0·04061174 | 20 |
| *ALDH7A1* | 5 | cg26327732 | 126000000 | *ALDH7A1* | 5 | ENSG00000164904 | 125904321 | rs77900059 | 5 | 125947276 | A | G | 0·0766871 | -0·141543 | 0·0214375 | 4·0401E-11 | 0·597954 | 0·0615997 | 2·8124E-22 | -0·236712 | 0·0433586 | 4·7775E-08 | 0·5774903 | 6 |
| *THEM4* | 1 | cg26347189 | 152000000 | *THEM4* | 1 | ENSG00000159445 | 151864172 | rs1490187 | 1 | 151844396 | C | T | 0·0848671 | -0·724341 | 0·0138528 | 0 | 0·397652 | 0·0610359 | 7·2665E-11 | -1·82154 | 0·281752 | 1·0126E-10 | 0·9509808 | 8 |
| *SLC25A40* | 7 | cg26365742 | 87503866 | *SLC25A40* | 7 | ENSG00000075303 | 87484277 | rs143725456 | 7 | 87144023 | C | T | 0·0173824 | -0·317074 | 0·0600179 | 1·2709E-07 | 1·42162 | 0·116259 | 2·203E-34 | -0·223037 | 0·0459896 | 1·2363E-06 | 0·02005653 | 4 |
| *PUSL1* | 1 | cg26369180 | 1246414 | *MRPL20* | 1 | ENSG00000242485 | 1339990 | rs115464315 | 1 | 1242288 | G | T | 0·0337423 | 0·185022 | 0·0347107 | 9·7997E-08 | 0·712977 | 0·0922523 | 1·0878E-14 | 0·259506 | 0·0591406 | 1·1442E-05 | 0·01216109 | 20 |
| *NDUFS8* | 11 | cg26406131 | 67803771 | *NDUFS8* | 11 | ENSG00000110717 | 67801097 | rs3133269 | 11 | 67804156 | C | T | 0·281186 | -0·313027 | 0·00890707 | 1·453E-270 | 0·228183 | 0·0371567 | 8·1954E-10 | -1·37182 | 0·226769 | 1·4536E-09 | 0·5058929 | 3 |
| *BID* | 22 | cg26427090 | 18217141 | *BID* | 22 | ENSG00000015475 | 18237221 | rs13058451 | 22 | 18167859 | C | T | 0·157464 | -0·0803716 | 0·0102309 | 3·9723E-15 | -0·264652 | 0·042165 | 3·4611E-10 | 0·303688 | 0·0619312 | 9·4078E-07 | 0·2181883 | 9 |
| *BID* | 22 | cg26427090 | 18217141 | *BCL2L13* | 22 | ENSG00000099968 | 18162504 | rs13058451 | 22 | 18167859 | C | T | 0·157464 | 0·145837 | 0·010208 | 2·6525E-46 | -0·264652 | 0·042165 | 3·4611E-10 | -0·551052 | 0·0958942 | 9·1134E-09 | 0·06976142 | 9 |
| *VARS2* | 6 | cg26467571 | 30882355 | *VARS2* | 6 | ENSG00000137411 | 30885127 | rs9264490 | 6 | 31232578 | G | A | 0·225971 | -0·175736 | 0·0140279 | 5·2734E-36 | 0·282467 | 0·0345632 | 3·0211E-16 | -0·622147 | 0·0908936 | 7·6594E-12 | 0·01013221 | 20 |
| *MACROD1* | 11 | cg26467918 | 63932088 | *BAD* | 11 | ENSG00000002330 | 64044739 | rs947939 | 11 | 63885287 | T | C | 0·137014 | 0·0907893 | 0·0111022 | 2·8955E-16 | 0·317972 | 0·0420973 | 4·2448E-14 | 0·285526 | 0·0514594 | 2·8801E-08 | 0·9950026 | 20 |
| *MACROD1* | 11 | cg26467918 | 63932088 | *PRDX5* | 11 | ENSG00000126432 | 64087421 | rs143643736 | 11 | 63936043 | T | A | 0·0143149 | -0·179218 | 0·0291687 | 8·0368E-10 | -0·957963 | 0·115162 | 8·9161E-17 | 0·187082 | 0·037854 | 7·7244E-07 | 0·06770646 | 6 |
| *SPRYD4* | 12 | cg26547506 | 56863038 | *GLS2* | 12 | ENSG00000135423 | 56873467 | rs10783792 | 12 | 56958673 | T | A | 0·177914 | -0·0560318 | 0·0110447 | 3·9118E-07 | -0·416329 | 0·0431012 | 4·4872E-22 | 0·134585 | 0·0299651 | 7·0753E-06 | 0·6857801 | 7 |
| *SPRYD4* | 12 | cg26547506 | 56863038 | *COQ10A* | 12 | ENSG00000135469 | 56662696 | rs10783792 | 12 | 56958673 | T | A | 0·177914 | -0·0524439 | 0·0110079 | 1·896E-06 | -0·416329 | 0·0431012 | 4·4872E-22 | 0·125967 | 0·0294816 | 1·9307E-05 | 0·6700861 | 12 |
| *SDHAF1* | 19 | cg26643967 | 36485282 | *SDHAF1* | 19 | ENSG00000205138 | 36486655 | rs7251575 | 19 | 36488986 | C | T | 0·230061 | -0·236708 | 0·0105753 | 5·738E-111 | -0·513069 | 0·0422401 | 5·9877E-34 | 0·461357 | 0·043215 | 1·3202E-26 | 0·2766149 | 19 |
| *BPHL* | 6 | cg26651049 | 3144103 | *BPHL* | 6 | ENSG00000137274 | 3136210 | rs9392465 | 6 | 3162378 | C | A | 0·315951 | -0·111121 | 0·00848226 | 3·2753E-39 | -0·199927 | 0·0332179 | 1·7587E-09 | 0·555808 | 0·101627 | 4·5237E-08 | 0·2860125 | 12 |
| *CS* | 12 | cg26734620 | 56694298 | *COQ10A* | 12 | ENSG00000135469 | 56662696 | rs11575234 | 12 | 56744276 | G | C | 0·0664622 | -0·0795648 | 0·0155085 | 2·8913E-07 | 0·917634 | 0·0600262 | 9·3024E-53 | -0·0867065 | 0·0178269 | 1·1515E-06 | 0·01318868 | 11 |
| *BCL2L13* | 22 | cg26766542 | 18211034 | *BID* | 22 | ENSG00000015475 | 18237221 | rs2587100 | 22 | 18210704 | C | G | 0·336401 | -0·0595481 | 0·00848626 | 2·2668E-12 | -0·191059 | 0·0335102 | 1·1875E-08 | 0·311674 | 0·0704354 | 9·6459E-06 | 0·01062208 | 5 |
| *HPDL* | 1 | cg26781150 | 45792542 | *MUTYH* | 1 | ENSG00000132781 | 45800488 | rs61789859 | 1 | 45806933 | A | G | 0·0439673 | -0·213652 | 0·0216418 | 5·4947E-23 | 0·444412 | 0·0711891 | 4·3009E-10 | -0·480752 | 0·0911156 | 1·3183E-07 | 0·415722 | 5 |
| *PNKD* | 2 | cg26786924 | 219000000 | *PNKD* | 2 | ENSG00000127838 | 219173315 | rs897877 | 2 | 219192755 | A | G | 0·387526 | -0·52565 | 0·00764386 | 0 | -0·366245 | 0·0329682 | 1·1335E-28 | 1·43524 | 0·130871 | 5·5145E-28 | 0·5063405 | 20 |
| *GATM* | 15 | cg26796135 | 45671001 | *GATM* | 15 | ENSG00000171766 | 45673869 | rs12593371 | 15 | 45640084 | A | G | 0·283231 | -0·570475 | 0·00829354 | 0 | 0·620775 | 0·0351955 | 1·2602E-69 | -0·918972 | 0·0537877 | 1·9136E-65 | 0·01640177 | 20 |
| *CARS2* | 13 | cg26801538 | 111000000 | *CARS2* | 13 | ENSG00000134905 | 111329854 | rs378399 | 13 | 111308040 | C | G | 0·342536 | -0·312315 | 0·012293 | 2·165E-142 | -0·271263 | 0·0337769 | 9·667E-16 | 1·15134 | 0·150353 | 1·8953E-14 | 0·03007292 | 20 |
| *COQ10A* | 12 | cg26804944 | 56660921 | *COQ10A* | 12 | ENSG00000135469 | 56662696 | rs57137641 | 12 | 56741228 | A | G | 0·0644172 | -0·0786409 | 0·015693 | 5·4092E-07 | -0·58399 | 0·0628982 | 1·6218E-20 | 0·134661 | 0·0305363 | 1·0343E-05 | 0·8381451 | 4 |
| *CRAT* | 9 | cg26805528 | 132000000 | *CRAT* | 9 | ENSG00000095321 | 131865278 | rs6478862 | 9 | 131867365 | T | C | 0·274029 | -0·547293 | 0·00833637 | 0 | 0·226414 | 0·0338422 | 2·2274E-11 | -2·41722 | 0·363175 | 2·8174E-11 | 0·142749 | 15 |
| *AGPAT5* | 8 | cg26814335 | 6584320 | *AGPAT5* | 8 | ENSG00000155189 | 6591531 | rs2980689 | 8 | 6579323 | T | A | 0·345603 | 0·155953 | 0·00823284 | 5·0631E-80 | 0·347654 | 0·032714 | 2·2301E-26 | 0·448587 | 0·0484007 | 1·8932E-20 | 0·253333 | 20 |
| *PLD6* | 17 | cg26906998 | 17108846 | *PLD6* | 17 | ENSG00000179598 | 17106969 | rs34235236 | 17 | 17118442 | C | T | 0·303681 | -0·24546 | 0·0112083 | 2·609E-106 | 0·26489 | 0·0359048 | 1·6122E-13 | -0·926649 | 0·132539 | 2·7198E-12 | 0·0146282 | 9 |
| *MDH2* | 7 | cg26942532 | 75678159 | *MDH2* | 7 | ENSG00000146701 | 75687097 | rs4732513 | 7 | 75607608 | C | T | 0·355828 | 0·0619237 | 0·0125969 | 8·8421E-07 | 0·409698 | 0·0330558 | 2·8114E-35 | 0·151145 | 0·0330768 | 4·8887E-06 | 0·01717351 | 3 |
| *STYXL1* | 7 | cg26942532 | 75678159 | *MDH2* | 7 | ENSG00000146701 | 75687097 | rs4732513 | 7 | 75607608 | C | T | 0·355828 | 0·0619237 | 0·0125969 | 8·8421E-07 | 0·409698 | 0·0330558 | 2·8114E-35 | 0·151145 | 0·0330768 | 4·8887E-06 | 0·01717351 | 3 |
| *DECR1* | 8 | cg27040700 | 91017761 | *DECR1* | 8 | ENSG00000104325 | 91038976 | rs2189619 | 8 | 91059247 | G | A | 0·343558 | 0·102207 | 0·0144027 | 1·2805E-12 | 0·224924 | 0·0337885 | 2·798E-11 | 0·454407 | 0·0935946 | 1·2035E-06 | 0·3072443 | 20 |
| *NDUFB8* | 10 | cg27072683 | 102000000 | *NDUFB8* | 10 | ENSG00000166136 | 102278480 | rs4919470 | 10 | 102298920 | T | C | 0·191207 | -0·0784186 | 0·00962617 | 3·7492E-16 | -0·530686 | 0·0384029 | 1·9599E-43 | 0·147768 | 0·0210564 | 2·2548E-12 | 0·5788163 | 10 |
| *COQ9* | 16 | cg27077106 | 57481227 | *COQ9* | 16 | ENSG00000088682 | 57488262 | rs59247888 | 16 | 57448361 | C | T | 0·0306748 | -0·247242 | 0·020754 | 1·0127E-32 | 0·419626 | 0·0751189 | 2·3215E-08 | -0·589196 | 0·116494 | 4·243E-07 | 0·8426164 | 3 |
| *ACSL1* | 4 | cg27112742 | 186000000 | *ACSL1* | 4 | ENSG00000151726 | 185712360 | rs55830512 | 4 | 185683945 | A | G | 0·170757 | -0·0537481 | 0·0103489 | 2·0627E-07 | -0·543561 | 0·0438792 | 3·0473E-35 | 0·0988815 | 0·0206447 | 1·6705E-06 | 0·04507276 | 10 |
| *NDUFS1* | 2 | cg27248887 | 207000000 | *NDUFS1* | 2 | ENSG00000023228 | 207005238 | rs12478671 | 2 | 207033835 | C | T | 0·474438 | -0·0564732 | 0·0087724 | 1·2138E-10 | 0·463321 | 0·0323931 | 2·0934E-46 | -0·121888 | 0·0207631 | 4·3475E-09 | 0·09840863 | 18 |
| *CLYBL* | 13 | cg27300028 | 100000000 | *CLYBL* | 13 | ENSG00000125246 | 100404153 | rs8000435 | 13 | 100310215 | C | G | 0·108384 | -0·48704 | 0·0127476 | 0 | 0·30806 | 0·0512126 | 1·7953E-09 | -1·58099 | 0·266065 | 2·8135E-09 | 0·01759503 | 9 |
| *COQ5* | 12 | cg27304573 | 121000000 | *SIRT4* | 12 | ENSG00000089163 | 120745585 | rs540520 | 12 | 120911786 | A | G | 0·309816 | 0·0452167 | 0·00884729 | 3·208E-07 | -0·301822 | 0·0366039 | 1·6431E-16 | -0·149812 | 0·034487 | 1·3989E-05 | 0·02541034 | 6 |
| *COQ5* | 12 | cg27304573 | 121000000 | *COQ5* | 12 | ENSG00000110871 | 120956657 | rs540520 | 12 | 120911786 | A | G | 0·309816 | -0·282434 | 0·00938652 | 6·685E-199 | -0·301822 | 0·0366039 | 1·6431E-16 | 0·935763 | 0·11767 | 1·829E-15 | 0·01294595 | 14 |
| *COQ5* | 12 | cg27304573 | 121000000 | *GATC* | 12 | ENSG00000257218 | 120891815 | rs540520 | 12 | 120911786 | A | G | 0·309816 | 0·257496 | 0·00862452 | 7·279E-196 | -0·301822 | 0·0366039 | 1·6431E-16 | -0·853139 | 0·107339 | 1·8943E-15 | 0·03258965 | 14 |
| *CHCHD6* | 3 | cg27308130 | 126000000 | *CHCHD6* | 3 | ENSG00000159685 | 126551156 | rs2102958 | 3 | 126419805 | C | T | 0·161554 | -0·0737463 | 0·0105748 | 3·0849E-12 | 0·310806 | 0·0426367 | 3·1081E-13 | -0·237274 | 0·0470859 | 4·6754E-07 | 0·1057423 | 20 |
| *NME4* | 16 | cg27315249 | 450562 | *NME4* | 16 | ENSG00000103202 | 453546 | rs35122295 | 16 | 469188 | C | A | 0·342536 | 0·262836 | 0·0089338 | 3·011E-190 | -0·208936 | 0·0328535 | 2·0225E-10 | -1·25797 | 0·202375 | 5·0981E-10 | 0·02593055 | 20 |
| *RHOT2* | 16 | cg27336518 | 722515 | *MCRIP2* | 16 | ENSG00000172366 | 695143 | rs1044662 | 16 | 715060 | C | T | 0·373211 | -0·0607726 | 0·00933556 | 7·5251E-11 | 0·452656 | 0·0334334 | 9·2013E-42 | -0·134258 | 0·0228841 | 4·4412E-09 | 0·1637513 | 7 |
| *LAP3* | 4 | cg27347728 | 17578864 | *LAP3* | 4 | ENSG00000002549 | 17594205 | rs7658240 | 4 | 17588950 | G | A | 0·340491 | 0·780178 | 0·0075467 | 0 | -0·51281 | 0·0307195 | 1·4662E-62 | -1·52138 | 0·0923176 | 5·1222E-61 | 0·09224615 | 20 |
| *PGAM5* | 12 | cg27362255 | 133000000 | *PGAM5* | 12 | ENSG00000247077 | 133293316 | rs7313839 | 12 | 133324216 | A | G | 0·383436 | -0·0669071 | 0·00809601 | 1·4063E-16 | -0·198737 | 0·0319809 | 5·1575E-10 | 0·336662 | 0·0677831 | 6·8086E-07 | 0·01475837 | 11 |
| *CLPB* | 11 | cg27367093 | 72145746 | *CLPB* | 11 | ENSG00000162129 | 72074580 | rs117190635 | 11 | 72104119 | A | G | 0·0460123 | -0·157588 | 0·0202978 | 8·2421E-15 | -0·959463 | 0·0795688 | 1·754E-33 | 0·164246 | 0·0251611 | 6·6754E-11 | 0·5283476 | 20 |
| *MACROD1* | 11 | cg27373749 | 63775612 | *COX8A* | 11 | ENSG00000176340 | 63743047 | rs4980517 | 11 | 63745677 | T | C | 0·396728 | -0·159966 | 0·0152027 | 6·8261E-26 | -0·674946 | 0·0305424 | 3·258E-108 | 0·237006 | 0·0249473 | 2·0941E-21 | 0·5460423 | 20 |
| *PDE2A* | 11 | cg27419075 | 72295168 | *PDE2A* | 11 | ENSG00000186642 | 72336410 | rs73534668 | 11 | 72194420 | T | C | 0·47546 | 0·0458809 | 0·0079943 | 9·5125E-09 | 0·254166 | 0·0327585 | 8·5757E-15 | 0·180515 | 0·0391229 | 3·9488E-06 | 0·03369989 | 5 |
| *DNAJC15* | 13 | cg27496116 | 43660964 | *DNAJC15* | 13 | ENSG00000120675 | 43640192 | rs6561105 | 13 | 43648719 | A | G | 0·518405 | -0·69521 | 0·0103389 | 0 | -0·269294 | 0·0318234 | 2·6249E-17 | 2·5816 | 0·307483 | 4·6223E-17 | 0·2452594 | 10 |
| *GFM1* | 3 | cg27533704 | 158000000 | *GFM1* | 3 | ENSG00000168827 | 158386215 | rs17699324 | 3 | 158424561 | C | T | 0·220859 | 0·0873782 | 0·00963652 | 1·2189E-19 | -0·288426 | 0·0409024 | 1·7692E-12 | -0·302948 | 0·0544243 | 2·6002E-08 | 0·08100637 | 10 |
| *ARF5* | 7 | cg27562431 | 127000000 | *ARF5* | 7 | ENSG00000004059 | 127230079 | rs1419428 | 7 | 127230276 | C | A | 0·411043 | -0·0670629 | 0·00799567 | 4·9701E-17 | 0·28878 | 0·0341552 | 2·7926E-17 | -0·232228 | 0·0390004 | 2·6084E-09 | 0·6218886 | 7 |

eQTL: expression quantitative trait loci.

mQTL: methylation quantitative trait loci.

SMR: summary-data-based Mendelian randomization.

HEIDI: heterogeneity in dependent instruments.

Only genome-wide significant mQTLs (*P* <5E-8) are taken into the analysis. We report SNP-gene combinations with *P*_SMR_ < genome-wide significance Bonferonni correction threshold of 0·05/nprobe (2550), and survived after the heterogeneity test (*P*_HEIDI_ ≥ 0·01).

β in eQTL association, regression coefficient of gene expression on SNP, log(OR).

SE, standard error.

β in mQTL association, regression coefficient of DNA methylation on SNP.

β in SMR association, regression coefficient of DNA methylation om gene expression.

**Supplementary Table 9.** Phenome-wide scan of the association between identified SNPs with other diseases traits using PhenoScanner.

| **Type of Cancer** | **Gene** | **Gene Chr·** | **Probe** | **topSNP** | **Effect allele** | **Other allele** | **Diseases and traits** | **study** | **β** | **SE** | ***P*** | **N** | **N_cases** | **N_controls** | **Ancestry** |
| --- | --- | --- | --- | --- | --- | --- | --- | --- | --- | --- | --- | --- | --- | --- | --- |
| Breast cancer | *PARK7* | 1 | ENSG00000116288 | rs35675666 | T | G | Ulcerative colitis | Anderson CA | -0·07696 | 0·01316 | 5·00E-09 | NA | NA | NA | European |
|  |  |  |  |  |  |  | Inflammatory bowel disease | IBDGC | -0·1419 | 0·0237 | 2·041E-09 | 34,652 | 12,882 | 21,770 | European |
|  | *NSUN4* | 1 | ENSG00000117481 | rs41293273 | C | T | ·· | ·· | ·· | ·· | ·· | ·· | ·· | ·· | ·· |
|  | *FDPS* | 1 | ENSG00000160752 | rs6677385 | A | C | ·· | ·· | ·· | ·· | ·· | ·· | ·· | ·· | ·· |
|  | *MPC2* | 1 | ENSG00000143158 | rs203777 | T | C | ·· | ·· | ·· | ·· | ·· | ·· | ·· | ·· | ·· |
|  | *MRPS18C* | 4 | ENSG00000163319 | rs1565909 | C | T | Upper aerodigestive tract cancers | McKay JD | -0·1133 | 0·01978 | 1·00E-08 | NA | NA | NA | European |
|  |  |  |  |  |  |  | Age at menopause | ReproGen | 0·19 | 0·02 | 1·2E-21 | 69,360 | 0 | 69,360 | European |
|  | *YBEY* | 21 | ENSG00000182362 | rs62224180 | C | G | ·· | ·· | ·· | ·· | ·· | ·· | ·· | ·· | ·· |
|  | *HSCB* | 22 | ENSG00000100209 | rs6519752 | G | A | ·· | ·· | ·· | ·· | ·· | ·· | ·· | ·· | ·· |
|  | *NSUN4* | 1 | cg14993813 | rs6682266 | C | T | ·· | ·· | ·· | ·· | ·· | ·· | ·· | ·· | ·· |
|  |  |  | cg17875957 | rs5013329 | T | C | ·· | ·· | ·· | ·· | ·· | ·· | ·· | ·· | ·· |
|  |  |  | cg17806798 | rs41293277 | T | C | ·· | ·· | ·· | ·· | ·· | ·· | ·· | ·· | ·· |
|  |  |  | cg04241075 |  |  |  | ·· | ·· | ·· | ·· | ·· | ·· | ·· | ·· | ·· |
|  |  |  | cg06741803 | rs56063031 | T | C | ·· | ·· | ·· | ·· | ·· | ·· | ·· | ·· | ·· |
|  |  |  | cg00530320 | rs6681857 | C | T | ·· | ·· | ·· | ·· | ·· | ·· | ·· | ·· | ·· |
|  |  |  | cg15580309 | rs111226885 | C | T | ·· | ·· | ·· | ·· | ·· | ·· | ·· | ·· | ·· |
|  | *SLC25A44* | 1 | cg19263494 | rs72708291 | T | A | ·· | ·· | ·· | ·· | ·· | ·· | ·· | ·· | ·· |
|  | *BCL2L11* | 2 | cg09907170 | rs73954926 | G | T | Hip circumference | Neale B | -0·02725 | 0·004923 | 3·106E-08 | 336,601 | 0 | 336,601 | European |
|  |  |  |  |  |  |  | Pulse rate | Neale B | 0·02958 | 0·005115 | 7·331E-09 | 317,756 | 0 | 317,756 | European |
|  |  |  | cg27608154 | rs73954941 | G | T | Hip circumference | Neale B | -0·02741 | 0·00491 | 2·381E-08 | 336,601 | 0 | 336,601 | European |
|  |  |  |  |  |  |  | Pulse rate | Neale B | 0·02921 | 0·005101 | 1·024E-08 | 317,756 | 0 | 317,756 | European |
|  | *SLC25A22* | 11 | cg23587532 | rs7928917 | T | G | ·· | ·· | ·· | ·· | ·· | ·· | ·· | ·· | ·· |
|  |  |  | cg11475788 | rs61876744 | T | C | ·· | ·· | ·· | ·· | ·· | ·· | ·· | ·· | ·· |
|  | *MRPL23* | 11 | cg07977153 | rs4929956 | G | T | ·· | ·· | ·· | ·· | ·· | ·· | ·· | ·· | ·· |
|  |  |  |  |  |  |  | ·· | ·· | ·· | ·· | ·· | ·· | ·· | ·· | ·· |
|  | *TRMT1* | 19 | cg22237401 | rs74569397 | T | C | ·· | ·· | ·· | ·· | ·· | ·· | ·· | ·· | ·· |
|  |  |  | cg12014333 | rs16995252 | G | A | ·· | ·· | ·· | ·· | ·· | ·· | ·· | ·· | ·· |
|  |  |  |  |  |  |  |  |  |  |  |  |  |  |  |  |
| Prostate cancer | *CASP8* | 2 | ENSG00000064012 | rs7560328 | A | C | Lymphocyte count | Astle W | -0·03056 | 0·003741 | 3·114E-16 | 173,480 | 0 | 173,480 | European |
|  |  |  |  |  |  |  | Lymphocyte percentage of white cells | Astle W | -0·02618 | 0·003703 | 1·542E-12 | 173,480 | 0 | 173,480 | European |
|  |  |  |  |  |  |  | Neutrophil percentage of white cells | Astle W | 0·02505 | 0·003709 | 1·436E-11 | 173,480 | 0 | 173,480 | European |
|  |  |  |  |  |  |  | Plateletcrit | Astle W | -0·02259 | 0·003803 | 2·861E-09 | 173,480 | 0 | 173,480 | European |
|  | *UQCC1* | 20 | ENSG00000101019 | rs4911178 | G | A | Arm fat-free mass left | Neale B | -0·02974 | 0·001598 | 2·778E-77 | 331,159 | 0 | 331,159 | European |
|  |  |  |  |  |  |  | Arm fat-free mass right | Neale B | -0·02937 | 0·001566 | 2·167E-78 | 331,221 | 0 | 331,221 | European |
|  |  |  |  |  |  |  | Arm predicted mass left | Neale B | -0·0298 | 0·001592 | 3·788E-78 | 331,146 | 0 | 331,146 | European |
|  |  |  |  |  |  |  | Arm predicted mass right | Neale B | -0·0293 | 0·001561 | 1·354E-78 | 331,216 | 0 | 331,216 | European |
|  |  |  |  |  |  |  | Basal metabolic rate | Neale B | -0·03362 | 0·001667 | 1·942E-90 | 331,307 | 0 | 331,307 | European |
|  |  |  |  |  |  |  | Comparative height size at age 10 | Neale B | -0·03891 | 0·001715 | 7·677E-114 | 332,021 | 0 | 332,021 | European |
|  |  |  |  |  |  |  | Forced expiratory volume in 1-second· predicted | Neale B | -0·0269 | 0·002816 | 1·283E-21 | 110,423 | 0 | 110,423 | European |
|  |  |  |  |  |  |  | Hand grip strength right | Neale B | -0·0215 | 0·001817 | 2·648E-32 | 335,842 | 0 | 335,842 | European |
|  |  |  |  |  |  |  | Height | Neale B | -0·05664 | 0·001784 | 8·585E-221 | 336,474 | 0 | 336,474 | European |
|  |  |  |  |  |  |  | Hip circumference | Neale B | -0·02466 | 0·00249 | 4·12E-23 | 336,601 | 0 | 336,601 | European |
|  |  |  |  |  |  |  | Impedance of leg left | Neale B | 0·02171 | 0·002293 | 2·868E-21 | 331,296 | 0 | 331,296 | European |
|  |  |  |  |  |  |  | Impedance of leg right | Neale B | 0·02143 | 0·002275 | 4·646E-21 | 331,301 | 0 | 331,301 | European |
|  |  |  |  |  |  |  | Leg fat-free mass left | Neale B | -0·03267 | 0·001655 | 1·143E-86 | 331,258 | 0 | 331,258 | European |
|  |  |  |  |  |  |  | Leg fat-free mass right | Neale B | -0·03366 | 0·001654 | 6·021E-92 | 331,285 | 0 | 331,285 | European |
|  |  |  |  |  |  |  | Leg predicted mass left | Neale B | -0·03249 | 0·001644 | 6·675E-87 | 331,253 | 0 | 331,253 | European |
|  |  |  |  |  |  |  | Leg predicted mass right | Neale B | -0·03345 | 0·001644 | 5·315E-92 | 331,285 | 0 | 331,285 | European |
|  |  |  |  |  |  |  | Sitting height | Neale B | -0·05767 | 0·001941 | 1·176E-193 | 336,172 | 0 | 336,172 | European |
|  |  |  |  |  |  |  | Trunk fat-free mass | Neale B | -0·03554 | 0·001583 | 1·57E-111 | 331,030 | 0 | 331,030 | European |
|  |  |  |  |  |  |  | Trunk predicted mass | Neale B | -0·03537 | 0·001578 | 3·117E-111 | 330,995 | 0 | 330,995 | European |
|  |  |  |  |  |  |  | Weight | Neale B | -0·03004 | 0·002197 | 1·534E-42 | 336,227 | 0 | 336,227 | European |
|  |  |  |  |  |  |  | Whole body fat-free mass | Neale B | -0·03473 | 0·001589 | 8·511E-106 | 331,291 | 0 | 331,291 | European |
|  |  |  |  |  |  |  | Whole body water mass | Neale B | -0·0343 | 0·001592 | 5·927E-103 | 331,315 | 0 | 331,315 | European |
|  | *NSUN4* | 1 | ENSG00000117481 | rs41293273 | C | T | ·· | ·· | ·· | ·· | ·· | ·· | ·· | ·· | ·· |
|  | *SLC25A37* | 8 | ENSG00000147454 | rs2928682 | A | G | Mean corpuscular hemoglobin | Astle W | -0·03729 | 0·003965 | 5·201E-21 | 173,480 | 0 | 173,480 | European |
|  |  |  |  |  |  |  | Mean corpuscular hemoglobin concentration | Astle W | -0·02374 | 0·003877 | 9·17E-10 | 173,480 | 0 | 173,480 | European |
|  |  |  |  |  |  |  | Mean corpuscular volume | Astle W | -0·03176 | 0·003954 | 9·62E-16 | 173,480 | 0 | 173,480 | European |
|  |  |  |  |  |  |  | Red blood cell count | Astle W | 0·02606 | 0·003984 | 6·114E-11 | 173,480 | 0 | 173,480 | European |
|  |  |  |  |  |  |  | Red cell distribution width | Astle W | 0·03267 | 0·003962 | 1·648E-16 | 173,480 | 0 | 173,480 | European |
|  |  |  |  |  |  |  | Mean corpuscular hemoglobin | Astle W | -0·03963 | 0·003933 | 7·115E-24 | 173,480 | 0 | 173,480 | European |
|  |  |  |  |  |  |  | Mean corpuscular hemoglobin concentration | Astle W | -0·02472 | 0·003845 | 1·295E-10 | 173,480 | 0 | 173,480 | European |
|  |  |  |  |  |  |  | Mean corpuscular volume | Astle W | -0·03374 | 0·003922 | 7·868E-18 | 173,480 | 0 | 173,480 | European |
|  |  |  |  |  |  |  | Red blood cell count | Astle W | 0·02742 | 0·003951 | 3·933E-12 | 173,480 | 0 | 173,480 | European |
|  |  |  |  |  |  |  | Red cell distribution width | Astle W | 0·03469 | 0·003931 | 1·085E-18 | 173,480 | 0 | 173,480 | European |
|  |  |  |  |  |  |  | Mean corpuscular volume | Astle W | -0·03374 | 0·003923 | 8E-18 | NA | NA | NA | European |
|  |  |  |  |  |  |  | Red cell distribution width | Astle W | -0·03469 | 0·003927 | 1E-18 | NA | NA | NA | European |
|  |  |  | cg07972488 | rs2294413 | C | G | Platelet distribution width | Astle W | 0·03094 | 0·004059 | 2·49E-14 | 173,480 | 0 | 173,480 | European |
|  | *NSUN4* | 1 | cg04241075 | rs41293277 | T | C | ·· | ·· | ·· | ·· | ·· | ·· | ·· | ·· | ·· |
|  |  | 1 | cg17806798 |  |  |  | ·· | ·· | ·· | ·· | ·· | ·· | ·· | ·· | ·· |
|  |  | 1 | cg15580309 | rs111226885 | C | T | ·· | ·· | ·· | ·· | ·· | ·· | ·· | ·· | ·· |
|  | *NUDT5* | 10 | cg22687873 | rs4750175 | T | C | ·· | ·· | ·· | ·· | ·· | ·· | ·· | ·· | ·· |
|  | *TSPO* | 22 | cg13160331 | rs138909 | A | T | Monocyte count | Astle W | 0·0347 | 0·003661 | 2·631E-21 | 173,480 | 0 | 173,480 | European |
|  |  |  |  |  |  |  | Monocyte percentage of white cells | Astle W | 0·02558 | 0·003651 | 2·438E-12 | 173,480 | 0 | 173,480 | European |
|  | *VARS2* | 6 | cg02186769 | rs2233959 | C | T | ·· | ·· | ·· | ·· | ·· | ·· | ·· | ·· | ·· |
|  |  |  | cg05103231 | rs2524108 | G | C | Eosinophil count | Astle W | -0·03835 | 0·004008 | 1·085E-21 | 173,480 | 0 | 173,480 | European |
|  |  |  |  |  |  |  | Eosinophil percentage of granulocytes | Astle W | -0·02578 | 0·004022 | 1·449E-10 | 173,480 | 0 | 173,480 | European |
|  |  |  |  |  |  |  | Eosinophil percentage of white cells | Astle W | -0·02265 | 0·004004 | 1·551E-08 | 173,480 | 0 | 173,480 | European |
|  |  |  |  |  |  |  | Granulocyte count | Astle W | -0·02664 | 0·004035 | 4·02E-11 | 173,480 | 0 | 173,480 | European |
|  |  |  |  |  |  |  | Hemoglobin concentration | Astle W | -0·02603 | 0·003985 | 6·468E-11 | 173,480 | 0 | 173,480 | European |
|  |  |  |  |  |  |  | High light scatter percentage of red cells | Astle W | -0·03717 | 0·004028 | 2·727E-20 | 173,480 | 0 | 173,480 | European |
|  |  |  |  |  |  |  | High light scatter reticulocyte count | Astle W | -0·03931 | 0·004028 | 1·702E-22 | 173,480 | 0 | 173,480 | European |
|  |  |  |  |  |  |  | Lymphocyte count | Astle W | -0·06116 | 0·004046 | 1·287E-51 | 173,480 | 0 | 173,480 | European |
|  |  |  |  |  |  |  | Lymphocyte percentage of white cells | Astle W | -0·02475 | 0·004004 | 6·395E-10 | 173,480 | 0 | 173,480 | European |
|  |  |  |  |  |  |  | Monocyte count | Astle W | -0·04954 | 0·004014 | 5·489E-35 | 173,480 | 0 | 173,480 | European |
|  |  |  |  |  |  |  | Myeloid white cell count | Astle W | -0·03082 | 0·004045 | 2·556E-14 | 173,480 | 0 | 173,480 | European |
|  |  |  |  |  |  |  | Neutrophil count | Astle W | -0·02316 | 0·004025 | 8·661E-09 | 173,480 | 0 | 173,480 | European |
|  |  |  |  |  |  |  | Neutrophil percentage of granulocytes | Astle W | 0·024 | 0·004022 | 2·396E-09 | 173,480 | 0 | 173,480 | European |
|  |  |  |  |  |  |  | Neutrophil percentage of white cells | Astle W | 0·02954 | 0·00401 | 1·755E-13 | 173,480 | 0 | 173,480 | European |
|  |  |  |  |  |  |  | Reticulocyte count | Astle W | -0·04502 | 0·004035 | 6·592E-29 | 173,480 | 0 | 173,480 | European |
|  |  |  |  |  |  |  | Reticulocyte fraction of red cells | Astle W | -0·04309 | 0·004032 | 1·176E-26 | 173,480 | 0 | 173,480 | European |
|  |  |  |  |  |  |  | Sum basophil neutrophil counts | Astle W | -0·02355 | 0·004031 | 5·181E-09 | 173,480 | 0 | 173,480 | European |
|  |  |  |  |  |  |  | Sum eosinophil basophil counts | Astle W | -0·03775 | 0·004012 | 4·929E-21 | 173,480 | 0 | 173,480 | European |
|  |  |  |  |  |  |  | Sum neutrophil eosinophil counts | Astle W | -0·02627 | 0·004029 | 6·973E-11 | 173,480 | 0 | 173,480 | European |
|  |  |  |  |  |  |  | White blood cell count | Astle W | -0·04999 | 0·004027 | 2·23E-35 | 173,480 | 0 | 173,480 | European |
|  |  |  |  |  |  |  | IgA deficiency | Bronson P | 0·82 | 0·05026 | 7·592E-60 | 6487 | 1635 | 4852 | European |
|  |  |  |  |  |  |  | Primary sclerosing cholangitis | Ji S | 0·6323 | 0·031 | 8·551E-100 | 14,890 | 2871 | 12,019 | European |
|  |  |  |  |  |  |  | Started insulin within one year diagnosis of diabetes | Neale B | 0·02376 | 0·004149 | 1·035E-08 | 15,397 | 1872 | 13,525 | European |
|  |  |  |  |  |  |  | Schizophrenia | PGC | -0·081 | 0·0134 | 1·342E-09 | 82,315 | 35,476 | 46,839 | Mixed |
|  |  |  | cg12457901 | rs2233980 | A | G | Basophil count | Astle W | 0·04095 | 0·00485 | 3·074E-17 | 173,480 | 0 | 173,480 | European |
|  |  |  |  |  |  |  | Eosinophil count | Astle W | 0·0612 | 0·004944 | 3·379E-35 | 173,480 | 0 | 173,480 | European |
|  |  |  |  |  |  |  | Eosinophil percentage of granulocytes | Astle W | 0·03285 | 0·004959 | 3·472E-11 | 173,480 | 0 | 173,480 | European |
|  |  |  |  |  |  |  | Eosinophil percentage of white cells | Astle W | 0·03128 | 0·004938 | 2·396E-10 | 173,480 | 0 | 173,480 | European |
|  |  |  |  |  |  |  | Granulocyte count | Astle W | 0·06505 | 0·004976 | 4·734E-39 | 173,480 | 0 | 173,480 | European |
|  |  |  |  |  |  |  | Hematocrit | Astle W | 0·0359 | 0·004894 | 2·205E-13 | 173,480 | 0 | 173,480 | European |
|  |  |  |  |  |  |  | Hemoglobin concentration | Astle W | 0·03973 | 0·004916 | 6·433E-16 | 173,480 | 0 | 173,480 | European |
|  |  |  |  |  |  |  | High light scatter percentage of red cells | Astle W | 0·03376 | 0·004969 | 1·093E-11 | 173,480 | 0 | 173,480 | European |
|  |  |  |  |  |  |  | High light scatter reticulocyte count | Astle W | 0·03941 | 0·00497 | 2·205E-15 | 173,480 | 0 | 173,480 | European |
|  |  |  |  |  |  |  | Lymphocyte count | Astle W | 0·09418 | 0·004989 | 1·777E-79 | 173,480 | 0 | 173,480 | European |
|  |  |  |  |  |  |  | Monocyte count | Astle W | 0·07309 | 0·00495 | 2·511E-49 | 173,480 | 0 | 173,480 | European |
|  |  |  |  |  |  |  | Myeloid white cell count | Astle W | 0·0694 | 0·004988 | 5·268E-44 | 173,480 | 0 | 173,480 | European |
|  |  |  |  |  |  |  | Neutrophil count | Astle W | 0·05881 | 0·004963 | 2·157E-32 | 173,480 | 0 | 173,480 | European |
|  |  |  |  |  |  |  | Neutrophil percentage of granulocytes | Astle W | -0·03295 | 0·004959 | 3·06E-11 | 173,480 | 0 | 173,480 | European |
|  |  |  |  |  |  |  | Platelet count | Astle W | 0·04141 | 0·005056 | 2·589E-16 | 173,480 | 0 | 173,480 | European |
|  |  |  |  |  |  |  | Plateletcrit | Astle W | 0·03569 | 0·005071 | 1·938E-12 | 173,480 | 0 | 173,480 | European |
|  |  |  |  |  |  |  | Red blood cell count | Astle W | 0·03571 | 0·004923 | 4·046E-13 | 173,480 | 0 | 173,480 | European |
|  |  |  |  |  |  |  | Red cell distribution width | Astle W | -0·02767 | 0·004908 | 1·73E-08 | 173,480 | 0 | 173,480 | European |
|  |  |  |  |  |  |  | Reticulocyte count | Astle W | 0·05489 | 0·004978 | 2·898E-28 | 173,480 | 0 | 173,480 | European |
|  |  |  |  |  |  |  | Reticulocyte fraction of red cells | Astle W | 0·04752 | 0·004975 | 1·275E-21 | 173,480 | 0 | 173,480 | European |
|  |  |  |  |  |  |  | Sum basophil neutrophil counts | Astle W | 0·06001 | 0·004972 | 1·523E-33 | 173,480 | 0 | 173,480 | European |
|  |  |  |  |  |  |  | Sum eosinophil basophil counts | Astle W | 0·06565 | 0·004949 | 3·642E-40 | 173,480 | 0 | 173,480 | European |
|  |  |  |  |  |  |  | Sum neutrophil eosinophil counts | Astle W | 0·06377 | 0·004968 | 1·023E-37 | 173,480 | 0 | 173,480 | European |
|  |  |  |  |  |  |  | White blood cell count | Astle W | 0·09439 | 0·004968 | 1·736E-80 | 173,480 | 0 | 173,480 | European |
|  |  |  |  |  |  |  | IgA deficiency | Bronson P | -1·192 | 0·06128 | 2·831E-84 | 6487 | 1635 | 4852 | European |
|  |  |  |  |  |  |  | Primary sclerosing cholangitis | Ji S | -1·094 | 0·036 | 4·586E-219 | 14,890 | 2871 | 12,019 | European |
|  |  |  |  |  |  |  | Basal metabolic rate | Neale B | -0·0212 | 0·002228 | 1·788E-21 | 331,307 | 0 | 331,307 | European |
|  |  |  |  |  |  |  | Diastolic blood pressure | Neale B | 0·02182 | 0·003411 | 1·588E-10 | 317,756 | 0 | 317,756 | European |
|  |  |  |  |  |  |  | Forced expiratory volume in 1-second· predicted percentage | Neale B | 0·04109 | 0·005843 | 2·039E-12 | 110,423 | 0 | 110,423 | European |
|  |  |  |  |  |  |  | Height | Neale B | -0·028 | 0·002387 | 9·081E-32 | 336,474 | 0 | 336,474 | European |
|  |  |  |  |  |  |  | Hip circumference | Neale B | -0·02293 | 0·003327 | 5·482E-12 | 336,601 | 0 | 336,601 | European |
|  |  |  |  |  |  |  | Leg fat-free mass left | Neale B | -0·02166 | 0·002212 | 1·231E-22 | 331,258 | 0 | 331,258 | European |
|  |  |  |  |  |  |  | Leg fat-free mass right | Neale B | -0·0225 | 0·002212 | 2·608E-24 | 331,285 | 0 | 331,285 | European |
|  |  |  |  |  |  |  | Leg predicted mass left | Neale B | -0·02153 | 0·002197 | 1·162E-22 | 331,253 | 0 | 331,253 | European |
|  |  |  |  |  |  |  | Leg predicted mass right | Neale B | -0·02219 | 0·002197 | 5·572E-24 | 331,285 | 0 | 331,285 | European |
|  |  |  |  |  |  |  | Sitting height | Neale B | -0·03652 | 0·002596 | 6·411E-45 | 336,172 | 0 | 336,172 | European |
|  |  |  |  |  |  |  | Started insulin within one year diagnosis of diabetes | Neale B | -0·05378 | 0·00501 | 8·717E-27 | 15,397 | 1872 | 13,525 | European |
|  |  |  |  |  |  |  | Weight | Neale B | -0·02359 | 0·002936 | 9·403E-16 | 336,227 | 0 | 336,227 | European |
|  |  |  |  |  |  |  | Whole body fat-free mass | Neale B | -0·02065 | 0·002125 | 2·523E-22 | 331,291 | 0 | 331,291 | European |
|  |  |  |  |  |  |  | Whole body water mass | Neale B | -0·02058 | 0·002128 | 3·906E-22 | 331,315 | 0 | 331,315 | European |
|  |  |  |  |  |  |  | Schizophrenia | PGC | 0·149 | 0·0173 | 6·997E-18 | 82,315 | 35,476 | 46,839 | Mixed |
|  |  |  |  |  |  |  | Eosinophil count | Astle W | -0·03748 | 0·004252 | 1·198E-18 | 173,480 | 0 | 173,480 | European |
|  |  |  |  |  |  |  | Eosinophil percentage of granulocytes | Astle W | -0·02342 | 0·004266 | 4·04E-08 | 173,480 | 0 | 173,480 | European |
|  |  |  |  |  |  |  | Granulocyte count | Astle W | -0·03234 | 0·00428 | 4·173E-14 | 173,480 | 0 | 173,480 | European |
|  |  |  |  |  |  |  | Hematocrit | Astle W | -0·03011 | 0·004208 | 8·318E-13 | 173,480 | 0 | 173,480 | European |
|  |  |  |  |  |  |  | Hemoglobin concentration | Astle W | -0·03164 | 0·004227 | 7·221E-14 | 173,480 | 0 | 173,480 | European |
|  |  |  |  |  |  |  | High light scatter percentage of red cells | Astle W | -0·03515 | 0·004273 | 1·942E-16 | 173,480 | 0 | 173,480 | European |
|  |  |  |  |  |  |  | High light scatter reticulocyte count | Astle W | -0·03992 | 0·004273 | 9·447E-21 | 173,480 | 0 | 173,480 | European |
|  |  |  |  |  |  |  | Lymphocyte count | Astle W | -0·06171 | 0·004292 | 6·951E-47 | 173,480 | 0 | 173,480 | European |
|  |  |  |  |  |  |  | Mean platelet volume | Astle W | 0·03223 | 0·00432 | 8·629E-14 | 173,480 | 0 | 173,480 | European |
|  |  |  |  |  |  |  | Monocyte count | Astle W | -0·03211 | 0·004259 | 4·761E-14 | 173,480 | 0 | 173,480 | European |
|  |  |  |  |  |  |  | Myeloid white cell count | Astle W | -0·03353 | 0·004291 | 5·532E-15 | 173,480 | 0 | 173,480 | European |
|  |  |  |  |  |  |  | Neutrophil count | Astle W | -0·02823 | 0·004269 | 3·796E-11 | 173,480 | 0 | 173,480 | European |
|  |  |  |  |  |  |  | Neutrophil percentage of granulocytes | Astle W | 0·02338 | 0·004267 | 4·253E-08 | 173,480 | 0 | 173,480 | European |
|  |  |  |  |  |  |  | Platelet count | Astle W | -0·03995 | 0·00435 | 4·102E-20 | 173,480 | 0 | 173,480 | European |
|  |  |  |  |  |  |  | Plateletcrit | Astle W | -0·0286 | 0·004362 | 5·497E-11 | 173,480 | 0 | 173,480 | European |
|  |  |  |  |  |  |  | Red blood cell count | Astle W | -0·03033 | 0·004233 | 7·816E-13 | 173,480 | 0 | 173,480 | European |
|  |  |  |  |  |  |  | Reticulocyte count | Astle W | -0·05031 | 0·004281 | 6·8E-32 | 173,480 | 0 | 173,480 | European |
|  |  |  |  |  |  |  | Reticulocyte fraction of red cells | Astle W | -0·04402 | 0·004278 | 7·68E-25 | 173,480 | 0 | 173,480 | European |
|  |  |  |  |  |  |  | Sum basophil neutrophil counts | Astle W | -0·02924 | 0·004276 | 7·992E-12 | 173,480 | 0 | 173,480 | European |
|  |  |  |  |  |  |  | Sum eosinophil basophil counts | Astle W | -0·03964 | 0·004256 | 1·239E-20 | 173,480 | 0 | 173,480 | European |
|  |  |  |  |  |  |  | Sum neutrophil eosinophil counts | Astle W | -0·03129 | 0·004274 | 2·435E-13 | 173,480 | 0 | 173,480 | European |
|  |  |  |  |  |  |  | White blood cell count | Astle W | -0·0518 | 0·004272 | 7·762E-34 | 173,480 | 0 | 173,480 | European |
|  |  |  |  |  |  |  | IgA deficiency | Bronson P | 1·13 | 0·0517 | 6·94E-106 | 6487 | 1635 | 4852 | European |
|  |  |  |  |  |  |  | Primary sclerosing cholangitis | Ji S | 0·7701 | 0·037 | 3·171E-104 | 14,890 | 2871 | 12,019 | European |
|  |  |  |  |  |  |  | Forced expiratory volume in 1-second· predicted percentage | Neale B | -0·03359 | 0·005074 | 3·617E-11 | 110,423 | 0 | 110,423 | European |
|  |  |  |  |  |  |  | Sitting height | Neale B | 0·02287 | 0·002248 | 2·681E-24 | 336,172 | 0 | 336,172 | European |
|  |  |  |  |  |  |  | Started insulin within one year diagnosis of diabetes | Neale B | 0·04372 | 0·004378 | 2·062E-23 | 15,397 | 1872 | 13,525 | European |
|  |  |  |  |  |  |  | Schizophrenia | PGC | -0·1035 | 0·0141 | 2·523E-13 | 82,315 | 35,476 | 46,839 | Mixed |
|  |  |  | cg26467571 | rs9264490 | G | A | Eosinophil count | Astle W | 0·04161 | 0·00402 | 4·16E-25 | 173,480 | 0 | 173,480 | European |
|  |  |  |  |  |  |  | Eosinophil percentage of granulocytes | Astle W | 0·02857 | 0·004034 | 1·405E-12 | 173,480 | 0 | 173,480 | European |
|  |  |  |  |  |  |  | Eosinophil percentage of white cells | Astle W | 0·02572 | 0·004017 | 1·526E-10 | 173,480 | 0 | 173,480 | European |
|  |  |  |  |  |  |  | Granulocyte count | Astle W | 0·02724 | 0·004047 | 1·679E-11 | 173,480 | 0 | 173,480 | European |
|  |  |  |  |  |  |  | Hemoglobin concentration | Astle W | 0·02653 | 0·003998 | 3·201E-11 | 173,480 | 0 | 173,480 | European |
|  |  |  |  |  |  |  | High light scatter percentage of red cells | Astle W | 0·03715 | 0·004041 | 3·816E-20 | 173,480 | 0 | 173,480 | European |
|  |  |  |  |  |  |  | High light scatter reticulocyte count | Astle W | 0·03942 | 0·004041 | 1·742E-22 | 173,480 | 0 | 173,480 | European |
|  |  |  |  |  |  |  | Lymphocyte count | Astle W | 0·06154 | 0·004058 | 6·22E-52 | 173,480 | 0 | 173,480 | European |
|  |  |  |  |  |  |  | Lymphocyte percentage of white cells | Astle W | 0·02467 | 0·004016 | 8·076E-10 | 173,480 | 0 | 173,480 | European |
|  |  |  |  |  |  |  | Monocyte count | Astle W | 0·05091 | 0·004027 | 1·212E-36 | 173,480 | 0 | 173,480 | European |
|  |  |  |  |  |  |  | Myeloid white cell count | Astle W | 0·03142 | 0·004057 | 9·56E-15 | 173,480 | 0 | 173,480 | European |
|  |  |  |  |  |  |  | Neutrophil count | Astle W | 0·02342 | 0·004037 | 6·553E-09 | 173,480 | 0 | 173,480 | European |
|  |  |  |  |  |  |  | Neutrophil percentage of granulocytes | Astle W | -0·02705 | 0·004034 | 2·011E-11 | 173,480 | 0 | 173,480 | European |
|  |  |  |  |  |  |  | Neutrophil percentage of white cells | Astle W | -0·03027 | 0·004023 | 5·321E-14 | 173,480 | 0 | 173,480 | European |
|  |  |  |  |  |  |  | Platelet count | Astle W | 0·02524 | 0·00411 | 8·241E-10 | 173,480 | 0 | 173,480 | European |
|  |  |  |  |  |  |  | Reticulocyte count | Astle W | 0·04595 | 0·004048 | 7·246E-30 | 173,480 | 0 | 173,480 | European |
|  |  |  |  |  |  |  | Reticulocyte fraction of red cells | Astle W | 0·04367 | 0·004045 | 3·621E-27 | 173,480 | 0 | 173,480 | European |
|  |  |  |  |  |  |  | Sum basophil neutrophil counts | Astle W | 0·024 | 0·004044 | 2·94E-09 | 173,480 | 0 | 173,480 | European |
|  |  |  |  |  |  |  | Sum eosinophil basophil counts | Astle W | 0·04113 | 0·004024 | 1·613E-24 | 173,480 | 0 | 173,480 | European |
|  |  |  |  |  |  |  | Sum neutrophil eosinophil counts | Astle W | 0·02667 | 0·004041 | 4·116E-11 | 173,480 | 0 | 173,480 | European |
|  |  |  |  |  |  |  | White blood cell count | Astle W | 0·05042 | 0·00404 | 9·447E-36 | 173,480 | 0 | 173,480 | European |
|  |  |  |  |  |  |  | IgA deficiency | Bronson P | -0·8303 | 0·05059 | 1·504E-60 | 6487 | 1635 | 4852 | European |
|  |  |  |  |  |  |  | Primary sclerosing cholangitis | Ji S | -0·644 | 0·031 | 5·875E-104 | 14,890 | 2871 | 12,019 | European |
|  |  |  |  |  |  |  | Hip circumference | Neale B | -0·02038 | 0·002731 | 8·473E-14 | 336,601 | 0 | 336,601 | European |
|  |  |  |  |  |  |  | Sitting height | Neale B | -0·02001 | 0·002132 | 6·404E-21 | 336,172 | 0 | 336,172 | European |
|  |  |  |  |  |  |  | Started insulin within one year diagnosis of diabetes | Neale B | -0·02635 | 0·004186 | 3·165E-10 | 15,397 | 1872 | 13,525 | European |
|  |  |  |  |  |  |  | Schizophrenia | PGC | 0·0836 | 0·0135 | 6·683E-10 | 82,315 | 35,476 | 46,839 | Mixed |
|  |  |  |  |  |  |  |  |  |  |  |  |  |  |  |  |
| Gastric cancer | *BAK1* | 6 | ENSG00000030110 | rs210143 | T | C | ·· | ·· | ·· | ·· | ·· | ·· | ·· | ·· | ·· |
|  |  |  | cg00700324 | rs511515 | A | G | Eosinophil count | Astle W | -0·03323 | 0·003885 | 1·197E-17 | 173,480 | 0 | 173,480 | European |
|  |  |  |  |  |  |  | Eosinophil percentage of granulocytes | Astle W | -0·03033 | 0·003898 | 7·174E-15 | 173,480 | 0 | 173,480 | European |
|  |  |  |  |  |  |  | Eosinophil percentage of white cells | Astle W | -0·03274 | 0·003882 | 3·334E-17 | 173,480 | 0 | 173,480 | European |
|  |  |  |  |  |  |  | Neutrophil percentage of granulocytes | Astle W | 0·03056 | 0·003898 | 4·498E-15 | 173,480 | 0 | 173,480 | European |
|  |  |  |  |  |  |  | Platelet count | Astle W | -0·1017 | 0·003971 | 1·49E-144 | 173,480 | 0 | 173,480 | European |
|  |  |  |  |  |  |  | Plateletcrit | Astle W | -0·1109 | 0·003981 | 9·54E-171 | 173,480 | 0 | 173,480 | European |
|  |  |  |  |  |  |  | Sum eosinophil basophil counts | Astle W | -0·03419 | 0·003889 | 1·466E-18 | 173,480 | 0 | 173,480 | European |
|  |  |  |  |  |  |  | Eosinophil counts | Astle W | -0·03323 | 0·003876 | 1E-17 | NA | NA | NA | European |
|  |  |  |  |  |  |  | Eosinophil percentage of white cells | Astle W | -0·03274 | 0·003877 | 3E-17 | NA | NA | NA | European |
|  |  |  |  |  |  |  | Sum eosinophil basophil counts | Astle W | -0·03419 | 0·00387 | 1E-18 | NA | NA | NA | European |
|  |  |  |  |  |  |  | Eosinophil count | Astle W | -0·0302 | 0·003837 | 3·463E-15 | 173,480 | 0 | 173,480 | European |
|  |  |  |  |  |  |  | Eosinophil percentage of granulocytes | Astle W | -0·02724 | 0·003848 | 1·466E-12 | 173,480 | 0 | 173,480 | European |
|  |  |  |  |  |  |  | Eosinophil percentage of white cells | Astle W | -0·02938 | 0·003833 | 1·806E-14 | 173,480 | 0 | 173,480 | European |
|  |  |  |  |  |  |  | Neutrophil percentage of granulocytes | Astle W | 0·02739 | 0·003849 | 1·104E-12 | 173,480 | 0 | 173,480 | European |
|  |  |  |  |  |  |  | Platelet count | Astle W | -0·09797 | 0·003921 | 9·06E-138 | 173,480 | 0 | 173,480 | European |
|  |  |  |  |  |  |  | Plateletcrit | Astle W | -0·1075 | 0·003931 | 1·14E-164 | 173,480 | 0 | 173,480 | European |
|  |  |  |  |  |  |  | Sum eosinophil basophil counts | Astle W | -0·0312 | 0·00384 | 4·485E-16 | 173,480 | 0 | 173,480 | European |
|  |  |  |  |  |  |  | Mean platelet volume | Qayyum R | -6·16 | 0·7757 | 2E-15 | NA | NA | NA | African |
|  |  |  |  |  |  |  | Platelet count | Schick UM | 4·94 | 0·8593 | 9E-09 | NA | NA | NA | Hispanic |
|  |  |  |  |  |  |  | Platelet counts | Gieger C | -4·957 | 0·3964 | 7E-36 | NA | NA | NA | European |
|  |  |  |  |  |  |  | Platelet counts | Li J | 8·923 | 1·59 | 2E-08 | NA | NA | NA | Mixed |
|  |  |  |  |  |  |  | Rheumatoid arthritis | Okada Y | -0·09431 | 0·01683 | 9·1E-09 | 58 284 | 14 361 | 43 923 | European |
|  |  |  |  |  |  |  |  |  |  |  |  |  |  |  |  |
| Lung cancer |  |  | cg12457901 | rs2523593 | C | T | Basophil count | Astle W | 0·04645 | 0·005011 | 1·862E-20 | 173,480 | 0 | 173,480 | European |
|  |  |  |  |  |  |  | Eosinophil count | Astle W | 0·06752 | 0·005108 | 7·021E-40 | 173,480 | 0 | 173,480 | European |
|  |  |  |  |  |  |  | Eosinophil percentage of granulocytes | Astle W | 0·03589 | 0·005125 | 2·52E-12 | 173,480 | 0 | 173,480 | European |
|  |  |  |  |  |  |  | Eosinophil percentage of white cells | Astle W | 0·03341 | 0·005103 | 5·843E-11 | 173,480 | 0 | 173,480 | European |
|  |  |  |  |  |  |  | Granulocyte count | Astle W | 0·07208 | 0·005142 | 1·24E-44 | 173,480 | 0 | 173,480 | European |
|  |  |  |  |  |  |  | Hematocrit | Astle W | 0·03788 | 0·005056 | 6·805E-14 | 173,480 | 0 | 173,480 | European |
|  |  |  |  |  |  |  | Hemoglobin concentration | Astle W | 0·04404 | 0·00508 | 4·359E-18 | 173,480 | 0 | 173,480 | European |
|  |  |  |  |  |  |  | High light scatter percentage of red cells | Astle W | 0·04281 | 0·005135 | 7·597E-17 | 173,480 | 0 | 173,480 | European |
|  |  |  |  |  |  |  | High light scatter reticulocyte count | Astle W | 0·04891 | 0·005135 | 1·64E-21 | 173,480 | 0 | 173,480 | European |
|  |  |  |  |  |  |  | Lymphocyte count | Astle W | 0·111 | 0·005156 | 9·79E-103 | 173,480 | 0 | 173,480 | European |
|  |  |  |  |  |  |  | Lymphocyte percentage of white cells | Astle W | 0·02834 | 0·005102 | 2·772E-08 | 173,480 | 0 | 173,480 | European |
|  |  |  |  |  |  |  | Monocyte count | Astle W | 0·08306 | 0·005115 | 2·758E-59 | 173,480 | 0 | 173,480 | European |
|  |  |  |  |  |  |  | Myeloid white cell count | Astle W | 0·07742 | 0·005155 | 5·565E-51 | 173,480 | 0 | 173,480 | European |
|  |  |  |  |  |  |  | Neutrophil count | Astle W | 0·06524 | 0·005129 | 4·614E-37 | 173,480 | 0 | 173,480 | European |
|  |  |  |  |  |  |  | Neutrophil percentage of granulocytes | Astle W | -0·03598 | 0·005125 | 2·208E-12 | 173,480 | 0 | 173,480 | European |
|  |  |  |  |  |  |  | Neutrophil percentage of white cells | Astle W | -0·03266 | 0·00511 | 1·656E-10 | 173,480 | 0 | 173,480 | European |
|  |  |  |  |  |  |  | Platelet count | Astle W | 0·0466 | 0·005225 | 4·71E-19 | 173,480 | 0 | 173,480 | European |
|  |  |  |  |  |  |  | Plateletcrit | Astle W | 0·0404 | 0·00524 | 1·269E-14 | 173,480 | 0 | 173,480 | European |
|  |  |  |  |  |  |  | Red blood cell count | Astle W | 0·03746 | 0·005087 | 1·797E-13 | 173,480 | 0 | 173,480 | European |
|  |  |  |  |  |  |  | Red cell distribution width | Astle W | -0·03277 | 0·005071 | 1·034E-10 | 173,480 | 0 | 173,480 | European |
|  |  |  |  |  |  |  | Reticulocyte count | Astle W | 0·06685 | 0·005144 | 1·278E-38 | 173,480 | 0 | 173,480 | European |
|  |  |  |  |  |  |  | Reticulocyte fraction of red cells | Astle W | 0·05908 | 0·00514 | 1·424E-30 | 173,480 | 0 | 173,480 | European |
|  |  |  |  |  |  |  | Sum basophil neutrophil counts | Astle W | 0·06646 | 0·005138 | 2·804E-38 | 173,480 | 0 | 173,480 | European |
|  |  |  |  |  |  |  | Sum eosinophil basophil counts | Astle W | 0·07269 | 0·005114 | 7·487E-46 | 173,480 | 0 | 173,480 | European |
|  |  |  |  |  |  |  | Sum neutrophil eosinophil counts | Astle W | 0·07083 | 0·005134 | 2·669E-43 | 173,480 | 0 | 173,480 | European |
|  |  |  |  |  |  |  | IgA deficiency | Bronson P | -1·245 | 0·0618 | 3·307E-90 | 6487 | 1635 | 4852 | European |
|  |  |  |  |  |  |  | Primary sclerosing cholangitis | Ji S | -1·207 | 0·044 | 7·158E-190 | 14,890 | 2871 | 12,019 | European |
|  |  |  |  |  |  |  | Arm fat-free mass left | Neale B | -0·02058 | 0·002194 | 6·76E-21 | 331,159 | 0 | 331,159 | European |
|  |  |  |  |  |  |  | Arm fat-free mass right | Neale B | -0·02002 | 0·00215 | 1·266E-20 | 331,221 | 0 | 331,221 | European |
|  |  |  |  |  |  |  | Arm predicted mass left | Neale B | -0·02091 | 0·002186 | 1·114E-21 | 331,146 | 0 | 331,146 | European |
|  |  |  |  |  |  |  | Basal metabolic rate | Neale B | -0·02376 | 0·002288 | 3·027E-25 | 331,307 | 0 | 331,307 | European |
|  |  |  |  |  |  |  | Diastolic blood pressure | Neale B | 0·02544 | 0·003503 | 3·838E-13 | 317,756 | 0 | 317,756 | European |
|  |  |  |  |  |  |  | Forced expiratory volume in 1-second· predicted percentage | Neale B | 0·04226 | 0·006005 | 1·968E-12 | 110,423 | 0 | 110,423 | European |
|  |  |  |  |  |  |  | Height | Neale B | -0·03119 | 0·002452 | 4·488E-37 | 336,474 | 0 | 336,474 | European |
|  |  |  |  |  |  |  | Hip circumference | Neale B | -0·02516 | 0·003418 | 1·803E-13 | 336,601 | 0 | 336,601 | European |
|  |  |  |  |  |  |  | Leg fat-free mass left | Neale B | -0·02418 | 0·002272 | 1·972E-26 | 331,258 | 0 | 331,258 | European |
|  |  |  |  |  |  |  | Leg fat-free mass right | Neale B | -0·02495 | 0·002271 | 4·698E-28 | 331,285 | 0 | 331,285 | European |
|  |  |  |  |  |  |  | Leg predicted mass left | Neale B | -0·02395 | 0·002257 | 2·624E-26 | 331,253 | 0 | 331,253 | European |
|  |  |  |  |  |  |  | Leg predicted mass right | Neale B | -0·02461 | 0·002257 | 1·119E-27 | 331,285 | 0 | 331,285 | European |
|  |  |  |  |  |  |  | Number of days or week of moderate physical activity 10+ minutes | Neale B | 0·04523 | 0·008261 | 4·371E-08 | 321,309 | 0 | 321,309 | European |
|  |  |  |  |  |  |  | Sitting height | Neale B | -0·04223 | 0·002667 | 1·867E-56 | 336,172 | 0 | 336,172 | European |
|  |  |  |  |  |  |  | Started insulin within one year diagnosis of diabetes | Neale B | -0·06636 | 0·005108 | 2·179E-38 | 15,397 | 1872 | 13,525 | European |
|  |  |  |  |  |  |  | Trunk fat-free mass | Neale B | -0·02236 | 0·002174 | 8·354E-25 | 331,030 | 0 | 331,030 | European |
|  |  |  |  |  |  |  | Trunk predicted mass | Neale B | -0·02217 | 0·002167 | 1·42E-24 | 330,995 | 0 | 330,995 | European |
|  |  |  |  |  |  |  | Weight | Neale B | -0·02588 | 0·003016 | 9·226E-18 | 336,227 | 0 | 336,227 | European |
|  |  |  |  |  |  |  | Whole body fat-free mass | Neale B | -0·02349 | 0·002182 | 5·106E-27 | 331,291 | 0 | 331,291 | European |
|  |  |  |  |  |  |  | Whole body water mass | Neale B | -0·02342 | 0·002185 | 8·772E-27 | 331,315 | 0 | 331,315 | European |
|  |  |  |  |  |  |  | Schizophrenia | PGC | 0·1685 | 0·0183 | 3·892E-20 | 82,315 | 35,476 | 46,839 | Mixed |
|  |  |  | cg14935711 |  |  |  | ·· | ·· | ·· | ·· | ·· | ·· | ·· | ·· | ·· |
|  |  |  | cg15848685 | rs2596500 | C | A | Basophil count | Astle W | -0·04491 | 0·004986 | 2·145E-19 | 173,480 | 0 | 173,480 | European |
|  |  |  |  |  |  |  | Eosinophil count | Astle W | -0·06747 | 0·005083 | 3·349E-40 | 173,480 | 0 | 173,480 | European |
|  |  |  |  |  |  |  | Eosinophil percentage of granulocytes | Astle W | -0·03577 | 0·0051 | 2·324E-12 | 173,480 | 0 | 173,480 | European |
|  |  |  |  |  |  |  | Eosinophil percentage of white cells | Astle W | -0·03337 | 0·005078 | 4·994E-11 | 173,480 | 0 | 173,480 | European |
|  |  |  |  |  |  |  | Granulocyte count | Astle W | -0·07204 | 0·005117 | 5·129E-45 | 173,480 | 0 | 173,480 | European |
|  |  |  |  |  |  |  | Hematocrit | Astle W | -0·03801 | 0·005032 | 4·221E-14 | 173,480 | 0 | 173,480 | European |
|  |  |  |  |  |  |  | Hemoglobin concentration | Astle W | -0·04403 | 0·005055 | 3·005E-18 | 173,480 | 0 | 173,480 | European |
|  |  |  |  |  |  |  | High light scatter percentage of red cells | Astle W | -0·04193 | 0·005109 | 2·266E-16 | 173,480 | 0 | 173,480 | European |
|  |  |  |  |  |  |  | High light scatter reticulocyte count | Astle W | -0·04798 | 0·005109 | 6·025E-21 | 173,480 | 0 | 173,480 | European |
|  |  |  |  |  |  |  | Lymphocyte count | Astle W | -0·1109 | 0·005131 | 1·59E-103 | 173,480 | 0 | 173,480 | European |
|  |  |  |  |  |  |  | Lymphocyte percentage of white cells | Astle W | -0·02822 | 0·005077 | 2·71E-08 | 173,480 | 0 | 173,480 | European |
|  |  |  |  |  |  |  | Monocyte count | Astle W | -0·08207 | 0·00509 | 1·753E-58 | 173,480 | 0 | 173,480 | European |
|  |  |  |  |  |  |  | Myeloid white cell count | Astle W | -0·07737 | 0·00513 | 2·066E-51 | 173,480 | 0 | 173,480 | European |
|  |  |  |  |  |  |  | Neutrophil count | Astle W | -0·06539 | 0·005104 | 1·409E-37 | 173,480 | 0 | 173,480 | European |
|  |  |  |  |  |  |  | Neutrophil percentage of granulocytes | Astle W | 0·03538 | 0·0051 | 3·974E-12 | 173,480 | 0 | 173,480 | European |
|  |  |  |  |  |  |  | Neutrophil percentage of white cells | Astle W | 0·03219 | 0·005085 | 2·463E-10 | 173,480 | 0 | 173,480 | European |
|  |  |  |  |  |  |  | Platelet count | Astle W | -0·04524 | 0·0052 | 3·341E-18 | 173,480 | 0 | 173,480 | European |
|  |  |  |  |  |  |  | Plateletcrit | Astle W | -0·03862 | 0·005216 | 1·321E-13 | 173,480 | 0 | 173,480 | European |
|  |  |  |  |  |  |  | Red blood cell count | Astle W | -0·03752 | 0·005062 | 1·25E-13 | 173,480 | 0 | 173,480 | European |
|  |  |  |  |  |  |  | Red cell distribution width | Astle W | 0·03301 | 0·005047 | 6·108E-11 | 173,480 | 0 | 173,480 | European |
|  |  |  |  |  |  |  | Reticulocyte count | Astle W | -0·06573 | 0·005118 | 9·629E-38 | 173,480 | 0 | 173,480 | European |
|  |  |  |  |  |  |  | Reticulocyte fraction of red cells | Astle W | -0·05795 | 0·005115 | 9·257E-30 | 173,480 | 0 | 173,480 | European |
|  |  |  |  |  |  |  | Sum basophil neutrophil counts | Astle W | -0·06648 | 0·005113 | 1·164E-38 | 173,480 | 0 | 173,480 | European |
|  |  |  |  |  |  |  | Sum eosinophil basophil counts | Astle W | -0·07201 | 0·005089 | 1·854E-45 | 173,480 | 0 | 173,480 | European |
|  |  |  |  |  |  |  | Sum neutrophil eosinophil counts | Astle W | -0·07091 | 0·005109 | 8·302E-44 | 173,480 | 0 | 173,480 | European |
|  |  |  |  |  |  |  | White blood cell count | Astle W | -0·1074 | 0·005108 | 3·8E-98 | 173,480 | 0 | 173,480 | European |
|  |  |  |  |  |  |  | IgA deficiency | Bronson P | 1·245 | 0·06168 | 1·434E-90 | 6487 | 1635 | 4852 | European |
|  |  |  |  |  |  |  | Primary sclerosing cholangitis | Ji S | 1·159 | 0·037 | 7·619E-244 | 14,890 | 2871 | 12,019 | European |
|  |  |  |  |  |  |  | Lung cancer in ever smokers | McKay JD | 0·1902 | 0·02394 | 2E-15 | NA | NA | NA | European |
|  |  |  |  |  |  |  | Schizophrenia | Goes FS | -0·1655 | 0·01816 | 8E-20 | NA | NA | NA | European |
|  |  |  |  |  |  |  | Arm predicted mass left | Neale B | 0·02014 | 0·002182 | 2·696E-20 | 331,146 | 0 | 331,146 | European |
|  |  |  |  |  |  |  | Basal metabolic rate | Neale B | 0·02289 | 0·002284 | 1·23E-23 | 331,307 | 0 | 331,307 | European |
|  |  |  |  |  |  |  | Diastolic blood pressure | Neale B | -0·02593 | 0·003497 | 1·233E-13 | 317,756 | 0 | 317,756 | European |
|  |  |  |  |  |  |  | Forced expiratory volume in 1-second· predicted percentage | Neale B | -0·04149 | 0·005995 | 4·527E-12 | 110,423 | 0 | 110,423 | European |
|  |  |  |  |  |  |  | Height | Neale B | 0·03083 | 0·002448 | 2·308E-36 | 336,474 | 0 | 336,474 | European |
|  |  |  |  |  |  |  | Hip circumference | Neale B | 0·02389 | 0·003412 | 2·504E-12 | 336,601 | 0 | 336,601 | European |
|  |  |  |  |  |  |  | Leg fat-free mass left | Neale B | 0·02329 | 0·002269 | 1·028E-24 | 331,258 | 0 | 331,258 | European |
|  |  |  |  |  |  |  | Leg fat-free mass right | Neale B | 0·02405 | 0·002268 | 2·769E-26 | 331,285 | 0 | 331,285 | European |
|  |  |  |  |  |  |  | Leg predicted mass left | Neale B | 0·02307 | 0·002253 | 1·343E-24 | 331,253 | 0 | 331,253 | European |
|  |  |  |  |  |  |  | Leg predicted mass right | Neale B | 0·02374 | 0·002253 | 5·957E-26 | 331,285 | 0 | 331,285 | European |
|  |  |  |  |  |  |  | Long-standing illness· disability or infirmity | Neale B | 0·0106 | 0·001634 | 8·828E-11 | 329,663 | 107,123 | 222,540 | European |
|  |  |  |  |  |  |  | Sitting height | Neale B | 0·04177 | 0·002662 | 1·848E-55 | 336,172 | 0 | 336,172 | European |
|  |  |  |  |  |  |  | Started insulin within one year diagnosis of diabetes | Neale B | 0·06645 | 0·005098 | 1·233E-38 | 15,397 | 1872 | 13,525 | European |
|  |  |  |  |  |  |  | Trunk fat-free mass | Neale B | 0·02163 | 0·00217 | 2·11E-23 | 331,030 | 0 | 331,030 | European |
|  |  |  |  |  |  |  | Trunk predicted mass | Neale B | 0·02144 | 0·002163 | 3·632E-23 | 330,995 | 0 | 330,995 | European |
|  |  |  |  |  |  |  | Weight | Neale B | 0·02464 | 0·00301 | 2·764E-16 | 336,227 | 0 | 336,227 | European |
|  |  |  |  |  |  |  | Whole body fat-free mass | Neale B | 0·02273 | 0·002179 | 1·73E-25 | 331,291 | 0 | 331,291 | European |
|  |  |  |  |  |  |  | Whole body water mass | Neale B | 0·02267 | 0·002182 | 2·722E-25 | 331,315 | 0 | 331,315 | European |
|  |  |  |  |  |  |  | Schizophrenia | PGC | -0·166 | 0·0183 | 1·163E-19 | 82,315 | 35,476 | 46,839 | Mixed |
|  |  |  | cg16958594 | rs2596495 | C | G | ·· | ·· | ·· | ·· | ·· | ·· | ·· | ·· | ·· |
|  |  |  |  |  |  |  |  |  |  |  |  |  |  |  |  |
| Melanoma | *SPG7* | 16 | cg09560549 | rs8060502 | G | A | ·· | ·· | ·· | ·· | ·· | ·· | ·· | ·· | ·· |
|  |  |  | cg15206445 |  |  |  |  |  |  |  |  |  |  |  |  |

NA: not applicable.

SNPs with *P*>5E-8, and β<0·01 were filtered or marked as "··" in the table

**Supplementary Table 10.** MR analysis used TwoSampleMR package on the causal effect of mtDNA copy number variation on cancer outcomes.

| **Outcome:** | **MR method** | **No. of SNP** | **OR (95% CI)** | ***P*-value** | **Heterogeneity test** | | **Pleiotropy test** | |
| --- | --- | --- | --- | --- | --- | --- | --- | --- |
|  |  |  |  |  | **Cochran's Q** | ***P*** | **intercept** | ***P*** |
| **Overall breast cancer** | MR Egger | 81 | 1·12 (0·89-1·35) | 0·328367047 | 212·84 | 3·17E-14 | -0·0041 | 0·14 |
|  | Weighted median | 81 | 1·00 (0·87-1·13) | 0·991080601 | NA | NA | NA | NA |
|  | Inverse variance weighted | 81 | 0·96 (0·85-1·08) | 0·55502848 | 218·72 | 7·95E-15 | NA | NA |
|  | Simple mode | 81 | 0·92 (0·59-1·25) | 0·612998787 | NA | NA | NA | NA |
|  | Weighted mode | 81 | 1·03 (0·84-1·22) | 0·782069575 | NA | NA | NA | NA |
|  | MR-PRESSO | NA | NA | <0·001 | NA | NA | NA | NA |
|  |  |  |  |  |  |  |  |  |
| **Breast cancer**  **(LuminalA)** | MR Egger | 80 | 1·21 (0·96-1·46) | 0·132752025 | 146·16 | 4·72E-06 | -0·006 | 0·14 |
|  | Weighted median | 80 | 1·14 (0·97-1·30) | 0·127449503 | NA | NA | NA | NA |
|  | Inverse variance weighted | 80 | 0·99 (0·87-1·12) | 0·904620943 | 152·36 | 1·41E-06 | NA | NA |
|  | Simple mode | 80 | 1·10 (0·70-1·50) | 0·630442814 | NA | NA | NA | NA |
|  | Weighted mode | 80 | 1·25 (1·04-1·45) | 0·03594628 | NA | NA | NA | NA |
|  | MR-PRESSO | NA | NA | <0·001 | NA | NA | NA | NA |
|  |  |  |  |  |  |  |  |  |
| **Breast cancer**  **(LuminalB)** | MR Egger | 80 | 0·90 (0·45-1·36) | 0·66848147 | 97·95 | 6·00E-02 | 3·17E-04 | 0·96 |
|  | Weighted median | 80 | 0·81 (0·47-1·16) | 0·24561807 | NA | NA | NA | NA |
|  | Inverse variance weighted | 80 | 0·92 (0·69-1·14) | 0·438122772 | 97·95 | 7·00E-02 | NA | NA |
|  | Simple mode | 80 | 0·67 (0·00-1·48) | 0·337236001 | NA | NA | NA | NA |
|  | Weighted mode | 80 | 0·78 (0·23-1·33) | 0·38272053 | NA | NA | NA | NA |
|  | MR-PRESSO | NA | NA | 0·09 | NA | NA | NA | NA |
|  |  |  |  |  |  |  |  |  |
| **Breast cancer**  **(Her2Negative)** | MR Egger | 80 | 1·13 (0·78-1·47) | 0·505872162 | 77·34 | 0·5 | -0·003 | 0·52 |
|  | Weighted median | 80 | 1·14 (0·87-1·41) | 0·331106708 | NA | NA | NA | NA |
|  | Inverse variance weighted | 80 | 1·02 (0·85-1·19) | 0·832252959 | 77·77 | 0·52 | NA | NA |
|  | Simple mode | 80 | 1·02 (0·50-1·55) | 0·932953491 | NA | NA | NA | NA |
|  | Weighted mode | 80 | 1·16 (0·79-1·53) | 0·440261331 | NA | NA | NA | NA |
|  | MR-PRESSO | NA | NA | 0·42 | NA | NA | NA | NA |
|  |  |  |  |  |  |  |  |  |
| **Breast cancer**  **(Her2Enriched)** | MR Egger | 80 | 0·90 (0·45-1·36) | 0·66848147 | 97·95 | 0·06 | 3·17E-04 | 0·96 |
|  | Weighted median | 80 | 0·81 (0·48-1·14) | 0·222936162 | NA | NA | NA | NA |
|  | Inverse variance weighted | 80 | 0·92 (0·69-1·14) | 0·438122772 | 97·95 | 0·07 | NA | NA |
|  | Simple mode | 80 | 0·67 (0·00-1·50) | 0·351415372 | NA | NA | NA | NA |
|  | Weighted mode | 80 | 0·78 (0·20-1·36) | 0·403849423 | NA | NA | NA | NA |
|  | MR-PRESSO | NA | NA | 0·09 | NA | NA | NA | NA |
|  |  |  |  |  |  |  |  |  |
| **Breast cancer**  **(TNBC)** | MR Egger | 80 | 0·82 (0·12-1·52) | 0·580397766 | 309·29 | 2·73E-29 | 1·68E-05 | 1 |
|  | Weighted median | 80 | 0·95 (0·66-1·24) | 0·739950468 | NA | NA | NA | NA |
|  | Inverse variance weighted | 80 | 0·82 (0·48-1·16) | 0·259188725 | 309·29 | 5·48E-29 | NA | NA |
|  | Simple mode | 80 | 1·21 (0·69-1·72) | 0·476322985 | NA | NA | NA | NA |
|  | Weighted mode | 80 | 0·98 (0·66-1·30) | 0·892521655 | NA | NA | NA | NA |
|  | MR-PRESSO | NA | NA | <0·001 | NA | NA | NA | NA |
|  |  |  |  |  |  |  |  |  |
| **Cervical cancer** | MR Egger | 50 | 1·01 (1·00-1·01) | 0·022820284 | 33·48 | 0·95 | -4·76E-05 | 0·13 |
|  | Weighted median | 50 | 1·00 (1·00-1·01) | 0·013249919 | NA | NA | NA | NA |
|  | Inverse variance weighted | 50 | 1·00 (1·00-1·00) | 0·020433063 | 35·79 | 0·92 | NA | NA |
|  | Simple mode | 50 | 1·00 (1·00-1·01) | 0·392344289 | NA | NA | NA | NA |
|  | Weighted mode | 50 | 1·00 (1·00-1·01) | 0·091991057 | NA | NA | NA | NA |
|  | MR-PRESSO | NA | NA | 0·85 | NA | NA | NA | NA |
|  |  |  |  |  |  |  |  |  |
| **Colorectal cancer** | MR Egger | 88 | 0·96 (0·56-1·37) | 0·860412909 | 148·91 | 3·03E-05 | -1·03E-03 | 0·83 |
|  | Weighted median | 88 | 1·08 (0·85-1·32) | 0·510635323 | NA | NA | NA | NA |
|  | Inverse variance weighted | 88 | 0·93 (0·74-1·12) | 0·438198558 | 148·99 | 3·99E-05 | NA | NA |
|  | Simple mode | 88 | 1·14 (0·69-1·58) | 0·572516887 | NA | NA | NA | NA |
|  | Weighted mode | 88 | 1·07 (0·77-1·37) | 0·652295599 | NA | NA | NA | NA |
|  | MR-PRESSO | NA | NA | <0·001 | NA | NA | NA | NA |
|  |  |  |  |  |  |  |  |  |
| **Endometrial cancer** | MR Egger | 87 | 1·46 (1·06-1·87) | 0·068871525 | 127·75 | 1·87E-03 | -9·91E-03 | 0·05 |
|  | Weighted median | 87 | 1·01 (0·73-1·30) | 0·920987234 | NA | NA | NA | NA |
|  | Inverse variance weighted | 87 | 1·03 (0·82-1·23) | 0·809842734 | 133·69 | 7·59E-04 | NA | NA |
|  | Simple mode | 87 | 1·19 (0·54-1·85) | 0·600712904 | NA | NA | NA | NA |
|  | Weighted mode | 87 | 1·26 (0·85-1·68) | 0·270802515 | NA | NA | NA | NA |
|  | MR-PRESSO | NA | NA | <0·001 | NA | NA | NA | NA |
|  |  |  |  |  |  |  |  |  |
| **Endometrial cancer (endometrioid)** | MR Egger | 87 | 1·46 (1·01-1·91) | 0·105571707 | 115·7 | 0·02 | -9·91E-03 | 0·05 |
|  | Weighted median | 87 | 1·28 (0·96-1·60) | 0·125309077 | NA | NA | NA | NA |
|  | Inverse variance weighted | 87 | 1·09 (0·86-1·31) | 0·483137706 | 118·69 | 0·01 | NA | NA |
|  | Simple mode | 87 | 1·16 (0·48-1·85) | 0·667856089 | NA | NA | NA | NA |
|  | Weighted mode | 87 | 1·32 (0·89-1·74) | 0·209163664 | NA | NA | NA | NA |
|  | MR-PRESSO | NA | NA | 0·004 | NA | NA | NA | NA |
|  |  |  |  |  |  |  |  |  |
| **Endometrial cancer**  **(non-endometrioid)** | MR Egger | 87 | 1·27 (0·19-2·34) | 0·667384265 | 110·35 | 0·03 | -0·02 | 0·22 |
|  | Weighted median | 87 | 0·65 (0·00-1·48) | 0·314911657 | NA | NA | NA | NA |
|  | Inverse variance weighted | 87 | 0·70 (0·16-1·25) | 0·204252321 | 112·36 | 0·03 | NA | NA |
|  | Simple mode | 87 | 0·93 (0·00-2·54) | 0·929828883 | NA | NA | NA | NA |
|  | Weighted mode | 87 | 0·78 (0·00-1·74) | 0·61977419 | NA | NA | NA | NA |
|  | MR-PRESSO | NA | NA | 0·04 | NA | NA | NA | NA |
|  |  |  |  |  |  |  |  |  |
| **Kidney cancer (female)** | MR Egger | 73 | 1·02 (0·00-2·16) | 0·973153973 | 67·26 | 0·6 | 0·01 | 0·3 |
|  | Weighted median | 73 | 0·96 (0·14-1·79) | 0·927890303 | NA | NA | NA | NA |
|  | Inverse variance weighted | 73 | 1·75 (1·23-2·26) | 0·033910609 | 68·34 | 0·6 | NA | NA |
|  | Simple mode | 73 | 0·66 (0·00-2·46) | 0·649971046 | NA | NA | NA | NA |
|  | Weighted mode | 73 | 0·71 (0·00-1·80) | 0·537453516 | NA | NA | NA | NA |
|  | MR-PRESSO | NA | NA | 0·6 | NA | NA | NA | NA |
|  |  |  |  |  |  |  |  |  |
| **Kidney cancer (male)** | MR Egger | 74 | 1·44 (0·40-2·49) | 0·493182348 | 94·39 | 0·04 | -9·18E-03 | 0·46 |
|  | Weighted median | 74 | 1·17 (0·51-1·84) | 0·637939404 | NA | NA | NA | NA |
|  | Inverse variance weighted | 74 | 1·01 (0·54-1·48) | 0·964724683 | 95·12 | 0·04 | NA | NA |
|  | Simple mode | 74 | 2·08 (0·80-3·35) | 0·264835246 | NA | NA | NA | NA |
|  | Weighted mode | 74 | 1·55 (0·67-2·44) | 0·331435192 | NA | NA | NA | NA |
|  | MR-PRESSO | NA | NA | 0·06 | NA | NA | NA | NA |
|  |  |  |  |  |  |  |  |  |
| **Gastric cancer** | MR Egger | 88 | 0·90 (0·37-1·44) | 0·712538895 | 108·49 | 0·05 | 8·10E-04 | 0·89 |
|  | Weighted median | 88 | 0·83 (0·49-1·17) | 0·277217154 | NA | NA | NA | NA |
|  | Inverse variance weighted | 88 | 0·93 (0·70-1·16) | 0·563218227 | 108·51 | 0·06 | NA | NA |
|  | Simple mode | 88 | 0·73 (0·08-1·37) | 0·330436946 | NA | NA | NA | NA |
|  | Weighted mode | 88 | 0·78 (0·39-1·18) | 0·226792423 | NA | NA | NA | NA |
|  | MR-PRESSO | NA | NA | 0·05 | NA | NA | NA | NA |
|  |  |  |  |  |  |  |  |  |
| **Lung cancer** | MR Egger | 61 | 1·32 (0·68-1·95) | 0·39726796 | 74·22 | 0·09 | -6·40E-03 | 0·36 |
|  | Weighted median | 61 | 1·02 (0·67-1·37) | 0·915513048 | NA | NA | NA | NA |
|  | Inverse variance weighted | 61 | 1·01 (0·74-1·27) | 0·957907602 | 75·27 | 0·09 | NA | NA |
|  | Simple mode | 61 | 1·03 (0·27-1·79) | 0·943162343 | NA | NA | NA | NA |
|  | Weighted mode | 61 | 1·05 (0·61-1·50) | 0·826637887 | NA | NA | NA | NA |
|  | MR-PRESSO | NA | NA | 0·11 | NA | NA | NA | NA |
|  |  |  |  |  |  |  |  |  |
| **Melanoma** | MR Egger | 62 | 1·00 (0·99-1·01) | 0·772726602 | 74·1 | 0·1 | -2·61E-05 | 0·71 |
|  | Weighted median | 62 | 1·00 (0·99-1·00) | 0·299735109 | NA | NA | NA | NA |
|  | Inverse variance weighted | 62 | 1·00 (1·00-1·00) | 0·900245477 | 74·28 | 0·12 | NA | NA |
|  | Simple mode | 62 | 1·00 (0·99-1·00) | 0·554349699 | NA | NA | NA | NA |
|  | Weighted mode | 62 | 1·00 (0·99-1·00) | 0·428430484 | NA | NA | NA | NA |
|  | MR-PRESSO | NA | NA | 0·12 | NA | NA | NA | NA |
|  |  |  |  |  |  |  |  |  |
| **Head and neck cancer** | MR Egger | 69 | 2·18 (1·02-3·35) | 0·194363421 | 86·17 | 0·06 | -0·02 | 0·25 |
|  | Weighted median | 69 | 1·41 (0·55-2·27) | 0·430442888 | NA | NA | NA | NA |
|  | Inverse variance weighted | 69 | 1·20 (0·62-1·79) | 0·535181544 | 87·89 | 0·05 | NA | NA |
|  | Simple mode | 69 | 0·55 (0·00-2·22) | 0·490127084 | NA | NA | NA | NA |
|  | Weighted mode | 69 | 1·43 (0·32-2·53) | 0·530983515 | NA | NA | NA | NA |
|  | MR-PRESSO | NA | NA | 0·04 | NA | NA | NA | NA |
|  |  |  |  |  |  |  |  |  |
| **Oralcavity cancer** | MR Egger | 69 | 1·52 (0·12-2·92) | 0·55850569 | 80·41 | 0·13 | -0·005 | 0·75 |
|  | Weighted median | 69 | 1·09 (0·04-2·14) | 0·877555568 | NA | NA | NA | NA |
|  | Inverse variance weighted | 69 | 1·24 (0·55-1·93) | 0·53566438 | 80·53 | 0·14 | NA | NA |
|  | Simple mode | 69 | 0·19 (0·00-2·38) | 0·144224194 | NA | NA | NA | NA |
|  | Weighted mode | 69 | 0·90 (0·00-2·48) | 0·897236616 | NA | NA | NA | NA |
|  | MR-PRESSO | NA | NA | 0·15 | NA | NA | NA | NA |
|  |  |  |  |  |  |  |  |  |
| **Oropharynx cancer** | MR Egger | 69 | 4·04 (2·66-5·42) | 0·051427817 | 70·53 | 0·36 | -0·03 | 0·05 |
|  | Weighted median | 69 | 2·76 (1·69-3·83) | 0·06231003 | NA | NA | NA | NA |
|  | Inverse variance weighted | 69 | 1·23 (0·52-1·93) | 0·573591503 | 74·57 | 0·27 | NA | NA |
|  | Simple mode | 69 | 0·41 (0·00-2·58) | 0·423781305 | NA | NA | NA | NA |
|  | Weighted mode | 69 | 3·44 (2·23-4·65) | 0·049172417 | NA | NA | NA | NA |
|  | MR-PRESSO | NA | NA | 0·2 | NA | NA | NA | NA |
|  |  |  |  |  |  |  |  |  |
| **Ovarian cancer**  **(borderline)** | MR Egger | 88 | 1·25 (0·44-2·06) | 0·586708257 | 145·04 | 7·16E-05 | 4·09E-04 | 0·97 |
|  | Weighted median | 88 | 1·19 (0·69-1·70) | 0·494751951 | NA | NA | NA | NA |
|  | Inverse variance weighted | 88 | 1·27 (0·87-1·68) | 0·243059948 | 145·05 | 9·46E-05 | NA | NA |
|  | Simple mode | 88 | 1·24 (0·13-2·36) | 0·701213105 | NA | NA | NA | NA |
|  | Weighted mode | 88 | 1·24 (0·58-1·91) | 0·519651288 | NA | NA | NA | NA |
|  | MR-PRESSO | NA | NA | <0·001 | NA | NA | NA | NA |
|  |  |  |  |  |  |  |  |  |
| **Ovarian cancer**  **(clear_cell)** | MR Egger | 88 | 0·72 (0·00-1·60) | 0·467050943 | 75·1 | 0·79 | 0·01 | 0·67 |
|  | Weighted median | 88 | 0·48 (0·00-1·16) | 0·034823842 | NA | NA | NA | NA |
|  | Inverse variance weighted | 88 | 0·61 (0·17-1·05) | 0·027911368 | 75·28 | 0·81 | NA | NA |
|  | Simple mode | 88 | 0·91 (0·00-2·40) | 0·897292369 | NA | NA | NA | NA |
|  | Weighted mode | 88 | 0·54 (0·00-1·49) | 0·206106464 | NA | NA | NA | NA |
|  | MR-PRESSO | NA | NA | 0·85 | NA | NA | NA | NA |
|  |  |  |  |  |  |  |  |  |
| **Ovarian cancer**  **(endometrioid)** | MR Egger | 88 | 0·45 (0·00-1·17) | 0·030393583 | 113·18 | 0·03 | 8·84E-03 | 0·03 |
|  | Weighted median | 88 | 0·76 (0·27-1·26) | 0·283531292 | NA | NA | NA | NA |
|  | Inverse variance weighted | 88 | 0·92 (0·55-1·29) | 0·645824883 | 119·96 | 0·01 | NA | NA |
|  | Simple mode | 88 | 0·61 (0·00-1·70) | 0·372946914 | NA | NA | NA | NA |
|  | Weighted mode | 88 | 0·43 (0·00-1·14) | 0·022333742 | NA | NA | NA | NA |
|  | MR-PRESSO | NA | NA | 0·008 | NA | NA | NA | NA |
| **Ovarian cancer**  **(epithelial)** | MR Egger | 88 | 2·06 (1·36-2·76) | 0·046839603 | 110·97 | 0·04 | 8·63E-03 | 0·22 |
|  | Weighted median | 88 | 1·78 (1·28-2·27) | 0·022401475 | NA | NA | NA | NA |
|  | Inverse variance weighted | 88 | 1·41 (1·05-1·76) | 0·057999825 | 112·92 | 0·03 | NA | NA |
|  | Simple mode | 88 | 1·67 (0·68-2·66) | 0·313047127 | NA | NA | NA | NA |
|  | Weighted mode | 88 | 1·80 (1·18-2·41) | 0·067456249 | NA | NA | NA | NA |
|  | MR-PRESSO | NA | NA | 0·03 | NA | NA | NA | NA |
|  |  |  |  |  |  |  |  |  |
| **Ovarian cancer**  **(high-grade_serous)** | MR Egger | 88 | 0·81 (0·27-1·35) | 0·44638853 | 125·14 | 3·76E-03 | 6·62E-03 | 0·72 |
|  | Weighted median | 88 | 0·86 (0·47-1·25) | 0·442624237 | NA | NA | NA | NA |
|  | Inverse variance weighted | 88 | 0·88 (0·61-1·15) | 0·358285156 | 125·32 | 4·50E-03 | NA | NA |
|  | Simple mode | 88 | 0·78 (0·00-1·64) | 0·57154999 | NA | NA | NA | NA |
|  | Weighted mode | 88 | 0·87 (0·33-1·40) | 0·600110691 | NA | NA | NA | NA |
|  | MR-PRESSO | NA | NA | 0·008 | NA | NA | NA | NA |
|  |  |  |  |  |  |  |  |  |
| **Ovarian cancer (invasive_epithelial)** | MR Egger | 88 | 0·79 (0·34-1·24) | 0·300388364 | 208·71 | 3·37E-12 | 5·89E-03 | 0·29 |
|  | Weighted median | 88 | 1·06 (0·83-1·29) | 0·624199786 | NA | NA | NA | NA |
|  | Inverse variance weighted | 88 | 0·97 (0·75-1·20) | 0·808527984 | 211·45 | 2·33E+12 | NA | NA |
|  | Simple mode | 88 | 1·05 (0·59-1·51) | 0·83239221 | NA | NA | NA | NA |
|  | Weighted mode | 88 | 1·05 (0·78-1·32) | 0·714682518 | NA | NA | NA | NA |
|  | MR-PRESSO | NA | NA | <0·001 | NA | NA | NA | NA |
|  |  |  |  |  |  |  |  |  |
| **Ovarian cancer**  **(low-grade_serous)** | MR Egger | 88 | 0·74 (0·00-1·77) | 0·562992134 | 81·31 | 0·62 | 6·51E-03 | 0·61 |
|  | Weighted median | 88 | 0·65 (0·00-1·47) | 0·310840779 | NA | NA | NA | NA |
|  | Inverse variance weighted | 88 | 0·93 (0·41-1·45) | 0·779496772 | 81·58 | 0·64 | NA | NA |
|  | Simple mode | 88 | 0·24 (0·00-1·84) | 0·083141021 | NA | NA | NA | NA |
|  | Weighted mode | 88 | 0·58 (0·00-1·71) | 0·347403875 | NA | NA | NA | NA |
|  | MR-PRESSO | NA | NA | 0·62 | NA | NA | NA | NA |
|  |  |  |  |  |  |  |  |  |
| **Ovarian cancer**  **(mucinous)** | MR Egger | 88 | 1·29 (0·63-1·95) | 0·45355519 | 82·38 | 0·59 | 8·09E-03 | 0·42 |
|  | Weighted median | 88 | 1·28 (0·80-1·75) | 0·309595557 | NA | NA | NA | NA |
|  | Inverse variance weighted | 88 | 1·02 (0·69-1·35) | 0·911990923 | 83·02 | 0·6 | NA | NA |
|  | Simple mode | 88 | 1·72 (0·67-2·76) | 0·312783765 | NA | NA | NA | NA |
|  | Weighted mode | 88 | 1·44 (0·86-2·03) | 0·224380919 | NA | NA | NA | NA |
|  | MR-PRESSO | NA | NA | 0·63 | NA | NA | NA | NA |
|  |  |  |  |  |  |  |  |  |
| **Ovarian cancer**  **(serous_invasive)** | MR Egger | 88 | 0·82 (0·28-1·36) | 0·468201537 | 221·38 | 6·86E-14 | 4·95E-03 | 0·45 |
|  | Weighted median | 88 | 1·11 (0·83-1·39) | 0·461181678 | NA | NA | NA | NA |
|  | Inverse variance weighted | 88 | 0·98 (0·71-1·25) | 0·875075412 | 222·83 | 7·08E-14 | NA | NA |
|  | Simple mode | 88 | 1·40 (0·84-1·97) | 0·237754714 | NA | NA | NA | NA |
|  | Weighted mode | 88 | 1·15 (0·83-1·48) | 0·398223421 | NA | NA | NA | NA |
|  | MR-PRESSO | NA | NA | <0·001 | NA | NA | NA | NA |
|  |  |  |  |  |  |  |  |  |
| **Pancreatic cancer** | MR Egger | 88 | 0·87 (0·03-1·71) | 0·748012904 | 98·02 | 0·18 | 6·68E-04 | 0·95 |
|  | Weighted median | 88 | 1·28 (0·68-1·89) | 0·422110111 | NA | NA | NA | NA |
|  | Inverse variance weighted | 88 | 0·89 (0·48-1·31) | 0·591994267 | 98·03 | 0·2 | NA | NA |
|  | Simple mode | 88 | 1·36 (0·07-2·64) | 0·641627638 | NA | NA | NA | NA |
|  | Weighted mode | 88 | 1·53 (0·66-2·40) | 0·341115477 | NA | NA | NA | NA |
|  | MR-PRESSO | NA | NA | 0·18 | NA | NA | NA | NA |
|  |  |  |  |  |  |  |  |  |
| **Prostate cancer** | MR Egger | 88 | 1·34 (1·06-1·63) | 0·047408286 | 229·92 | 4·60E-15 | -4·66E-03 | 0·19 |
|  | Weighted median | 88 | 1·06 (0·91-1·21) | 0·427930503 | NA | NA | NA | NA |
|  | Inverse variance weighted | 88 | 1·14 (0·99-1·28) | 0·084211578 | 234·57 | 1·72E-15 | NA | NA |
|  | Simple mode | 88 | 1·06 (0·77-1·36) | 0·689770287 | NA | NA | NA | NA |
|  | Weighted mode | 88 | 1·08 (0·89-1·26) | 0·433115323 | NA | NA | NA | NA |
|  | MR-PRESSO | NA | NA | <0·001 | NA | NA | NA | NA |
|  |  |  |  |  |  |  |  |  |
| **Thyroid cancer** | MR Egger | 88 | 0·88 (0·01-1·76) | 0·783651333 | 89·13 | 0·39 | 4·00E-03 | 0·68 |
|  | Weighted median | 88 | 0·79 (0·17-1·42) | 0·46899325 | NA | NA | NA | NA |
|  | Inverse variance weighted | 88 | 1·04 (0·60-1·47) | 0·866915168 | 89·3 | 0·41 | NA | NA |
|  | Simple mode | 88 | 0·70 (0·00-1·87) | 0·552596459 | NA | NA | NA | NA |
|  | Weighted mode | 88 | 0·75 (0·00-1·53) | 0·474708344 | NA | NA | NA | NA |
|  | MR-PRESSO | NA | NA | 0·47 | NA | NA | NA | NA |

NA: not applicable.

Cochran Q statistic implemented in MR Egger and IVW method, *P*>0·05 indicates no heterogeneity exists.

The intercept of MR Egger can be used to indicate whether directional horizontal pleiotropy is driving the results of MR analysis, there are no directional pleiotropies if *P* >0·05.

MR-PRESSO can detect and adjust for any outliers reflecting horizontal pleiotropic biases, where *p* value for Global test >0·05 indicates no horizontal pleiotropic outliers.

**Supplementary Table 11.** MR analysis used TwoSampleMR package on the causal effect of cancers on mtDNA copy number variation.

| **Exposure:** | **MR method** | **No. of SNP** | **OR (95% CI)** | ***P*-value** | **Heterogeneity test** | | **Pleiotropy test** | |
| --- | --- | --- | --- | --- | --- | --- | --- | --- |
|  |  |  |  |  | **Cochran's Q** | ***P*** | **intercept** | ***P*** |
| **Overall breast cancer** | MR Egger | 152 | 1·01 (0·99-1·02) | 0·328367047 | 273·28 | 3·18E-09 | 5·97E-04 | 0·88 |
|  | Weighted median | 152 | 1·00 (0·99-1·01) | 0·991080601 | NA | NA | NA | NA |
|  | Inverse variance weighted | 152 | 1·00 (1·00-1·01) | 0·55502848 | 273·32 | 4·29E-09 | NA | NA |
|  | Simple mode | 152 | 1·00 (0·98-1·03) | 0·612998787 | NA | NA | NA | NA |
|  | Weighted mode | 152 | 1·00 (0·99-1·02) | 0·782069575 | NA | NA | NA | NA |
|  | MR-PRESSO | NA | NA | <0·001 | NA | NA | NA | NA |
|  |  |  |  |  |  |  |  |  |
| **Breast cancer**  **(LuminalA)** | MR Egger | 110 | 1·00 (0·99-1·01) | 0·740230698 | 168·98 | 1·60E-04 | 6·11E-04 | 0·79 |
|  | Weighted median | 110 | 1·00 (0·99-1·01) | 0·69003811 | NA | NA | NA | NA |
|  | Inverse variance weighted | 110 | 1·00 (0·99-1·01) | 0·841680957 | 169·1 | 1·99E-04 | NA | NA |
|  | Simple mode | 110 | 1·00 (0·98-1·02) | 0·811452803 | NA | NA | NA | NA |
|  | Weighted mode | 110 | 1·00 (0·99-1·01) | 0·641237243 | NA | NA | NA | NA |
|  | MR-PRESSO | NA | NA | <0·001 | NA | NA | NA | NA |
|  |  |  |  |  |  |  |  |  |
| **Breast cancer**  **(LuminalB)** | MR Egger | 18 | 1·02 (1·00-1·04) | 0·66848147 | 16·56 | 0·41 | -2·52E-03 | 0·2 |
|  | Weighted median | 18 | 1·00 (0·99-1·01) | 0·24561807 | NA | NA | NA | NA |
|  | Inverse variance weighted | 18 | 1·00 (1·00-1·01) | 0·438122772 | 18·38 | 0·37 | NA | NA |
|  | Simple mode | 18 | 1·00 (0·98-1·01) | 0·337236001 | NA | NA | NA | NA |
|  | Weighted mode | 18 | 1·00 (0·98-1·01) | 0·38272053 | NA | NA | NA | NA |
|  | MR-PRESSO | NA | NA | 0·37 | NA | NA | NA | NA |
|  |  |  |  |  |  |  |  |  |
| **Breast cancer**  **(Her2Negative)** | MR Egger | 19 | 1·00 (0·98-1·02) | 0·960886547 | 12·87 | 0·74 | 2·41E-04 | 0·87 |
|  | Weighted median | 19 | 1·00 (0·99-1·01) | 0·867736701 | NA | NA | NA | NA |
|  | Inverse variance weighted | 19 | 1·00 (0·99-1·01) | 0·621533216 | 12·9 | 0·8 | NA | NA |
|  | Simple mode | 19 | 1·01 (0·99-1·03) | 0·552238175 | NA | NA | NA | NA |
|  | Weighted mode | 19 | 1·00 (0·98-1·01) | 0·662475478 | NA | NA | NA | NA |
|  | MR-PRESSO | NA | NA | 0·78 | NA | NA | NA | NA |
|  |  |  |  |  |  |  |  |  |
| **Breast cancer**  **(Her2Enriched)** | MR Egger | 4 | 1·00 (0·95-1·05) | 0·989571392 | 0·88 | 0·64 | -1·23E-03 | 0·84 |
|  | Weighted median | 4 | 0·99 (0·98-1·01) | 0·300169295 | NA | NA | NA | NA |
|  | Inverse variance weighted | 4 | 0·99 (0·98-1·01) | 0·303612943 | 0·93 | 0·82 | NA | NA |
|  | Simple mode | 4 | 0·99 (0·97-1·01) | 0·39375419 | NA | NA | NA | NA |
|  | Weighted mode | 4 | 0·99 (0·97-1·01) | 0·404972898 | NA | NA | NA | NA |
|  | MR-PRESSO | NA | NA | 0·92 | NA | NA | NA | NA |
|  |  |  |  |  |  |  |  |  |
| **Breast cancer**  **(TNBC)** | MR Egger | 17 | 0·94 (0·87-1·02) | 0·183053572 | 197·36 | 7·29E-34 | 3·93E-03 | 0·52 |
|  | Weighted median | 17 | 0·98 (0·97-1·00) | 0·058395691 | NA | NA | NA | NA |
|  | Inverse variance weighted | 17 | 0·97 (0·94-1·00) | 0·032404523 | 203·03 | 1·94E-34 | NA | NA |
|  | Simple mode | 17 | 0·98 (0·96-1·01) | 0·231370705 | NA | NA | NA | NA |
|  | Weighted mode | 17 | 0·98 (0·96-1·01) | 0·167750164 | NA | NA | NA | NA |
|  | MR-PRESSO | NA | NA | <0·001 | NA | NA | NA | NA |
|  |  |  |  |  |  |  |  |  |
| **Colorectal cancer** | MR Egger | 26 | 1·06 (0·98-1·13) | 0·154084308 | 95·5 | 1·74E-10 | -7·20E-03 | 0·08 |
|  | Weighted median | 26 | 1·00 (0·99-1·02) | 0·887046706 | NA | NA | NA | NA |
|  | Inverse variance weighted | 26 | 0·99 (0·97-1·01) | 0·257551859 | 108·44 | 2·29E-12 | NA | NA |
|  | Simple mode | 26 | 1·01 (0·98-1·03) | 0·513694885 | NA | NA | NA | NA |
|  | Weighted mode | 26 | 1·01 (0·99-1·02) | 0·476119511 | NA | NA | NA | NA |
|  | MR-PRESSO | NA | NA | <0·001 | NA | NA | NA | NA |
|  |  |  |  |  |  |  |  |  |
| **Endometrial cancer** | MR Egger | 16 | 1·04 (0·99-1·09) | 0·11577545 | 24·55 | 0·04 | -5·09E-03 | 0·13 |
|  | Weighted median | 16 | 1·01 (0·99-1·02) | 0·301202294 | NA | NA | NA | NA |
|  | Inverse variance weighted | 16 | 1·00 (0·99-1·02) | 0·700133862 | 29·2 | 0·02 | NA | NA |
|  | Simple mode | 16 | 0·99 (0·97-1·02) | 0·657744023 | NA | NA | NA | NA |
|  | Weighted mode | 16 | 1·01 (0·99-1·03) | 0·398000501 | NA | NA | NA | NA |
|  | MR-PRESSO | NA | NA | 0·019 | NA | NA | NA | NA |
|  |  |  |  |  |  |  |  |  |
| **Endometrial cancer (endometrioid)** | MR Egger | 11 | 1·04 (0·96-1·12) | 0·343366466 | 20·99 | 0·01 | -6·29E-03 | 0·28 |
|  | Weighted median | 11 | 1·00 (0·98-1·01) | 0·673502619 | NA | NA | NA | NA |
|  | Inverse variance weighted | 11 | 0·99 (0·98-1·01) | 0·536209225 | 24·06 | 7·46E-03 | NA | NA |
|  | Simple mode | 11 | 0·99 (0·97-1·02) | 0·624423033 | NA | NA | NA | NA |
|  | Weighted mode | 11 | 1·00 (0·98-1·02) | 0·974731872 | NA | NA | NA | NA |
|  | MR-PRESSO | NA | NA | 0·004 | NA | NA | NA | NA |
|  |  |  |  |  |  |  |  |  |
| **Kidney cancer (female)** | MR Egger | 3 | 1·00 (0·99-1·00) | 0·402923312 | 0·05 | 0·82 | 5·70E-03 | 0·35 |
|  | Weighted median | 3 | 1·00 (0·995-1·003) | 0·7009869 | NA | NA | NA | NA |
|  | Inverse variance weighted | 3 | 1·00 (0·995-1·004) | 0·99513983 | 2·69 | 0·26 | NA | NA |
|  | Simple mode | 3 | 1·00 (0·99-1·00) | 0·693357183 | NA | NA | NA | NA |
|  | Weighted mode | 3 | 1·00 (0·99-1·00) | 0·728417725 | NA | NA | NA | NA |
|  | MR-PRESSO | NA | NA | NA | NA | NA | NA | NA |
|  |  |  |  |  |  |  |  |  |
| **Kidney cancer (male)** | MR Egger | 8 | 1·00 (0·99-1·00) | 0·549954461 | 2·35 | 0·89 | 1·35E-03 | 0·52 |
|  | Weighted median | 8 | 1·00 (0·997-1·004) | 0·950863929 | NA | NA | NA | NA |
|  | Inverse variance weighted | 8 | 1·00 (0·997-1·003) | 0·894466082 | 2·81 | 0·9 | NA | NA |
|  | Simple mode | 8 | 1·00 (0·99-1·00) | 0·430432803 | NA | NA | NA | NA |
|  | Weighted mode | 8 | 1·00 (0·995-1·003) | 0·882421733 | NA | NA | NA | NA |
|  | MR-PRESSO | NA | NA | 0·91 | NA | NA | NA | NA |
|  |  |  |  |  |  |  |  |  |
| **Gastric cancer** | MR Egger | 8 | 0·99 (0·96-1·03) | 0·707752072 | 10·99 | 0·09 | 1·19E-03 | 0·73 |
|  | Weighted median | 8 | 1·00 (0·98-1·01) | 0·807931012 | NA | NA | NA | NA |
|  | Inverse variance weighted | 8 | 1·00 (0·99-1·01) | 0·870038067 | 11·23 | 0·13 | NA | NA |
|  | Simple mode | 8 | 1·00 (0·97-1·02) | 0·773260235 | NA | NA | NA | NA |
|  | Weighted mode | 8 | 1·00 (0·98-1·01) | 0·890170703 | NA | NA | NA | NA |
|  | MR-PRESSO | NA | NA | 0·11 | NA | NA | NA | NA |
|  |  |  |  |  |  |  |  |  |
| **Lung cancer** | MR Egger | 4 | 0·96 (0·84-1·09) | 0·615149527 | 17·16 | 1·88E-04 | 0·01 | 0·49 |
|  | Weighted median | 4 | 1·01 (0·99-1·02) | 0·334587871 | NA | NA | NA | NA |
|  | Inverse variance weighted | 4 | 1·01 (0·98-1·04) | 0·34294477 | 23·33 | 3·45E-05 | NA | NA |
|  | Simple mode | 4 | 1·00 (0·99-1·02) | 0·594555087 | NA | NA | NA | NA |
|  | Weighted mode | 4 | 1·01 (0·99-1·02) | 0·437999902 | NA | NA | NA | NA |
|  | MR-PRESSO | NA | NA | 0·03 | NA | NA | NA | NA |
|  |  |  |  |  |  |  |  |  |
| **Melanoma** | MR Egger | 6 | 12·99 (7·23-18·76) | 0·432506855 | 22·52 | 1·58E-04 | -4·35E-03 | 0·49 |
|  | Weighted median | 6 | 2·00 (0·47-3·54) | 0·374398817 | NA | NA | NA | NA |
|  | Inverse variance weighted | 6 | 1·74 (0·00-4·23) | 0·660801244 | 25·82 | 9·69E-05 | NA | NA |
|  | Simple mode | 6 | 6·71 (2·84-10·57) | 0·378612628 | NA | NA | NA | NA |
|  | Weighted mode | 6 | 2·13 (0·06-4·20) | 0·506768419 | NA | NA | NA | NA |
|  | MR-PRESSO | NA | NA | 0·002 | NA | NA | NA | NA |
|  |  |  |  |  |  |  |  |  |
| **Head and neck cancer** | Wald ratio | 1 | 0·98 (0·96-0·99) | 1·35E-03 | NA | NA | NA | NA |
|  |  |  |  |  |  |  |  |  |
| **Oropharynx cancer** | Wald ratio | 1 | 0·97 (0·96-0·98) | 1·06E-08 | NA | NA | NA | NA |
|  |  |  |  |  |  |  |  |  |
| **Ovarian cancer**  **(borderline)** | MR Egger | 8 | 1·00 (0·95-1·06) | 0·879911168 | 50·99 | 2·98E-09 | 4·89E-03 | 0·5 |
|  | Weighted median | 8 | 1·00 (0·99-1·02) | 0·470326925 | NA | NA | NA | NA |
|  | Inverse variance weighted | 8 | 1·02 (1·00-1·04) | 0·036433048 | 55·3 | 1·30E-09 | NA | NA |
|  | Simple mode | 8 | 1·01 (0·99-1·02) | 0·390501616 | NA | NA | NA | NA |
|  | Weighted mode | 8 | 1·00 (0·99-1·01) | 0·869406867 | NA | NA | NA | NA |
|  | MR-PRESSO | NA | NA | <0·001 | NA | NA | NA | NA |
|  |  |  |  |  |  |  |  |  |
| **Ovarian cancer**  **(clear cell)** | Wald ratio | 1 | 1·00 (0·98-1·02) | 0·793872573 | NA | NA | NA | NA |
|  |  |  |  |  |  |  |  |  |
| **Ovarian cancer**  **(epithelial)** | MR Egger | 4 | 1·05 (0·66-1·45) | 0·823496838 | 53·09 | 2·97E-12 | -6·47E-03 | 0·9 |
|  | Weighted median | 4 | 1·00 (0·99-1·02) | 0·77465714 | NA | NA | NA | NA |
|  | Inverse variance weighted | 4 | 1·02 (0·98-1·07) | 0·364914998 | 53·67 | 1·32E-11 | NA | NA |
|  | Simple mode | 4 | 0·99 (0·97-1·01) | 0·530329153 | NA | NA | NA | NA |
|  | Weighted mode | 4 | 1·00 (0·98-1·01) | 0·636168686 | NA | NA | NA | NA |
|  | MR-PRESSO | NA | NA | <0·001 | NA | NA | NA | NA |
|  |  |  |  |  |  |  |  |  |
| **Ovarian cancer (invasive_epithelial)** | MR Egger | 12 | 0·98 (0·83-1·13) | 0·828283131 | 275·99 | 1·82E-53 | 6·15E-04 | 0·95 |
|  | Weighted median | 12 | 1·01 (0·99-1·03) | 0·320914329 | NA | NA | NA | NA |
|  | Inverse variance weighted | 12 | 0·99 (0·93-1·04) | 0·647924206 | 276·09 | 9·42E-53 | NA | NA |
|  | Simple mode | 12 | 1·01 (0·99-1·03) | 0·380059767 | NA | NA | NA | NA |
|  | Weighted mode | 12 | 1·01 (0·99-1·03) | 0·260909938 | NA | NA | NA | NA |
|  | MR-PRESSO | NA | NA | <0·001 | NA | NA | NA | NA |
|  |  |  |  |  |  |  |  |  |
| **Ovarian cancer**  **(mucinous)** | MR Egger | 5 | 0·95 (0·9-0·99) | 0·104206173 | 1·74 | 0·63 | 0·02 | 0·1 |
|  | Weighted median | 5 | 1·00 (0·99-1·01) | 0·789082922 | NA | NA | NA | NA |
|  | Inverse variance weighted | 5 | 1·00 (0·99-1·01) | 0·89779554 | 7·35 | 0·12 | NA | NA |
|  | Simple mode | 5 | 1·01 (0·99-1·03) | 0·452156729 | NA | NA | NA | NA |
|  | Weighted mode | 5 | 1·01 (0·99-1·03) | 0·45889389 | NA | NA | NA | NA |
|  | MR-PRESSO | NA | NA | 0·15 | NA | NA | NA | NA |
|  |  |  |  |  |  |  |  |  |
| **Ovarian cancer**  **(serous_invasive)** | MR Egger | 16 | 0·97 (0·89-1·06) | 0·556719615 | 254·89 | 2·80E-46 | 3·15E-03 | 0·65 |
|  | Weighted median | 16 | 1·01 (0·99-1·02) | 0·424410132 | NA | NA | NA | NA |
|  | Inverse variance weighted | 16 | 0·99 (0·96-1·03) | 0·642500517 | 258·72 | 1·98E-46 | NA | NA |
|  | Simple mode | 16 | 1·00 (0·99-1·02) | 0·643094676 | NA | NA | NA | NA |
|  | Weighted mode | 16 | 1·00 (0·99-1·01) | 0·819483627 | NA | NA | NA | NA |
|  | MR-PRESSO | NA | NA | <0·001 | NA | NA | NA | NA |
|  |  |  |  |  |  |  |  |  |
| **Prostate cancer** | MR Egger | 132 | 1·00 (0·99-1·02) | 0·470823211 | 277·96 | 8·92E-13 | -7·02E-05 | 0·91 |
|  | Weighted median | 132 | 1·00 (0·99-1·01) | 0·93117307 | NA | NA | NA | NA |
|  | Inverse variance weighted | 132 | 1·00 (1·00-1·01) | 0·216832098 | 277·99 | 1·31E-12 | NA | NA |
|  | Simple mode | 132 | 1·00 (0·98-1·02) | 0·973365616 | NA | NA | NA | NA |
|  | Weighted mode | 132 | 1·00 (0·99-1·01) | 0·951323395 | NA | NA | NA | NA |
|  | MR-PRESSO | NA | NA | <0·001 | NA | NA | NA | NA |
|  |  |  |  |  |  |  |  |  |
| **Thyroid cancer** | MR Egger | 3 | 1·03 (1·01-1·04) | 0·231263767 | 1·07 | 0·3 | -0·01 | 0·22 |
|  | Weighted median | 3 | 1·00 (1·00-1·01) | 0·400509398 | NA | NA | NA | NA |
|  | Inverse variance weighted | 3 | 1·00 (0·99-1·01) | 0·964371803 | 9·4 | 9·09E-03 | NA | NA |
|  | Simple mode | 3 | 1·00 (0·99-1·01) | 0·846176061 | NA | NA | NA | NA |
|  | Weighted mode | 3 | 1·00 (1·00-1·01) | 0·358355473 | NA | NA | NA | NA |
|  | MR-PRESSO | NA | NA | NA | NA | NA | NA | NA |

NA: not applicable.

Cochran Q statistic implemented in MR Egger and IVW method, *P*>0·05 indicates no heterogeneity exists.

The intercept of MR Egger can be used to indicate whether directional horizontal pleiotropy is driving the results of MR analysis, there are no directional pleiotropies if *P* >0·05.

MR-PRESSO can detect and adjust for any outliers reflecting horizontal pleiotropic biases, where *p* value for Global test >0·05 indicates no horizontal pleiotropic outliers.

**Supplementary Figure 1.** Leave-one-out sensitivity analyses of the SNPs represented the expression mitochondrial-related genes and breast cancer outcome.


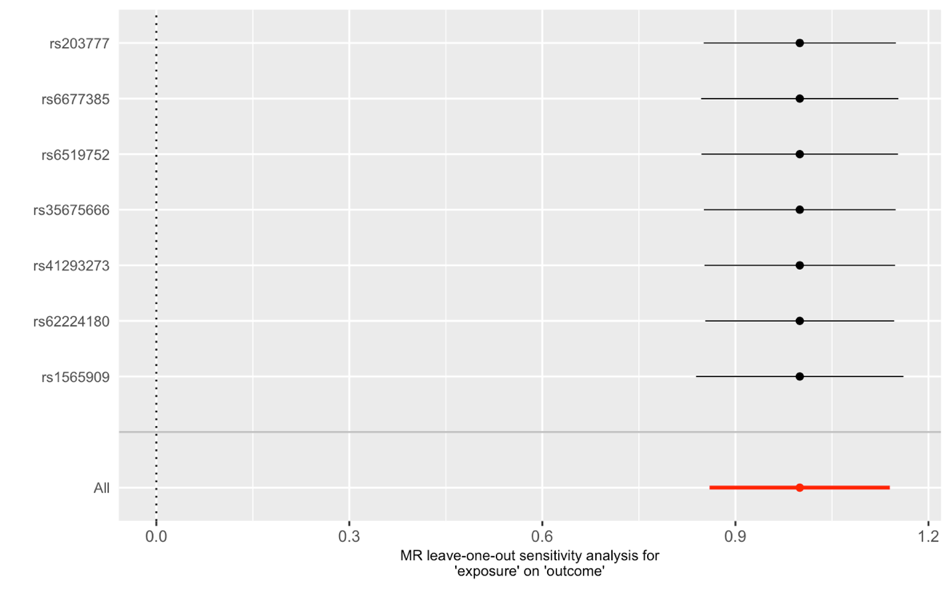


The estimated causal effect is shown for each excluded SNP and the overall estimate using all the SNPs is shown in red. The error bars represent the 95% confidence intervals.

**Supplementary Figure 2.** Leave-one-out sensitivity analyses of the SNPs represented the expression of mitochondrial-related genes and prostate cancer outcome.


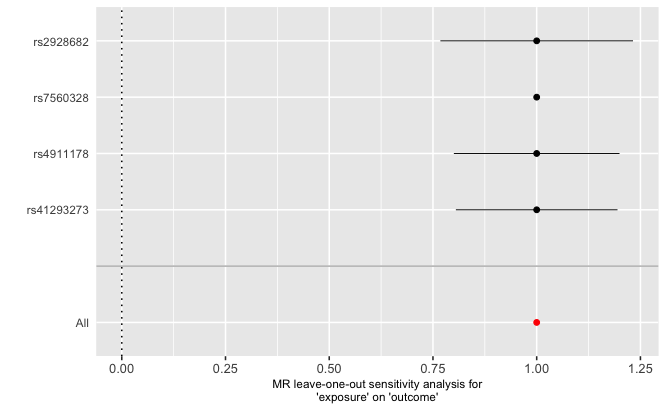


The estimated causal effect is shown for each excluded SNP and the overall estimate using all the SNPs is shown in red. The error bars represent the 95% confidence intervals.

**Supplementary Figure 3.** Mendelian randomization results for the association between expression of mitochondrial-related genes and breast cancer risk classified by intrinsic–like subtypes.

^a^ Represents the effect size (β) of a variant on mRNA expressions. β>0 means positive association and β<0 means negative association.

^b^ ‘Colocalization’ indicates PP.H4 between eQTLs and cancer outcomes. PP.H4 > 0·8 is the well-applied cut-off for the evidence of colocalization.

**Supplementary Figure 4.** Leave-one-out sensitivity analyses of the SNPs represented the DNA methylation of mitochondrial-related genes and breast cancer outcome.


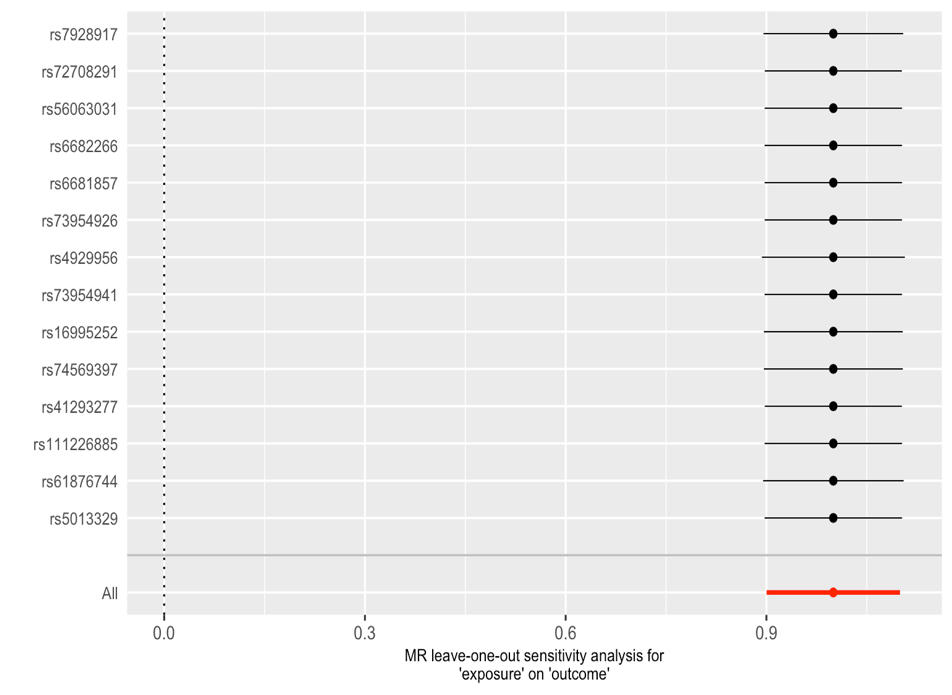


The estimated causal effect is shown for each excluded SNP and the overall estimate using all the SNPs is shown in red. The error bars represent the 95% confidence intervals.

**Supplementary Figure 5.** Leave-one-out sensitivity analyses of the SNPs represented the DNA methylation of mitochondrial-related genes and prostate cancer outcome.


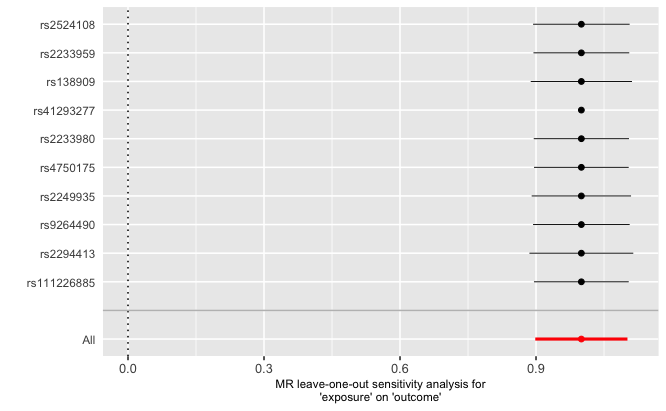


The estimated causal effect is shown for each excluded SNP and the overall estimate using all the SNPs is shown in red. The error bars represent the 95% confidence interval.

**Supplementary Figure 6.** Mendelian randomization results for the association between randomization results for the association between DNA methylation of mitochondrial-related and luminal A-like breast cancer risk.

^a^ Represents the effect size (β) of a variant on DNA methylation. β>0 means positive association and β<0 means negative association.

^b^ ‘Colocalization’ indicates PP.H4 between mQTLs and cancer outcomes. PP.H4 > 0·8 is the well-applied cut-off for the evidence of colocalization.

**Supplementary Figure 7.** Results of SNPs and SMR associations across mQTL, eQTL and breast and prostate cancer GWAS.

The top plot shows −log10(*P*-values) of SNPs from the A. breast cancer GWAS, B. prostate cancer GWAS for *NSUN4* (ENSG00000117481). The red diamonds and blue circles represent −log10 (*P*-values) from SMR tests for associations of gene expression and methylation probes with cancer, respectively. The solid diamonds and circles are the probes not rejected by the HEIDI test. The yellow star indicates the variant rs41293273 that represented the expression of NSUN4 in both breast and prostate cancer. The second plot shows −log10 (*P*-values) of the SNP association for the NSUN4 gene expression from the eQTL study. The third to fifth plot shows −log10 (*P*-values) of shared SNP associations for methylation probes for both breast and prostate cancer from the mQTL study. C. Prioritizing genes around the *NSUN4* locus for both breast and prostate cancer.

**Supplementary Figure 8.** Manhattan plots show the phenome-wide scan results of genetic variants in *NSUN4*.

A.

B.

C.

D.

E.

F.

G.

Phenome-wide scan plot for A. rs41293273. B. rs6682266. C. rs5013329. D. rs41293277. E. rs56063031. F. rs6681857. G. rs111226885. Disease related traits are plotted on the x-axis and negative log-base-10 of the *P* value are showed on the y-axis. The grey line indicates genome-wide significant threshold (α) of *P*=5 × 10^− 8^.

**Supplementary Figure 9.** Manhattan plots show the phenome-wide scan results of genetic variants in *FDPS* and *NUDT5*.

A.

B.

A. Phenome-wide scan plot for rs6677385. B. Phenome-wide scan plot for rs4750175. Disease related traits are plotted on the x-axis and negative log-base-10 of the *P* value are showed on the y-axis. The grey line indicates genome-wide significant threshold (α) of *P*=5 × 10^− 8^.

**Supplementary Figure 10.** Manhattan plots show the phenome-wide scan results of genetic variants in *VARS2*.

A.

B.

C.

Phenome-wide scan plot for A. rs2523593. B. rs2596500. C. rs2596495. Disease related traits are plotted on the x-axis and negative log-base-10 of the *P* value are showed on the y-axis. The grey line indicates genome-wide significant threshold (α) of *P*=5 × 10^− 8^.

**References**

1. Zhang H, Ahearn TU, Lecarpentier J, Barnes D, Beesley J, Qi G, et al. Genome-wide association study identifies 32 novel breast cancer susceptibility loci from overall and subtype-specific analyses. Nat Genet. 2020;52(6):572-81.

2. Backman JD, Li AH, Marcketta A, Sun D, Mbatchou J, Kessler MD, et al. Exome sequencing and analysis of 454,787 UK Biobank participants. Nature. 2021;599(7886):628-34.

3. Lyon MS, Andrews SJ, Elsworth B, Gaunt TR, Hemani G, Marcora E. The variant call format provides efficient and robust storage of GWAS summary statistics. Genome Biol. 2021;22(1):32.

4. Sakaue S, Kanai M, Tanigawa Y, Karjalainen J, Kurki M, Koshiba S, et al. A cross-population atlas of genetic associations for 220 human phenotypes. Nat Genet. 2021;53(10):1415-24.

5. O'Mara TA, Glubb DM, Amant F, Annibali D, Ashton K, Attia J, et al. Identification of nine new susceptibility loci for endometrial cancer. Nat Commun. 2018;9(1):3166.

6. Laskar RS, Muller DC, Li P, Machiela MJ, Ye Y, Gaborieau V, et al. Sex specific associations in genome wide association analysis of renal cell carcinoma. Eur J Hum Genet. 2019;27(10):1589-98.

7. Trepo E, Caruso S, Yang J, Imbeaud S, Couchy G, Bayard Q, et al. Common genetic variation in alcohol-related hepatocellular carcinoma: a case-control genome-wide association study. Lancet Oncol. 2022;23(1):161-71.

8. Wang Y, McKay JD, Rafnar T, Wang Z, Timofeeva MN, Broderick P, et al. Rare variants of large effect in BRCA2 and CHEK2 affect risk of lung cancer. Nat Genet. 2014;46(7):736-41.

9. Lesseur C, Diergaarde B, Olshan AF, Wunsch-Filho V, Ness AR, Liu G, et al. Genome-wide association analyses identify new susceptibility loci for oral cavity and pharyngeal cancer. Nat Genet. 2016;48(12):1544-50.

10. Phelan CM, Kuchenbaecker KB, Tyrer JP, Kar SP, Lawrenson K, Winham SJ, et al. Identification of 12 new susceptibility loci for different histotypes of epithelial ovarian cancer. Nat Genet. 2017;49(5):680-91.

11. Schumacher FR, Al Olama AA, Berndt SI, Benlloch S, Ahmed M, Saunders EJ, et al. Association analyses of more than 140,000 men identify 63 new prostate cancer susceptibility loci. Nat Genet. 2018;50(7):928-36.

12. Pillalamarri V, Shi W, Say C, Yang S, Lane J, Guallar E, et al. Whole-exome sequencing in 415,422 individuals identifies rare variants associated with mitochondrial DNA copy number. HGG Adv. 2023;4(1):100147.

13. Davies NM, Holmes MV, Davey Smith G. Reading Mendelian randomisation studies: a guide, glossary, and checklist for clinicians. BMJ. 2018;362:k601.

14. Zhu Z, Zhang F, Hu H, Bakshi A, Robinson MR, Powell JE, et al. Integration of summary data from GWAS and eQTL studies predicts complex trait gene targets. Nat Genet. 2016;48(5):481-7.

15. Genomes Project C, Auton A, Brooks LD, Durbin RM, Garrison EP, Kang HM, et al. A global reference for human genetic variation. Nature. 2015;526(7571):68-74.

16. Wu Y, Zeng J, Zhang F, Zhu Z, Qi T, Zheng Z, et al. Integrative analysis of omics summary data reveals putative mechanisms underlying complex traits. Nat Commun. 2018;9(1):918.

17. Higgins JP, Thompson SG, Deeks JJ, Altman DG. Measuring inconsistency in meta-analyses. BMJ. 2003;327(7414):557-60.

18. Bowden J, Davey Smith G, Burgess S. Mendelian randomization with invalid instruments: effect estimation and bias detection through Egger regression. Int J Epidemiol. 2015;44(2):512-25.

19. Burgess S, Thompson SG. Interpreting findings from Mendelian randomization using the MR-Egger method. Eur J Epidemiol. 2017;32(5):377-89.

20. Verbanck M, Chen CY, Neale B, Do R. Detection of widespread horizontal pleiotropy in causal relationships inferred from Mendelian randomization between complex traits and diseases. Nat Genet. 2018;50(5):693-8.

21. Burgess S, Bowden J, Fall T, Ingelsson E, Thompson SG. Sensitivity Analyses for Robust Causal Inference from Mendelian Randomization Analyses with Multiple Genetic Variants. Epidemiology. 2017;28(1):30-42.

22. Burgess S, Small DS, Thompson SG. A review of instrumental variable estimators for Mendelian randomization. Stat Methods Med Res. 2017;26(5):2333-55.

23. Giambartolomei C, Vukcevic D, Schadt EE, Franke L, Hingorani AD, Wallace C, et al. Bayesian test for colocalisation between pairs of genetic association studies using summary statistics. PLoS Genet. 2014;10(5):e1004383.
